# Rigid and concave, 2,4-*cis*-substituted azetidine derivatives: A platform for asymmetric catalysis

**DOI:** 10.1038/s41598-018-24784-3

**Published:** 2018-04-25

**Authors:** Akina Yoshizawa, Antonio Feula, Louise Male, Andrew G. Leach, John S. Fossey

**Affiliations:** 1 School of Chemistry, University of Birmingham, Edgbaston, Birmingham, West Midlands B15 2TT UK; 2 X-Ray Crystallography Facility, School of Chemistry, University of Birmingham, Edgbaston, Birmingham, West Midlands B15 2TT UK; 3 School of Pharmacy and Biomolecular Sciences, Liverpool John Moores University, Byrom Street, Liverpool, L3 3AF UK

## Abstract

A series of single enantiomer, 2,4-*cis-*disubstituted amino azetidines were synthesised and used as ligands for copper-catalysed Henry reactions of aldehydes with nitromethane. Optimisation of ligand substituents and the reaction conditions was conducted. The enantiomeric excess of the formed products was highest when alkyl aldehydes were employed in the reaction (>99% e.e.). The absolute stereochemistry of one representative azetidine derivative salt was determined by analysis of the Flack parameter of an XRD single crystal structure. The origin of selectivity in catalysis was investigated computationally, revealing the importance of the amino-substituent in determining the stereochemical outcome. A racemic platinum complex of a *cis*-disubstituted azetidine is examined by XRD single crystal structure analysis with reference to its steric parameters, and analogies to the computationally determined copper complex catalyst are drawn. A preliminary example of the use of a *cis*-disubstituted azetidine scaffold in thiourea H-bonding catalyst is noted in the supporting information.

## Introduction

**Figure 1:**
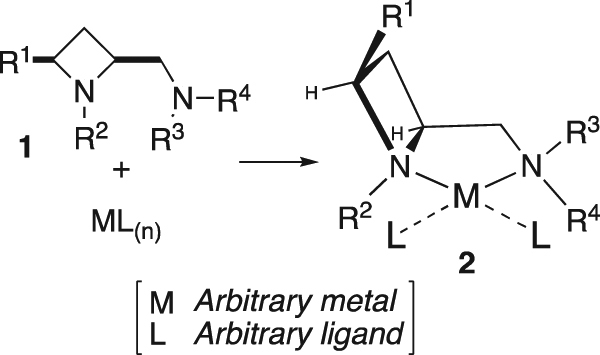
Arbitrary depiction of the complexation of **1** with a square-planar metal to give complex **2**.

Enzymes are often excellent catalysts that are able to achieve very high levels of stereoselectivity. One property of enzymes that has been implicated in their capacity to deliver highly stereoselective reaction outcomes is that their active sites are concave, well-defined, cavities^1^. When flexible arrays of ligands are arranged around a metal centre they can be constrained into similar concave shapes but few ligands are inherently concave in a rigid fashion. Upon surveying the crystal structures of some cis-azetidine derivatives it was reasoned that the amino-azetidine scaffold might be one such ligand. The *cis*-ring geometry of ligand **1** makes it inherently concave and in the chelation complex **2**, the R^1^-substiuent should point over the metal (Fig. 1) and offer a rigid platform to potentially strongly influence the stereoselectivity of an asymmetric reaction catalysed by such a complex. As a result of an on-going interest in the synthesis of nitrogen-containing heterocycles^2–7^, co-authors of this report have identified a protocol for delivery of 2,4-*cis*-disubstituted azetidine derivatives as single diastereoisomers^8,9^.

**Figure 2:**
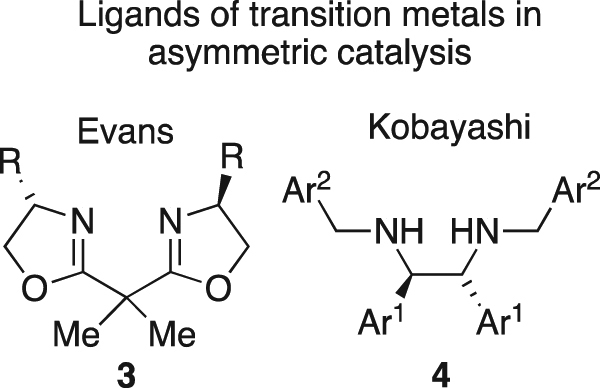
*C*_2_-Symmetric bisoxazoline (**3**) and diamine (**4**) ligands.

Chelating-*N*,*N*′ *C*_2_-symmetric ligands are well-developed as stereoselectivity-engendering motifs in asymmetric catalysis, following the introduction of anionic semicorrin and related *N*,*N*′-ligands by Pfaltz^10^ and others^11^. Corey *et al*. employed a neutral analogue (**3**) as a ligand for an iron(III)-catalysed Diels-Alder reaction^12^. Evans and co-workers deployed **3** and related ligands for copper-catalysed transformations^13^. This work deeply impacted the field of asymmetric catalysis, providing a platform for both asymmetric reaction development and understanding of the underlying principles of asymmetric Lewis acid catalysis^14–16^. *C*_2_-symmetric *N*,*N*′-chelating bis-amine (**4**) and bis-imine ligands (Fig. 2) have been popularised by Kobayashi and co-workers (Including research co-authored by one of the authors of this report)^17–21^, for catalysis of a range of asymmetric transformations. Whilst ligands and complexes displaying *C*_2_-symmetry offer some advantages, including ease of synthetic access, the virtues of *C*_1_-symmetric asymmetric catalysts remain strong, with numerous reports detailing and contrasting them^22–26^.

Reasoning that the shape of the chiral pocket described by a *cis*-disubstituted azetidine *N*,*N*′-ligand has the potential to offer a unique *C*_1_-symmetric catalytic platform, and that access to single enantiomer azetidines has already been established, we posed the question: Can 2,4-*cis*-disubstituted azetidine *N*,*N*′-ligands be developed to deliver high enantiomeric excess in transition metal-catalysed carbon-carbon bond forming reactions?

**Figure 3:**
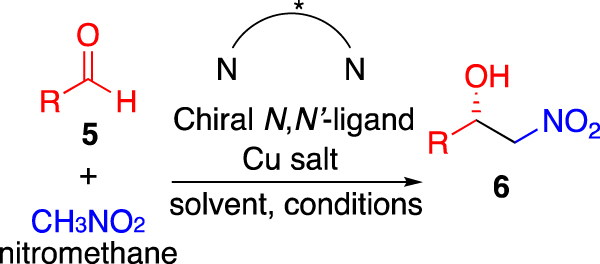
General scheme for the additon of nitromethane to an aldehyde (**5**) under control of a chiral diamine ligated copper catalyst, to furnish a nitro-aldol adduct (**6**).

The stereochemical utility of *cis*-disubstituted azetidines **1** may be probed by examining their use as ligands in asymmetric catalysis, as such the copper-catalysed Henry reaction was selected for this investigation (Fig. 3)^27^. *C*_2_-Symmetric^28–35^, and *C*_1_-symmetric *N*,*N*′-ligands^36,37^ have previously been use to engender asymmetry in the Henry reaction^38–42^. Therefore, the Henry reaction is ideal for demonstrating tractability and stereochemical scope of **1**-type ligands in asymmetric catalysis.

## Results and Discussion

**Figure 4:**
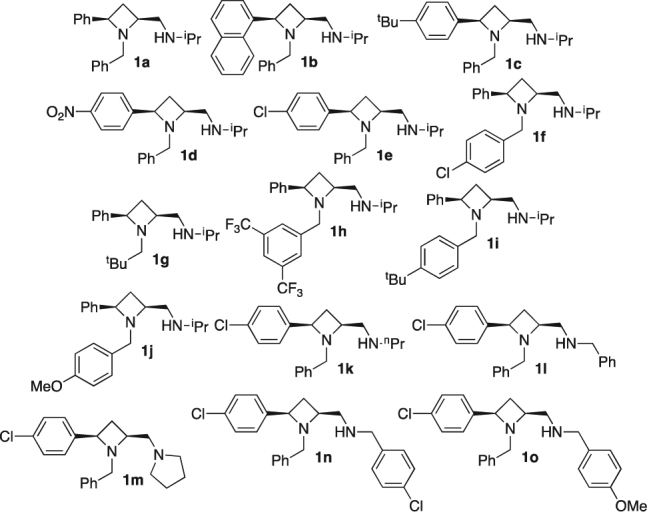
Single enantiomer azetidines **1a-o** deployed as ligands for asymmetric copper-catalysed Henry reactions.

In order to evaluate and optimise the stereochemical induction potential of *cis*-disubstituted azetidines **1** in the Henry reaction of Fig. 3, a total of fifteen single enantiomer ligands, **1a-o** (Fig. 4), were prepared^8^ (For full details see supporting information).

**Figure 5:**
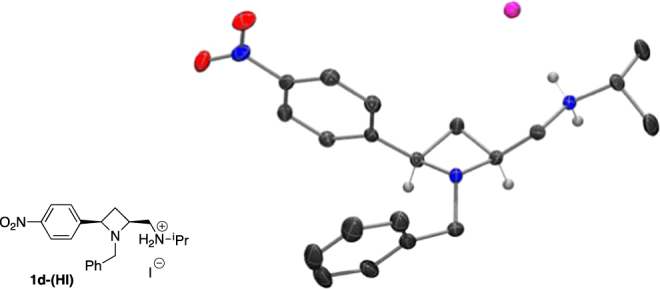
Chemical structure (left) and crystal structure (right) of (**2*S*, 4*R***) **1d**, Flack parameter -0.017(2) thus confirming stereochemical assignment.

The relative (*cis*) and absolute stereochemistry of the HI salt of ligand **1d** were determined by single crystal X-ray diffraction structure analysis (Fig. 5), and reference to the Flack parameter determined as −0.017(2), thus confirming (2*S*, 4*R*) stereochemistry^43^.

Since simple alkyl amine derivatives of **1** (positions R^3^ and R^4^, Fig. 1) were chronologically first available to this project as single enantiomers, azetidine **1a** was selected for initial investigation (Table 1, entry 1). In the reaction of *para*-nitrobenzaldehyde (**5a**) with nitromethane 5 mol% loading of a 1:1 mixture of copper(II)acetate.(H_2_O) and ligand **1a** mixture, a promising 95% conversion and 35% e.e. of **6a** was obtained. Increasing the ring substituent size from phenyl to 1-naphthyl (**1b**) resulted in lower conversion and racemic product (Table 1, entry 2, 86% conv., 1% e.e.). Similarly adding a tertiary butyl group to the azetidine’s phenyl substituent (**1c**) also lowered the conversion and gave very low enantiomeric excess in contrast to the first ligand tested (Table 1, entry 3, 90% conv., 9% e.e.; *versus* entry 1). Electron withdrawing substituents 4-nitro- (**1d**, Table 1, entry 4) and 4-chloro- (**1e**, Table 1, entry 5) on the ring substituent phenyl group showed good conversion (95 and 96% respectively). Only 25% e.e. in **6a** was obtained with **1d** as ligand, whereas an improved e.e. of 47% was observed for use of **1e** (Table 1, entries 4 and 5). Thus, the 4-chlorophenyl derivative is the most effective 2-position substituent among those initially tested (**1e**). Switching the 4-chloro- and phenyl substituents (R^2^ and R^3^, Table 1, entry 6 **1f**, *versus* entry 5 **1e**), gave slightly lower conversion (82%) and e.e. (32%). Changing the aldehyde to benzaldehyde (**5b**) in the reaction facilitated by ligand **1a** (Table 1, entry 7) gave a lower conversion than using 4-nitrobenzaldehyde (**5a**), which is expected on electronic grounds (**5a** being more prone to nucleophilic attack than **5b**); pleasingly, the enantiomeric excess increased to 77%. Both ligands **1e** and **1f** also resulted in forming catalysts capable of delivering product **6a** with similar outcomes, Table 1, entries 8 (88% conv., 71% e.e.) and 9 (77% conv., 77% e.e.) respectively. Changing position R^3^ to tertiary butyl (**1g**), 3,5-bis-trifluoromethyl-phenyl (**1h**), 4-tertiary butyl-phenyl (**1i**) and 4-methoxy-phenyl (**1j**) (Table 1, entries 10 to 13) did not improve the conversion or e.e. beyond that observed in Table 1, entry 7. Based on these results ligand **1e** was retained for further study.

**Table 1:** Benzaldehyde (**5a**) and para-nitro-benzaldehyde (**5b**) as substrates in the Henry reaction employing ligands **1a–j**, furnish products **6a** and **b**.


**Entry**	**Aldehyde R** ^**1**^	**Ligand**	**R** ^**2**^	**R** ^**3**^	**Conv./%**	**E.E./%**
1	NO_2_	**1a**	Ph	Ph	95	35
2	NO_2_	**1b**	1-Naphth	Ph	86	1
3	NO_2_	**1c**	4-*t*Bu-C_6_H_4_	Ph	90	9
4	NO_2_	**1d**	4-NO_2_-C_6_H_4_	Ph	96	25
5	NO_2_	**1e**	4-Cl-C_6_H_4_	Ph	95	47
6	NO_2_	**1f**	Ph	4-Cl-C_6_H_4_	82	32
7	H	**1a**	Ph	Ph	78	77
8	H	**1e**	4-Cl-C_6_H_4_	Ph	88	71
9	H	**1f**	Ph	4-Cl-C_6_H_4_	77	77
10	H	**1g**	Ph	*t*-Bu	36	3
11	H	**1h**	Ph	3,5-(CF_3_)_2_-C_6_H_3_	48	14
12	H	**1i**	Ph	4-*t*-Bu-C_6_H_4_	67	48
13	H	**1j**	Ph	4-MeO-C_6_H_4_	69	65

Choosing the addition of nitromethane to benzaldehyde (**5b**) and employing ligand **1e** various copper sources were next investigated (Table 2, entries 1 to 10). To confirm a ligand accelerated reaction is occurring and no background reaction is compromising enantiomeric excess, the following reactions were performed: (i) in the absence of ligand **1e** and copper source; (ii) in the presence of 5 mol% ligand **1e** and the absence of a copper source; and (iii) in the absence of ligand **1e** and in the presence of a copper source (Cu(OAc)_2_·H_2_O, 5 mol%); Table 2 entries 1, 2 and 3 respectively. After 16 hours, at room temperature product (**6b**) was not observed in all three cases, confirming no background or ligand-free metal catalysed reactions are leading to measurable amounts of product under the standard conditions used. Upon comparing catalyst loading (1 mol% and 10 mol%; Table 2 entries 4 and 6 respectively, *versus* 5 mol% entry 5), a lower loading reduced the rate of product formation (21% conversion after 48 h), whilst increasing the loading offered no observed advantage, as such 5 mol% loading was employed for all subsequent copper-catalysed Henry reactions in this report. Copper(I)acetate as a metal source (Table 2 entry 7) offered no advantage in terms of conversion to product **6b** and lowered the enantiomeric excess (compared to Table 2 entry 5). The use of CuSO_4_.5H_2_O, CuCl_2_.2H_2_O, and Cu(OTf)_2_ as copper sources (Table 2 entries 8, 9 and 10 respectively) failed to deliver any product **6b**. Next the effect of solvent was investigated (Table 2 entries 11 to 17, *versus* entry 5). Methanol and *iso*-propanol gave 96 and 78% conversion (Table 2 entry 11 and 12 respectively), both delivered product **6b** in 32% e.e. Tetrahydrofuran and diethyl ether both gave essentially racemic product in 43 and 73% conversion (Table 2 entries 13 and 14 respectively). Toluene offered no advantage (Table 2 entry 15; 46% conversion and 39% e.e.). Dichloromethane and acetonitrile gave acceptable conversions, Table 2 entries 16 (88%) and 17 (86%) respectively, whilst enantiomeric excess was low (21% and 7% respectively). The initial, literature-informed selections of Cu(OAc)_2_·H_2_O as the copper source, and ethanol as the solvent are confirmed as superior. As expected, the enantioselectivity follows a temperature dependant trend, across a 0 to 60 °C (Table 2 entries, 18, 5, 19 and 20; 0 °C, 18 °C, 40 °C and 60 °C respectively). The highest enantiomeric excess, of 79%, was observed at the lowest temperature (0 °C, entry 18) although 48 h was required to reach 93% conversion. As expected the highest temperature gave an improved conversion but compromised the enantioselectivity somewhat (98% conversion and 51% e.e., entry 20).

**Table 2:** The Henry reaction of **5b** with ligand **1e** and **a** range of copper sources, to furnish **6b**.


**Entry**	**Copper Source**	**Loading/X mol%**	**Solvent**	**Temp./°C**	**Time/h**	**Conv./%**	**E.E./%**
1	None	0	EtOH	18	16	0	—
2	None	5 **1e** only	EtOH	18	16	0	—
3	Cu(OAc)_2_·H_2_O	5 no ligand	EtOH	18	16	0	—
4	Cu(OAc)_2_·H_2_O	1	EtOH	18	48	21	—
5	Cu(OAc)_2_·H_2_O	5	EtOH	18	16	88	71
6	Cu(OAc)_2_·H_2_O	10	EtOH	18	16	83	62
7	Cu(OAc)	5	EtOH	18	16	82	55
8	CuSO_4_·5H_2_O	5	EtOH	18	16	0	—
9	CuCl_2_·2H_2_O	5	EtOH	18	16	0	—
10	Cu(OTf)_2_	5	EtOH	18	16	0	—
11	Cu(OAc)_2_·H_2_O	5	MeOH	18	16	96	32
12	Cu(OAc)_2_·H_2_O	5	*i-*PrOH	18	16	78	32
13	Cu(OAc)_2_·H_2_O	5	THF	18	16	43	6
14	Cu(OAc)_2_·H_2_O	5	Et_2_O	18	16	73	5
15	Cu(OAc)_2_·H_2_O	5	Toluene	18	16	46	39
16	Cu(OAc)_2_·H_2_O	5	CH_2_Cl_2_	18	16	88	21
17	Cu(OAc)_2_·H_2_O	5	CH_3_CN	18	16	86	7
18	Cu(OAc)_2_·H_2_O	5	EtOH	0	48	93	79
19	Cu(OAc)_2_·H_2_O	5	EtOH	40	16	93	64
20	Cu(OAc)_2_·H_2_O	5	EtOH	60	16	98	51

The results displayed in Tables 1 and 2 lead to the interim conclusion that optimal conditions determined thus far, when the exocyclic amine = -NH*i*Pr, (and with reference to substituent numbering of Fig. 1) are: R^1^ = 4-chloro-phenyl; R^2^ = benzyl; ethanol as solvent; and Cu(OAc)_2_·H_2_O as metal source. Thus, for further screening and optimisation, the same solvent and metal source were retained at 5 mol% catalyst loading. Reactions were run for 16 hours at room temperature (18 °C, for convenience, whilst recognising that e.e. may be improved by lowering the reaction temperature and extending the time). Comparing the use of *iso*-propylamine functionalised ligand **1e** (Table 1, entries 5 and 8) in the reactions of aldehydes **5a** and **5b** to those mediated by the *n*-propylamine congener (Table 3, entries 1 and 2) there are no obvious advantages or differences between branched (**1e**) and linear (**1k**) secondary alkyl amine functionalities. Whereas the *N*-benzyl secondary amine analogue (**1l**) offered an increased enantiomeric excess for the same reactions (Table 3, entries 3 and 4), where products **6a** and **6b** were obtained in 81% and 87% e.e. respectively. Tertiary amine derivative **1m** (pyrrolidine substituent **1m**, Table 3, entries 5 and 6) gave *circa*. 80% conversion but the enantiomeric excesses of products **6a** and **6b** were only 5 and 28% respectively. Having now identified the *N*-benzyl derivative **1l** as the best ligand to this point, electron -poor (**1n** 4-chlorobenzyl) and -rich ligand analogues (**1o** 4-methoxybenzyl) were compared in the catalysed formation of **6b** (Table 3, entry 4, ligand **1l** *versus* entries 7 and 8, ligands **1n** and **1o** respectively). Among these three ligands compared, the electron-rich analogue **1o** was inferior (64% conversion and 66% e.e.). Ligand **1n** was also slightly inferior to ligand **1l** in the same **6b**-forming reaction. Ligand **1l** was therefore identified as the *best* of the ligands prepared in this report and used to demonstrate substrate scope (Table 4).

**Table 3:** Benzaldehyde (**5a**) and para-nitro-benzaldehyde (**5b**) as substrates in the Henry reaction employing ligands **1k–o**, furnish products **6a** and **b**.


Entry	R^1^	Ligand	R^2^	R^3^	Conv./%	E.E./%
1	NO_2_	**1k**	*n-*Pr	H	86	65
2	H	**1k**	*n*-Pr	H	87	74
3	NO_2_	**1l**	Bn	H	92	81
4	H	**1l**	Bn	H	86	87
5	NO_2_	**1m**	*N*-Pyrrolidine		80	5
6	H	**1m**	*N*-Pyrrolidine		78	28
7	H	**1n**	4-Cl-Bn	H	74	83
8	H	**1o**	4-MeO-Bn	H	64	66

**Table 4:** Reaction of **5a–j** in the Henry reaction employing ligands **1l**, furnish products **6a–j**.


**Entry**	**R**	**Product**	**AlogP**	**Mol Wt**	**Conv./%**	E.E./%
1	4-NO_2_-C_6_H_4_	**6a**	0.905	212.16	98	91
2	Ph	**6b**	0.997	167.16	91	95
3	4-MeO-C_6_H_4_	**6c**	1.005	197.19	66	92
4	4-Cl-C_6_H_4_	**6d**	1.650	201.61	95	95
5	4-Me-C_6_H_4_	**6e**	1.305	181.19	73	93
6	4-*t-*Bu-C_6_H_4_	**6f**	2.294	223.27	85	93
7	1-Naphth	**6g**	2.150	217.22	83	93
8	2-Ph-C_6_H_4_	**6h**	2.663	243.26	85	86
9	**Cy**	**6i**	1.204	173.21	**98**	**>99**
10	***t-*** **Bu**	**6j**	0.670	147.17	**99**	**>99**

Since the Henry reaction between aldehydes and nitromethane is a widely used protocol for the delivery of 2-nitro-ethanol derivatives, which are in turn often reductively transformed into corresponding methylene amino alcohols, for use in biology-facing applications, the addition of nitromethane to ten aldehydes under optimal conditions was investigated (Substrate scope in the nucleophile part was not investigated in this study. Future work will include investigation of diastereoselectivity of nitromethylene derivatives. Since amino-methylation is an important reaction in the synthesis of drug-like molecules it is a suitable reaction to assess the capacity of *cis*-azetidines to deliver asymmetric products in this first study). Recognising that ligand **1l** gave slightly higher conversion than ligand **1e** at room temperature, for laboratory operational ease, 50 h reactions at 0 °C were conducted in anticipation of achieving good to high yield across the set.

Under the optimised conditions, aldehydes **5a** and **5b** (Table 4, entries 1 and 2 respectively) provided the corresponding products **6a** and **6b** in 98% and 91% conversion and 91% and 95% enantiomeric excess respectively. When 4-methoxybenzaldehyde **5c** was subjected to the optimised reaction conditions (Table 4, entry 3) only 66% conversion to product **6c** was observed, whilst enantiomeric excess was a reasonable 92%. The use of electron-poor 4-chlorobenzaldehyde (**5d**) resulted in good conversion and enantiomeric excess in product **6d** (95% conv., 95% e.e., Table 4, entry 4). Benzaldehyde derivatives bearing 4-alkyl substituents (**5e** 4-methyl and **5f** 4-*tert*-butyl, Table 4, entry 5 and entry 6 respectively) gave acceptable conversions of 73% and 85% respectively, enantiomeric excesses were on a par with the preceding four table entries (**6e** and **6f** both 93% e.e.). Aldehyde **5g**, 1-naphthylaldehyde (Table 4, entry 7), gave 83% conversion and 93% enantiomeric excess. Since this is similar to the level of conversion and enantiomeric excess when **5f** was employed (4-*tert*-butyl-phenyl aldehyde, Table 4, entry 6), an extended aromatic surface, an additional ring, offers no divergence from an additional bulky alkyl group suggesting steric parameters rather than π-interactions may lie at the origin of the observed selectivity. To test this hypothesis alkyl aldehydes cyclohexyl aldehyde **5i** and pivaldehyde **5j** were tested (Table 4, entries 9 and 10 respectively). To our delight the reactions to form **6i** and **6j** proceeded with excellent conversion and in excellent enantiomeric excess, Table 4, entry 9 and 10 (**6i**, 98% conv., >99% e.e. and **6j**, 99% conv., >99% e.e. respectively), thus confirming π-interactions are not required in order to achieve high conversion and selectivity.

According to analysis using the LLAMA web tool^44^, from the University of Leeds (UK), this small set of products, **6a-j** all lie within *Lipinski-space*, molecular weight less than 500 and logP less than 5, furthermore 5 of the 10 products accessed fall within *lead-like space*. Whilst far from exhaustive in substrate scope this does confirm the reactions giving good to excellent enantiomeric excess for C-C bond forming reactions are giving rise to lead-like compounds that may be of utility in drug discovery. Future work in this area will include direct access to libraries of enantiopure small molecules by extending substrate scope in both nucleophile and electrophile and developing novel asymmetric transformations facilitated by the *cis*-*substituted* azetidine scaffold. In order to achieve these ambitions a deeper understanding of the stereochemical influence of the ligand-metal manifold is required, as such a stereochemical rationale was next sought through transition state modelling.

### Stereochemical rationale and transition state modelling

**Figure 6:**
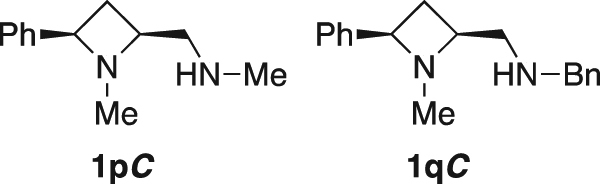
Structure of ligands **1pC** and **1qC** investigated computationally in this study.

**Figure 7:**
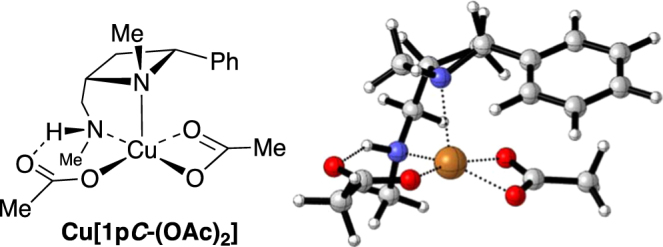
Representations of the minimised structure of a copper(II)acetate derived complex of **1pC**.

To explore the properties of the ligands and their metal complexes, quantum mechanical calculations were undertaken. Previous studies have shown that the M06-2×/6-31G* (with the LANL basis set and *pseudo*-potential on copper) level of theory is suitable for studying the copper catalysed Henry reaction and have described the structures of minima and transition states for the process^45^. These protocols have been adapted for use in this study of 2,4-*cis*-di*substituted* azetidine ligands **1** in the asymmetric copper-catalysed Henry reaction of this study (All calculations were performed in Gaussian09 (Gaussian 09, Revision A.02). Minima and transition states were characterised by calculation of vibrational frequencies)^46–50^. A model azetidine-containing ligand (**1p*C***, Fig. 6), with reduced complexity, is deployed in the initial calculations with copper(II)acetate as the metal source, using the aforementioned protocol, and structures of this complex were obtained by editing structures optimised by Das *et al*.^51^, resulting in the computed structure given in Fig. 7.

**Figure 8:**
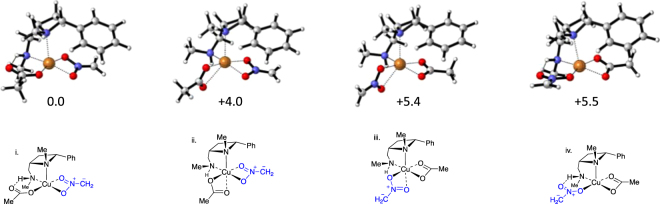
Four minima and free energies in kcal/mol for the complexes calculated to be formed upon exchange of an acetate from **1pC** with a nitromethane anion.

After ligand complexation, the next step along the reaction coordinate is for one or other of the acetate ligands of the formed complex to be exchanged with an anion of nitromethane, several ligand arrangements were considered. These calculations revealed that the lowest energy conformation (Fig. 8, i) is where the right hand acetate group from Fig. 7 (as drawn) is replaced with nitromethane anion, and the left hand (as drawn) acetate retains a H-bonding interaction with the ligand’s NH proton. All other conformations (Fig. 8, ii, iii and iv) involve loss of the hydrogen bond between acetate and the NH of the azetidine ligand and are more than 4 kcal/mol higher in energy. This hydrogen bonding interaction with an acetate is a key influence that ensures a significant preference for replacing only one specific acetate ligand. When the ligand conformation is altered such that the NHMe presents a methyl group towards the acetate rather than a proton, the energy increases by 4.0 kcal/mol (Fig. 8, ii) and a six-coordinate *pseudo*-octahedral geometry on the copper centre is imposed by a now bidentate acetate-copper interaction. When the nitromethane anion replaces the hydrogen bonding acetate the *pseudo*-octahedral geometry and hydrogen bonded geometries (Fig. 7, acetate on left side as drawn resulting in structures depicted as Fig. 8 iii and iv) have the same energy suggesting that the nitromethane anion forms a weaker hydrogen bond (than acetate), which is approximately equivalent to its interaction with metal. Alternative conformations including those with variations in the azetidine ring were all prohibitively high in energy and result in the azetidine dissociating from the copper. The acetate group has a strong preference for forming a copper-oxygen bond and a hydrogen bond and this dictates a preference for displacing the acetate lacking a hydrogen bond with nitromethane anion. The cyclic binding mode for a ligand containing an O = X-O^−^ (X = C-R or S(O)-R) functionality is in congruence with analogous complexes (featuring sulfonyl groups) previously described by calculations and observed in XRD single crystal structures^51–56^.

**Figure 9:**
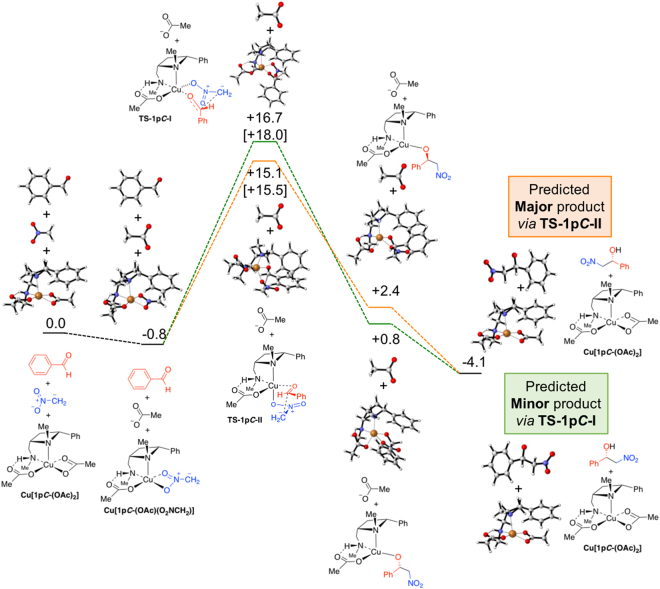
Calculated reaction coordinates and free energies in kcal/mol for the 1pC-catalyed reaction of benzaldehyde (**5b**) with the anion of nitromethane. Two diastereomeric transition states leading to opposite enantiomers of product **6b** depicted, free energies in parenthesis include solvation in THF.

Having established a strong preference for replacing one acetate group and for a single backbone conformation of the azetidine ligand, with some variation permitted in the positioning of the benzyl group on the exocyclic nitrogen atom, transition states for the reaction between the complex of nitromethane anion and benzaldehyde were investigated. For these initial calculations azetidine ligand **1p*C*** was used, resulting in the overall profile depicted in Fig. 9 ^51^.

**Figure 10:**
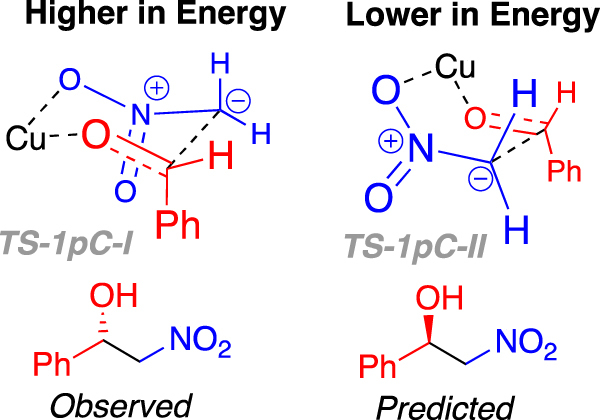
Drawings highlighting the axially substituted six-memebered chair-like transition state TS-1pC-I (left upper) and the eclipsed boat-like transition state (TS-1qC-II), and the products to which the lead (lower images respectively). The disconnect between the experimentally observed and the computationally predicted products, using this model, are highlighted.

Importantly, the model system described in Fig. 9 incorrectly predicts the stereochemical outcome observed experimentally (**1a-o**). The higher energy of the two transition states depicted as **TS1-1p*C*-I** (Fig. 9) adopts a six-membered chair-like orientation, with the phenyl group of the benzaldehyde derived part in an axial position, this higher transition state would lead to the experimentally observed (*S*) product (Fig. 10, left). Whereas the computationally predicted outcome, in this case, resulting from the lower energy transition state **TS1-1p*C*-II** (Fig. 9), which adopts a boat-like orientation, would be the incorrect (*R*) enantiomer (Fig. 10, right). Placing a phenyl group in the equatorial position in **TS1-1p*C*-I** is not possible because this space is filled by the phenyl of ligand **1p*C***. Clearly a feature not considered up to this point is critical in determining the stereochemical outcome of **1**-ligated copper-catalysed Henry reactions.

**Figure 11:**
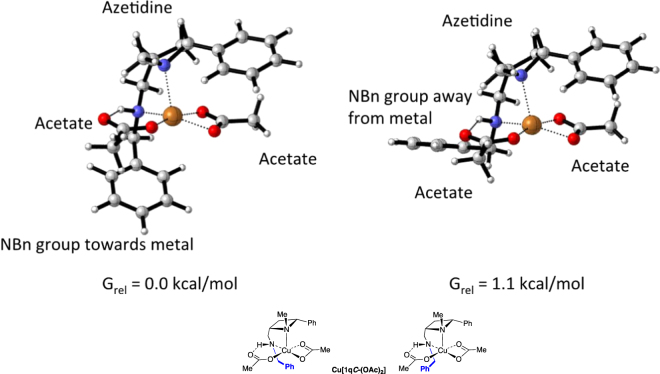
Two calculated minima and free energies in kcal/mol for the calculated copper(II)acetate-derived complex of **1q*C***.

Three possibilities were considered: (i) Alternative coordination geometries around the copper; (ii) the inclusion of the benzyl substituent (simplified to a methyl in the previous calculation) on the exocyclic nitrogen (**1p*C***); and (iii) solvation effects. Addressing each in turn: (i) Several alternative coordination geometries were studied but none were found to be lower in energy. (ii) Swapping the H*N*-methyl group of **1p*C*** to an H*N*-benzyl substituent (**1q*C***) increases the computational complexity but results in an interesting observation. First the conformations of **1q*C*** (Fig. 6) upon complexation with copper(II) acetate were explored, using the protocol described above. The lowest energy structure (Fig. 11, left) involves the phenyl of the H*N*-benzyl substituent projected towards the copper atom; whereas an alternative with the phenyl directed away from the metal centre is 1.1 kcal/mol higher in energy (Fig. 11, right). In both structures copper adopts a pseudo-square-based pyramidal coordination geometry, and in the lowest energy structure the phenyl of the benzyl occupies the region of space that a sixth ligand might otherwise occupy in an octahedral complex. The *cis*-stereochemistry of the ligand creates a structure with a concave cavity which envelops the copper. The acetate hydrogen bond and preferential displacement of the non-hydrogen bonding acetate, described previously, were assumed to operate in this larger system.

**Figure 12:**
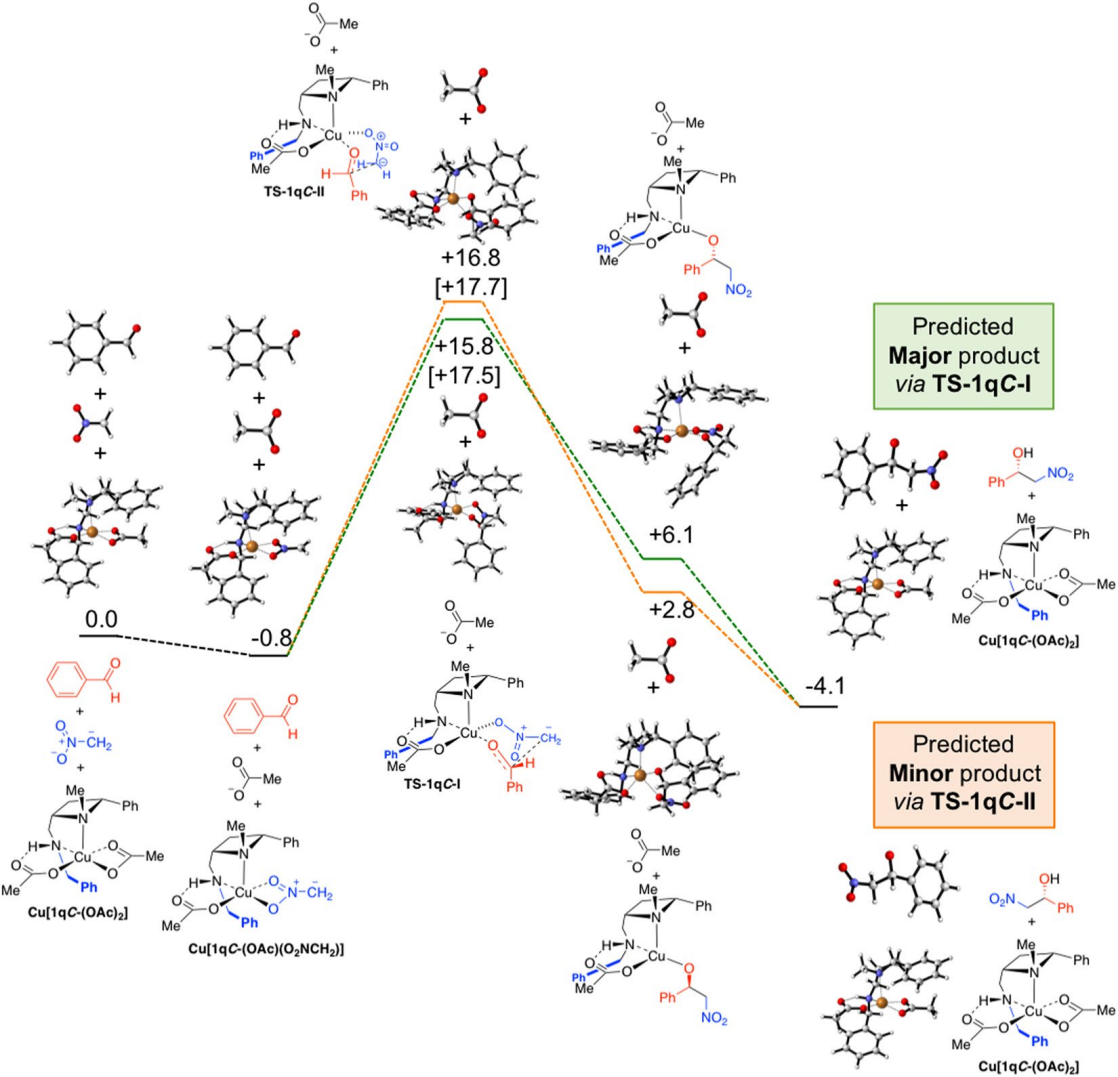
Calculated reaction coordinates and free energies in kcal/mol for the **1q*C***-catalyed reaction of benzaldehyde (**5b**) with the anion of nitromethane. Two diastereomeric transition states (**TS-1q*C*-I** and **TS-1q*C*-II**) leading to opposite enantiomers of product **6b** depicted, free energies in parenthesis include solvation in THF.

**Figure 13:**
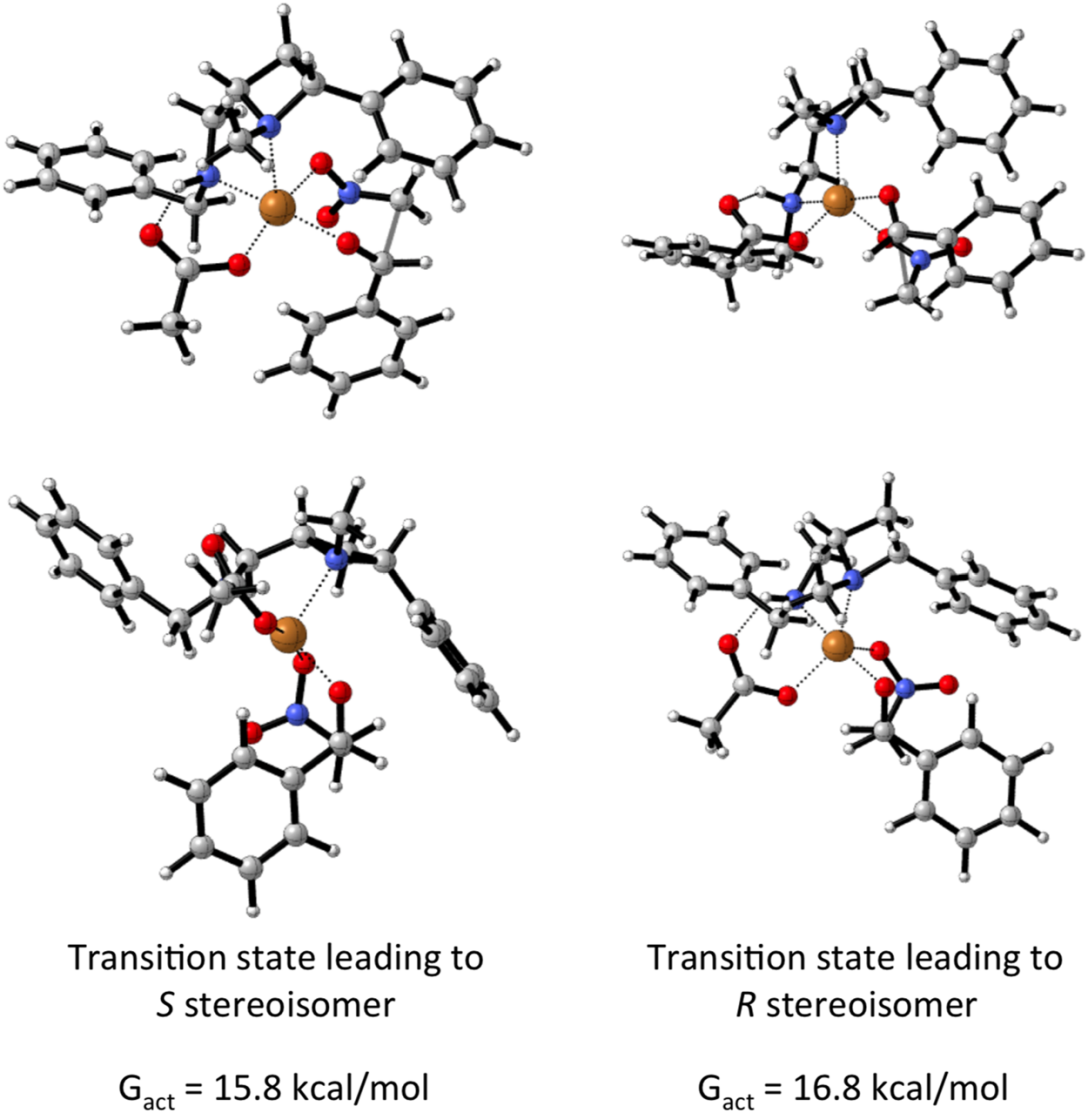
Representations of the two diastereomeric transition states **TS-1q*C*-I** (left upper and lower) and **TS-1q*C*-II** (right upper and lower), from Fig. 12, leading to the formation of enantiomers of **6b**.

Employing ligand **1q*C*** in the computed reaction of nitromethane and benzaldehyde led to the computationally predicted reaction outcome preference being in agreement with the experimentally observed (*S*)-stereoisomer of **6a** (Fig. 12). Whilst this benzyl group is oriented away from copper in the lowest energy transition states, it is exerting an important influence on the reaction outcome. The lowest energy transition states for attack on the *Si* and *Re* faces of the aldehyde involve a square pyramidal arrangement of ligands around the copper ion with the azetidine nitrogen occupying the apical position (Fig. 13). In both **TS1q*C*-I** and **TS1q*C*-II** the nitromethane anion occupies the rear right coordination site as drawn (Fig. 13 upper left and right respectively), likely because this places the anionic nitro-oxygen *trans* to the electronegative oxygen of the acetate. Were the nitromethane anion and aldehyde swapped such that, in both cases, the aldehyde lies at the rear of the complex (not drawn, see ESI tables of coordinates), the nitromethane anions are then *cis* to the acetate and result in transition states with activation barriers of 20.3 (leading to (*R*)-product) and 29.3 kcal/mol (leading to (*S*) product) respectively. Furthermore, the face of the aldehyde presented to the nitromethane anion is governed by a minimisation of a steric clash between the phenyl substituents of the aldehyde and the ligand. In the favoured TS (Fig. 13 left upper and lower) the phenyl ring of the aldehyde is orientated in an *endo* fashion (towards the metal centre), minimising steric interaction with the concave ligand architecture. Whereas, in the higher energy scenario the phenyl group of the aldehyde lies *exo* (in a direction away from the metal) but experiences more of a clash with the ligand’s phenyl substituent (right side of images as drawn in Fig. 13 right upper and lower). (iii) Increasing the polarity of the solvent (using solvation single points with the settings for THF *c.f*. Table 1, entry 13) is computed to decrease the difference in free energy between transition states in each system and therefore suggests that more polar solvents are likely to be detrimental for selectivity, which is broadly in line with experimental evidence.

The origin of the change in selectivity upon replacing N-Me (ligand **1p*C***) with N-Bn (ligand **1q*C***) in the model is a subtle change for many of the interactions involved in each of the transition states, but the tightness of binding of the amine to the metal and the impact of this on the ligand *trans* to it is likely key. In each of the key transition states, the ligand *trans* to the amine is the forming oxy anion (the aldehyde oxygen). In the transition states leading to the observed product, the N-Cu distance is 2.05 Å for both ligands and the Cu-O distance is also unchanged at 1.99 Å. By contrast, in the transition state leading to the disfavoured (*R*) product, the N-Cu distance is 2.04 Å for N-Me (ligand 1-I) and 2.03 Å for N-Bn (ligand **1q*C***); this small change causes a larger corresponding change in the Cu-O distance which increases from 1.92 Å for N-Me to 1.96 Å for N-Bn. The N-Me system is better able to stabilise the transition state leading to the (*R*) product than is N-Bn and hence the computed stereoselectivity inverts to that agreeing with experimental observations.

**Figure 14:**
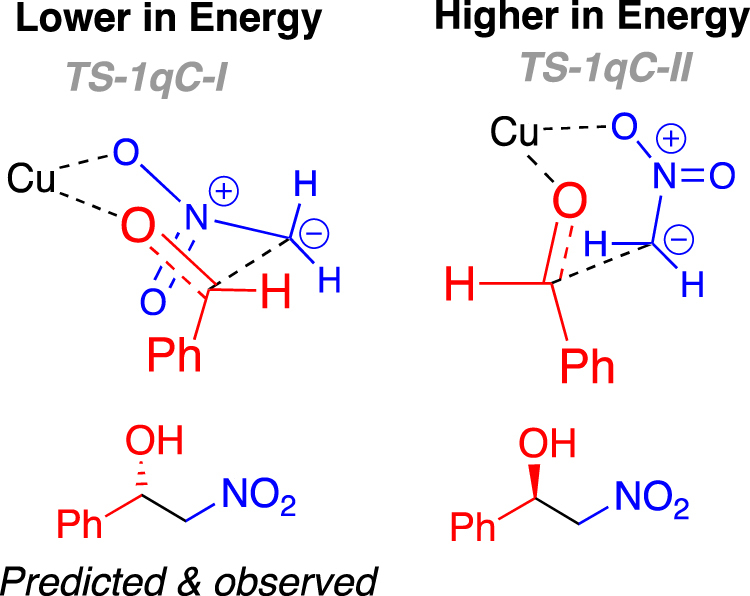
Drawings highlighting the axially substituted six-memebered chair-like transition state **TS-1q*C*-I** (left upper) and the eclipsed boat-like transition state (**TS-1q*C*-II**), and the products to which the lead (lower images respectively). The agreement between the experimentally observed and the computationally predicted products, using this model, are highlighted.

For both ligands studied computationally (**1p*C*** and **1q*C***), the geometry of the transition state leading to the *S* isomer is broadly the same. This is a six-membered chair-like structure in which the phenyl of the benzaldehyde is placed in an axial position. The space that an equivalent equatorial substituent would fill is occupied by the phenyl of the ligand. The geometry of the transition state leading to the *R* isomer is also largely unchanged when the ligand is changed. This is a boat transition state which permits the phenyl of the aldehyde to be positioned in an equatorial-like position. This is the trade-off that determines selectivity: A chair-like transition state with an axial aldehyde substituent or a boat-like transition state with an equatorial, eclipsed, aldehyde substituent. The axial position, in this case is not as strongly disfavoured as might be expected, because the transannular axial positions around the transition state are occupied by the other oxygen of the nitro group and the empty space that would otherwise be occupied by a sixth ligand on copper (making it octahedral). This axial positioning is therefore not strongly disfavoured (Fig. 14).

### Model metal complex X-ray diffraction crystal structure

**Figure 15:**
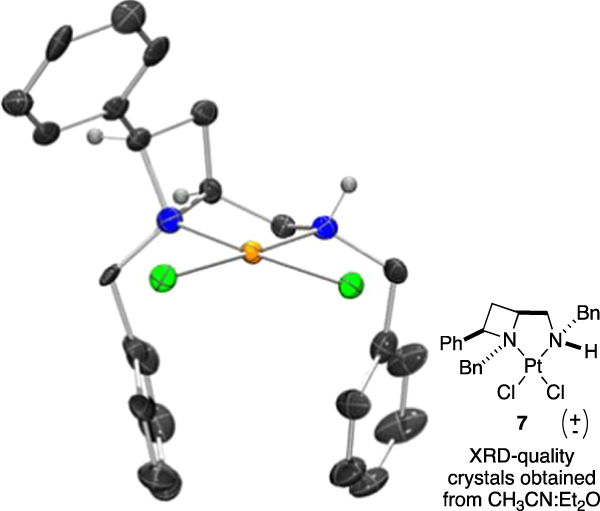
Representation of one molecule from the single crystal XRD structure of platinum complex **7**. Elipsoids plot at 50% probability, and rendered using Ortep-III for Windows and PovRay v3.7.

During the course of developing azetidine derivatives as chiral ligands for copper-catalysed Henry reactions various attempts to form, isolate and study azetidine metal complexes were made. Whilst crystals suitable for X-ray diffraction structure determination were not isolated from attempts using various copper salts, solvents and techniques, it was possible to form a small number of crystals of a square planar platinum(II) chloride complex from racemic ligand and K_2_PtCl_4_. Whilst platinum complexes are not the main topic of study in this report the crystal structure of **7** (Fig. 15) might be instructive for understanding steric interactions and corroborating selectivity hypotheses. Details of this and a small number of additional complexes of platinum and palladium will be reported in detail in a later report. Herein, the crystal structure of complex (*rac*)-**7** is used to inform discussion.

From the crystal structure of **7** it is clear the 2,4-*cis* stereochemistry of the azetidine ligand imparts a rigid geometry presenting the 2-substiuent out over the metal atom, in one quadrant of the front view of the molecule (as depicted in Fig. 15). The *N*-benzyl groups of the ring and the secondary amine part point down, as depicted, occupying two other quadrants of the molecule. This leaves the fourth, top right, quadrant open and presenting and N-H group as a potential H-bond donor. The *N*,*N*′-1,2-chelation and structural features observed in this model complex help to picture features that may be common across the ligand family and between various transition metal centres.

**Figure 16:**
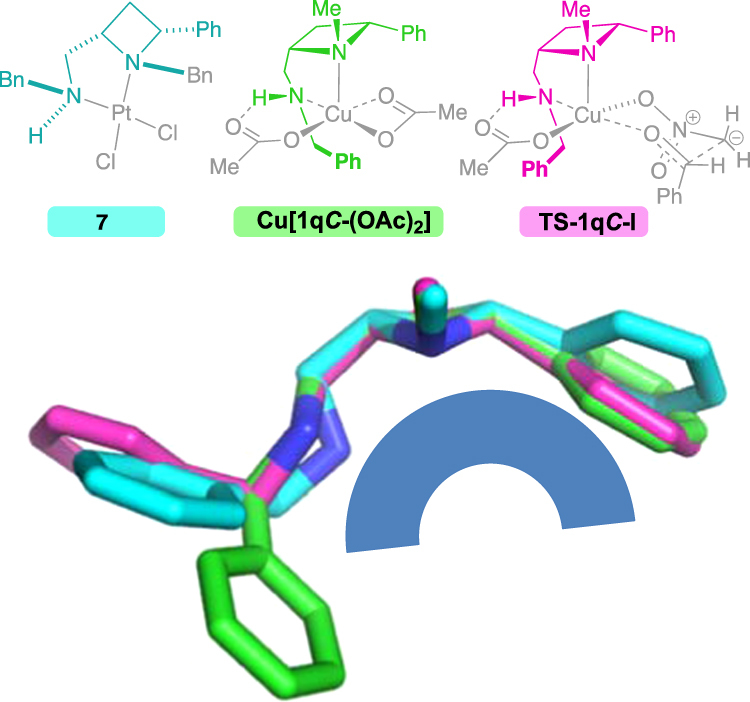
Larger central lower image, overlay of the structures (indecated and colour coded above) of: Ligth blue, XRD-derived platinum(II) complex **7**; light green, the calcualted structural minima for the coppe(II) acetate complex of **1q*C***; magenta, the calculted transtion state, leading to the experimentally observed stereochemical outcome, **TS-1q*C*-I**.

To explore the degree to which the rigid structure of the ligand is retained, even in a complex with a different metal and other ligands, the structure of complex **7** is overlaid with that found in the calculations for the lowest energy transition state (Fig. 13, **TS-1q*C*-I**) and diacetate complex (**Cu[1q*C*-(OAc)**_**2**_**]**), and shown in Fig. 16.

The common substructures components of these complexes are depicted, and colour coded: The platinum complex **7** is shown in light blue, copper diacetate complex is shown in green (**Cu[1q*C*-(OAc)**_**2**_**]**) and the copper transition state is shown in magenta **(TS-1q*C*-I**). Apart from a slight twist of the phenyl ring attached to the azetidine ring, much of the shared ligand architecture is rigid and invariant among the calculated copper complex structures and the experimentally observed platinum complex, the shared, rigid concave form of the ligand is retained across the three systems. Variation is restricted to the sidechain CH_2_N-benzyl group which adjusts to accommodate the preference of the platinum centre. That part can adapt to best complement the reactant complex or the transition state, as discussed earlier. The stable and isolable platinum complex provides a reasonable approximation for evaluating steric parameters and effects in **1**-mediated transition metal catalysis.

**Figure 17:**
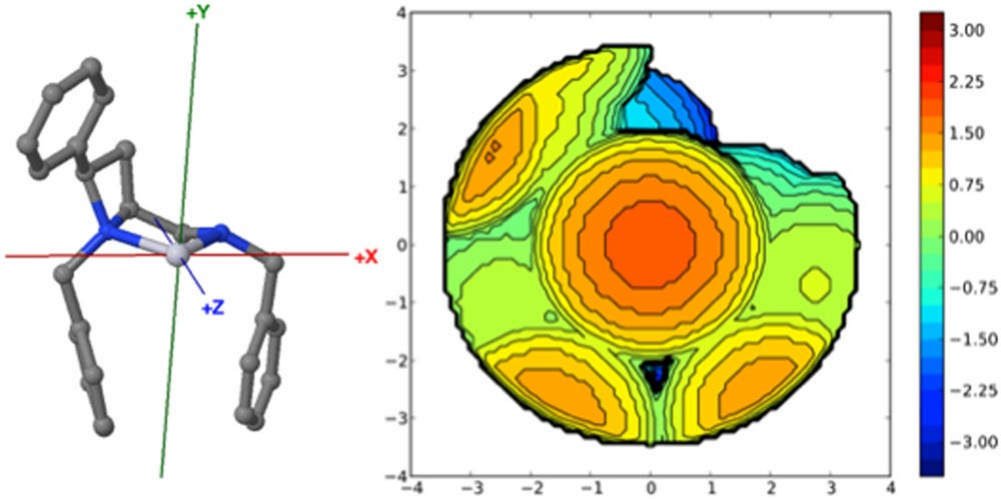
Left: Image defining axis, obtained from X-ray crystal structure of **7**, chlorides removed. Right: SambVca 2.0 generated buried volume plot depicting 62.1%V_Bur_ SW 69.6%; NW 67.2%; NE 43.3%; SE 68.4%, derived from a molecule of **11** in its X-ray crystal structure.

Inspired by publications from Nolan^57^, Cavallo^58^ and their respective co-workers a steric description of the crystal structure of platinum complex **7** was sought, and examination using the free web tool SambVca 2.0 was attempted^59^. The corresponding PDB file was uploaded to SambVca for analysis (one molecule of **7** from the unit cell of the crystal structure, with chlorides removed). An overall buried volume was determined to be 62.1%V_bur_, and as surmised from visual inspection of the crystal structure, the quadrant map generated (Fig. 17) confirms the *cis*-geometry of the ligand to imparts steric constraint or pressure in three of the four quadrants leaving the NH-containing quadrant essentially vacant.

Notably the two largest (or most buried) quadrants correspond to the azetidine ring 2-phenyl group and the NH-benzyl group, occupying the North West and South-East quadrants as drawn (67.2%V_bur_ and 68.4%V_bur_, respectively). The NH-benzyl group exerts the largest steric pressure around the coordination centre that corroborates the importance of including it in the preceding calculations of this report and helps to further rationalise the structure-selectivity relationships witnessed earlier.

## Conclusions

In conclusion, the 2,4-*cis*-disubstitution pattern about azetidines **1** has been demonstrated as a useful chiral scaffold about which to build ligands for asymmetric copper-catalysed Henry reactions. Through optimisation of ligand structure and conditions a system capable of delivering enantiopure (to the limits of the used analysis method) products from the addition of nitromethane to alkyl aldehydes. Furthermore, whilst a preliminary result, the same *cis*-substituted azetidine scaffold was capable of engendering asymmetry under an organocatalytic manifold (see supporting information). Computational analysis was employed to help rationalise the stereochemical outcome observed in the copper catalysed reactions herein. Initially, simplified ligand systems failed to correctly predict the observed stereochemical outcome, but inclusion of a critical group permitted the corroboration of computation and experimentation, feeding into new models of C-C bond formation. We plan to design new ligands drawing on the knowledge acquired here and deploy them across a wider substrate scope and in more metal-mediated reactions. The initial organocatalytic result will likely be followed up in a future project within the team.

### Data Availability

Procedures for ligand synthesis protocols, tables of data, spectrums and CIF files may be found among the additional supporting material (Citations to synthetic procedures, materials and methods contained within the supporting information that are not already cited in the main text are given here to acknowledge there importance to the information reported in the main text)^60–94^. A version of this manuscript was lodged with a pre-print server prior to peer evaluation of this manuscript^95^.

## Electronic supplementary material

Supporting Information

1d CIF

7 CIF

7 fcf
