# Supplementary material for: Rigid and concave, 2,4-cis-substituted azetidine derivatives: A platform for asymmetric catalysis
Source: Sci Rep. 2018 Apr 25;8:6541. doi: 10.1038/s41598-018-24784-3 (PMC5916886; doi:10.1038/s41598-018-24784-3)
Supplement: Supplementary file 1 — Supporting Information [file 41598_2018_24784_MOESM1_ESM.pdf]

Supporting Information

Rigid and concave, 2,4-*cis*-azetidine derivatives: A platform for asymmetric catalysis

Akina Yoshizawa,[(a)] *Antonio Feula,*[(a)] *Louise Male,*[(b)] *Andrew G. Leach,*[(c)*] *John S. Fossey*[(a)*]

(a) School of Chemistry, University of Birmingham, Edgbaston, Birmingham, West Midlands, B15 2TT, UK; (b) X-Ray Crystallography Facility, School of Chemistry, University of Birmingham, Edgbaston, Birmingham, West Midlands, B15 2TT, UK; (c) School of Pharmacy and Biomolecular Sciences, Liverpool John Moores University, Byrom Street, Liverpool L3 3AF, UK.

Table of Contents

General apparatus and equipment 1

Experimental 2

General procedures 2
Synthesis 4

Preliminary organocatalysis experiment 32

HPLC Traces 34

NMR Spectrums 44

Computational data tables 96

Single crystal X-ray diffraction data 138

CDCC Deposition Numbers 138
XRD Data tables for compound (2S, 4R)-1d 138
XRD Data tables for (rac)-7 142

References 168

General apparatus and equipment

Reagents were used as purchased from commercial suppliers without further purification. Anhydrous solvents were dispensed from a solvent purification system (SPS), monitored by Karl-Fisher titrations for water levels. Proton NMR spectra were recorded at 300 MHz on a Bruker AVIII300 NMR spectrometer or at 400 MHz on a Bruker AVIII400 NMR spectrometer. All ^{13}C NMR spectra are proton decoupled and recorded at 101 MHz on a Bruker AVIII400 NMR spectrometer or at 75.5 MHz on a Bruker AVIII300 NMR spectrometer, ^{19}F NMR spectra are proton decoupled and recorded at 282 MHz on a Bruker AVIII300 NMR spectrometer. The PENDANT technique ((+) = CH and CH_3 and (-) = C and CH_2) and two-dimensional NMR spectroscopy were used to confirm assignments in some cases. Proton NMR chemical shifts are reported in ppm relative to TMS (δ 0.00) or residual solvent signals, ^{13}C NMR shifts relative to chloroform (δ 77.36). Coupling constants *J* are given in Hertz (Hz). Melting points were carried out in triplicate and

an average of the values taken and reported as a range using Stuart™ digital melting point apparatus (SMP10). Infrared spectra were recorded on a PerkinElmer 100 FT-IR spectrometer at room temperature. Optical rotations were recorded on a polar 2001 Automatic Polarimeter, measurements of each sample were recorded three times and the average used. The majority of column chromatography was carried out using a Combiflash R*f* 200i (silica 4-12 g column), column traces were recorded at two wavelengths (254 nm and 280 nm) and ELS. However, amino azetidines **1** were typically purified by manual flash column chromatography, occasionally requiring repeat purifications in order to obtain analytically pure material. Enantiomeric excess (% *e.e.*) of products **6** were determined by HPLC (Shimadzu LC2010) using OD (Eurocel 01, 5 μm) and amylose-1 (Lux® 5 μm) column. All calculations were performed in Gaussian09. Minima and transition states were confirmed by computation of second-derivatives.

Experimental

General procedures

General procedure A: Preparation of N-tert *butyl sulfinylamides **S1***

Pyridinium *p*-toluenesulfonate (0.05 equiv.) was added to a suspension of aldehyde (3.00 equiv.), (*R*)-*tert*-butylsulfinamide (1.00 equiv.) and anhydrous magnesium sulfate (5.00 equiv.) in dichloromethane (100 mL). The mixture was stirred for 24 hours and monitored by TLC. After the reaction was judged complete by TLC, magnesium sulfate was removed by filtration and the volatiles removed *in vacuo*. The crude material thus obtained was purified by column chromatography using a *Combiflash Rf 200i.*

*General procedure B: Preparation of homoallyl sulfonamides **S2***

Allyl bromide (3.00 equiv.) was added to a suspension of activated zinc powder (3.00 equiv.) in anhydrous tetrahydrofuran (30 mL) at 0 °C and stirred for five minutes. Indium(III) triflate (1.30 equiv.) and (*E*)-*N-tert* butyl sulfinylamides **S1** (1.00 equiv.) were added and the mixture was stirred for 16 hours at room temperature. The reaction mixture was quenched by addition of saturated aqueous sodium bicarbonate solution, extracted with ethyl acetate (3 x 30 mL), washed with water (3 x 20 mL), dried over anhydrous magnesium sulfate, filtered and the volatile components were removed *in vacuo*. This material was purified by column chromatography using a *Combiflash Rf 200i.*

*General procedure C: Preparation of primary homoallyl amines **S3***

Sulfinamides (**S2**) were added to the solution of hydrochloric acid (4.00 M in dioxane) and stirred for 16 hours at room temperature. The reaction mixture was quenched with water, extracted with dichloromethane (3 x 30 mL), washed with water (3 x 20 mL) and dried over magnesium sulfate and the volatile compounds were removed *in vacuo*.

*General procedure D: Preparation of secondary homoallyl amines **S4***

To primary amines **S3** aldehydes (1.10 equiv.) were added in methanol and the reaction mixture was heated at reflux for 16 hours. The reaction mixture was allowed to cool to room temperature and excess sodium borohydride was added

to the reaction mixture which was stirred for a further three hours. The reaction mixture was quenched with distilled water and extracted with ethyl acetate (3 x 30 mL). The combined organic layers were washed with water (3 x 20 mL), dried over anhydrous magnesium sulfate and volatile components were removed *in vacuo*. This material was purified by column chromatography using a *Combiflash Rf 200i*.

*General procedure E: Cyclisation and derivatization of homoallylamines **S3** to synthesise azetidine derivatives **1***

Sodium hydrogen carbonate (5.00 equiv.) and iodine (3.00 equiv.) were added to a solution of homoallylamines **S3** (1.00 equiv.) in acetonitrile and the mixture was stirred at ≤ 20 °C for 16 hours. The reaction solution was quenched and washed with saturated aqueous sodium thiosulfate solution (2 x 10 mL), extracted with ethyl acetate (3 x 30 mL), washed with water (3 x 20 mL), dried over anhydrous magnesium sulfate, filtrated and the volatiles were removed *in vacuo*. The iodoazetidine thus formed (and confirmed by ^{1}H NMR spectroscopy) were not further manipulated and taken immediately on to the next step where they were stirred at room temperature for 48 hours in neat amine. Unreacted amines were removed *in vacuo* and the crude material was purified by flash column chromatography on silica.

General procedure F: Preparation of racemic Henry reaction adducts

In order to identify analytical HPLC for enantiomeric excess determination of asymmetric catalyst mediated Henry reaction adducts, corresponding racemates were required to determine conditions for enantiomer separation/resolution by analytical HPLC using a chiral stationary phase. Aqueous sodium hydroxide solution (10 M, 1.00 equiv.) was added very slowly to a solution of aldehyde (1.00 equiv.) and nitromethane (1.00 equiv.) in ethanol. The reaction mixture was stirred at room temperature for 10 minutes then acetic acid (1.00 equiv.) was added and the mixture stirred for a further five minutes. Diethyl ether was added to the reaction mixture and the resulting precipitate was removed by filtration. The organic extracts were washed with water, dried over anhydrous magnesium sulfate, filtrated and concentrated *in vacuo*, the material obtained was purified by column chromatography using a *Combiflash Rf 200i.*

General procedure G: Standard conditions for the asymmetric Henry deploying azetidines as ligands

Amino azetidine ligands **1a-o** (0.025 mmol) and metal salt (0.025 mmol) were stirred at room temperature for 1 hour in solvent (1.00 mL). Aldehydes **5a-j** (0.50 mmol) and excess nitromethane (5 mmol) were added and the resulting mixture was stirred at a defined temperature for a specified amount of time. After the reaction was judged completed, as determined by TLC, the reaction was quenched by addition of saturated aqueous ammonium chloride solution (1 x 5 mL), extracted with diethyl ether (3 x 10 mL), washed with water (3 x 10 mL), dried over anhydrous magnesium sulfate, filtered and the volatiles were removed *in vacuo*. Conversion was determined by ^{1}H NMR spectroscopy and the residues were purified by a silica gel chromatography using a *Combiflash Rf 200i* to afford the nitroaldol products **6a-j**. The enantiomeric excess was determined by analytical HPLC using a chiral stationary phase.

Synthesis

An Ellman auxiliary[1] route is used to deliver single enantiomer amines for cyclisation to azetidines according to a route previously described by us.[2] The first step is the synthesis of sulfinamides **S1a-e**, Scheme S 1, followed by allylation Scheme S 2 to **S2a-e**, auxiliary removal to **S3a-e** and reductive amination to **S4a-j** Scheme S 3. Subsequent iodine mediated cyclisation and direct iodine displacement by amines to give products **S5a-o** Scheme S 4

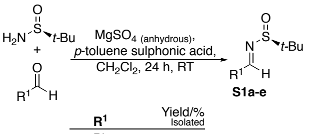

	R^1	Yield/% Isolated
**a**	Ph	62
**b**	1-Naphth	78
**c**	4-*t*-Bu-C_6H_4	76
**d**	4-NO_2-C_6H_4	61
**e**	4-Cl-C_6H_4	76

Scheme S 1*. General procedure A leading to products* ***S1a-e****.*

Synthesis of (S, E)-N-benzylidene-2-methylpropane-2-sulfinamide, ***S1a****.*

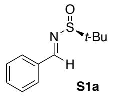

Prepared using *General Procedure A* with (*S*)-*tert*-butanesulfinamide (2.00 g, 1.00 equiv., 16.5 mmol), benzaldehyde (5.25 g, 3.00 equiv., 49.5 mmol), pyridinium *p*-toluenesulfonate (207 mg, 0.05 equiv., 0.83 mmol), magnesium sulfate (9.93 g, 5.00 equiv., 82.5 mmol) and dichloromethane (100 mL). Purified using a *Combiflash Rf 200i* (silica 12 g column, 0-20% EtOAc/*n*-hexane). The characterisation reported is consistent with that given the literature.[3] Colourless oil (2.15 g, 10.3 mmol, 62%). TLC (EtOAc/n-hexane = 10:90 v/v, visualised by UV 254 nm): Rf = 0.29; ^{1}H NMR (300 MHz, $CDCl_3$): δ 8.60 (1H, s, CH=N), 7.82 (2H, dd, *J* 7.9 & 1.5, ArH), 7.56-7.45 (3H, m, ArH), 1.27 (9H, s, *t*-Bu); ^{13}C NMR (101 MHz, PENDANT, $CDCl_3$): δ 162.8 (CH, (+), CH=N), 134.1 (C, (-), Ar), 132.4 (CH, (+), Ar), 129.4 (CH, (+), Ar), 129.0 (CH, (+), Ar), 57.8 (C, (-), *t*-Bu), 22.6 (CH_3, (+), *t*-Bu); IR (neat) νmax = 3062 (w), 3026 (w), 2981 (m), 2930 (w), 1606 (s), 1450 (s), 1364 (m), 1084 (s) cm^{-1} [lit ($CHCl_3$):[3a] 2964, 1608, 1574, 1451, 1364, 1171, 1077 cm^{-1}]; MS TOF AP+ (m/z): 210.1 $[M+H]^+$.

Synthesis of (S, E)-2-methyl-N-(naphthalen-1-ylmethylene)propane-2-sulfinamide, ***S1b****.*

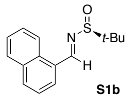

Prepared using *General Procedure A* with (*S*)-*tert*-butanesulfinamide (1.00 g, 1.00 equiv., 8.25 mmol), 1-naphthaldehyde (2.87 g, 3.00 equiv., 24.8 mmol), pyridinium *p*-toluenesulfonate (104 mg, 0.05 equiv., 0.41 mmol), magnesium sulfate (4.97 g, 5.00 equiv., 41.3 mmol) and dichloromethane (50 mL). Purified using a *Combiflash Rf 200i* (silica 12 g column, 0-20% EtOAc/*n*-hexane). The characterisation reported is consistent with that given the literature.[4] Yellow oil (1.67 g, 6.44 mmol, 78%). TLC (EtOAc/*n*-hexane = 5:95 v/v, visualised with $KMnO_4$): R_f = 0.15; ^{1}H NMR (300 MHz, $CDCl_3$,): δ 9.16 (1H, s, C*H*=N), 9.04 (1H, d, *J* 8.6, Ar*H*), 8.04 (2H, dd, *J* 7.1 & 8.2, Ar*H*), 7.93 (1H, d, *J* 8.7, Ar*H*), 7.68-7.55 (3H, m, Ar*H*), 1.33 (9H, s, *t*-Bu); ^{13}C NMR (101 MHz, PENDANT, $CDCl_3$): δ 162.5 (CH, (+), *C*H=N), 133.9 (C, (-), Ar), 133.3 (CH, (+), Ar), 132.1 (CH, (+), Ar), 131.3 (C, (-), Ar), 129.4 (C, (-), Ar), 128.9 (CH, (+), Ar), 128.1 (CH, (+), Ar), 126.5 (CH, (+), Ar), 125.3 (CH, (+), Ar), 124.4 (CH, (+), Ar), 57.7 (C, (-), *t*-Bu), 22.7 (CH_3, (+), *t*-Bu); IR (neat) ν_{max} = 3056 (w), 2960 (m), 1581 (s), 1511 (m), 1363 (m),

1175 (m), 1078 (s) cm^{-1} [lit[4] (*R* isomer, KBr): 3054, 2965, 1579, 1512, 1364, 1178, 1073 cm^{-1}]; MS TOF ES+ (*m/z*): 282.1 $[M+Na]^+$.

*Synthesis of (S, E)-N-(4-(tert-butyl)benzylidene)-2-methylpropane-2-sulfinamide, **S1c**.*

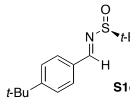

S1c

Prepared using *General Procedure A* with (S)-tert-butanesulfinamide (1.00 g, 1.00 equiv., 8.25 mmol), 4-*tert* butylbenzaldehyde (4.01 g, 3.00 equiv., 24.8 mmol), pyridinium *p*-toluenesulfonate (104 mg, 0.50 equiv., 0.41 mmol), magnesium sulfate (4.97 g, 5.00 equiv., 41.3 mmol) and dichloromethane (50.0 mL). Purified using a *Combiflash Rf 200i* (silica 12 g column, 0-20% EtOAc/*n*-hexane). White crystalline solid (1.67 g, 6.29 mmol, 76%). mp: 76-78 °C; TLC (EtOAc:*n*-hexane = 5:95 v/v, visualised with $KMnO_4$): R_f = 0.29; δ 8.57 (1H, s, C*H*=N), 7.80 (2H, d, *J* 8.4 Hz, Ar*H*), 7.51 (2H, d, *J* 8.4 Hz, Ar*H*), 1.35 (9H, s, *t*-Bu), 1.26 (9H, s, *t*-Bu); ^{13}C NMR (101 MHz, PENDANT, $CDCl_3$): δ 162.5 (CH, (+), *C*H=N), 156.2 (C, (-), Ar), 131.6 (C, (-), Ar), 129.3 (CH, (+), Ar), 126.0 (CH, (+), Ar), 57.7 (C, (-), *t*-Bu), 35.2 (C, (-), *t*-Bu), 31.1 (CH_3, (+), *t*-Bu), 22.6 (CH_3, (+), *t*-Bu); IR (solid) ν_{max} = 2961 (m), 2905 (m), 2869 (m), 1594 (s), 1561 (s), 1475 (m), 1411 (w), 1364 (s), 1084 (s) cm^{-1}; MS TOF ES+ (*m/z*): 288.2 $[M+Na]^+$.

*Synthesis of (S, E)-2-Methyl-N-(4-nitrobenzylidene)propane-2-sulfinamide, **S1d**.*

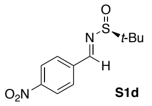

S1d

Prepared using *General Procedure A* with (*S*)-*tert*-butanesulfinamide (1.00 g, 1.00 equiv., 8.25 mmol), 4-nitrobenzaldehyde (3.74 g, 3.00 equiv., 24.8 mmol), pyridinium *p*-toluenesulfonate (104 mg, 0.05 equiv., 0.41 mmol), magnesium sulfate (4.97 g, 5.00 equiv., 41.3 mmol) and dichloromethane (50.0 mL). Purified using a *Combiflash Rf 200i* (silica 12 g column, 0-5% Et_2O/CH_2Cl_2). The characterisation reported is consistent with that given the literature.[5] Pale yellow solid (1.29 g, 5.07 mmol, 61%). mp: 139-140 °C [lit:[5] 140-142 °C]; TLC (Et_2O: CH_2Cl_2 = 2:98 v/v, visualised with $KMnO_4$): R_f = 0.46; ^{1}H NMR (300 MHz, $CDCl_3$,): δ 8.68 (1H, s, C*H*=N), 8.35 (2H, d, *J* 8.8, Ar*H*), 8.03 (2H, d, *J* 8.9, Ar*H*), 1.30 (9H, s, *t*-Bu); ^{13}C NMR (101 MHz, PENDANT, $CDCl_3$): δ 160.7 (CH, (+), *C*H=N), 149.9 (C, (-), Ar), 138.9 (C, (-), Ar), 130.1 (CH, (+), Ar), 124.2 (CH, (+), Ar), 58.5 (C, (-), *t*-Bu), 22.7 (CH_3, (+), *t*-Bu); IR (Solid) ν_{max} = 3109 (w), 2949 (w), 1587 (m), 1516 (s), 1472 (m), 1341 (s), 1175 (m), 1084 (s), 857 (m), 834 (m), 732 (m), 688 (m), 661 (m) cm^{-1}[lit[5] ($CHCl_3$): 3009, 2985, 2337, 1616, 1527, 1348, 1074 cm^{-1}]; MS TOF AP+ (*m/z*): 255.1 $[M+H]^+$.

*Synthesis of (S, E)-N-(4-chlorobenzylidene)-2-methylpropane-2-sulfinamide, **S1e**.*

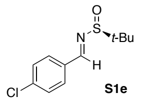

S1e

Prepared using *General Procedure A* with (*S*)-*tert*-butanesulfinamide (1.00 g, 1.00 equiv., 8.25 mmol), 4-chlorobenzaldehyde (3.48 g, 3.00 equiv., 24.8 mmol), pyridinium *p*-toluenesulfonate (104 mg, 0.0500 equiv., 0.413 mmol) magnesium sulfate (4.97 g, 5.00 equiv., 41.3 mmol) and dichloromethane (50.0 mL). Purified using a *Combiflash Rf 200i* (silica 12 g column, 0-20% EtOAc/*n*-hexane). Data reported is in accordance with that found in the literature.[6] White crystalline solid (1.53 g, 6.28 mmol, 76%). mp: 39-41 °C [lit:[6] 37-38 °C]; TLC (EtOAc:*n*-hexane = 10:90 v/v, visualised by UV 254 nm): R_f = 0.29; ^{1}H NMR (300 MHz, $CDCl_3$,): δ 8.55 (1H, s,

C*H*=N), 7.80 (2H, d, *J* 8.6, Ar*H*), 7.46 (2H, d, *J* 8.5, Ar*H*), 1.27 (9H, s, *t*-Bu); ^{13}C NMR (101 MHz, PENDANT, $CDCl_3$): δ 161.5 (CH, (+), *C*H=N), 138.7 (C, (-), Ar), 132.5 (C, (-), Ar), 130.6 (CH, (+), Ar), 129.3 (CH, (+), Ar), 58.0 (C, (-), *t*-Bu), 22.6 (CH_3, (+), *t*-Bu); IR (solid) ν_{max} = 2961 (m), 1592 (s), 1566 (s), 1489 (m), 1404 (m), 1363 (m), 1083 (s) cm^{-1}; MS TOF ES+ (*m/z*): 266.05 [M+Na]$^+$.

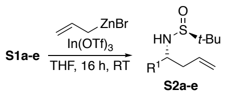

	R^1	D.R. Initial	Yield/% Isolated	D.R. Isolated
**a**	Ph	*8:1*	56	>99:1
**b**	1-Naphth	*5:1*	56	>99:1
**c**	4-*t*-Bu-C_6H_4	*6:1*	77	>99:1
**d**	4-NO_2-C_6H_4	*16:1*	83	>99:1
**e**	4-Cl-C_6H_4	*11:1*	62	>99:1

Scheme S 2*. General procedure B, the conversion of* ***S1a-e*** *into* ***S2a-e****.*

*Synthesis of (*S*)-2-methyl-*N*-((*R*)-1-phenylbut-3-en-1-yl)propane-2-sulfinamide,* ***S2a****.*

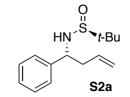

Prepared using *General Procedure B* with (*S, E*)-*N*-benzylidene-2-methylpropane-2-sulfinamide **S1a** (600 mg, 1.00 equiv., 2.87 mmol), allyl bromide (0.50 mL, 2.0 equiv., 5.73 mmol), zinc powder (469 mg, 2.50 equiv., 7.17 mmol), indium(III) trifluoromethanesulfonate (2.09 g, 1.30 equiv., 3.73 mmol), anhydrous tetrahydrofuran (30 mL). Purified using a *Combiflash Rf 200i* (silica 12 g column, 0-40% EtOAc/petroleum ether). Data reported is in accord with that found in the literature.[7] White solid (407 mg, 1.62 mmol, 56%). mp: 68-70 ºC; dr = >99:1 (crude dr = 8:1); TLC (EtOAc:petroleum ether = 30:70 v/v, visualised with $KMnO_4$): R_f = 0.28; ^{1}H NMR (300 MHz, $CDCl_3$,): δ 7.35-7.25 (5H, m, Ar*H*), 5.80-5.67 (1H, m, CH_2C*H*CH_2), 5.22-5.15 (2H, m, CH_2CHC*H*$_2$), 4.50-4.44 (1H, m, PhC*H*NH), 3.68 (1H, br s, N*H*), 2.65-2.56 (1H, m, CHC*H*HCH), 2.52-2.42 (1H, m, CHCH*H*CH), 1.19 (9H, s, *t*-Bu); ^{13}C NMR (101 MHz, PENDANT, $CDCl_3$): δ 141.7 (C, (-), Ar), 134.2 (CH, (+), CH_2*C*HCH_2), 128.5 (CH, (+), Ar), 127.7 (CH, (+), Ar), 127.5 (CH, (+), Ar), 119.3 (CH_2, (-),CH_2CH*C*H$_2$), 57.0 (CH, (+), *C*HCH_2CH), 55.6 (C, (-), *t*-Bu), 43.5 (CH_2, (-), CH*C*H$_2$CH), 22.6 (CH_3, (+), *t*-Bu); IR (Solid) ν_{max} = 3337 (w), 3209 (m), 3085 (w), 3058 (w), 3030 (w), 2957 (m), 2907 (m), 2908 (m), 2869 (m), 1644 (m), 1451 (s), 1348 (m), 1292 (s), 1139 (m), 1115 (m), 1050 (s), 906 (m), 958 (m), 917 (s), 777 (s), 755 (s), 696 (s) cm^{-1} [lit:[7] (film) 3033, 2927, 2869, 2235, 1640, 1456, 1058, 911, 734, 701 cm^{-1}]; MS TOF ES+ (*m/z*): 274.1 [M+Na]$^+$.

*Synthesis of (*S*)-2-methyl-*N*-((*R*)-1-(naphthalen-1-yl)but-3-en-1-yl)propane-2-sulfinamide,* ***S2b****.*

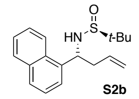

Prepared using *General Procedure B* with (*S, E*)-2-methyl-*N*-(naphthalen-1-ylmethylene)propane-2-sulfinamide **S1b** (600 mg, 1.00 equiv., 2.31 mmol), allyl bromide (0.40 mL, 4.6 mmol), zinc powder (378 mg, 2.50 equiv., 5.78 mmol), indium(III) trifluoromethanesulfonate (1.69 g, 1.30 equiv., 3.01 mmol), anhydrous tetrahydrofuran (30.0 mL). Purified using a *Combiflash Rf 200i* (silica 12 g column, 0-40% EtOAc/petroleum ether). ^{1}H and ^{13}C NMR spectroscopy, optical rotation, IR and mass spectra reported are consistent with that found in the literature.[8] Pale yellow sticky oil (389 mg, 1.29 mmol, 56%). dr = >99:1 (crude compound dr = 5:1); TLC (EtOAc:pet ether = 30:70 v/v, visualised by UV 254 nm): R_f = 0.33; ^{1}H NMR (400 MHz, $CDCl_3$,): δ 8.19 (1H, d, *J* 8.4, Ar*H*),

7.88 (1H, d, *J* 9.4, Ar*H*), 7.79 (1H, d, *J* 6.1, Ar*H*), 7.59-7.44 (4H, m, Ar*H*), 5.84-5.74 (1H, m, CH_2C*H*CH_2), 5.33-5.29 (1H, m, CH_2CHC*H_2*), 5.26-5.18 (2H, m, C*H*NH), 3.82 (1H, s, N*H*), 2.88-2.80 (1H, m, CHC*H_2*CH), 2.71-2.63 (1H, m, CHC*H_2*CH), 1.22 (9H, s, *t*-Bu); ^{13}C NMR (101 MHz, PENDANT, $CDCl_3$): 137.2 (C, (-), Ar), 134.3 (CH, (+), CH_2*C*HCH_2), 134.0 (C, (-), Ar), 131.0 (C, (-), Ar), 129.0 (CH, (+), Ar), 128.2 (CH, (+), Ar), 126.2 (CH, (+), Ar), 125.6 (CH, (+), Ar), 125.2 (CH, (+), Ar), 125.0 (CH, (+), Ar), 123.2 (CH, (+), Ar), 119.4 (CH_2, (-),CH_2CH*C*H_2), 55.8 (C, (-), *t*-Bu), 53.5 (CH, (+), *C*HCH_2CH), 42.3 (CH_2, (-), CH*C*H_2CH), 22.7 (CH_3, (+), *t*-Bu); IR (neat) ν_{max} = 3207 (m), 2958 (m), 2929 (m), 2868 (w), 1639 (w), 1600 (w), 1518 (w), 1364 (m), 1169 (m), 1059 (s), 917 (m), 778 (s) cm^{-1} [lit (film):[8] 3281, 3209, 2957, 2923, 2866, 1639, 1597, 1511, 1364, 1169, 1063, 920, 779, 751 cm^{-1}]; MS TOF ES+ (*m/z*): 324.1 $[M+Na]^+$.

*Synthesis of (*S*)-*N*-((*R*)-1-(4-(*tert*-butyl)phenyl)but-3-en-1-yl)-2-methylpropane-2-sulfinamide,* ***S2c***.

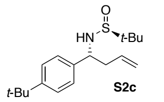

Prepared using *General Procedure B* with (*R, E*)-*N*-(4-(*tert*-butyl)benzylidene)-2-methylpropane-2-sulfinamide **S1c** (600 mg, 1.00 equiv., 2.31 mmol), allyl bromide (0.391 mL, 2.00 equiv., 4.52 mmol), zinc powder (370 mg, 2.50 equiv., 5.65 mmol), indium(III) trifluoromethanesulfonate (1.65 g, 1.30 equiv., 2.94 mmol), anhydrous tetrahydrofuran (30 mL). Purified by flash column chromatography (silica gel 40-63 μm, 30 % EtOAc/petroleum ether). Colourless oil (533 mg, 1.73 mmol, 77%), dr = >99:1 (crude compound dr = 6:1); TLC (EtOAc:petroleum ether = 30:70 v/v, visualised with $KMnO_4$): R_f = 0.31; ^1H NMR (400 MHz, $CDCl_3$,): δ7.35-7.22 (4H, m, Ar*H*), 5.75-5.71 (1H, m, CH_2C*H*CH_2), 5.22-5.16 (2H, m, CH_2CHC*H_2*), 4.47-4.43 (1H, m, PhC*H*NH), 3.67 (1H, s, N*H*), 2.62-2.45 (2H, m, CHC*H_2*CH), 1.32 (9H, s, *t*-Bu), 1.20 (9H, s, *t*-Bu); ^{13}C NMR (101 MHz, PENDANT, $CDCl_3$): δ150.4 (C, (-), Ar), 138.6 (C, (-), Ar), 134.4 (CH, (+), CH_2*C*HCH_2), 127.0 (CH, (+), Ar), 125.3 (CH, (+), Ar), 119.2 (CH_2, (-),CH_2CH*C*H_2), 56.7 (CH, (+), *C*HCH_2CH), 55.7 (C, (-), *t*-Bu), 43.5 (CH_2, (-), CH*C*H_2CH), 34.5 (C, (-), *t*-Bu), 31.4 (CH_3, (+), *t*-Bu), 22.7 (CH_3, (+), *t*-Bu); IR (neat) ν_{max} = 3440 (w), 3207 (w), 3078 (w), 2959 (s), 2906 (m), 2868 (m), 1640 (w), 1510 (m), 1474 (m), 1363 (m), 1269 (m), 1110 (m), 1053 (s), 995 (m), 913 (m), 831 (s) cm^{-1}; MS TOF ES+ (*m/z*): 330.2 $[M+Na]^+$; HRMS (m/z): $[M+Na]^+$ calcd. for $C_{18}H_{29}NONaS^+$, 330.1862; found, 330.1864.

*Synthesis of (*R*)-2-methyl-*N*-((*S*)-1-(4-nitrophenyl)but-3-en-1-yl)propane-2-sulfinamide,* ***S2d***.

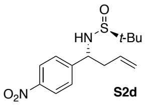

Prepared using *General Procedure B* with (*R*, *E*)-2-methyl-*N*-(4-nitrobenzylidene) propane-2-sulfinamide **S1d** (1.00 g, 1.00 equiv., 3.93 mmol), allyl bromide (0.681 mL, 2.00 equiv., 7.86 mmol), zinc powder (643 mg, 2.50 equiv., 9.83 mmol), indium(III) trifluoromethanesulfonate (2.87 g, 1.30 equiv., 5.11 mmol), anhydrous tetrahydrofuran (30 mL). Purified using a *Combiflash Rf 200i* (silica 12 g column, 0-70% EtOAc/petroleum ether). Pale yellow crystalline solid (968 mg, 3.27 mmol, 83%). mp: 112-113 °C; dr = >99:1 (crude product dr = 16:1); TLC (EtOAc:petroleum ether = 50:50 v/v, visualised with $KMnO_4$): R_f = 0.36; ^1H NMR (300 MHz, $CDCl_3$,): δ 8.22 (2H, d, *J* 8.8 Hz, Ar*H*), 7.51 (2H, d, *J* 8.7 Hz, Ar*H*), 5.72-5.64 (1H, m, CH_2C*H*CH_2), 5.23-5.16 (2H, m, CH_2CHC*H_2*), 4.63-4.58 (1H, m, PhC*H*NH), 3.76 (1H, s, N*H*), 2.63-2.46 (2H, m, CHC*H_2*CH), 1.22 (9H, s, *t*Bu); ^{13}C NMR (101

MHz, PENDANT, $CDCl_3$): δ 149.3 (C, (-), Ar), 147.5 (C, (-), Ar), 133.0 (CH, (+), CH_2*C*HCH_2), 128.4 (CH, (+), Ar), 123.8 (CH, (+), Ar), 120.3 (CH_2, (-),CH_2CH*C*H_2), 56.4 (CH, (+), *C*HCH_2CH), 56.0 (C, (-), *t*Bu), 43.1 (CH_2, (-), CH*C*H_2CH), 22.5 (CH_3, (+), *t*Bu); IR (solid) ν_{max} = 3206 (w), 3079 (w), 2962 (w), 2933 (w), 2867 (w), 1641 (w), 1601 (m), 1519 (s), 1474 (w), 1344 (s), 1052 (s), 1014 (m), 919 (m), 854 (s) cm^{-1}; MS TOF ES+ (*m/z*): 319.11 $[M+Na]^+$; HRMS (m/z): $[M+Na]^+$ calcd. for $C_{14}H_{20}N_2O_3NaS^+$, 319.1087; found, 319.1093

Synthesis of (R)-2-methyl-N-((S)-1-(4-nitrophenyl)but-3-en-1-yl)propane-2-sulfinamide, ***S2e***.

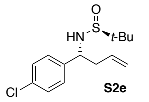

Prepared using *General Procedure B* with (*R*, *E*)-2-methyl-*N*-(4-nitrobenzylidene) propane-2-sulfinamide **S1e** (1.00 g, 1.00 equiv., 3.93 mmol), allyl bromide (0.68 mL, 2.00 equiv., 7.86 mmol), zinc powder (643 mg, 2.50 equiv., 9.83 mmol), indium(III) trifluoromethanesulfonate (2.87 g, 1.30 equiv., 5.11 mmol), anhydrous tetrahydrofuran (30 mL). *Purified using* a Combiflash R*f*200i (silica 12 g column, 0-70% EtOAc/petroleum ether. Pale yellow crystalline solid (968 mg, 3.27 mmol, 83%). Mp: 112-113 °C; dr = >99:1 (crude product dr = 16:1); TLC (EtOAc:petroleum ether = 50:50 v/v, visualised with $KMnO_4$): R_f = 0.36; ^1H NMR (300 MHz, $CDCl_3$,): δ 8.22 (2H, d, *J* 8.8, Ar*H*), 7.51 (2H, d, *J* 8.7, Ar*H*), 5.72-5.64 (1H, m, CH_2C*H*CH_2), 5.23-5.16 (2H, m, CH_2CHC*H*$_2$), 4.63-4.58 (1H, m, PhC*H*NH), 3.76 (1H, s, N*H*), 2.63-2.46 (2H, m, CHC*H*$_2$CH), 1.22 (9H, s, *t*-Bu); ^{13}C NMR (101 MHz, PENDANT, $CDCl_3$): δ 149.3 (C, (-), Ar), 147.5 (C, (-), Ar), 133.0 (CH, (+), CH_2*C*HCH_2), 128.4 (CH, (+), Ar), 123.8 (CH, (+), Ar), 120.3 (CH_2, (-),CH_2CH*C*H_2), 56.4 (CH, (+), *C*HCH_2CH), 56.0 (C, (-), *t*-Bu), 43.1 (CH_2, (-), CH*C*H_2CH), 22.5 (CH_3, (+), *t*-Bu); IR (solid) ν_{max} = 3206 (w), 3079 (w), 2962 (w), 2933 (w), 2867 (w), 1641 (w), 1601 (m), 1519 (s), 1474 (w), 1344 (s), 1052 (s), 1014 (m), 919 (m), 854 (s) cm^{-1}; MS TOF ES+ (*m/z*): 319.11 $[M+Na]^+$; HRMS (m/z): $[M+Na]^+$ calcd. for $C_{14}H_{20}N_2O_3NaS^+$, 319.1087; found, 319.1093

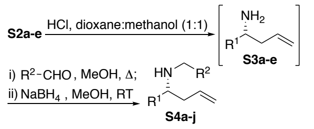

	R¹	R²	Yield/% Isolated over three steps
a	Ph	Ph	55
b	1-Naphth	Ph	75
c	4-*t*-Bu-C_6H_4	Ph	70
d	4-NO_2-C_6H_4	Ph	84
e	4-Cl-C_6H_4	Ph	65
f	Ph	4-Cl-C_6H_4	52
g	Ph	*t*-Bu	43
h	Ph	3,5-$(CF_3)_2$-C_6H_3	35
i	Ph	4-*t*-Bu-C_6H_4	55
j	Ph	4-MeO-C_6H_4	30

Scheme S 3*. General procedure C, the conversion of* ***S2a-e*** *into secondary homoallyl amines* ***S4a-j****, via amines* ***S3a-e****.*

Synthesis of (R)-1-phenylbut-3-en-1-amine ***S3a***.

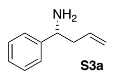

Prepared using *General Procedure C* with (*S*)-2-methyl-*N*-((*R*)-1-phenylbut-3-en-1-yl)propane-2-sulfinamide **S2a** (407 mg, 1.62 mmol) and hydrochloric acid (4 M in dioxane, 4 mL). The characterisation reported is consistent with that given in the literature.[7, 9] Colourless liquid (151 mg, 1.03 mmol, 63%). ^1H NMR (300 MHz, $CDCl_3$,): δ 7.34-7.23 (5H, m, Ar*H*), 5.77-5.69 (1H, m, CH_2C*H*CH_2), 5.16-5.06 (2H, m, CH_2CHC*H*$_2$), 4.00 (1H, dd, *J* 8.1, 5.3, C*H*N), 2.46-2.35 (2H, m, CHC*H*$_2$CH), 1.55 (2H, br s, N*H*$_2$); ^{13}C NMR (101 MHz, PENDANT,

$CDCl_3$): δ 145.9 (C, (-), Ar), 135.5 (CH, (+), CH_2*C*HCH_2), 128.4 (CH, (+), Ar), 127.0 (CH, (+), Ar), 126.3 (CH, (+), Ar), 117.7 (CH_2, (-), CH_2CH*C*H_2), 55.4 (CH, (+), Ph*C*HNH_2), 44.2 (CH_2, (-), CH*C*H_2CH); IR (neat) ν_{max} = 3371 (w, NH), 3070 (w), 3028 (w), 2977 (w), 2908 (w), 2850 (w), 1639 (m), 1603 (m), 1493 (m), 1453 (m), 1336 (w), 1069 (w), 1028 (w), 997 (m), 913 (s), 839 (m), 757 (s), 698 (s) cm^{-1} [lit (film):[9] 3379 (NH), 3065, 3026, 1639, 1492 cm^{-1}]; MS TOF ES+ (*m/z*): 131.1 $[M-NH_2]^+$.

Synthesis of (R)-1-(naphthalen-1-yl)but-3-en-1-amine ***S3b***.

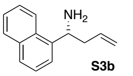

Prepared using *General Procedure C* with (*R*)-2-methyl-*N*-((*S*)-1-(naphthalen-1-yl)but-3-en-1-yl)propane-2-sulfinamide **S2b** (389 mg, 1.26 mmol) and hydrochloric acid (4 M in dioxane, 3.9 mL). Pale yellow oil (212 mg, 1.07 mmol, 85%). ^{1}H NMR (400 MHz, $CDCl_3$,): δ 8.12 (1H, dd, *J* 8.6 & 1.3, Ar*H*), 7.86 (1H, dd, *J* 7.9 & 1.6, Ar*H*), 7.74 (1H, dt, *J* 8.2 & 1.1, Ar*H*), 7.64 (1H, dt, *J* 7.2 & 0.9, Ar*H*), 7.53-7.44 (3H, m, Ar*H*), 5.85 (1H, dddd, *J* 16.8, 10.1, 7.8 & 6.3, CH_2C*H*CH_2), 5.21-5.11 (2H, m, CH_2CHC*H_2*), 4.84 (1H, dd, *J* 8.5 & 4.2, C*H*N), 2.68-2.42 (2H, m, CHC*H_2*CH), 1.63 (2H, br s, N*H_2*); ^{13}C NMR (101 MHz, PENDANT, $CDCl_3$): δ 141.5 (C, (-), Ar), 135.7 (CH, (+), CH_2*C*HCH_2), 133.9 (C, (-), Ar), 130.8 (C, (-), Ar), 129.1 (CH, (+), Ar), 127.4 (CH, (+), Ar), 126.0 (CH, (+), Ar), 125.6 (CH, (+), Ar), 125.5 (CH, (+), Ar), 122.9 (CH, (+), Ar), 122.5 (CH, (+), Ar), 117.8 (CH_2, (-), CH_2CH*C*H_2), 50.3 (CH, (+), Ph*C*HNH_2), 43.2 (CH_2, (-), CH*C*H_2CH); IR (neat) ν_{max} = 3360 (w, NH), 3059 (w), 2975 (w), 2921 (w), 2860 (w), 1638 (m, HC=C), 1597 (m), 1509 (m), 1436 (w), 1395 (w), 1258 (w), 1166 (w), 1065 (w), 996 (m), 914 (s), 860 (m), 798 (s), 775 (s) cm^{-1}; MS TOF AP+ (*m/z*): 198.1 $[M+H]^+$; HRMS (m/z): $[M+H]^+$ calcd. for $C_{14}H_{16}N^+$, 198.1277; found, 198.1282.

Synthesis of (R)-1-(4-(tert-butyl)phenyl)but-3-en-1-amine ***S3c***.

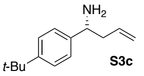

Prepared using *General Procedure C* with (*R*)-*N*-((*S*)-1-(4-(*tert*-butyl)phenyl)but-3-en-1-yl)-2-methylpropane-2-sulfinamide **S2c** (533 mg, 1.73 mmol) and hydrochloric acid (4 M in dioxane, 5.3 mL). Colourless oil (296 mg, 1.46 mmol, 84%). ^{1}H NMR (400 MHz, $CDCl_3$,): δ 7.35 (2H, d, *J* 8.4, Ar*H*), 7.27 (2H, d, *J* 8.3, Ar*H*), 5.76 (1H, dddd, *J* 17.1, 10.2, 8.0 & 6.2, CH_2C*H*CH_2), 5.21-4.98 (2H, m, CH_2CHC*H_2*), 3.97 (1H, dd, *J* 8.2 & 5.2, C*H*N), 2.50-2.35 (2H, m, C*H_2*CHCH_2), 1.98 (2H, br s, N*H_2*)1.32 (9H, s, *t*-Bu); ^{13}C NMR (101 MHz, PENDANT, $CDCl_3$): δ 149.9 (C, (-), Ar), 142.5 (C, (-), Ar), 135.5 (CH, (+), CH_2*C*HCH_2), 126.0 (CH, (+), Ar), 125.3 (CH, (+), Ar), 117.6 (CH_2, (-),CH_2CH*C*H_2), 55.0 (CH_2, (-), *C*HN), 43.9 (CH_2, (-), CH*C*H_2CH), 34.5 (C, (-), *t*-Bu), 31.4 (CH_3, (+), *t*-Bu); IR (neat) ν_{max} = 3304 (w, NH), 3077 (w), 2961 (s), 2904 (m), 2868 (w), 1640 (m, HC=C), 1582 (m), 1510 (m), 1462 (s), 1435 (w), 1410 (w), 1362 (m), 1269 (m), 1203 (w), 1110 (m), 997 (m), 913 (s), 829 (s) cm^{-1}; MS TOF ES+ (*m/z*): 204.19 $[M+H]^+$; HRMS (m/z): $[M+H]^+$ calcd. for $C_{14}H_{22}N^+$, 204.1747; found, 204.1754.

Synthesis of (R)-1-(4-nitrophenyl)but-3-en-1-amine ***S3d***.

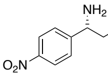
S3d

Prepared using *General Procedure C* with (*R*)-2-methyl-*N*-((*S*)-1-(4-nitrophenyl)but-3-en-1-yl)propane-2-sulfinamide **S2d** (650 mg, 2.19 mmol) and hydrochloric acid (4 M in dioxane, 5 mL). The characterisation reported is consistent with that in the literature.[10] Pale yellow oil (386 mg, 2.01 mmol, 92% yield).; ^{1}H NMR (400 MHz, $CDCl_3$,): δ 8.19 (2H, d, *J* 8.8, Ar*H*), 7.53 (2H, d, *J* 8.8, Ar*H*), 5.88-5.55 (1H, m, $CH_2C\mathit{H}CH_2$), 5.22-5.05 (2H, m, $CH_2CHC\mathit{H_2}$), 4.15 (1H, dd, *J* 8.0, 5.2 Hz, C*H*N), 2.57-2.29 (2H, m, $CHC\mathit{H_2}CH$), 1.60 (2H, br s, $N\mathit{H_2}$); ^{13}C NMR (101 MHz, PENDANT, $CDCl_3$): δ 153.3 (C, (-), Ar), 147.0 (C, (-), Ar), 134.3 (CH, (+), $CH_2\mathit{C}HCH_2$), 127.3 (CH, (+), Ar), 123.7 (CH, (+), Ar), 118.7 (CH_2, (-), $CH_2CH\mathit{C}H_2$), 54.9 (CH, (+), Ph*C*HNH_2), 44.1 (CH_2, (-), $CH\mathit{C}H_2CH$); IR (neat) ν_{max} = 3376 (w, NH), 3077 (w), 2980 (w), 2926 (w), 2854 (w), 1640 (m, HC=C), 1600 (m), 1513 (s), 1437 (w), 1342 (s), 1180 (w), 1108 (m), 998 (m), 917 (s), 852 (s), 752 (s), 699 (s) cm^{-1}; MS TOF AP- (*m/z*): 191.07 [M-H]$^{-}$.

Synthesis of (R)-1-(4-chlorophenyl)but-3-en-1-amine ***S3e***.

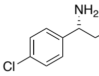
S3e

Prepared using *General Procedure C* with (*R*)-*N*-((*S*)-1-(4-chlorophenyl)but-3-en-1-yl)-2-methylpropane-2-sulfinamide **S2e** (436 mg, 1.53 mmol) and hydrochloric acid (4 M in dioxane, 4.4 mL). The characterisation reported is consistent with that given the literature.[9] Colourless oil (189 mg, 1.04 mmol, 68%). ^{1}H NMR (300 MHz, $CDCl_3$,): δ 7.32-7.27 (4H, m, Ar*H*), 5.76-5.65 (1H, m, $CH_2C\mathit{H}CH_2$), 5.15-5.07 (2H, m, $CH_2CHC\mathit{H_2}$), 3.99 (1H, dd, *J* 7.9 & 5.4, C*H*N), 2.45-2.30 (2H, m, $CHC\mathit{H_2}CH$), 1.53 (2H, br s, $N\mathit{H_2}$); ^{13}C NMR (101 MHz, PENDANT, $CDCl_3$): δ 144.3 (C, (-), Ar), 135.0 (CH, (+), $CH_2\mathit{C}HCH_2$), 132.5 (C, (-), Ar), 128.5 (CH, (+), Ar), 127.8 (CH, (+), Ar), 118.0 (CH_2, (-), $CH_2CH\mathit{C}H_2$), 54.8 (CH, (+), Ph*C*HNH_2), 44.2 (CH_2, (-), $CH\mathit{C}H_2CH$); IR (neat) ν_{max} = 3375 (w, NH), 3077 (w), 2978 (w), 2907 (w), 2844 (w), 1640 (m, HC=C), 1593 (m), 1491 (s), 1090 (s) 1013 (s), 915 (s), 822 (s) cm^{-1} [lit (film):[9] 3375 (NH), 3076, 1640 (HC=C), 1091 cm^{-1}]; MS TOF AP+ (m/z): 165.0 [M-NH_2]$^{+}$.

*Synthesis of (*R*)-*N*-benzyl-1-phenylbut-3-en-1-amine* ***S4a***.

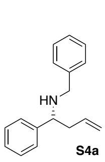
S4a

Prepared using *General Procedure **D*** with (*S*)-1-phenylbut-3-en-1-amine **S3a** (151 mg, 1.00 equiv., 1.03 mmol), benzaldehyde (109 mg, 1.00 equiv., 1.03 mmol), sodium borohydride (77.6 mg, 2.00 equiv., 2.05 mmol) and methanol (20 mL). Purified using a *Combiflash Rf 200i* (silica 4 g column, 0-30% EtOAc/*n*-hexane). The characterisation (^{1}H, ^{13}C NMR spectroscopy, and optical rotation value) reported is consistent with that given the literature.[11] Colourless oil (216 mg, 0.910 mmol, 88% yield over two steps). TLC (EtOAc:*n*-hexane= 10:90 v/v, visualised with $KMnO_4$): R_f = 0.30; ^{1}H NMR (300 MHz, $CDCl_3$,): δ 7.38-7.23 (10H, m, Ar*H*), 5.78-5.63 (1H, m, $CH_2C\mathit{H}CH_2$), 5.11-5.01 (2H, m, $CH_2CHC\mathit{H_2}$), 3.71-3.67 (1H, m, $C\mathit{H}CH_2CH$), 3.67 (1H, d, *J* =13.3, PhCH*H*N), 3.52 (1H, d, *J* 13.3 Hz, PhCH*H*N), 2.46-2.35 (2H, m, $CHC\mathit{H_2}CH$), 1.75 (br s, 1H, N*H*); ^{13}C NMR (101 MHz, PENDANT, $CDCl_3$): δ 143.9 (C, (-), Ar), 140.7 (C, (-), Ar), 135.5 (CH, (+), $CH_2\mathit{C}HCH_2$), 128.4 (CH, (+), Ar), 128.3 (CH, (+), Ar), 128.1 (CH, (+), Ar), 127.3 (CH, (+), Ar), 127.1 (CH, (+), Ar), 126.8 (CH, (+), Ar), 117.6 (CH_2,

(-),CH_2CHCH_2), 61.6 (CH, (+), Ph*C*HNH), 51.5 (CH_2, (-), NH*C*H_2Ph), 43.2 (CH_2, (-), CH*C*H_2CH); IR (neat) ν_{max} = 3328 (w), 3063 (w), 2978 (w), 2908 (w), 2834 (w), 1639 (w), 1603 (w), 1493 (m), 1453 (s), 1356 (w), 1307 (w), 1198 (w), 1115 (m), 1071 (m), 1028 (m), 995 (m), 914 (s), 824 (w), 757 (s), 732 (s), 696 (s) cm^{-1}; MS TOF ES+ (*m/z*): 238.2 $[M+H]^+$.

*Synthesis of (*R*)-*N*-benzyl-1-(naphthalen-1-yl)but-3-en-1-amine **S4b**.*

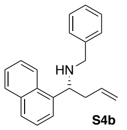
S4b

Prepared using *General Procedure **D*** with (*R*)-1-(naphthalen-1-yl)but-3-en-1-amine **S3b** (212 mg, 1.00 equiv., 1.07 mmol), benzaldehyde (114 mg, 1.00 equiv., 1.07 mmol), sodium borohydride (81.3 mg, 2.00 equiv., 2.15 mmol) and methanol (20 mL). Purified using a *Combiflash Rf 200i* (silica 4 g column, 0-30% EtOAc/*n*-hexane). The characterisation reported is consistent with that given the literature.[12] Pale yellow oil (272 mg, 0.946 mmol, 88% yield over two steps). TLC (EtOAc:*n*-hexane= 10:90 v/v, visualised with $KMnO_4$): R_f = 0.32; ^{1}H NMR (400 MHz, $CDCl_3$,): δ8.22-8.15 (1H, m, Ar*H*), 7.93-7.84 (1H, m, Ar*H*), 7.84-7.74 (2H, m, Ar*H*), 7.55-7.43 (3H, m, Ar*H*), 7.37-7.19 (5H, m, Ar*H*), 5.86-5.80 (1H, m, CH_2C*H*CH_2), 5.17-5.02 (2H, m, CH_2CHC*H_2*), 4.59 (1H, dd, *J* 8.4 & 4.6, C*H*CH_2CH), 3.75 (1H, d, *J* 13.2, NHC*H*H), 3.58 (1H, d, *J* 13.2, NHCH*H*), 2.69-2.43 (2H, m, CHC*H_2*CH), 1.77 (1H, br s, N*H*); ^{13}C NMR (101 MHz, PENDANT, $CDCl_3$): δ140.7 (C, (-), Ar), 139.1 (C, (-), Ar), 135.6 (CH, (+), CH_2*C*HCH_2), 134.1 (C, (-), Ar), 131.7 (C, (-), Ar), 129.0 (CH, (+), Ar), 128.4 (CH, (+), Ar), 128.2 (CH, (+), Ar), 127.4 (CH, (+), Ar), 126.9 (CH, (+), Ar), 125.7 (CH, (+), Ar), 125.3 (CH, (+), Ar), 123.9 (CH, (+), Ar), 123.0 (CH, (+), 117.7 (CH_2, (-), CH_2CH*C*H_2), 57.1 (CH, (+), *C*HNH), 51.7 (CH_2, (-), NH*C*H_2), 42.2 (CH_2, (-), naphthCH*C*H_2) IR (neat) ν_{max} = 3324 (w), 3061 (m), 2909 (w), 2831 (w), 1638 (m), 1596 (m), 1453 (m), 1393 (m), 1167 (w), 1075 (w), 1028 (m), 994 (m), 912 (s), 799 (s), 776 (s) cm^{-1} [lit[12] ($CHCl_3$): 3437, 3062, 2928, 2856, 1638, 1597, 1456, 1394, 1167, 1106, 1028, 997, 917, 800, 778 cm^{-1}]; MS TOF ES+ (m/z): 288.2 $[M+H]^+$.

*Synthesis of (*R*)-*N*-benzyl-1-(4-(tert-butyl)phenyl)but-3-en-1-amine **S4c**.*

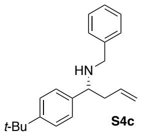
S4c

Prepared using *General Procedure **D*** with (*S*)-1-(4-(*tert*-butyl)phenyl)but-3-en-1-amine **S3c** (296 mg, 1.00 equiv., 1.46 mmol), benzaldehyde (154 mg, 1.00 equiv., 1.46 mmol), sodium borohydride (110 mg, 2.00 equiv., 2.91 mmol) and methanol (20 mL). Purified using a *Combiflash Rf 200i* (silica 4 g column, 0-30% EtOAc/*n*-hexane). Colourless oil (356 mg, 1.21 mmol, 83% yield over two steps). TLC (EtOAc:*n*-hexane = 5:95 v/v, visualised with $KMnO_4$): R_f= 0.32; ^{1}H NMR (400 MHz, $CDCl_3$,): δ 7.37-7.21 (9H, m, Ar*H*), 5.77-5.67 (1H, m, CH_2C*H*CH_2), 5.11-5.03 (2H, m, CH_2CHC*H_2*), 3.67 (1H, t, *J* 6.5, C*H*CH_2CH), 3.69 (1H, d, *J* 13.2, NHC*H_2*Ph), 3.53 (1H, d, *J* 13.3, NHC*H_2*Ph), 2.47-2.35 (2H, m, CHC*H_2*CH), 1.88 (1H, br s, N*H*), 1.33 (9H, s, *t*-Bu); ^{13}C NMR (101 MHz, PENDANT, $CDCl_3$): δ 149.8 (C, (-), Ar), 140.7 (C, (-), Ar), 135.7 (CH, (+), CH_2*C*HCH_2), 128.3 (CH, (+), Ar), 128.2 (CH, (+), Ar), 126.9 (CH, (+), Ar), 126.8 (CH, (+), Ar), 125.3 (CH, (+), Ar), 117.5 (CH_2, (-), CH_2CH*C*H_2), 61.2 (CH, (+), Ph*C*HNH), 51.5 (CH_2, (-), NH*C*H_2Ph), 43.1 (CH_2, (-), PhCH*C*H_2), 34.5 (C, (-),*t*-Bu), 31.5 (CH_3, (+), *t*-Bu); IR (neat) ν_{max} = 3334 (w), 3063 (w), 3028 (w), 2962 (s), 2904 (m), 2867 (m), 2833 (w), 1640 (w), 1603 (m), 1508 (w), 1495 (w), 1457 (m), 1363 (m), 1269 (m), 1203 (w), 1109 (m), 1017 (m), 995 (m), 914

(s), 830 (s), 731 (s), 697 (s) cm^{-1}; MS TOF ES+ (*m/z*): 294.2 [M+H]$^+$; HRMS (m/z): [M+H]$^+$ calcd. for $C_{21}H_{28}N^+$, 294.2216; found, 294.2220.

*Synthesis of (*R*)-*N*-benzyl-1-(4-nitrophenyl)but-3-en-1-amine* ***S4d****.*

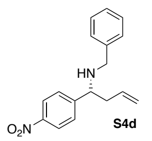

S4d

Prepared using *General Procedure* ***D*** with (*S*)-1-(4-nitrophenyl)but-3-en-1-amine **S3d** (386 mg, 1.00 equiv., 2.01 mmol), benzaldehyde (213 mg, 1.00 equiv., 2.01 mmol), sodium borohydride (152 mg, 2.00equiv., 4.02 mmol) and methanol (20.0 mL). Purified using a *Combiflash Rf 200i* (silica 4 g column, 0-30% EtOAc/*n*-hexane). The characterisation reported is consistent with that given the literature.[11] Pale yellow oil (515 mg, 1.82 mmol, 91% yield over two steps). TLC (EtOAc:*n*-hexane =5:95 v/v, visualised with $KMnO_4$): R_f = 0.18; ^{1}H NMR (400 MHz, $CDCl_3$,): δ 8.20 (2H, d, *J* 8.8, Ar*H*), 7.55 (2H, d, *J* 8.6, Ar*H*), 7.34-7.22 (5H, m, Ar*H*), 5.68-5.63 (1H, m, $CH_2C\mathit{H}CH_2$), 5.09-5.05 (2H, m, $CH_2CHC\mathit{H_2}$), 3.82 (1H, dd, *J* 7.9 & 5.6, $C\mathit{H}CH_2CH$), 3.65 (1H, d, *J* 13.3, NHC*H*H), 3.52 (1H, d, *J* 13.4, NHCH*H*), 2.45-2.32 (2H, m, $CHC\mathit{H_2}CH$), 1.81 (1H, br s, N*H*); ^{13}C NMR (101 MHz, PENDANT, $CDCl_3$): δ 151.9 (C, (-), Ar), 147.2 (C, (-), Ar), 140.0 (C, (-), Ar), 134.3 (CH, (+), $CH_2\mathit{C}HCH_2$), 128.5 (CH, (+), Ar), 128.2 (CH, (+), Ar), 128.0 (CH, (+), Ar), 127.1 (CH, (+), Ar), 123.7 (CH, (+), Ar), 118.6 (CH_2, (-), $CH_2CH\mathit{C}H_2$), 61.2 (CH, (+), *C*HNH), 51.6 (CH_2, (-), $NH\mathit{C}H_2$), 43.0 (CH_2, (-), $CH\mathit{C}H_2CH$); IR (neat) ν_{max} = 3334 (w), 3061 (w), 3027 (w), 2927 (w), 2837 (w), 1640 (w), 1600 (w), 1516 (s), 1454 (m), 1342 (w), 1197 (w), 1108 (s), 995 (s), 918 (s), 854 (s), 823 (m), 736 (s), 697 (s) cm^{-1} [lit[11] ($CHCl_3$): 3335, 3078, 3029, 2924, 2849, 1640, 1599, 1520, 1455, 1347, 1315, 1109, 1014, 996, 920, 856, 824, 754, 700 cm^{-1}]; MS TOF ES+ (*m/z*): 283.2 [M+H]$^+$.

*Synthesis of (*R*)-*N*-benzyl-1-(4-chlorophenyl)but-3-en-1-amine* ***S4e****.*

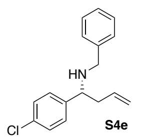

S4e

Prepared using *General Procedure* ***D*** with (*S*)-1-(4-chlorophenyl)but-3-en-1-amine **S3e** (189 mg, 1.00 equiv., 1.04 mmol), benzaldehyde (110 mg, 1.00 equiv., 1.04 mmol), sodium borohydride (79.1 mg, 2.00 equiv., 2.08 mmol) and methanol (20 mL). Purified using a *Combiflash Rf 200i* (silica 4 g column, 0-30% EtOAc/*n*-hexane). The characterisation reported is consistent with that given the literature.[12] Colourless oil (268 mg, 0.986 mmol, 95% yield over two steps). TLC (EtOAc:*n*-hexane =20:80 v/v, visualised with $KMnO_4$): R_f = 0.55; ^{1}H NMR (300 MHz, $CDCl_3$,): δ 7.34-7.21 (9H, m, Ar*H*), 5.74-5.60 (1H, m, $CH_2C\mathit{H}CH_2$), 5.09-5.03 (2H, m, $CH_2CHC\mathit{H_2}$), 3.69-3.63 (2H, m, $C\mathit{H}CH_2CH$ & NHCH*H*), 3.49 (1H, d, *J* 13.3, NHCH*H*), 2.39-2.33 (2H, m, $CHC\mathit{H_2}CH$)), 1.73 (1H, br s, N*H*); ^{13}C NMR (101 MHz, PENDANT, $CDCl_3$): δ 142.4 (C, (-), Ar), 140.4 (C, (-), Ar), 135.0 (CH, (+), $CH_2\mathit{C}HCH_2$), 132.6 (C, (-), Ar), 128.7 (CH, (+), Ar), 128.6 (CH, (+), Ar), 128.4 (CH, (+), Ar), 128.1 (CH, (+), Ar), 127.0 (CH, (+), Ar), 118.0 (CH_2, (-),$CH_2CH\mathit{C}H_2$), 61.0 (CH, (+), Ph*C*HNH), 51.4 (CH_2, (-), $NH\mathit{C}H_2Ph$), 43.1 (CH_2, (-), $CH\mathit{C}H_2CH$); IR (neat) ν_{max} = 3335 (w), 3064 (w), 2979 (w), 2909 (w),2832 (w), 1639 (m), 1598 (w), 1491 (s), 1454 (s), 1343 (w), 1090 (s), 1014 (s), 916 (s), 824 (s), 697 (s) cm^{-1} [lit[12] ($CHCl_3$): 3396, 3077, 2931, 2844, 1646, 1460, 1046, 917, 765 cm^{-1}]; MS TOF ES+ (*m/z*): 272.1 [M+H]$^+$; HRMS (m/z): [M+H]$^+$ calcd. for $C_{17}H_{19}N^{35}Cl^+$, 272.1201; found, 272.1205.

*Synthesis of (*R*)-*N*-(4-chlorobenzyl)-1-phenylbut-3-en-1-amine* ***S4f***.

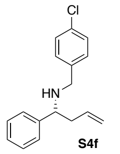
S4f

Prepared using *General Procedure* ***D*** with (*S*)-1-phenylbut-3-en-1-amine **S3a** (150 mg, 1.02 mmol), 4-chlorobenzaldehyde (143 mg, 1.02 mmol), sodium borohydride (77.1 mg, 2.04 mmol) and methanol (20 mL). Purified using a *Combiflash Rf 200i* (silica 4 g column, 0-30% EtOAc/*n*-hexane). Colourless oil (230 mg, 0.846 mmol, 83% yield over two steps). TLC (EtOAc:*n*-hexane = 20:80 v/v, visualised with $KMnO_4$): R_f = 0.57; ^{1}H NMR (300 MHz, $CDCl_3$,): δ 7.35-7.17 (9H, m, Ar*H*), 5.77-5.63 (1H, m, CH_2C*H*CH_2), 5.11-5.02 (2H, m, CH_2CHC*H_2*), 3.68 (1H, t, *J* 7.6, C*H*CH_2CH), 3.66 (1H, d, *J* 14.6, NHC*H_2*), 3.49 (1H, d, *J* 13.5, NHC*H_2*), 2.48-2.32 (2H, m, CHC*H_2*CH), 1.68 (1H, br s, N*H*); ^{13}C NMR (101 MHz, PENDANT, $CDCl_3$): δ 143.6 (C, (-), Ar), 139.1 (C, (-), Ar), 135.4 (CH, (+), CH_2*C*HCH_2), 132.5 (C, (-), Ar), 129.5 (CH, (+), Ar), 128.5 (CH, (+), Ar), 127.3 (CH, (+), Ar), 127.2 (CH, (+), Ar), 117.7 (CH_2, (-), CH_2CH*C*H_2), 61.6 (CH, (+), *C*HNH), 50.7 (CH_2, (-), NH*C*H_2), 43.1 (CH_2, (-), CH*C*H_2); IR (neat) ν_{max} = 3328 (w), 3063 (w), 3027 (w), 2978 (w), 2912 (w), 2834 (w), 1639 (m), 1599 (w), 1490 (s), 1453 (s), 1407 (w), 1356 (w), 1197 (w), 1090 (s), 1015 (s), 995 (m), 915 (s), 804 (s), 700 (s) cm^{-1}; MS TOF ES+ (*m/z*): 272.1 $[M+H]^+$; HRMS (m/z): $[M+H]^+$ calcd. for $C_{17}H_{19}N^{35}Cl^+$, 272.1201; found, 272.1204.

*Synthesis of (*R*)-*N*-neopentyl-1-phenylbut-3-en-1-amine* ***S4g***.

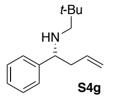
S4g

Prepared using *General Procedure* ***D*** with (*S*)-1-phenylbut-3-en-1-amine **S3a** (150 mg, 1.00 equiv., 1.02 mmol), trimethylacetaldehyde (87.8 mg, 1.00 equiv., 1.02 mmol), sodium borohydride (77.1 mg, 2.00 equiv., 2.04 mmol) and methanol (20 mL). Purified using a *Combiflash Rf 200i* (silica 4 g column, 0-30% EtOAc/*n*-hexane). Colourless oil (150 mg, 0.690 mmol, 68% yield over two steps). TLC (EtOAc:*n*-hexane =15:85 v/v, visualised with $KMnO_4$): R_f = 0.48; ^{1}H NMR (400 MHz, $CDCl_3$,): δ 7.34-7.20 (5H, m, Ar*H*), 5.81-5.70 (1H, m, CH_2C*H*CH_2), 5.11-5.03 (2H, m, CH_2CHC*H_2*), 3.58 (1H, dd, *J* 8.1 & 5.6, C*H*CH_2CH), 2.44-2.29 (2H, m, CHC*H_2*CH), 2.17 (2H, q, *J* 9.6, NHC*H_2t*-Bu), 1.38 (1H, br s, N*H*), 0.87 (9H, s, *t*-Bu); ^{13}C NMR (101 MHz, PENDANT, $CDCl_3$): δ 144.8 (C, (-), Ar), 135.9(CH, (+), CH_2*C*HCH_2), 128.2 (CH, (+), Ar), 127.2 (CH, (+), Ar), 126.7 (CH, (+), Ar), 117.2 (CH_2, (-), CH_2CH*C*H_2), 63.1 (CH, (+), *C*HNH), 60.0 (CH_2, (-), NH*C*H_2*t*-Bu), 43.5 (CH_2, (-), PhCH*C*H_2), 31.6 (C, (-), *t*-Bu), 27.8 (CH_3, (+), *t*-Bu); IR (neat) ν_{max} = 3349 (w), 3075 (w), 3027 (w), 2952 (s), 2906 (m), 2866 (m), 2798 (w), 1639 (m), 1602 (m), 1474 (m), 1454 (m), 1395 (w), 1362 (m), 1283 (w), 1189 (w), 1122 (m), 1069 (w), 994 (m), 915 (s), 755 (s), 699 (s) cm^{-1}; MS TOF ES+ (*m/z*): 218.2 $[M+H]^+$; HRMS (m/z): $[M+H]^+$ calcd. for $C_{15}H_{24}N^+$, 218.1903; found, 218.1910.

*Synthesis of (*R*)-*N*-(3,5-bis(trifluoromethyl)benzyl)-1-phenylbut-3-en-1-amine* ***S4h***.

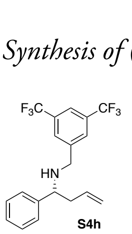
S4h

Prepared using *General Procedure* ***D*** with (*S*)-1-phenylbut-3-en-1-amine **S3a** (150 mg, 1.00 equiv., 1.02 mmol), 3,5-bis(trifluoromethyl)benzaldehyde (247 mg, 1.00 equiv., 1.02 mmol), sodium borohydride (77.1 mg, 2.00 equiv., 2.04 mmol) and methanol (20 mL). Purified using a *Combiflash Rf 200i* (silica 4 g column, 0-30% EtOAc/*n*-hexane). Colourless oil (250 mg, 0.670 mmol, 56% yield over two steps). TLC (EtOAc:*n*-hexane =10:90 v/v, visualised with $KMnO_4$): R_f = 0.45. ^{1}H NMR (300 MHz, $CDCl_3$,): δ7.72 (3H, s, Ar*H*), 7.38-7.23

(5H, m, Ar*H*), 5.81-5.67 (1H, m, $CH_2C\mathit{H}CH_2$), 5.15-5.06 (2H, m, $CH_2CHC\mathit{H_2}$), 3.78-3.63 (3H, m, $C\mathit{H}CH_2CH$ & $NHC\mathit{H_2}$), 2.51-2.36 (2H, m, $CHC\mathit{H_2}CH$), 1.82 (1H, br s, N*H*); ^{13}C NMR (101 MHz, PENDANT, $CDCl_3$): δ 143.4 (C, (-), Ar), 143.2 (C, (-), Ar), 135.2(CH, (+), $CH_2\mathit{C}HCH_2$), 131.4 (C, (-), q, $^3J_{C\text{-}F}$ = 33.2, Ar), 128.6 (CH, (+), Ar), 128.2 (CH, (+), Ar), 127.4 (CH, (+), Ar), 127.2 (CH, (+), Ar), 123.5 (C, q, $^1J_{C\text{-}F}$ = 272.1, CF_3), 120.8 (CH, (+), q, $^2J_{C\text{-}F}$ = 3.4, Ar), 117.9 (CH_2, (-), $CH_2CH\mathit{C}H_2$), 62.1 (CH, (+), Ph*C*HNH), 50.6 (CH_2, (-), $NH\mathit{C}H_2$), 43.0 (CH_2, (-), $PhCH\mathit{C}H_2$). ^{19}F NMR (282 MHz, $CDCl_3$): δ -62.8 (s); IR (neat) ν_{max} = 3343 (w), 3082 (w), 3030 (w), 2983 (w), 2918 (w), 2838 (w), 2801 (w), 1641 (w), 1623 (w), 1493 (w), 1455 (w), 1355 (w), 1383 (w), 1275 (s), 1168 (s), 1125 (s), 1028 (w), 996 (w), 918 (m), 891 (m), 843 (m), 760 (m), 732 (w), 700 (s), 681 (s) cm^{-1}; MS TOF ES+ (*m/z*): 374.1 $[M+H]^+$; HRMS (m/z): $[M+H]^+$ calcd. for $C_{19}H_{18}NF_6$, 374.1338; found, 374.1344.

*Synthesis of (*R*)-*N*-(4-(*tert*-butyl)benzyl)-1-phenylbut-3-en-1-amine* ***S4i***.

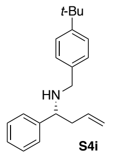

Prepared using *General Procedure **D*** with (*S*)-1-phenylbut-3-en-1-amine **S3a** (150 mg, 1.00 equiv., 1.02 mmol), 4-*tert*-buthylbenzaldehyde (165 mg, 1.00equiv., 1.00 mmol), sodium borohydride (77.1 mg, 2.00 equiv., 2.04 mmol) and methanol (20 mL). Purified using a *Combiflash Rf 200i* (silica 4 g column, 0-30% ethyl acetrate/*n*-hexane). Colourless oil (259 mg, 0.883 mmol, 87% yield over two steps). TLC (EtOAc:*n*-hexane =15:85 v/v, visualised with $KMnO_4$): R_f = 0.42; ^1H NMR (400 MHz, $CDCl_3$,): δ 7.38-7.23 (7H, m, Ar*H*), 7.19 (2H, d, *J* 8.2, Ar*H*), 5.77-5.66 (1H, m, $CH_2C\mathit{H}CH_2$), 5.10-5.02 (2H, m, $CH_2CHC\mathit{H_2}$), 3.71 (1H, dd, *J* 7.7 & 6.0, $C\mathit{H}CH_2CH$), 3.63 (1H, d, *J* 13.1, $NHC\mathit{H_2}Ph$), 3.50 (1H, d, *J* 13.2, $NHC\mathit{H_2}Ph$), 2.47-2.35 (2H, m, $CHC\mathit{H_2}CH$), 1.69 (1H, br s, N*H*), 1.31 (9H, s, *t*-Bu); ^{13}C NMR (101 MHz, PENDANT, $CDCl_3$): δ 149.7 (C, (-), Ar), 144.0 (C, (-), Ar), 137.7 (C, (-), Ar), 135.6 (CH, (+), $CH_2\mathit{C}HCH_2$), 128.4 (CH, (+), Ar), 127.8 (CH, (+), Ar), 127.3 (CH, (+), Ar), 127.0 (CH, (+), Ar), 125.3 (CH, (+), Ar), 117.5 (CH_2, (-), $CH_2CH\mathit{C}H_2$), 61.8 (CH, (+), Ph*C*HNH), 51.1 (CH_2, (-), $NH\mathit{C}H_2$), 43.2 (CH_2, (-), $PhCH\mathit{C}H_2$), 34.5 (C, (-), *t*-Bu), 31.4 (CH_3, (+), *t*-Bu); IR (neat) ν_{max} = 3334 (w), 3061 (w), 3026 (w), 2962 (m), 2095 (w), 2868 (w), 2832 (w), 1640 (w), 1603 (w), 1513 (m), 1492 (m), 1453 (s), 1363 (m), 1269 (m), 1202 (w), 1111 (m), 1015 (w), 995 (w), 915 (m), 831 (m), 758 (s) cm^{-1}; MS TOF ES+ (*m/z*): 294.2 $[M+H]^+$, 316.2 $[M+Na]^+$; HRMS (m/z): $[M+H]^+$ calcd. for $C_{21}H_{28}N^+$, 294.2216; found, 294.2224.

*Synthesis of (*R*)-*N*-(4-methoxybenzyl)-1-phenylbut-3-en-1-amine* ***S4j***.

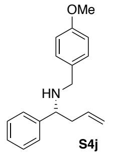

Prepared using *General Procedure **D*** with (*S*)-1-phenylbut-3-en-1-amine **S3a** (0.150 g, 1.00 equiv., 1.02 mmol), 4-methoxybenzaldehyde (139 mg, 1.00 equiv., 1.02 mmol), sodium borohydride (77.1 mg, 2.00 equiv., 2.04 mmol) and methanol (20 mL). Purified using a *Combiflash Rf 200i* (silica 4 g column, 0-30% EtOAc/*n*-hexane). The characterisation reported is consistent with that in the literature.[12] Colourless oil (178 mg, 0.485 mmol, 48% yield over two steps). TLC (EtOAc:*n*-hexane =10:90 v/v, visualised with $KMnO_4$): R_f = 0.23; ^1H NMR (300 MHz, $CDCl_3$,): δ 7.36-7.15 (7H, m, Ar*H*), 6.84 (2H, d, *J* 8.7, Ar*H*), 5.74-5.63 (1H, m, $CH_2C\mathit{H}CH_2$), 5.10-5.01 (2H, m, $CH_2CHC\mathit{H_2}$), 3.79 (3H, s, OMe), 3.68 (1H, t, *J* 7.6, $C\mathit{H}CH_2CH$), 3.61 (1H, d, *J* 13.0, $NHCH\mathit{H}$), 3.45 (1H, d, *J* 13.0, $NHC\mathit{H}H$), 2.47-2.32 (2H, m, $CHC\mathit{H_2}CH$), 1.69 (1H, br s, N*H*); ^{13}C NMR (101 MHz, PENDANT,

$CDCl_3$): δ 158.5 (C, (-), Ar), 143.9 (C, (-), Ar), 135.5(CH, (+), CH_2*C*HCH_2), 132.8 (C, (-), Ar), 129.3 (CH, (+), Ar), 128.4 (CH, (+), Ar), 127.3 (CH, (+), Ar), 127.0 (CH, (+), Ar), 117.5 (CH_2, (-), CH_2CH*C*H_2), 113.7 (CH, (+), Ar), 61.5 (CH, (+), Ph*C*HNH), 55.3 (CH_3, (+), OMe), 50.8 (CH_2, (-), NH*C*H_2), 43.1 (CH_2, (-), PhCH*C*H_2); IR (neat) ν_{max} = 3325 (w), 3063 (w), 3027 (w), 3001 (w), 2936 (w), 2907 (w), 2834 (w), 1639 (w), 1611 (m), 1585 (w), 1510 (s), 1453 (m), 1355 (w), 1301 (m), 1244 (s), 1173 (m), 1105 (m), 1035 (s), 995 (m), 915 (s), 817 (s), 775 (s) cm^{-1}; MS TOF ES+ (*m/z*): 268.2 $[M+H]^+$.

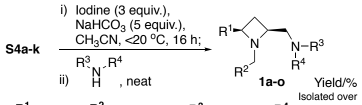

	R¹	R²	R³	R⁴	Yield/% Isolated over two steps
a	Ph	Ph	*i*-Pr	H	12
b	1-Naphth	Ph	*i*-Pr	H	4
c	4-*t*-Bu-C_6H_4	Ph	*i*-Pr	H	6
d	4-NO_2-C_6H_4	Ph	*i*-Pr	H	18
e	4-Cl-C_6H_4	Ph	*i*-Pr	H	22
f	Ph	4-Cl-C_6H_4	*i*-Pr	H	15
g	Ph	*t*-Bu	*i*-Pr	H	32
h	Ph	3,5-$(CF_3)_2$-C_6H_3	*i*-Pr	H	25
i	Ph	4-*t*-Bu-C_6H_4	*i*-Pr	H	38
j	Ph	4-MeO-C_6H_4	*i*-Pr	H	43
k	4-Cl-C_6H_4	Ph	*n*-Pr	H	45
l	4-Cl-C_6H_4	Ph	Bn	H	38
m	4-Cl-C_6H_4	Ph	*N*-Pyrrolidine		36
n	4-Cl-C_6H_4	Ph	4-Cl-Bn	H	31
o	4-Cl-C_6H_4	Ph	4-MeO-Bn	H	42

Scheme S 4*. General procedure D, two-step conversion of homoallyl amines* ***S4a-j*** *into azetidine derivatives* ***1a-o****.*

Synthesis of N*-(((2*S*,4*R*)-1-benzyl-4-phenylazetidin-2-yl)methyl)propan-2-amine* ***1a****.*

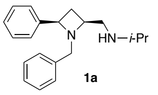

Prepared using *General Procedure E* with (*R*)-*N*-benzyl-1-phenylbut-3-en-1-amine (216 mg, 1.00 equiv., 0.910 mmol), iodine (693 mg, 3.00 equiv., 2.73 mmol), $NaHCO_3$ (382 mg, 5.00 equiv., 4.55 mmol), acetonitrile (30 mL) and isopropyl amine (269 mg, 5.00 equiv., 4.55 mmol). Purified by flash column chromatography (silica gel 40-63 µm, 0-100% EtOAc/*n*-hexane) and further triturated with 70% EtOAc/*n*-hexane (v/v). White powder (33.0 mg, 0.112 mmol, 12% yield over two steps). $[\alpha]_D^{21}$ +9.2 (*c* 1.00, CH_2Cl_2); mp: 153-154 °C; TLC (MeOH:EtOAc =10:90 v/v, visualised with $KMnO_4$); ^{1}H NMR (300 MHz, $CDCl_3$,): 7.48-7.30 (10H, m, Ar*H*), 4.23 (1H, t, *J* 8.2, PhC*H*CH_2), 3.90 (1H, d, *J* 12.4, PhC*H*HN), 3.87-3.78 (1H, m, NC*H*CH_2NH), 3.53 (1H, d, *J* 12.4, PhCH*H*N), 2.99 (1H, dd, *J* 12.6 & 3.7, NCHC*H*H), 2.69-2.57 (2H, m, NHC*H*$(CH_3)_2$ & PhCHC*H*H), 2.49-2.40 (2H, m, & PhCHC*H*H & NCHCH*H*), 1.26 (3H, d, *J* 6.5, Me), 1.19 (3H, d, *J* 6.6, Me); ^{13}C NMR (101 MHz, $CDCl_3$): δ 140.9 (C, (-), Ar), 138.2 (C, (-), Ar), 129.6 (CH, (+), Ar), 129.5 (CH, (+), Ar), 128.8 (CH, (+), Ar), 128.5 (CH, (+), Ar), 128.1 (CH, (+), Ar), 127.0 (CH, (+), Ar), 65.8 (CH, (+), Ph*C*HCH_2), 60.5 (CH_2, (-), Ph*C*H_2N), 58.1 (CH, (+), N*C*HCH_2), 52.2 (CH, (+), *C*H$(CH_3)_2$), 47.8 (CH_2, (-), CH*C*H_2NH), 30.2 (CH_2, (-), CH*C*H_2CH), 19.8 (CH_3, (+), Me), 18.1 (CH_3, (+), Me); IR (powder) ν_{max} = 2954 (s), 2798 (s), 2477 (w), 1604 (w), 1569 (w), 1494 (m), 1454 (m), 1396 (m), 1364 (w), 1308 (w), 1212 (w), 1153 (m), 1007 (m), 753 (s), 697 (s) cm^{-1}; MS TOF EI+ (*m/z*): 294.2 $[M]^+$; HRMS (m/z): $[M]^+$ calcd. for $C_{20}H_{26}N_2^+$, 294.2091; found, 294.2094

Synthesis of N*-(((2*S*,4*R*)-1-benzyl-4-(naphthalen-1-yl)azetidin-2-yl)methyl) propan-2-amine* ***1b****.*

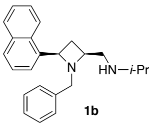

Prepared using *General Procedure E* with (*R*)-*N*-benzyl-1-(naphthalen-1-yl)but-3-en-1-amine (272 mg, 1.00 equiv., 0.946 mmol), iodine (721 mg, 3.00 equiv., 2.84 mmol), $NaHCO_3$ (398 mg, 5.00equiv., 4.73 mmol), acetonitrile (30 mL) and isopropyl amine (0.405 mL, 5.00 equiv., 4.73 mmol). Purified by flash column chromatography (silica gel 40-63 μm, 0-20% MeOH/EtOAc (v/v)) and further triturated with 70% EtOAc/*n*-hexane (v/v). White powder (13.0 mg, 0.0377 mmol, 4% yield over two steps); $[\alpha]_D^{21}$ +33.3 (*c* 1.00, CH_2Cl_2); mp: 169-172 °C; TLC (100% EtOAc, visualised with $KMnO_4$): R_f = 0.47; ^{1}H NMR (400 MHz, DMSO d_6): δ; 9.25-9.0 (1H (measured 1.8H), br s), 8.28 (1H, d, *J* 7.9, Ar*H*), 7.96 (1H, d, *J* 7.7, Ar*H*), 7.84 (1H, d, *J* 8.1, Ar*H*), 7.69 (1H, d, *J* 7.0, Ar*H*), 7.60-7.44 (8H, m, Ar*H*), 4.68 (1H, t, *J* 7.0, ArC*H*CH_2), 4.21 (2H, app br s, PhC*H*HN & NC*H*CH_2NH), 3.87-3.83 (1H, m, NC*H*CH), 3.53 (1H, t, *J* 8.3, ArCH*H*N), 2.84-2.77 (2H, m, NHC*H*$(CH_3)_2$ & CHCH*H*CH), 2.03-1.96 (1H, m, CHC*H*HCH), 1.24-1.20 (1H, m, C*H*), 1.04 (3H, d, *J* 6.5, C*H*$_3$), 0.94 (3H, d, *J* 6.5, C*H*$_3$); ^{13}C NMR (101 MHz, DMSO d_6): δ 134.1 (CH, Ar), 132.6 (CH, Ar), 131.2 (CH, Ar), 130.4 (CH, Ar), 129.5 (CH, Ar), 129.3 (CH, Ar), 128.8 (C, Ar), 127.7 (C, Ar), 126.5 (CH, Ar), 126.2 (CH, Ar), 126.1 (CH, Ar), 124.5 (C, Ar), 123.6 (CH, Ar), 59.4 (CH, naphth *C*HCH_2), 54.8 (CH, N*C*HCH_2), 49.7 (CH_2, Ph*C*H$_2$N), 49.2 (CH_2, NCH*C*H$_2$NH), 48.5 (CH, NH*C*H$(CH_3)_2$), 37.9 (CH_2, CH*C*H$_2$CH), 22.3 (CH_3, Me), 15.0 (CH_3, Me), *one quaternary carbon not observed*; IR (neat) ν_{max} = 2963 (s), 2933 (s), 2803 (s), 2720 (m), 1594 (m), 1564 (s), 1516 (w), 1498 (w), 1459 (s), 1437 (s), 1393 (m), 1369 (m), 1333 (m), 1267 (w), 1246 (w), 1123 (m), 1090 (m), 1033 (m), 1006 (m) 922 (w) cm^{-1}; MS TOF ES+ (*m/z*): 345.23 $[M+H]^+$; HRMS (m/z): $[M+H]^+$ calcd. for $C_{24}H_{29}N_2^+$, 345.2325; found, 345.2334.

Synthesis of N*-(((2*S*,4*R*)-1-Benzyl-4-(4-(*tert*-butyl)phenyl)azetidin-2-yl)methyl) propan-2-amine* ***1c****.*

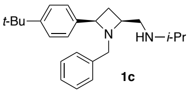

Prepared using *General Procedure E* with (*R*)-*N*-benzyl-1-(4-(*tert*-butyl)phenyl)but-3-en-1-amine (356 mg, 1.0 equiv., 1.21 mmol), iodine (924 mg, 3.0 equiv., 3.64 mmol), $NaHCO_3$ (510 mg, 5.0 equiv., 6.07 mmol), acetonitrile (15 mL) and isopropyl amine (0.52 mL, 5.0 equiv., 6.1 mmol). Purified by flash column chromatography (silica gel 40-63 μm 100% EtOAc) and washed with 70% EtOAc/*n*-hexane (v/v). White powder (25.4 mg, 0.0725 mmol, 6% yield over two steps). $[\alpha]_D^{21}$ +9.8 (*c* 1.00, CH_2Cl_2); mp: 137-139 °C; TLC (100%EtOAc, visualised with $KMnO_4$): R_f = 0.23; ^{1}H NMR (300 MHz, $CDCl_3$,): δ 7.50-7.20 (9H, m, Ar*H*), 4.22 (1H, t, *J* 8.2, PhC*H*CH_2), 3.93 (1H, d, *J* 12.3, PhC*H*HN), 3.80-3.74 (1H, m, NC*H*CH_2NH), 3.50 (1H, d, *J* 12.3, PhCH*H*N), 3.05 (1H, dd, *J* 12.5 & 3.5, CHC*H*HNH), 2.64-2.43 (3H, m, NHC*H*$(CH_3)_2$, CHC*H*HCH & CHC*H*HNH), 2.33 (1H, dd, *J* 12.5 & 4.2, CHCH*H*CH), 1.34 (9H, s, *t*-Bu), 1.27 (3H, d, *J* 6.6, Me), 1.19 (3H, d, *J* 6.5, Me); ^{13}C NMR: (101 MHz, PENDANT, $CDCl_3$): δ 151.0 (C, (-), Ar), 138.2 (C, (-), Ar), 137.9 (C, (-), Ar), 129.6 (CH, (+), Ar), 129.4 (CH, (+), Ar), 128.4 (CH, (+), Ar), 126.7 (CH, (+), Ar), 125.6 (CH, (+), Ar), 65.6 (CH, (+), Ph*C*HCH_2), 60.5 (CH_2, (-), Ph*C*H$_2$N), 58.1 (CH, (+), N*C*HCH_2), 50.4 (CH, (+), *C*H$(CH_3)_2$), 47.7 (CH_2, (-), CH*C*H$_2$NH), 34.6 (C, (-), *t*-Bu), 31.4 (CH_3, (+), *t*-Bu), 30.4 (CH_2, (-), CH*C*H$_2$CH), 19.8 (CH_3, (+), Me), 18.1 (CH_3, (+), Me); IR (neat) ν_{max} =

3020 (m), 2958 (s), 2868 (s), 2708 (s), 1570 (w), 1510 (w), 1454 (m), 1393 (m), 1362 (m), 1306 (m), 1269 (s), 1207 (w), 1152 (m), 1110 (m), 1017 (m), 924 (w), 831 (s), 736 (s), 702 (s) cm^{-1}; MS TOF EI+ (*m/z*): 350.3 $[M]^+$; HRMS (m/z): $[M]^+$ calcd. for $C_{24}H_{34}N_2^+$, 350.2717; found, 350.2725.

Synthesis of N-(((2S,4R)-1-benzyl-4-(4-nitrophenyl)azetidin-2-yl)methyl) propan-2-amine ***1d****.*

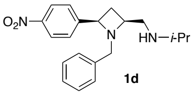

1d

Prepared using *General Procedure E* with (*R*)-*N*-benzyl-1-(4-nitrophenyl)but-3-en-1-amine (515 mg, 1.0 equiv., 1.82 mmol), iodine (1.39 g, 3.0 equiv., 5.5 mmol), $NaHCO_3$ (770 mg, 5.0 equiv., 9.12 mmol), acetonitrile (30 mL) and isopropyl amine (0.75 mL, 5.0 equiv., 9.1 mmol). Purified by flash column chromatography (silica gel 40-63 μm, 100% EtOAc). Colourless crystalline solid (113 mg, 0.333 mmol, 18% yield over two steps). TLC (100% EtOAc, visualised with $KMnO_4$): R_f = 0.21; $[\alpha]_D^{21}$ +11.1 (*c* 1.00, CH_2Cl_2); mp: 155-157 °C; ^{1}H NMR (400 MHz, $CDCl_3$,): δ 8.21 (2H, d, *J* 8.7, Ar*H*), 7.63 (2H, d, *J* 8.7, Ar*H*), 7.42-7.31 (5H, m, Ar*H*), 4.28 (1H, t, *J* 8.2, PhC*H*CH_2), 3.96-3.89 (1H, m, NC*H*CH_2NH), 3.84 (1H, d, *J* 12.3, PhC*H*HN), 3.61 (1H, d, *J* 12.2, PhCH*H*N), 3.04-2.94 (1H, m, NHC*H*$(CH_3)_2$), 2.89 (1H, dd, *J* 12.7 & 3.8, CHC*H*HNH), 2.77 (1H, dt, *J* 11. & 7.6, NCHC*H*H), 2.61 (1H, dd, *J* 12.7 & 5.4, CHC*H*HNH), 2.35 (1H, dt, *J* 11.2 & 8.6, NCHCH*H*), 1.29 (3H, d, *J* 6.6, Me), 1.21 (3H, d, *J* 6.6, Me); ^{13}C NMR (101 MHz, PENDANT, $CDCl_3$): δ 148.6 (C, (-), Ar), 147.5 (C, (-), Ar), 137.3 (C, (-), Ar), 129.7 (CH, (+), Ar), 129.4 (CH, (+), Ar), 128.5 (CH, (+), Ar), 127.7 (CH, (+), Ar), 123.9 (CH, (+), Ar), 64.8 (CH, (+), Ph*C*HCH_2), 60.9 (CH_2, (-), Ph*C*H_2N), 58.1 (CH, (+), N*C*HCH_2), 52.0 (CH, (+), *C*H$(CH_3)_2$), 47.6 (CH_2, (-), CH*C*H_2NH), 31.5 (CH_2, (-), CH*C*H_2CH), 19.3 (CH_3, (+), Me), 18.2 (CH_3, (+), Me); IR (neat) ν_{max} = 3030 (m), 2939 (s), 2787 (s), 2705 (m), 1600 (m), 1516 (s), 1454 (m), 1393 (m), 1343 (s), 1289 (w), 1269 (w), 1245 (w), 1212 (w), 1151 (w), 1108 (m), 1070 (w), 844 (m), 978 (w), 921 (w), 854 (s), 782 (w), 733 (s), 798 (s) cm^{-1}; MS TOF ES+ (*m/z*): 340.2 $[M+H]^+$; HRMS (m/z): $[M+H]^+$ calcd. for $C_{20}H_{26}N_3O_2^+$, 340.2020; found, 340.2029.

Synthesis of N-(((2R,4S)-1-Benzyl-4-(4-chlorophenyl)azetidin-2-yl)methyl) propan-2-amine ***1e****.*

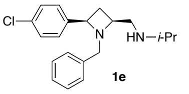

1e

Prepared using *General Procedure E* with (*R*)-*N*-benzyl-1-(4-chlorophenyl)but-3-en-1-amine (268 mg, 1.00 equiv., 0.99 mmol), iodine (751 mg, 3.00 equiv., 2.96 mmol), $NaHCO_3$ (414 mg, 5.00 equiv., 4.93 mmol), acetonitrile (30 mL) and isopropyl amine (0.425 mL, 5.00 equiv., 4.93 mmol). Purified by flash column chromatography (silica gel 40-63 μm, 100% EtOAc). White powder (71.0 mg, 22% yield over two steps). TLC (MeOH:EtOAc =10:90 v/v, visualised with $KMnO_4$): R_f = 0.49; $[\alpha]_D^{21}$ +4.4 (*c* 1.00, CH_2Cl_2); mp: 160-162 °C; ^{1}H NMR (400 MHz, $CDCl_3$,): δ 7.43-7.33 (9H, m, Ar*H*), 4.16 (1H, t, *J* 8.2, PhC*H*CH_2), 3.84 (1H, d, *J* 12.4, PhC*H*HN), 3.86-3.80 (1H, m, NC*H*CH_2NH), 3.52 (1H, d, *J* 12.3, PhCH*H*N), 2.92 (1H, dd, *J* 12.6 & 3.5, NCHC*H*H), 2.80-2.71 (1H, m, NHC*H*$(CH_3)_2$), 2.67-2.61 (1H, m, CHCH*H*CH), 2.48 (1H, dd, *J* 12.6 & 5.0, NCHC*H*H), 2.43-2.36 (1H, m, CHC*H*HCH), 1.27 (3H, d, *J* 6.7, Me), 1.19 (3H, d, *J* 6.6, Me); ^{13}C NMR (101 MHz, $CDCl_3$): δ 139.5 (C, (-), Ar), 137.8 (C, (-), Ar), 133.7 (C, (-), Ar), 129.6 (CH, (+,), Ar), 129.4 (CH, (+), Ar), 128.9 (CH, (+), Ar), 128.5 (CH, (+), Ar), 128.5 (CH, (+), Ar), 65.2 (CH, (+), Ph*C*HCH_2), 60.5 (CH_2, (-), Ph*C*H_2N), 58.1 (CH, (+), N*C*HCH_2), 52.2 (CH, (+), *C*H$(CH_3)_2$), 47.7 (CH_2, (-), CH*C*H_2NH), 30.8 (CH_2, (-), CH*C*H_2CH), 19.6 (CH_3, (+), Me), 18.1

(CH_3, (+), Me); IR (neat) ν_{max} = 2934 (s), 2791 (s), 2705 (m), 1597 (m), 1572 (m), 1490 (s), 1454 (s), 1393 (s), 1294 (w), 1275 (w), 1245 (w), 1212 (w), 1153 (s), 1088 (s), 1013 (s), 979 (w), 943 (w), 922 (w), 823 (s), 785 (s), 742 (s), 703 (s) cm^{-1}; MS TOF ES+ (*m/z*): 329.2 $[M+H]^+$; HRMS (m/z): $[M+H]^+$ calcd. for $C_{20}H_{26}N_2{}^{35}Cl^+$, 329.1779; found, 329.1786.

*Synthesis of N-(((2S,4R)-1-(4-Chlorobenzyl)-4-phenylazetidin-2-yl)methyl) propan-2-amine **1f**.*

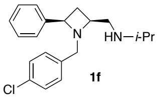
1f

Prepared using *General Procedure E* with (*R*)-*N*-(4-chlorobenzyl)-1-phenylbut-3-en-1-amine (150 mg, 1.0 equiv., 0.55 mmol), iodine (420 mg, 3.0 equiv., 1.7 mmol), $NaHCO_3$ (232 mg, 5.0 equiv., 2.8 mmol), acetonitrile (30 mL) and isopropyl amine (0.23 mL, 5.0 equiv., 2.8 mmol). Purified using a *Combiflash Rf 200i* (silica 4 g column, 0-100% EtOAc/*n*-hexane (v/v)). White powder (28 mg, 0.09 mmol, 15% yield over two steps). TLC (MeOH: CH_2Cl_2 = 2:98 v/v, visualised with $KMnO_4$): R_f = 0.23; $[\alpha]_D^{21}$ +7.3 (*c* 1.00, CH_2Cl_2); mp: 164-166 °C; ^1H NMR (400 MHz, $CDCl_3$,); 7.42-7.27 (9H, m, Ar*H*), 4.14 (1H, t, *J* 8.2, PhC*H*CH_2), 3.88-3.82 (2H, m, NC*H*CH_2NH & PhC*H*HN), 3.58 (1H, d, *J* 12.7, PhCH*H*N), 3.08 (1H, hept, NHC*H*$(CH_3)_2$), 2.87 (1H, dd, *J* 12.6 & 3.5, CHC*H*HNH), 2.76-2.64 (2H, m, NCHC*H*H & CHC*H*HNH), 2.26 (1H, dt, *J* 11.2 & 8.6, NCHCH*H*), 1.34 (3H, d, *J* 6.6, Me), 1.29 (3H, d, *J* 6.6, Me); ^{13}C NMR (101 MHz, PENDANT, $CDCl_3$): δ 141.2 (C, (-), Ar), 136.3 (C, (-), Ar), 134.0 (C, (-), Ar), 131.1 (CH, (+), Ar), 129.2 (CH, (+), Ar), 128.6 (CH, (+), Ar), 127.9 (CH, (+), Ar), 126.8 (CH, (+), Ar), 65.7 (CH, (+), Ph*C*HCH_2), 59.8 (CH_2, (-), Ph*C*H_2N), 57.9 (CH, (+), N*C*HCH_2), 52.0 (CH, (+), *C*H$(CH_3)_2$), 47.8 (CH_2, (-), CH*C*H_2NH), 31.9 (CH_2, (-), CH*C*H_2CH), 19.3 (CH_3, (+), Me), 18.4 (CH_3, (+), Me); IR (neat) ν_{max} = 3026 (m), 2958 (s), 2818 (m), 1598 (w), 1571 (w), 1492 (m), 1458 (w), 1433 (w), 1406 (w), 1309 (w), 1245 (s), 1213 (w), 1154 (m), 1090 (m), 1015 (m), 954 (w), 843 (m), 809 (m), 751 (s), 689 (s) cm^{-1}; MS TOF ES+ (*m/z*): 329.2 $[M+H]^+$; HRMS (m/z): $[M+H]^+$ calcd. for $C_{20}H_{26}N_2Cl^+$, 329.1779; found, 329.1787;

*Synthesis of N-(((2S,4R)-1-neopentyl-4-phenylazetidin-2-yl)methyl)propan-2-amine **1g**.*

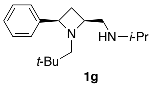
1g

Prepared using *General Procedure E* with (*R*)-*N*-neopentyl-1-phenylbut-3-en-1-amine (150 mg, 1.0 equiv., 0.69 mmol), iodine (525 mg, 3.0 equiv., 2.1 mmol), $NaHCO_3$ (289 mg, 5.0 equiv., 3.5 mmol), acetonitrile (10 mL) and isopropyl amine (203 mg, 5.0 equiv., 3.5 mmol). Pale brown powder (61.0 mg, 0.22 mmol, 32% yield over two steps). $[\alpha]_D^{21}$ +30.5 (*c* 1.00, CH_2Cl_2); TLC (5% MeOH/DCM, visualised with $KMnO_4$): R_f = 0.23; ^1H NMR (300 MHz, $CDCl_3$,): δ 7.46-7.22 (5H, m, Ar*H*), 3.94 (1H, t, *J* 8.1, C*H*CH_2CH), 3.68-3.60 (1H, m, NC*H*CH_2N), 3.57-3.45 (1H, m, NHC*H*$(CH_3)_2$), 3.26 (1H, dd, *J* 12.5 & 2.4, NCHC*H*H), 3.01 (1H, dd, *J* 12.5 & 7.7, NCHC*H*H), 2.74-2.68 (1H, m, CHCH*H*CH), 2.45 (2H, s, *t*-BuCH*H*N), 2.22-2.12 (1H, m, CHCH*H*CH), 1.55 (6H, d, *J* 6.5, Me), 0.63 (9H, s, *t*-Bu); ^{13}C NMR (75.5 MHz, $CDCl_3$): δ 143.2 (C, Ar), 128.4 (CH, Ar), 127.6 (CH, Ar), 127.4 (CH, Ar), 72.6 (CH, Ph*C*HCH_2), 70.1 (CH, N*C*HCH_2), 58.7 (CH_2, *t*Bu*C*H_2N), 51.8 (CH, *C*H$(CH_3)_2$), 47.9 (CH_2, CH*C*H_2NH), 31.5 (CH_2, CH*C*H_2CH), 29.7 (C, *t*-Bu), 28.4 (CH_3, *t*-Bu), 19.8 (CH_3, Me), 19.2 (CH_3, Me); IR (neat) ν_{max} = 2951 (s), 2867 (s), 1639 (m), 1601 (m), 1567 (m), 1460 (s), 1394 (s), 1363 (s), 1318 (m), 1252

(w), 1207 (w), 1157 (m), 1073 (m), 1026 (m), 917 (m), 758 (s), 732 (s), 700 (s) cm^{-1}; MS TOF ES+ (*m/z*): 275.3 $[M+H]^+$; HRMS (m/z): $[M+H]^+$ calcd. for $C_{18}H_{31}N_2^+$, 275.2483; found, 275.4529;

Synthesis of N*-(((2*S*,4*R*)-1-(3,5-bis(trifluoromethyl)benzyl)-4-phenylazetidin-2-yl)methyl)propan-2-amine* ***1h***.

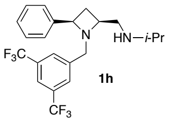

Prepared using *General Procedure E* with (*R*)-*N*-(3,5-bis(trifluoromethyl)benzyl)-1-phenylbut-3-en-1-amine (150 mg, 1.0 equiv., 0.4 mmol), iodine (306 mg, 3.0 equiv., 1.2 mmol), $NaHCO_3$ (169 mg, 5.0 equiv., 2.0 mmol), acetonitrile (20 mL) and isopropyl amine (0.172 mL, 5.00 equiv., 2.0 mmol). Purified using a *Combiflash Rf 200i* (silica 4 g column, 0-100% EtOAc/*n*-hexane) and triturated with Et_2O (2 mL). White powder (58 mg, 0.14 mmol, 34% yield over two steps). TLC (MeOH: EtOAc =2:98 v/v, visualised with $KMnO_4$): R_f = 0.33; $[\alpha]_D^{21}$ +9.6 (*c* 1.00, CH_2Cl_2); mp: 159-162 °C; ^{1}H NMR (400 MHz, $CDCl_3$): δ 7.63 (3H, dd, *J* 17.6 and 1.8, Ar*H*), 7.23-7.15 (5H, m, Ar*H*), 4.09-3.95 (3H, m, PhC*H*CH_2, NC*H*CH_2NH & PhCH*H*N), 3.78 (1H, d, *J* 13.5, PhC*H*HN), 3.54-3.44 (1H, m, NHC*H*$(CH_3)_2$), 3.09 (1H, dd, *J* 12.7 & 3.3, NCHC*H*H), 3.01 (1H, dt, *J* 12.7 & 7.8, NCHCH*H*), 2.87 (1H, dt, *J* 11.1 & 7.5, CHCH*H*CH), 2.22 (1H, dt, *J* 11.1 & 8.5, CHCH*H*CH), 1.50 (3H, d, *J* 5.0, Me), 1.48 (3H, d, *J* 5.0, Me); ^{13}C NMR (101 MHz, PENDANT, $CDCl_3$): δ 140.9 (C, Ar), 140.0 (C, Ar), 131.4 (C, q, $^3J_{C\text{-}F}$ = 33.9 Hz, Ar), 129.2 (CH, Ar), 128.4 (CH, Ar), 127.9 (CH, Ar), 126.8 (CH, Ar), 122.9 (C, q, $^1J_{C\text{-}F}$ = 212 Hz, CF_3), 121.2 (CH, q, $^2J_{C\text{-}F}$ = 3.3 Hz, Ar), 67.0 (CH, Ph*C*HCH_2), 60.4 (CH_2, *C*H_2N), 57.9 (CH, N*C*HCH_2), 51.8 (CH, *C*H$(CH_3)_2$), 47.8 (CH_2, CH*C*H_2NH), 33.8 (CH_2, CH*C*H_2CH), 19.0 (CH_3, Me), 18.7 (CH_3, Me); ^{19}F NMR (282 MHz, $CDCl_3$): δ 62.8 (s, CF_3); IR (neat) ν_{max} = 2959 (s), 2837 (s), 2801 (s), 1579 (w), 1459 (w), 1383 (m), 1349 (m), 1278 (s), 1173 (m), 1130 (s), 896 (w), 843 (w), 754 (m), 700 (s) cm^{-1}; MS TOF ES+ (*m/z*): 431.2 $[M+H]^+$; HRMS (m/z): $[M+H]^+$ calcd. for $C_{22}H_{25}N_2F_6^+$, 431.1916; found, 431.1918.

Synthesis of N*-(((2*S*,4*R*)-1-(4-(tert-butyl)benzyl)-4-phenylazetidin-2-yl)methyl) propan-2-amine* ***1i***.

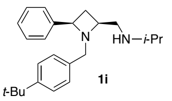

Prepared using *General Procedure E* with (*R*) *N*-(4-(*tert*-butyl)benzyl)-1-phenylbut-3-en-1-amine (150 mg, 1.0 equiv., 0.51 mmol), iodine (389 mg, 3.0 equiv., 1.5 mmol), $NaHCO_3$ (215 mg, 5.0 equiv., 2.6 mmol), acetonitrile (10 mL) and isopropyl amine (1 mL, 5.0 equiv., 2.6 mmol). Purified by flash column chromatography (silica gel 40-63 μm, 0-5% MeOH/EtOAc). Pale brown oil (68.1 mg, 0.19 mmol, 38% yield over two steps). $[\alpha]_D^{21}$ +87.9 (*c* 1.00, CH_2Cl_2); TLC (100% EtOAc, visualised with $KMnO_4$): R_f = 0.47; ^{1}H NMR (300 MHz, $CDCl_3$,): δ 7.48-7.29 (9H, m Ar*H*), 4.23 (1H, t, *J* 8.2, PhC*H*CH_2), 3.87-3.74 (2H, m, NC*H*CH_2NH & PhC*H*HN), 3.49 (1H, d, *J* 12.3, PhCH*H*N), 3.04 (1H, dd, *J* 12.5 & 3.2, CHC*H*HNH), 2.65-2.34 (4H, m, NHC*H*$(CH_3)_2$, NCHC*H*$_2$ & CHC*H*HNH), 1.29 (9H, s, *t*-Bu), 1.24 (3H, d, *J* 6.6, Me), 1.16 (3H, d, *J* 6.6, Me); ^{13}C NMR (75.5 MHz, $CDCl_3$): δ 151.8 (C, Ar), 140.8 (C, Ar), 135.1 (C, Ar), 129.3 (CH, Ar), 128.8 (CH, Ar), 128.1 (CH, Ar), 127.0 (CH, Ar), 126.3 (CH, Ar), 65.8 (CH, Ph*C*HCH_2), 59.9 (CH_2, Ph*C*H_2N), 58.3 (CH, N*C*HCH_2), 52.2 (CH, *C*H$(CH_3)_2$), 47.3 (CH_2, CH*C*H_2NH), 34.7 (C, *t*-Bu), 31.3 (CH_3, *t*-Bu), 30.0 (CH_2, CH*C*H_2CH), 19.9 (CH_3, Me), 17.9 (CH_3, Me); IR (neat) ν_{max} = 3020 (m), 2958 (s), 2871 (s), 2793 (s), 2708 (s), 1600 (w), 1570 (w), 1511 (w), 1492 (w), 1453 (w), 1393 (m), 1362 (m), 1307 (w), 1269 (m), 1245 (w), 1215 (w), 1152 (m), 1109 (m), 1017 (m), 979 (w),

927 (w), 858 (w), 841 (w), 812 (w), 782 (w), 755 (m), 734 (s), 699 (s) cm^{-1}; MS TOF EI+ (*m/z*): 351.4 [M+H]$^+$; HRMS (m/z): [M+H]$^+$ calcd. for $C_{24}H_{35}N_2$, 351.2795; found, 351.2798

Synthesis of N-*(((2*S*,4*R*)-1-(4-methoxybenzyl)-4-phenylazetidin-2-yl)methyl) propan-2-amine* ***1j****.*

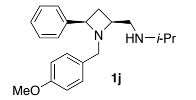

1j

Prepared using *General Procedure E* with (*R*)- *N*-(4-methoxybenzyl)-1-phenylbut-3-en-1-amine (150 mg, 1.0 equiv., 0.56 mmol), iodine (311 mg, 3.0 equiv., 1.7 mmol), $NaHCO_3$ (170 mg, 5.0 equiv., 2.0 mmol), acetonitrile (10 mL) and isopropyl amine (0.167 mL, 5.0 equiv., 2.0 mmol). Purified using a *Combiflash Rf 200i* (silica 4 g column, 0-100% EtOAc/*n*-hexane and 0-10% MeOH/CH_2Cl_2). Pale brown oil (78.3 mmol, 0.241 mmol, 43% yield over two steps). $[\alpha]_D^{21}$ +48.0 (*c* 1.00, CH_2Cl_2); TLC (10% MeOH/EtOAc, visualised with $KMnO_4$): R_f = 0.42; ^{1}H NMR (300 MHz, $CDCl_3$,): δ 7.44-7.29 (7H, m Ar*H*), 6.94 (2H, d, *J* 8.6, Ar*H*), 4.20 (1H, t, *J* 8.3, PhC*H*CH_2), 3.85-3.80 (5H, m, NC*H*CH_2NH, PhC*H*HN & OMe), 3.47 (1H, d, *J* 12.4, PhCH*H*N), 2.94 (1H, dd, *J* 12.5 & 4.6, CHC*H*HNH), 2.82-2.75 (1H, m, NHC*H*$(CH_3)_2$), 2.69-2.62 (1H, m, NCHC*H*H), 2.56 (1H, dd, *J* 12.5 & 4.3, CHC*H*HNH), 2.30 (1H, dt, *J* 11.3 & 8.6, NCHCH*H*), 1.27 (3H, d, *J* 6.5, Me), 1.21 (3H, d, *J* 6.5, Me); ^{13}C NMR (101 MHz, $CDCl_3$): δ 159.7 (C, (-), Ar), 141.2 (C, (-), Ar), 130.9 (CH, (+), Ar), 130.0 (C, (-), Ar), 128.7 (CH, (+), Ar), 128.0 (CH, (+), Ar), 126.8 (CH, (+), Ar), 114.8 (CH, (+), Ar), 65.4 (CH, (+), Ph*C*HCH_2), 59.8 (CH_2, (-), Ph*C*H_2N), 57.7 (CH, (+), N*C*HCH_2), 55.5 (CH_3, (+), OMe), 52.1 (CH, (+), *C*H$(CH_3)_2$), 48.5 (CH_2, (-), NCH*C*H_2NH), 30.3 (CH_2, (-), CH*C*H_2CH), 19.7 (CH_3, (+), Me), 18.2 (CH_3, (+), Me); IR (neat) ν_{max} = 2936 (m), 2833 (m), 2781 (m), 2705 (m), 1610 (s), 1584 (m), 1511 (s), 1454 (m), 1393 (m), 1354 (w), 1302 (m), 1246 (s), 1175 (m), 1155 (m), 1109 (w), 1030 (s), 852 (m), 822 (m), 788 (w), 749 (s), 734 (s), 699 (s) cm^{-1}; MS TOF ES+ (*m/z*): 325.2 [M+H]$^+$; HRMS (m/z): [M+H]$^+$ calcd. for $C_{21}H_{29}N_2O^+$, 325.2274; found, 325.2282.

Synthesis of N-*(((2*S*,4*R*)-1-Benzyl-4-(4-chlorophenyl)azetidin-2-yl)methyl) propan-1-amine* ***1k****.*

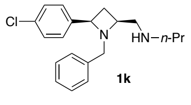

1k

Prepared using *General Procedure E* with (*R*)-*N*-benzyl-1-(4-chlorophenyl)but-3-en-1-amine (150 mg, 1.0 equiv., 0.6 mmol), iodine (420 mg, 3.0 equiv., 1.7 mmol), $NaHCO_3$ (232 mg, 5.0 equiv., 2.8 mmol), acetonitrile (15 mL) and *n*-propyl amine (163 mg, 5.0 equiv., 2.8 mmol). Purified using a *Combiflash Rf 200i* (silica 4 g column, 0-100% EtOAc/*n*-hexane) and washed with Et_2O (2 mL). Pale brown powder (82.0 mg, 45% yield over two steps). mp: 161-163 ºC; $[\alpha]_D^{21}$ +13.2 (*c* 1.00, CH_2Cl_2); TLC (100% EtOAc, visualised with $KMnO_4$): R_f = 0.24; ^{1}H NMR (400 MHz, $CDCl_3$,): δ 7.42-7.34 (9H, m, Ar*H*), 4.17 (1H, t, *J* 8.2, PhC*H*CH_2), 3.87 (1H, d, *J* 12.2, PhC*H*HN), 3.72-3.66 (1H, m, NC*H*CH_2NH), 3.49 (1H, d, *J* 12.2, PhCH*H*N), 3.04 (1H, dd, *J* 12.8 & 2.6, CHC*H*HNH), 2.64-2.56 (2H, m, NCHC*H*H & *n*-propyl), 2.47-2.42 (1H, m, NCHCH*H*), 2.26 (1H, dd, *J* 12.8 & 5.1, CHC*H*HNH), 2.19-2.12 (1H, m, *n*-propyl), 1.72-1.55 (2H, m, *n*-propyl), 0.86 (3H, t, *J* 7.4, *n*-propyl); ^{13}C NMR (101 MHz, PENDANT, $CDCl_3$): δ 139.3 (C, (-), Ar), 137.6 (C, (-), Ar), 133.7 (C, (-), Ar), 129.5 (CH, (+), Ar), 129.4 (CH, (+), Ar), 128.9 (CH, (+), Ar), 128.4_2 (CH, (+), Ar), 128.3_8 (CH, (+), Ar), 65.3 (CH, (+), Ph*C*HCH_2), 60.5 (CH_2, (-), Ph*C*H_2N), 58.0 (CH, (+), N*C*HCH_2), 49.9 (CH_2, (-),*n*-propyl), 49.5 (CH_2, (-), CH*C*H_2N), 30.5 (CH_2, (-), CH*C*H_2CH), 19.4 (CH_2, (-),*n*-propyl), 11.0 (CH_3, (+), *n*-propyl); IR (neat) ν_{max} = 2953 (s), 2884 (m), 2851 (m), 2801

(s), 1573 (m), 1492 (s), 1455 (m), 1440 (m), 1407 (m), 1363 (m), 1295 (w), 1265 (s), 1208 (m), 1157 (m), 1089 (s), 1043 (w), 1015 (s), 968 (w), 922 (w), 821 (s), 779 (w), 734 (s), 701 (s) cm^{-1}; MS TOF ES+ (*m/z*): 329.2 $[M+H]^+$; HRMS (m/z): $[M+H]^+$ calcd. for $C_{20}H_{26}N_2{}^{35}Cl^+$, 329.1779$^+$; found, 329.1786.

Synthesis of N*-benzyl-1-((*2R, 4S*)-1-benzyl-4-(4-chlorophenyl)azetidin-2-yl)methanamine* ***1l*****.**

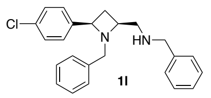
1l

Prepared using *General Procedure E* with (*R*)-*N*-benzyl-1-(4-chlorophenyl)but-3-en-1-amine (1.50 g, 1.0 equiv., 5.5 mmol), iodine (4.20 g, 3.0 equiv., 16.6 mmol), $NaHCO_3$ (2.32 g, 5.0 equiv., 27.6 mmol), acetonitrile (15 mL) and benzylamine (2.96 mL, 5.0 equiv., 27.6 mmol). Purified using a *Combiflash Rf 200i* (silica 12 g column, 0-50% EtOAc/*n*-hexane and 0-5% MeOH/DCM). Pale yellow oil (782 mg, 2.1 mmol, 38% yield over two steps). $[\alpha]_D^{21}$ +47.1 (*c* 1.00, CH_2Cl_2); TLC (30% EtOAc/Hexane, visualised with $KMnO_4$): R_f = 0.26; ^{1}H NMR (400 MHz, $CDCl_3$,): δ 7.34-7.18 (14H, m, Ar*H*), 3.94 (1H, t, *J* 8.1, PhC*H*CH_2), 3.75 (1H, d, *J* 12.8, PhC*H*HN), 3.58 (2H, d, *J* 2.1, PhC*H*$_2$N), 3.51 (1H, d, *J* 12.8, PhCH*H*N), 3.32-3.26 (1H, m, NC*H*CH_2NH), 2.50 (1H, dd, *J* 12.1 & 4.4, CHC*H*HNH), 2.43-2.35 (2H, m, NCHCH*H* & CHC*H*HNH), 1.92 (1H, dt, *J* 10. & 8.6, CHC*H*HNH), 1.43 (1H, br s, N*H*); ^{13}C NMR (101 MHz, $CDCl_3$): δ 142.1 (C, Ar), 140.6 (C, Ar), 138.6 (C, Ar), 132.6 (C, Ar), 129.1 (CH, Ar), 128.2 (CH, Ar), 128.2$_8$ (CH, Ar), 128.2$_3$ (CH, Ar), 128.1 (CH, Ar), 127.9 (CH, Ar), 127.2 (CH, Ar), 126.7 (CH, Ar), 64.6 (CH, Ph*C*HCH_2), 62.6 (CH, (+), N*C*HCH_2), 61.5 (CH_2, Ph*C*H$_2$N), 54.1 (CH_2, Ph*C*H$_2$N), 53.6 (CH_2, NCH*C*H$_2$NH), 31.1 (CH_2, CH*C*H$_2$CH); IR (neat) ν_{max} = 3315 (w), 3083 (w), 3062 (w), 3027 (w), 3000 (w), 2816 (m), 1598 (w), 1490 (s), 1453 (s), 1411 (w), 1382 (w), 1354 (w), 1292 (w), 1236 (w), 1206 (w), 1155 (w), 1087 (s), 1057 (w), 1028 (w), 1013 (s), 939 (w), 909 (w), 822 (s), 792 (w), 734 (s), 696 (s) cm^{-1}; MS TOF ES+ (*m/z*): 377.2 $[M+H]^+$; HRMS (m/z): $[M+H]^+$ calcd. for $C_{24}H_{26}N_2{}^{35}Cl^+$, 377.1779$^+$; found, 377.1776

*Synthesis of 1-(((2*S*,4*R*)-1-Benzyl-4-(4-chlorophenyl)azetidin-2-yl)methyl) pyrrolidine* ***1m*****.**

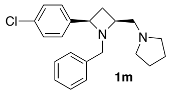
1m

Prepared using *General Procedure E* with (*R*)-*N*-benzyl-1-(4-chlorophenyl)but-3-en-1-amine (150 mg, 1.00 equiv., 0.552 mmol), iodine (420 mg, 3.0 equiv., 1.7 mmol), $NaHCO_3$ (232 mg, 5.0 equiv., 2.8 mmol), acetonitrile (10 mL) and pyrrolidine (169 mg, 5.0 equiv., 2.8 mmol). Pale brown powder (67 mg, 0.20 mmol, 36% yield over two steps). $[\alpha]_D^{21}$ +52.7 (*c* 1.00, CH_2Cl_2); mp: 157-159 °C; TLC (5% MeOH/DCM, visualised with $KMnO_4$): R_f = 0.24; ^{1}H NMR (300 MHz, $CDCl_3$,): δ 7.36-7.24 (9H, m, Ar*H*), 4.10 (1H, t, *J* 8.1, C*H*CH_2CH), 3.85-3.77 (2H, m, NC*H*CH_2N & PhC*H*HN), 3.57 (1H, d, *J* 12.4, PhCH*H*N), 3.21 (4H, br s, pyrrolidine), 2.91-2.80 (2H, m, CHC*H*HN & CHC*H*HCH), 2.62 (1H, dd, *J* 12.8 & 2.8, CHCH*H*N), 2.07 (4H, br s, pyrrolidine), 1.96 (1H, dt, *J* 10.7 & 8.4, CHCH*H*CH); ^{13}C NMR (75.5 MHz, PENDANT, $CDCl_3$): δ 140.5 (C, (-), Ar), 137.1 (C, (-), Ar), 133.2 (C, (-), Ar), 129.7 (CH, (+), Ar), 128.6$_4$ (CH, (+), Ar), 128.5$_6$ (CH, (+), Ar), 127.9 (CH, (+), Ar), 127.8 (CH, (+), Ar), 65.1 (CH, (+), Ph*C*HCH_2), 60.9 (CH_2, (-), Ph*C*H$_2$N), 59.7 (CH, (-), N*C*HCH_2), 57.2 (CH_2, (+), CH*C*H$_2$N), 54.4 (CH_2, (-), pyrrolidine), 35.4 (CH_2, (-), CH*C*H$_2$CH), 23.2 (CH_2, (-), pyrrolidine); IR (neat) ν_{max} = 3027 (m), 2883 (m), 2824 (m), 2676 (m), 2605 (w), 2475 (w), 1598 (w), 1490 (s), 1453 (s), 1396 (m), 1354 (m), 1272 (w), 1213 (w),

1157 (m), 1087 (s), 1013 (s), 939 (w), 912 (w), 821 (s), 780 (w), 733 (s), 703 (s) cm^{-1}; MS TOF ES+ (*m/z*): 341.18 $[M+H]^+$; HRMS (m/z): $[M+H]^+$ calcd. for $C_{21}H_{26}N_2{}^{35}Cl^+$, 341.1779; found, 341.1786;

*Synthesis of 1-((*2R, 4S*)-1-benzyl-4-(4-chlorophenyl)azetidin-2-yl)-*N*-(4-chlorobenzyl)methanamine* ***1n***.

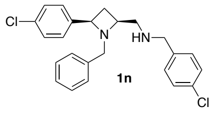
1n

Prepared using *General Procedure E* with (*R*)-*N*-benzyl-1-(4-chlorophenyl)but-3-en-1-amine (250 mg, 1.0 equiv., 0.92 mmol), iodine (702 mg, 3.0 equiv., 2.8 mmol), $NaHCO_3$ (387 mg, 5.0 equiv., 4.6 mmol), acetonitrile (15 mL) and 4-chlorobenzylamine (653 mg, 5.0 equiv., 4.6 mmol). Purified using a *Combiflash Rf 200i* (silica 4 g column, 0-100% EtOAc/*n*-hexane and 0-5% MeOH/DCM). Pale yellow oil (118 mg, 0.287 mmol, 31% yield over two steps). $[\alpha]_D^{21}$ +59.1 (*c* 1.00, CH_2Cl_2); TLC (50% EtOAc/*n*-hexane, visualised with $KMnO_4$): R_f = 0.50; ^1H NMR (400 MHz, $CDCl_3$,): δ 7.39-7.09 (13H, m, Ar*H*), 3.96 (1H, t, *J* 8.1 , PhC*H*CH_2), 3.76 (1H, d, *J* 12.8, PhC*H*HN), 3.54-3.47 (3H, m, PhC*H*HN & CH_2NHC*H_2*), 3.31-3.24 (1H, m, NC*H*CH_2NH), 2.45 (1H, dd, *J* 12.1 & 4.4, CHC*H*HNH), 2.41-2.36 (1H, m, NCHCH*H*), 2.32 (1H, dd, *J* 12.1 & 4.5, CHC*H*HNH), 1.97-1.90 (1H, m, CHC*H*HCH), 1.55 (1H, br s, N*H*); ^{13}C NMR (75.5 MHz, $CDCl_3$): δ 142.0 (C, Ar), 139.0 (C, Ar), 138.6 (C, Ar), 132.7 (C, Ar), 132.3 (C, Ar), 129.3 (CH, Ar), 129.1 (CH, Ar), 128.3 (CH, Ar), 128.3 (CH, Ar), 128.2 (CH, Ar), 128.2 (CH, Ar), 127.2 (CH, Ar), 64.6 (CH, Ph*C*HCH_2), 62.6 (CH, N*C*HCH_2), 61.5 (CH_2, PhCH_2N), 53.4 (CH_2, Ph*C*H_2N), 53.3 (CH_2, NCH*C*H_2NH), 31.0 (CH_2, CH*C*H_2CH); IR (neat) ν_{max} = 3086 (w), 3065 (w), 3029 (w), 2831 (m), 1597 (w), 1490 (s), 1454 (m), 1407 (m), 1381 (w), 1292 (w), 1234 (w), 1165 (w), 1088 (s), 1060 (m), 1014 (s), 940 (w), 821 (s), 742 (m), 702 (s) cm^{-1}; MS TOF ES+ (*m/z*): 411.14 $[M+H]^+$; HRMS (m/z): $[M+H]^+$ calcd. for $C_{24}H_{25}N_2Cl_2^+$, 411.1389; found, 411.1396;

*Synthesis of 1-((*2R, 4S*)-1-benzyl-4-(4-chlorophenyl)azetidin-2-yl)-*N*-(4-methoxybenzyl)methanamine* ***1o***.

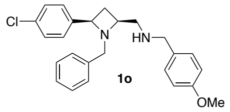
1o

Prepared using *General Procedure E* with (*R*)-*N*-benzyl-1-(4-chlorophenyl)but-3-en-1-amine (250 mg, 1.0 equiv., 0.92 mmol), iodine (702 mg, 3.0 equiv., 2.8 mmol), $NaHCO_3$ (387 mg, 5.0 equiv., 4.6 mmol), acetonitrile (15 mL) and 4-methoxybenzylamine (632 mg, 5.0 equiv., 4.6 mmol). Purified using a *Combiflash Rf 200i* (silica 4 g column, 0-100% EtOAc/*n*-hexane and 0-5% MeOH/DCM). Pale yellow oil (156 mg, 0.38 mmol, 42% yield over two steps). $[\alpha]_D^{21}$ +81.6 (*c* 1.00, CH_2Cl_2); TLC (50% EtOAc/hexane, visualised with $KMnO_4$): R_f = 0.26; ^1H NMR (300 MHz, $CDCl_3$,): δ 7.36-7.09 (11H, m, Ar*H*), 6.83 (2H, d, *J* 8.7, Ar*H*), 3.94 (1H, t, *J* 8.2, PhC*H*CH_2), 3.80 (3H, s, OMe), 3.75 (1H, d, *J* 12.8, PhC*H*HN), 3.53-3.48 (3H, m, PhC*H_2*N & CH_2NHC*H_2*), 3.33-3.24 (1H, m, NC*H*CH_2NH), 2.49 (1H, dd, *J* 12.1 & 4.5, CHC*H*HNH), 2.44-2.33 (2H, m, CHC*H*HNH & NCHCH*H*), 1.90 (1H, dt, *J* 10.2 & 8.8, CHC*H*HCH), 1.43 (1H, br s, N*H*); ^{13}C NMR (75.5 MHz, $CDCl_3$): δ 158.5 (C, Ar), 142.1 (C, Ar), 138.6 (C, Ar), 132.6 (C, Ar), 129.1 (CH, Ar), 128.7 (CH, Ar), 128.3 (C, Ar), 128.3 (CH, Ar), 128.2 (CH, Ar), 128.2 (CH, Ar), 127.2 (CH, Ar), 113.6 (CH, Ar), 64.6 (CH, Ph*C*HCH_2), 62.6 (CH, (+), N*C*HCH_2), 61.5 (CH_2, Ph*C*H_2N), 55.3 (CH_2, Ph*C*H_2N), 53.5 (CH_2, NCH*C*H_2NH), 31.2 (CH_2, CH*C*H_2CH); IR (neat) ν_{max} = 3089 (w), 3065 (w), 3029 (w), 2996 (w), 2830 (m), 1611 (m), 1585 (m), 1511 (s), 1490 (s), 1454 (s), 1384 (w), 1357 (w), 1300 (m), 1245 (s), 1173 (m), 1088 (m), 1035 (s), 1016 (s), 943 (w), 821 (s), 742 (m), 701 (s)

cm^{-1}; MS TOF ES+ (*m/z*): 407.19 [M+H]$^+$; HRMS (m/z): [M+H]$^+$ calcd. for $C_{25}H_{28}N_2OCl^+$, 407.1885; found, 407.1893.

*Synthesis of ((2*S*,4*R*)-1-benzyl-4-phenylazetidin-2-yl)methanamine,* ***1s****.*

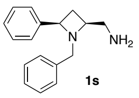

1s

Caesium carbonate (0.87 mmol, 283 mg) and iodine (0.29 mmol, 74 mg), were added to *N*-benzyl-1-((2*S*,4*R*)-1-benzyl-4-phenylazetidin-2-yl)methanamine **1r** (0.29 mmol, 100 mg),[13] in CH_3CN (15 mL) and stirred at room temperature until starting azetidine was completely consumed (TLC). Subsequent treatment with HCl (3 M, aqueous) at 60 °C (1 h) followed by neutralisation with NaOH (2 M, aqueous), extraction into dichloromethane, drying over magnesium sulfate, filtration and evaporation to dryness *in vacuo* afforded a yellow oil, 73 mg, >99%. $[\alpha]_D^{20}$ = +107.8 (*c* 1.4, $CHCl_3$). All other characterisation matched that reported for the racemate.[14] IR 3061, 3027, 2019, 2851, 1601, 1493, 1454, 1353, 1303, 1209, 1157; ^{1}H NMR (δ; 300 MHz, $CDCl_3$); 1.22 (2H, br, N*H*$_2$), 1.76 (1H, $_{apt}$q, $_{obs}J$ = 8.7, PhCHCH*H*), 2.26-2.35 (3H, m, PhCHC*H*H and NCHC*H*$_2$NH$_2$), 3.07-3.15 (1H, m, NC*H*CH$_2$NH$_2$), 3.59 (2H, ABq, J_{AB} = 12.9, NC*H*$_2$Ph), 3.95 (1H, $_{apt}$t, $_{obs}J$ = 8.1, PhC*H*CH$_2$), 7.12-7.40 (10H, m, Ph*H*); ^{13}C NMR (δ; 100 MHz, $CDCl_3$), 29.79 (CH$_2$), 45.60 (CH$_2$), 61.45 (CH$_2$), 64.59 (CH), 64.89 (CH), 126.70 (CH), 127.05 (CH), 127.08 (CH), 128.17 (CH), 128.21 (CH), 129.12 (CH), 138.97 (C), 143.56 (C). High-resolution MS calcd. for [M+H]$^+$ formula $C_{17}H_{21}N_2^+$: 253.1699; found: 253.1717.

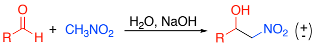

5a-j **6a-j (*rac*)**

Scheme S 5*. Conversion of aldehydes* ***5a-j*** *into the corresponding racemic nitromethane Henry reaction adducts* ***6a-j****.*

Synthesis of (rac)*-1-(4-nitrophenyl)-2-nitroethanol* **(rac)*-6a*****.**

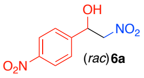

Prepared using *General Procedure F* with 4-nitrobenzaldehyde (0.30 g, 2.0 mmol), nitromethane (0.11 mL, 2.0 mmol), sodium hydroxide aqueous solution (10 M, 0.2 mL, 2.0 mmol) and acetic acid (0.11 mL, 2.0 mmol) and ethanol (3 mL). Purified using a *Combiflash Rf 200i* (silica 4 g column, 0-30% EtOAc/*n*-hexane). The characterisation (^{1}H and ^{13}C NMR spectroscopy, IR and mass spectra) reported is consistent with that given the literature (racemic product).[15] Pale yellow crystalline solid (0.33 g, 1.5 mmol, 77% yield). mp: 92-94 °C; TLC (EtOAc:*n*-hexane = 20:80 v/v, visualised by UV 254 nm): R_f = 0.34; ^{1}H NMR (300 MHz, $CDCl_3$,): δ 8.30 (2H, d, *J* 8.8 Hz, Ar*H*), 7.65 (2H, d, *J* 8.5 Hz, Ar*H*), 5.66-5.61 (1H, m, C*H*OH), 4.63-4.60 (2H, m, C*H*$_2$NO$_2$), 3.16 (1H, br s, O*H*); ^{13}C NMR (101 MHz, PENDANT, $CDCl_3$): δ 148.2 (C, (-), Ar), 144.9 (C, (-), Ar), 126.9 (CH, (+), Ar), 124.2 (CH, (+), Ar), 80.6 (CH$_2$, (-), *C*H$_2$NO$_2$), 70.0 (CH, (+), *C*HOH); IR (solid) ν_{max} = 3520 (broad, m), 3115 (w), 3083 (w), 2921 (w) 1553 (s), 1518 (s), 1348 (s), 1083 (m), 857 (m) cm^{-1} [lit:[15] (racemic, film):3458, 3113, 3082, 2984, 2931, 1527, 1348, 1085, 857 cm^{-1}]; MS Scan AP+ (*m/z*): 213.3 [M+H]$^+$;

Synthesis of (S)-1-(4-nitrophenyl)-2-nitroethanol ***(S)-6a****.*

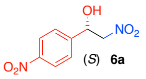
(*S*) **6a**

Prepared using *General Procedure G* with 4- nitrobenzaldehyde (75.6 mg, 1.0 equiv., 0.5 mmol), nitromethane (0.27 mL, 10 equiv., 5.0 mmol), $Cu(OAc)_2{\cdot}H_2O$ (4.99 mg, 5 mol%, 0.025 mmol), *N*-benzyl-1-((*2R*, *4S*)-1-benzyl-4-(4-chlorophenyl)azetidin-2-yl)methanamine (9.42 mg, 5 mol%, 0.025 mmol) and ethanol (1 mL). Purified using a *Combiflash Rf 200i* (silica 4 g column, 0-30% EtOAc/*n*-hexane). The characterisation reported is consistent with that given the literature.[16] Pale yellow solid (85.7 mg, 0.429 mmol, 86% yield); 91% *e.e.* was determined by HPLC using a chiral stationary phase (Eurocel 01, 5 µm, OD, 6.5 % IPA/*n*-hexane, 0.5 mL/min, UV 254 nm), minor enantiomer (*R*) t_r = 37.0 min, major enantiomer (*S*) t_r = 44.9 min); $[\alpha]_D^{21}$ +19.9 (*c* 1.00, CH_2Cl_2); mp: 87-89 °C; TLC (EtOAc:*n*-hexane= 20:80 v/v, visualised by UV 254 nm): R_f = 0.20; ^{1}H NMR (300 MHz, $CDCl_3$,): δ 8.27 (2H, d, *J* 8.8 Hz, Ar*H*), 7.63 (2H, d, *J* 9.0 Hz, Ar*H*), 5.62 (1H, dd, *J* 7.8, 4.5 Hz, C*H*OH), 4.76-4.52 (2H, m, C*H*$_2$NO$_2$), 3.19 (1H, br s, O*H*); ^{13}C NMR (101 MHz, PENDANT, $CDCl_3$): δ 148.2 (C, (-), Ar), 144.9 (C, (-), Ar), 127.0 (CH, (+), Ar), 124.2 (CH, (+), Ar), 80.6 (CH$_2$, (-), *C*H$_2$NO$_2$), 70.0 (CH, (+), *C*HOH); IR (solid) ν_{max} = 3484 (broad, m), 3116 (w), 3083 (w), 2913 (w) 1546 (s), 1509 (s), 1347 (s), 1076 (s), 860 (s) cm^{-1}; MS TOF AP+ (*m/z*): 213.1 [M+H]$^+$. The absolution configuration was assigned as (*S*) by comparison of the optical rotation with the following literature values: [lit[16b] $[\alpha]_D^{21}$-31.6 (*c* 1.05, CH_2Cl_2, 78% *e.e.*, (*R*)-isomer)], [lit[16a] $[\alpha]_D^{25}$+36.1 (*c* 0.98, CH_2Cl_2, 95% *e.e.*, (*S*)-isomer)].

*Synthesis of (*rac*)-1-phenyl-2-nitroethanol* **(rac)*-6b*****.*

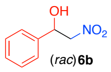
(*rac*)**6b**

Prepared using *General Procedure F* with benzaldehyde (0.300 g, 2.83 mmol), nitromethane (0.15 mL, 2.8 mmol), sodium hydroxide aqueous solution (10 M, 0.28 mL, 2.8 mmol), acetic acid (0.16 mL, 2.8 mmol) and ethanol (3 mL). Purified by flash column chromatography (silica gel 40-63 µm, 15-20 % EtOAc/*n*-hexane) .The characterisation (^{1}H and ^{13}C NMR spectroscopy, IR and mass spectra) reported is consistent with that given the literature (racemic product).[15] Colourless oil (0.366 g, 2.2 mmol, 77% yield). TLC (EtOAc:*n*-hexane = 20:80 v/v, visualised by UV 254 nm): R_f = 0.47; ^{1}H NMR (300 MHz, $CDCl_3$,): δ 7.41-7.34 (5H, m, Ar*H*), 5.46 (1H, dd, *J* 9.4 & 3.2, C*H*OH), 4.61 (1H, dd, *J* 13.3 & 9.4, C*H*HNO$_2$), 4.51 (1H, dd, *J* 13.3 & 3.2, CH*H*NO$_2$), 2.88 (1H, br s, O*H*); ^{13}C NMR (101 MHz, PENDANT, $CDCl_3$): δ 138.1 (C, (-), Ar), 129.1 (CH, (+), Ar), 129.0 (CH, (+), Ar), 126.0 (CH, (+), Ar), 81.2 (CH$_2$, (-), *C*H$_2$NO$_2$), 71.0 (CH, (+), *C*HCH$_2$); IR (neat) ν_{max} = 3432 (broad, m), 3065 (w), 3033 (w), 2921 (w), 1548 (s) 1495 (m), 1453 (m), 1417 (m), 1377 (s), 1288 (m), 1192 (m), 1065 (m), 894 (m), 764 (m), 698 (s), 522 (m) cm^{-1} [lit:[15] (racemic, film): 3442, 3065, 3033, 2922, 1556, 1495, 1454, 1419, 1378 cm^{-1}]; MS TOF EI+ (*m/z*): 167.07 [M]$^+$.

Synthesis of (S)-1-phenyl-2-nitroethanol ***(S)-6b****.*

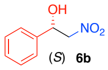
(*S*) **6b**

Prepared using *General Procedure G* with benzaldehyde (53.1 mg, 1.0 equiv., 0.50 mmol), nitromethane (0.27 mL, 10.0 equiv., 5.0 mmol), $Cu(OAc)_2{\cdot}H_2O$ (4.99 mg, 5 mol%, 0.025 mmol), *N*-benzyl-1-((*2R*, *4S*)-1-benzyl-4-(4-chlorophenyl)azetidin-2-yl)methanamine (9.42 mg, 5 mol%, 0.025 mmol) and ethanol (1 mL). Purified

using a *Combiflash Rf 200i* (silica 4 g column, 0-30% EtOAc/*n*-hexane). The characterisation reported is consistent with that given the literature.[16a, 17] Colourless oil (68.0 mg, 0.407 mmol, 81% yield); 95% *e.e.* was determined by HPLC using a chiral stationary phase (Eurocel 01, 5 μm, OD, 6.0 % IPA/*n*-hexane, 0.5 mL/min, UV 230 nm), minor enantiomer (*R*) t_r = 20.9 min, major enantiomer (*S*) t_r = 25.0 min); $[\alpha]_D^{21}$ +26.8 (*c* 1.00, CH_2Cl_2); TLC (EtOAc:*n*-hexane= 20:80 v/v, visualised with $KMnO_4$): R_f = 0.31; ^{1}H NMR (300 MHz, $CDCl_3$,): δ 7.42-7.35 (5H, m, Ar*H*), 5.47 (1H, dd, *J* 9.5 & 3.0, C*H*OH), 4.62 (1H, dd, *J* 13.4 & 9.6, C*H*HNO$_2$), 4.52 (1H, dd, *J* 13. & 3.1, CH*H*NO$_2$), 2.79 (1H, br s, O*H*); ^{13}C NMR (101 MHz, PENDANT, $CDCl_3$): δ 138.1 (C, (-), Ar), 129.1 (CH, (+), Ar), 129.0 (CH, (+), Ar), 125.9 (CH, (+), Ar), 81.2 (CH_2, (-), *C*H$_2$NO$_2$), 71.0 (CH, (+), *C*HCH$_2$); IR (neat) ν_{max} = 3431 (broad, m), 3068 (w), 3033 (w), 2921 (w), 1547 (s) 1495 (m), 1454 (m), 1418 (m), 1377 (s), 1288 (m), 1193 (m), 1065 (m), 894 (m), 763 (m), 698 (s) cm^{-1}; MS TOF AP- (*m/z*): 166.0 [M-H]$^-$. The absolution configuration was assigned as (*S*) by comparison of the optical rotation with the following literature values: [lit:[17] $[\alpha]_D^{20}$ -42.6 (*c* 1.0, CH_2Cl_2, 94% *e.e.*, (*R*)-isomer)], [lit:[16a] $[\alpha]_D^{25}$ +40.2 (*c* 0.96, CH_2Cl_2), 98% *e.e.*, (*S*)-isomer].

Synthesis of (rac)*-1-(4-methoxyphenyl)-2-nitroethanol* **(rac)*-6c*.**

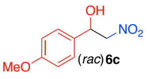

Prepared using *General Procedure F* with 4-methoxybenzaldehyde (0.300 g, 2.2 mmol), nitromethane (0.12 mL, 2.2 mmol), sodium hydroxide aqueous solution (10 M, 0.22 mL, 2.2 mmol) and acetic acid (0.13 mL, 2.2 mmol) and ethanol (3 mL). Purified using a *Combiflash Rf 200i* (silica 4 g column, 0-30% EtOAc/*n*-hexane). The characterisation (^{1}H and ^{13}C NMR spectroscopy, IR and mass spectra) reported is consistent with that given the literature (racemic product).[15] Colourless oil (0.334 g, 1.69 mmol, 77% yield); TLC (EtOAc:*n*-hexane= 20:80 v/v, visualised by UV 254 nm): R_f = 0.19; ^{1}H NMR (300 MHz, $CDCl_3$,): δ 7.33 (2H, d, *J* 8.7 Hz, Ar*H*), 6.94 (2H, d, *J* 8.6, Ar*H*), 5.42 (1H, dd, *J* 9.2 & 2.2, C*H*OH), 4.62 (1H, dd, *J* 13.1 & 9.6, CHC*H*HNO$_2$), 4.49 (1H, dd, *J* 13.2 & 3.2, CHCH*H*NO$_2$), 3.83 (3H, s, OMe), 2.85 (1H, br s, O*H*); ^{13}C NMR (101 MHz, PENDANT, $CDCl_3$): δ 160.0 (C, (-), Ar), 130.2 (C, (-), Ar), 127.3 (CH, (+), Ar), 114.4 (CH, (+), Ar), 81.2 (CH_2, (-), CH*C*H$_2$NO$_2$), 70.7 (CH, (+), Ph*C*HCH$_2$), 55.4 (CH_3, (+), OMe); IR (neat) ν_{max} = 3457 (broad, m), 2969 (w), 2926 (w), 2840 (w), 1709 (w), 1549 (s), 1377 (m), 1245 (s), 1027 (s) cm^{-1} [lit:[15] (racemic, film):3452, 3005, 2960, 2839, 2036, 1895, 1553, 1379, 1250, 1030 cm^{-1}]; MS Scan AP+ (*m/z*): 197.3 [M]$^+$.

*Synthesis of (*S*)-1-(4-methoxyphenyl)-2-nitroethanol* **(*S*)-6c**.

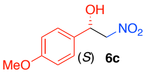

Prepared using *General Procedure G* with 4-methoxybenzaldehyde (68.1 mg, 1.0 equiv., 0.50 mmol), nitromethane (0.27 mL, 10 equiv., 5.0 mmol), $Cu(OAc)_2{\cdot}H_2O$ (4.99 mg, 5 mol%, 0.025 mmol), *N*-benzyl-1-((2*R*, 4*S*)-1-benzyl-4-(4-chlorophenyl)azetidin-2-yl)methanamine (9.42 mg, 5 mol%, 0.025 mmol) and ethanol (1 mL). Purified using a *Combiflash Rf 200i* (silica 4 g column, 0-30% EtOAc/*n*-hexane). The characterisation reported is consistent with that given the literature.[16a, 17] Colourless oil (73.0 mg, 0.37 mmol, 74% yield); 93% *e.e.* was determined by HPLC with a chiral stationary phase (Eurocel 01, 5 μm, OD, 6.0% IPA/*n*-hexane, 0.5 mL/min, UV 220 nm), minor enantiomer (*R*) t_r = 26.1 min, major enantiomer (*S*) t_r = 30.7 min); $[\alpha]_D^{21}$ +24.5 (*c* 1.00, CH_2Cl_2); TLC

(EtOAc:*n*-hexane= 20:80 v/v, visualised with $KMnO_4$): R_f = 0.21; ^{1}H NMR (400 MHz, $CDCl_3$,): δ 7.32 (2H, d, *J* 8.4, Ar*H*), 6.92 (2H, d, *J* 8.8, Ar*H*), 5.41 (1H, dd, *J* 9.6 & 3.1, C*H*OH), 4.60 (1H, dd, *J* 13.3 & 9.4, CHC*H*HNO_2), 4.48 (1H, dd, *J* 13.3 & 3.1, CHCH*H*NO_2), 2.77 (1H, br s, O*H*); ^{13}C NMR (101 MHz, PENDANT, $CDCl_3$): δ 160.1 (C, (-), Ar), 130.2 (C, (-), Ar), 127.3 (CH, (+), Ar), 114.4 (CH, (+), Ar), 81.3 (CH_2, (-), CH*C*H_2NO_2), 70.7 (CH, (+), *C*HCH_2), 55.4 (CH_3, (+), OMe); IR (neat) ν_{max} 3452 (broad, m), 2969 (w), 2925 (w), 2840 (w), 1677 (w), 1549 (s), 1377 (m), 1245 (s), 1026 (s) cm^{-1}; MS TOF AP- (*m/z*): 196.0 $[M-H]^-$. The absolution configuration was assigned as (*S*) by comparison of the optical rotation with the following literature values: [lit[17] $[\alpha]_D^{20}$-31.9 (*c* 1.00, CH_2Cl_2, 94% *e.e.*, (*R*)-isomer)], [lit:[16a] $[\alpha]_D^{25}$+28.4 (*c* 1.76, CH_2Cl_2), 95% *e.e.*, (*S*)-isomer].

*Synthesis of (*rac*)-1-(4-chlorophenyl)-2-nitroethanol* **(rac)-6d**.

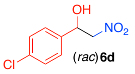

Prepared using *General Procedure F* with 4-chlorobenzaldehyde (0.30 g, 2.1 mmol), nitromethane (0.12 mL, 2.1 mmol), sodium hydroxide aqueous solution (10 M, 0.21 mL, 2.1 mmol) and acetic acid (0.13 mL, 2.13 mmol) and ethanol (3 mL). Purified using a *Combiflash Rf 200i* (silica 4 g column, 0-30% EtOAc/*n*-hexane). The characterisation reported is consistent with that given the literature (*S* isomer).[18] Colourless oil (0.261 g, 1.29 mmol, 61% yield). TLC (EtOAc:*n*-hexane = 20:80 v/v, visualised by UV 254 nm): R_f = 0.35; ^{1}H NMR (300 MHz, $CDCl_3$,): δ 7.40-7.33 (4H, m, Ar*H*), 5.45 (1H, dt, *J* 9.1 & 5.9, C*H*OH), 4.58 (1H, dd, *J* 13.4 & 9.2, C*H*HNO_2), 4.48 (1H, dd, *J* 13.4 & 3.4, CH*H*NO_2), 2.94 (1H, br, s, O*H*); ^{13}C NMR (101 MHz, PENDANT, $CDCl_3$): δ 136.5(C, (-), Ar), 134.9 (C, (-), Ar), 129.3 (CH, (+), Ar), 127.3 (CH, (+), Ar), 81.0 (CH_2, (-), *C*H_2NO_2), 70.3 (CH, (+), *C*HOH); IR (neat) ν_{max} = 3470 (broad, m), 2921 (w), 1594 (m), 1549 (s), 1491 (s), 1411 (m), 1377 (s), 1339 (s), 1294 (m), 1207 (m), 1191 (m), 1085 (s), 1014 (s), 895 (m), 823 (s), 739 (s), 660 (m), 524 (s) cm^{-1} [lit:[18] ((*S*)-isomer, KBr): 3436, 2922, 1596, 1557, 1493, 1414, 1379, 1209, 1191, 1089, 1014, 896, 829, 740, 661, 528 cm^{-1}]; MS TOF EI+ (*m/z*): 201.04 $[M]^+$.

Synthesis of (S)-1-(4-chlorophenyl)-2-nitroethanol **(S)-6d**.

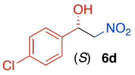

Prepared using *General Procedure G* with 4- chlorobenzaldehyde (70.3 mg, 1.0 equiv., 0.50 mmol), nitromethane (0.27 mL, 10 equiv., 5.0 mmol), $Cu(OAc)_2{\cdot}H_2O$ (4.99 mg, 5 mol%, 0.025 mmol), *N*-benzyl-1-((2*R*, 4*S*)-1-benzyl-4-(4-chlorophenyl)azetidin-2-yl)methanamine (9.42 mg, 5 mol%, 0.025 mmol) and ethanol (1 mL). Purified using a *Combiflash Rf 200i* (silica 4 g column, 0-30% EtOAc/*n*-hexane). The characterisation reported is consistent with that given the literature.[16a, 17] Colourless oil (70 mg, 0.347 mmol, 69% yield); 95% *e.e.* was determined by HPLC using a chiral stationary phase (Eurocel 01, 5 µm, OD, 6.0 % IPA/*n*-hexane, 0.5 mL/min, UV 220 nm), minor enantiomer (*R*) t_r = 20.1 min, major enantiomer (*S*) t_r = 23.8 min); $[\alpha]_D^{21}$ +26.9 (*c* 1.00, CH_2Cl_2); TLC (EtOAc:*n*-hexane= 20:80 v/v, visualised with $KMnO_4$): R_f = 0.26; ^{1}H NMR (300 MHz, $CDCl_3$,): δ 7.40-7.33 (4H, m, Ar*H*), 5.45 (1H, dd, *J* 9.2 & 3.3, C*H*OH), 4.60-4.47 (2H, m, CHC*H*$_2NO_2$), 2.98 (1H, br s, O*H*); ^{13}C NMR (101 MHz, PENDANT, $CDCl_3$): δ 136.5(C, (-), Ar), 134.9 (C, (-), Ar), 129.3 (CH, (+), Ar), 127.3 (CH, (+), Ar), 81.0 (CH_2, (-), *C*H_2NO_2), 70.3 (CH, (+), *C*HOH); IR (neat) ν_{max} = 3467 (broad, m), 2921 (w), 1597 (m), 1549 (s),

1492 (s), 1413 (m), 1377 (s), 1343 (m), 1289 (m), 1209 (m), 1192 (m), 1084 (s), 1014 (s), 895 (m), 825 (s), 739 (s), 660 (m) cm^{-1}; MS TOF AP- (*m/z*): 200.0 $[M-H]^-$. The absolution configuration was assigned as (*S*) by comparison of the optical rotation with the following literature values: [lit[17] $[\alpha]_D^{20}$-38.1 (*c* 1.00, CH_2Cl_2), 92% *e.e.*, (*R*)-isomer], [lit[16a] $[\alpha]_D^{25}$+19.9 (*c* 1.00, CH_2Cl_2), 98% *e.e.*, (*S*)-isomer)].

Synthesis of (rac)*-1-(4-methylphenyl)-2-nitroethanol* **(rac)*-6e*.**

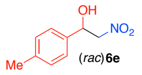

Prepared using *General Procedure F* with *p*-tolualdehyde (0.30 g, 2.5 mmol), nitromethane (0.13 mL, 2.5 mmol), Sodium hydroxide aqueous solution (10 M, 0.25 mL, 2.5 mmol) and acetic acid (0.14 mL, 2.5 mmol) and ethanol (3 mL). Purified using a *Combiflash Rf 200i* (silica 4 g column, 0-30% EtOAc/*n*-hexane). The characterisation reported is consistent with that given the literature (*S* isomer).[18] Colourless solid (0.391 g, 2.16 mmol, 86%). TLC (EtOAc:*n*-hexane = 20:80 v/v, visualised by UV 254 nm):R_f = 0.40; ^{1}H NMR (300 MHz, $CDCl_3$):δ 7.28 (2H, d, *J* 8.1, Ar*H*), 7.20 (2H, d, *J* 8.0, Ar*H*), 5.36 (1H, dd, *J* 9.4 & 3.2, C*H*OH), 4.59 (1H, dd, *J* 13.2 & 9.5, CH*H*NO_2), 4.48 (1H, dd, *J* 13.2 & 3.0, CH*H*NO_2), 2.85 (1H, br, s, O*H*), 2.36 (3H, s, Me); ^{13}C NMR (101 MHz, PENDANT, $CDCl_3$):δ 139.0 (C, (-), Ar), 135.2 (C, (-), Ar), 129.7 (CH, (+), Ar), 125.9 (CH, (+), Ar), 81.3 (CH_2, (-), *C*H_2NO_2), 70.9 (CH, (+), *C*HOH), 21.2 (CH_3, (+), Me); IR (neat) ν_{max} = 3540 (w), 3439 (w), 3028 (w), 2922 (w), 1549 (s), 1417 (m), 1376 (s), 1337 (s), 1206 (m), 1076 (m), 1040 (m), 894 (w), 816 (s), 693 (m) cm^{-1} [lit[18] ((*S*)-isomer, KBr):3539, 3421, 3028, 2922, 1557, 1418, 1378, 1340, 1205, 1078, 1041, 895, 819, 692 cm^{-1}]; MS Scan AP- (*m/z*): 181.2 $[M]^-$.

Synthesis of (S)*-1-(4-methylphenyl)-2-nitroethanol* **(S)*-6e*.**

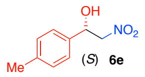

Prepared using *General Procedure G* with *p*-tolualdehyde (60.1 mg, 1.0 equiv., 0.5 mmol), nitromethane (0.27 mL, 10 equiv., 5.0 mmol), $Cu(OAc)_2{\cdot}H_2O$ (4.99 mg, 5 mol%, 0.025 mmol), *N*-benzyl-1-((*2R*, *4S*)-1-benzyl-4-(4-chlorophenyl)azetidin-2-yl)methanamine (9.42 mg, 5 mol%, 0.025 mmol) and ethanol (1 mL). Purified using a *Combiflash Rf 200i* (silica 4 g column, 0-30% EtOAc/*n*-hexane). The characterisation reported is consistent with that given the literature.[16a, 19] Colourless oil (65 mg, 0.359 mmol, 72% yield); 93% *e.e.* was determined by HPLC with a chiral stationary phase (Eurocel 01, 5 μm, OD, 6.0% IPA/*n*-hexane, 0.5 mL/min, UV 210 nm), minor enantiomer (*R*) t_r = 20.6 min, major enantiomer (*S*) t_r = 24.3 min); $[\alpha]_D^{21}$ +35.1 (*c* 1.00, CH_2Cl_2); mp: 55-57 °C; TLC (EtOAc:*n*-hexane= 20:80 v/v, visualised by UV 254 nm): R_f = 0.25; ^{1}H NMR (300 MHz, $CDCl_3$,): δ 7.28 (2H, d, *J* 8.1, Ar*H*), 7.21 (2H, d, *J* 7.9, Ar*H*), 5.41 (1H, dd, *J* 9.6, 3.1 Hz, C*H*OH), 4.59 (1H, dd, *J* 13.2 & 9.6, CH*H*NO_2), 4.48 (1H, dd, *J* 13.2 & 3.0, CH*H*NO_2), 2.74 (1H, br s, O*H*), 2.36 (3H, s, Me); ^{13}C NMR (101 MHz, PENDANT, $CDCl_3$): δ 139.0 (C, (-), Ar), 135.2 (C, (-), Ar), 129.7 (CH, (+), Ar), 125.9 (CH, (+), Ar), 81.3 (CH_2, (-), CH*C*H_2NO_2), 70.9 (CH, (+), Ph*C*HCH_2), 21.2 (CH3, (+), Me); IR (solid) ν_{max} = 3550 (broad, m), 3040 (w), 2956 (m), 1552 (s), 1419 (m), 1381 (s), 1339 (m), 1216 (m), 1077 (s), 1030 (m), 902 (m), 829 (s), 667 (s) cm^{-1}; MS TOF AP- (*m/z*): 180.1 $[M-H]^-$. The absolution configuration was assigned as (*S*) by comparison of the optical rotation with the following

literature values: [lit:[19] $[\alpha]_D^{25}$-26.8 (*c* 4.60, CH_2Cl_2, 93% *e.e.*, (*R*)-isomer)], [lit:[16a] $[\alpha]_D^{25}$+24.8 (*c* 1.10, CH_2Cl_2), 98% *e.e.*, (*S*)-isomer].

Synthesis of (rac)-1-(4-tert-butylphenyl)-2-nitroethanol **(rac)-6f**.

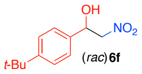
(*rac*) **6f**

Prepared using *General Procedure F* with 4-*tert*-buthylbenzaldehyde (0.30 g, 1.9 mmol), nitromethane (0.1 mL, 1.9 mmol), sodium hydroxide aqueous solution (10 M, 0.19 mL, 1.9 mmol) and acetic acid (0.1 mL, 1.9 mmol) and ethanol (3 mL). Purified using a *Combiflash Rf 200i* (silica 4 g column, 0-30% EtOAc/*n*-hexane). The characterisation (^{1}H and ^{13}C NMR spectroscopy, IR and mass spectra) reported is consistent with that given the literature (*R* isomer).[20] White crystalline solid (0.342 g, 1.53 mmol, 83%). mp: 80-82 °C [lit:[20] (*R*-isomer) 70-72 °C ((*R*)-isomer)]; TLC (EtOAc: *n*-hexane= 20:80 /v, visualized by UV 254 nm): R_f = 0.50; ^{1}H NMR (300 MHz, $CDCl_3$,): δ 7.42 (2H, d, *J* 8.4 Hz, Ar*H*), 7.33 (2H, d, *J* 8.3 Hz, Ar*H*), 5.43 (1H, dd, *J* 9.5, 2.2 Hz, PhC*H*CH_2), 4.65-4.47 (2H, m, C*H_2*NO_2), 2.79 (1H, br, s, O*H*), 1.32 (9H, s, *t*Bu); ^{13}C NMR (101 MHz, PENDANT, $CDCl_3$): δ 152.2 (C, (-), Ar), 135.1 (CH, (-), Ar), 126.0 (CH, (+), Ar), 125.7 (CH, (+), Ar), 81.2 (CH_2, (-), CH*C*H_2NO_2), 70.9 (CH, (+), Ph*C*HCH_2), 34.7 (C, (-), *t*Bu), 31.3 (CH_3, (+), *t*Bu); IR (solid) ν_{max} = 3516 (broad, m), 2953 (m), 2901 (m), 2867 (m), 1552 (s), 1510 (m), 1417 (m), 1383 (s), 1266 (m), 1218 (m), 1106 (m), 1076 (s), 897 (m), 828 (s),748 (m) cm^{-1} [lit:[20] (thin film (*R*)-isomer) 3546, 2963, 2360, 1555, 1378, 1219, 1078, 836, 772 cm^{-1}]; MS TOF ES+ (*m/z*): 246.3 $[M+Na]^+$; HRMS (m/z): $[M+Na]^+$ calcd. for $C_{12}H_{17}NO_3Na$, 246.1106; found, 246.1107.

Synthesis of (S)-1-(4-tert-butylphenyl)-2-nitroethanol **(S)-6f**.

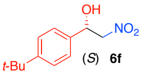
(*S*) **6f**

Prepared using *General Procedure G* with 4-*tert*-buthylbenzaldehyde (81.1 mg, 1.0 equiv., 0.5 mmol), nitromethane (0.27 mL, 10 equiv., 5.0 mmol), $Cu(OAc)_2{\cdot}H_2O$ (4.99 mg, 5 mol%, 0.025 mmol), *N*-benzyl-1-((*2R*, *4S*)-1-benzyl-4-(4-chlorophenyl)azetidin-2-yl)methanamine (9.42 mg, 5 mol%, 0.025 mmol) and ethanol (1 mL). Purified using a *Combiflash Rf 200i* (silica 4 g column, 0-30% EtOAc/*n*-hexane). The characterisation reported is consistent with that given the literature.[20-21] White solid (85.7 mg, 0.384 mmol, 77% yield); 93% *e.e.* was HPLC using a chiral stationary phase (Eurocel 01, 5 µm, OD, 6.0% IPA/*n*-hexane, 0.5 mL/min, UV 220 nm), minor enantiomer (*R*) t_r = 17.1 min, major enantiomer (*S*) t_r = 20.9 min); $[\alpha]_D^{21}$ +23.9 (*c* 1.00, $CHCl_3$); mp: 68-70 °C; TLC (EtOAc:*n*-hexane= 20:80 v/v, visualised with $KMnO_4$): R_f = 0.40; ^{1}H NMR (300 MHz, $CDCl_3$,): δ 7.42 (2H, d, *J* 8.4, Ar*H*), 7.33 (2H, d, *J* 8.3, Ar*H*), 5.44 (1H, dd, *J* 9.6 & 3.0, C*H*OH), 4.62 (1H, dd, *J* 13.3 & 9.7, CHC*H*HNO_2), 4.51 (1H, dd, *J* 13.3 & 3.0, CHCH*H*NO_2), 2.73 (1H, br, s, O*H*), 1.32 (9H, s, *t*-Bu); ^{13}C NMR (101 MHz, PENDANT, $CDCl_3$): δ 152.2 (C, (-), Ar), 135.1 (CH, (-), Ar), 126.0 (CH, (+), Ar), 125.7 (CH, (+), Ar), 81.2 (CH_2, (-), CH*C*H_2NO_2), 70.9 (CH, (+), Ph*C*HCH_2), 34.7 (C, (-), *t*-Bu), 31.3 (CH_3, (+), *t*-Bu); IR (solid) ν_{max} = 3550 (broad, m), 2955 (m), 2901 (m), 2867 (m), 1553 (s), 1510 (m), 1420 (m), 1385 (s), 1266 (m), 1216 (m), 1107 (m), 1076 (s), 902 (s), 829 (s), 749 (m), 667 (s) cm^{-1}; MS TOF AP- (*m/z*): 222.1 $[M-H]^-$. The absolution configuration was assigned as (*S*) by comparison of the optical rotation with the following literature values: [lit:[20] $[\alpha]_D^{21}$-31.35 (*c* 0.7, $CHCl_3$, 92% *e.e.*, (*R*)-isomer)], [lit:[21] $[\alpha]_D^{25}$+38.3 (*c* 1.00, $CHCl_3$, 99% *e.e.*, (*S*)-isomer)].

Synthesis of (rac)*-1-(1-naphthyl)-2-nitroethanol* **(rac)*-6g*.**

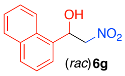

Prepared using *General Procedure F* with 1-naphthaldehyde (0.30 g, 1.9 mmol), nitromethane (0.10 mL, 1.92 mmol), sodium hydroxide aqueous solution (10 M, 0.19 mL, 1.9 mmol) and acetic acid (0.11 mL, 1.9 mmol) and ethanol (3 mL). Purified using a *Combiflash Rf 200i* (silica 4 g column, 0-30% EtOAc/*n*-hexane). The characterisation reported is consistent with that given the literature (*S* isomer).[18] Yellow oil (0.324 g, 78% yield); TLC (EtOAc:*n*-hexane = 20:80 v/v, visualised by UV 254 nm): R_f = 0.47; ^{1}H NMR (300 MHz, $CDCl_3$): δ 8.04 (1H, d, *J* 8.7, Ar*H*), 7.93-7.85 (2H, m, Ar*H*), 7.77 (1H, d, *J* 7.2, Ar*H*), 7.63-7.50 (3H, m, Ar*H*), 6.28 (1H, dd, *J* 8.1 & 3.8, C*H*OH), 4.69-4.66 (2H, m, CH_2NO$_2$), 2.88 (1H, br, s, O*H*); ^{13}C NMR (101 MHz, PENDANT, $CDCl_3$): δ 133.8 (C, (-), Ar), 133.5 (C, (-), Ar), 129.6 (C, (-), Ar), 129.4 (CH, (+), Ar), 129.3 (CH, (+), Ar), 127.1 (CH, (+), Ar), 126.1 (CH, (+), Ar), 125.5 (CH, (+), Ar), 123.9 (CH, (+), Ar), 121.8 (CH, (+), Ar), 80.8 (CH_2, (-), *C*H_2NO_2), 68.3 (CH, (+), *C*HOH); IR (neat) ν_{max} = 3512 (broad, m), 3056 (w), 2918 (w), 1598 (w), 1549 (s), 1508 (s), 1377 (s), 1334 (s), 1202 (m), 1165 (m), 1096 (m), 1047 (m), 896 (m), 802 (s), 773 (s), 696 (m), 623 (m) cm^{-1} [lit:[18] ((*S*)-isomer, KBr): 3543, 3060, 3930, 1554, 1513, 1378, 1201, 1166, 1098, 1052, 897, 804, 776, 697, 624 cm^{-1}]; MS TOF ES- (*m/z*): 216.1 $[M-H]^-$.

Synthesis of (S)*-1-(1-naphthyl)-2-nitroethanol* **(S)*-6g*.**

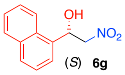

Prepared using *General Procedure G* with 1-naphthaldehyde (78.1 mg, 1.0 equiv., 0.5 mmol), nitromethane (0.27 mL, 10 equiv., 5 mmol), $Cu(OAc)_2{\cdot}H_2O$ (4.99 mg, 5 mol%, 0.025 mmol), *N*-benzyl-1-((2*R*, 4*S*)-1-benzyl-4-(4-chlorophenyl)azetidin-2-yl)methanamine (9.42 mg, 5 mol%, 0.025 mmol) and ethanol (1 mL). Purified using a *Combiflash Rf 200i* (silica 4 g column, 0-30% EtOAc/*n*-hexane). The characterisation reported is consistent with that given the literature.[16] Yellow oil (93.0 mg, 0.428 mmol, 86% yield); 93% *e.e.* was determined by HPLC with a chiral stationary phase (Eurocel 01, 5 µm, OD, 6.0 % IPA/*n*-hexane, 0.5 mL/min, UV 254 nm), minor enantiomer (*R*) t_r = 23.1 min, major enantiomer (*S*) t_r = 34.7 min; $[\alpha]_D^{21}$ +18.3 (*c* 1.00, CH_2Cl_2); TLC (EtOAc:*n*-hexane= 20:80 v/v, visualised by UV 254 nm): R_f = 0.33; ^{1}H NMR (400 MHz, $CDCl_3$,): δ 8.03-7.93 (1H, m, Ar*H*), 7.89 (2H, ddt, *J* 20.9, 8.2 & 0.9, Ar*H*), 7.77 (1H, dt, *J* 7.2 & 1.0, Ar*H*), 7.60 (1H, m, Ar*H*), 7.60-7.47 (2H, m, Ar*H*), 6.27 (1H, dt, *J* 8.5 & 3.1, C*H*OH), 4.69-4.66 (2H, m, CH_2NO$_2$), 2.82-2.90 (1H, br s, O*H*); ^{13}C NMR (101 MHz, PENDANT, $CDCl_3$): δ 133.8 (C, (-), Ar), 133.5 (C, (-), Ar), 129.6 (C, (-), Ar), 129.5 (CH, (+), Ar), 129.4 (CH, (+), Ar), 127.1 (CH, (+), Ar), 126.1 (CH, (+), Ar), 125.5 (CH, (+), Ar), 123.9 (CH, (+), Ar), 121.8 (CH, (+), Ar), 80.8 (CH_2, (-), *C*H_2NO_2), 68.4 (CH, (+), *C*HOH); IR (neat) ν_{max} = 3527 (broad, m), 3058 (w), 2921 (w), 1598 (w), 1547 (s), 1511 (s), 1376 (s), 1331 (m), 1201 (m), 1166 (m), 1097 (m), 1047 (m), 895 (m), 803 (s), 773 (s), 696 (m) cm^{-1}; MS TOF AP+ (*m/z*): 217.1 $[M]^+$. The absolution configuration was assigned as (*S*) by comparison of the optical rotation with the following literature values: [lit:[16b] $[\alpha]_D^{23}$ -24.9 (*c* 1.08, CH_2Cl_2, 87% *e.e.*, (*R*)-isomer)], [lit:[16a] $[\alpha]_D^{25}$ +26.0 (*c* 1.06, CH_2Cl_2, 98% *e.e.*, (*S*)-isomer)].

Synthesis of (rac)*-1-(biphenyl-2yl)-2-nitroethanol* **(rac)*-6h***.

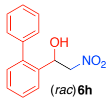

Prepared using *General Procedure F* with biphenyl-2-carboxaldehyde (0.10 g, 0.55 mmol), nitromethane (0.03 mL, 0.6 mmol), sodium hydroxide aqueous solution (10 M, 0.06 mL, 0.6 mmol) and acetic acid (0.03 mL, 0.5 mmol) and ethanol (2mL). Purified using a *Combiflash Rf 200i* (silica 4 g column, 0-30% EtOAc/*n*-hexane). The characterisation reported is consistent with that given the literature (single enantiomer product).[18] Colourless oil (0.112 g, 0.460 mmol, 84% yield); TLC (EtOAc:*n*-hexane = 20:80 v/v, visualised by UV 254 nm): R_f = 0.43; ^{1}H NMR (300 MHz, $CDCl_3$): δ 7.65 (1H, dd, *J* 7.5, 1.7 Hz, Ar*H*), 7.50-7.35 (5H, m, Ar*H*), 7.32-7.24 (3H, m, Ar*H*), 5.58 (1H, dd, *J* 9.6 & 2.7, C*H*OH), 4.50 (1H, dd, *J* 13.5 & 9.6, CHC*H*HNO$_2$), 4.34 (1H, dd, *J* 13.5 & 2.7), 2.80 (1H, br s, O*H*); ^{13}C NMR (101 MHz, PENDANT, $CDCl_3$): δ 140.9 (C, (-), Ar), 139.8 (C, (-), Ar), 135.4 (C, (-), Ar), 130.5 (CH, (+), Ar), 128.9 (CH, (+), Ar), 128.8 (CH, (+), Ar), 128.7 (CH, (+), Ar), 128.3 (CH, (+), Ar), 127.9 (CH, (+), Ar), 126.1 (CH, (+), Ar),80.3 (CH_2, (-), CH*C*H$_2$NO$_2$), 67.6 (CH, (+), Ph*C*HOH); IR (neat) ν_{max} = 3479 (broad, m), 3060 (w), 3026 (w), 1708 (w), 1596 (w), 1550 (s), 1477(s), 1376 (s), 1281 (m), 1188 (m), 1068 (s), 1009 (m), 893 (m), 763 (s), 702 (s), 607 (s) cm^{-1} [lit:[18] ((*S*)-isomer, KBr):3544, 3060, 3026, 1683, 1596, 1556, 1478, 1377, 1340, 1282, 1188, 1069, 1009, 894, 764, 704, 608 cm^{-1}]; MS TOF AP- (*m/z*): 243.1 [M]$^-$.

Synthesis of (S)*-1-(biphenyl-2yl)-2-nitroethanol* **(S)*-6h***.

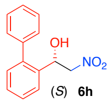

Prepared using *General Procedure G* with biphenyl-2-carboxaldehyde (91.1 mg, 1.0 equiv., 0.50 mmol), nitromethane (0.27 mL, 10 equiv., 5.0 mmol), $Cu(OAc)_2{\cdot}H_2O$ (4.99 mg, 5 mol%, 0.03 mmol), *N*-benzyl-1-((2*R*, 4*S*)-1-benzyl-4-(4-chlorophenyl)azetidin-2-yl)methanamine (9.42 mg, 5 mol%, 0.03 mmol) and ethanol (1 mL). Purified using a *Combiflash Rf 200i* (silica 4 g column, 0-30% EtOAc/*n*-hexane). The characterisation reported is consistent with that given the literature.[18] Colourless oil (68.0 mg, 56% yield); 87% *e.e.* was determined by HPLC with a chiral stationary phase (Eurocel 01, 5 µm, OD, 3.0 % IPA/*n*-hexane, 0.5 mL/min, UV 220 nm), minor enantiomer (*R*) t_r = 17.5 min, major enantiomer (*S*) t_r = 20.3 min); $[\alpha]_D^{21}$ +9.1 (*c* 1.00, CH_2Cl_2); TLC (EtOAc:*n*-hexane= 20:80 v/v, visualised by UV 254 nm): R_f = 0.32; ^{1}H NMR (300 MHz, $CDCl_3$,): δ 7.66-7.28 (9H, m, Ar*H*), 5.58 (1H, dd, *J* 9.6, 2.7 Hz, C*H*OH), 4.51 (1H, dd, *J* 13.6, 9.6, CHC*H*HNO$_2$), 4.35 (1H, dd, *J* 13.6, 2.7, CHCH*H*NO$_2$), 2.75 (1H, br s, O*H*); ^{13}C NMR (101 MHz, PENDANT, $CDCl_3$): δ 140.9 (C, (-), Ar), 139.8 (C, (-), Ar), 135.4 (C, (-), Ar), 130.5 (CH, (+), Ar), 128.9 (CH, (+), Ar), 128.8 (CH, (+), Ar), 128.7 (CH, (+), Ar), 128.3 (CH, (+), Ar), 127.9 (CH, (+), Ar), 126.1 (CH, (+), Ar), 80.3 (CH_2, (-), CH*C*H$_2$NO$_2$), 67.6 (CH, (+), Ph*C*HOH); IR (neat) ν_{max} = 3540 (broad, m), 3060 (w), 3026 (w), 1710 (w), 1597 (w), 1549 (s), 1478 (m), 1376 (s), 1280 (m), 1188 (m), 1068 (s), 1009 (m), 893 (m), 763 (s), 702 (s) cm^{-1}; MS TOF AP- (*m/z*): 242.1 [M-H]$^-$. The absolution configuration was assigned as (*S*) by comparison of the optical rotation with the following literature values: [lit:[18] $[\alpha]_D^{20}$+23.0 (*c* 1.00, CH_2Cl_2, 90% *e.e.*, (*S*)-isomer)].

*Synthesis of (*rac*)-1-cyclohexyl-2-nitroethanol* ***(*****rac*****)-6i*.**

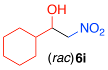
(*rac*) **6i**

Prepared using *General Procedure F* with cyclohexanecarboxaldehyde (0.30 g, 2.7 mmol), nitromethane (0.15 mL, 2.7 mmol), Sodium hydroxide aqueous solution (10 M, 0.27 mL, 2.7 mmol) and acetic acid (0.15 mL, 2.7 mmol) and ethanol (3 mL). Purified using a *Combiflash Rf 200i* (silica 4 g column, 0-30% EtOAc/*n*-hexane). The characterisation (^{1}H and ^{13}C NMR spectroscopy, IR and mass spectra) reported is consistent with that given the literature (racemic product).[15] Colourless oil (0.36 g, 2.1 mmol, 78% yield). TLC (EtOAc:*n*-hexane = 20:80 v/v, visualised by UV 254 nm): R_f = 0.34; ^{1}H NMR (300 MHz, $CDCl_3$): δ 4.51 (1H, dd, *J* 13.1 & 3.2, CHC*H*HNO_2), 4.43 (1H, dd, *J* 13. & 8.6, CHCH*H*NO_2), 4.14-4.08 (1H, m, C*H*OH), 2.62 (1H, br, s, O*H*), 1.86-1.78 (3H, m, Cy), 1.72-1.66 (2H, m, Cy), 1.55-1.43 (1H, m, Cy), 1.33-1.05 (5H, m, Cy); ^{13}C NMR (101 MHz, PENDANT, $CDCl_3$): δ 79.3 (CH_2, (-),CH*C*H_2NO_2), 72.9 (CH, (+), *C*HOH), 41.4 (CH, (+), Cy), 28.8 (CH_2, (-), Cy), 28.0 (CH_2, (-), Cy), 26.1 (CH_2, (-), Cy), 25.9 (CH_2, (-), Cy), 25.8 (CH_2, (-), Cy); IR (neat) ν_{max} = 3454 (broad, m), 2925 (s), 2853 (m), 1551 (s), 1450 (m), 1423 (w), 1382 (m), 1066 (m), 1203 (m), 1046 (m), 894 (m), 651 (m) cm^{-1} [lit:[15] (racemic, film):3450, 2925, 2855, 1557, 1450, 1424, 1376 cm^{-1}]; MS Scan AP+ (*m/z*): 174.3 $[M+H]^+$.

*Synthesis of (*S*)-1-cyclohexyl-2-nitroethanol* ***(S)-6i*.**

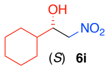
(*S*) **6i**

Prepared using *General Procedure G* with cyclohexanecarboxaldehyde (56.1 mg, 1.0 equiv., 0.5 mmol), nitromethane (0.27 mL, 10 equiv., 5.0 mmol), $Cu(OAc)_2{\cdot}H_2O$ (4.99 mg, 5 mol%, 0.025 mmol), *N*-benzyl-1-((2*R*, 4*S*)-1-benzyl-4-(4-chlorophenyl)azetidin-2-yl)methanamine (9.42 mg, 5 mol%, 0.025 mmol) and ethanol (1 mL). Purified using a *Combiflash Rf 200i* (silica 4 g column, 0-30% EtOAc/*n*-hexane). The characterisation reported is consistent with that given the literature.[16] Colourless oil (74.0 mg, 0.271 mmol., 54% yield); >99% *e.e.* was determined by chiral HPLC (Eurocel 01, 5 µm, OD, 6.0 % IPA/*n*-hexane, 0.5 mL/min, UV 210 nm), (major enantiomer (*S*) t_r = 40.3 min); $[\alpha]_D^{21}$ +5.2 (*c* 1.00, CH_2Cl_2); TLC (EtOAc:*n*-hexane= 20:80 v/v, visualised with $KMnO_4$): R_f = 0.49; ^{1}H NMR (400 MHz, $CDCl_3$,): δ 4.49 (1H, dd, *J* 13.1 & 3.0, CHC*H*HNO_2), 4.43 (1H, dd, *J* 13.1 & 8.9, CHCH*H*NO_2), 4.12-4.08 (1H, m, C*H*OH), 2.68 (1H, br s, O*H*), 1.86-1.77 (3H, m, Cy), 1.72-1.65 (2H, m, Cy), 1.52-1.43 (1H, m, Cy), 1.32-1.04 (5H, m, Cy); ^{13}C NMR (101 MHz, PENDANT, $CDCl_3$): δ 79.3 (CH_2, (-),CH*C*H_2NO_2), 72.9 (CH, (+), *C*HOH), 41.4 (CH, (+), Cy), 28.8 (CH_2, (-), Cy), 28.0 (CH_2, (-), Cy), 26.1 (CH_2, (-), Cy), 25.9 (CH_2, (-), Cy), 25.8 (CH_2, (-), Cy); IR (neat) ν_{max} = 3411 (broad, m), 2926 (s), 2854 (m), 1550 (s), 1450 (m), 1424 (w), 1384 (m), 1202 (m), 1066 (m), 1045 (m), 894 (m) cm^{-1}; MS TOF AP- (*m/z*): 172.1 $[M-H]^-$. The absolution configuration was assigned as (*S*) by comparison of the optical rotation with the following literature values: [lit[16b] $[\alpha]_D^{21}$ -21.6 (*c* 1.33, $CHCl_3$, 93% *e.e.*, (*R*) isomer)], [lit[16a] $[\alpha]_D^{25}$ +18.0 (*c* 1.02, CH_2Cl_2), 93% *e.e.*, (*S*) isomer].

*Synthesis of (*rac*)-3,3-dimethyl-1-nitrobutan-2-ol* ***(*****rac*****)-6j*.**

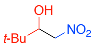
(*rac*) **6j**

Prepared using *General Procedure F* with trimethylacetaldehyde (0.20 g, 2.3 mmol), nitromethane (0.13 mL, 2.3 mmol), sodium hydroxide aqueous solution (10 M, 0.23 mL, 2.3 mmol) and acetic acid (0.13 mL, 2.3 mmol) and ethanol (2 mL). The characterisation (^{1}H and ^{13}C NMR spectroscopy, IR and mass spectra) reported is

consistent with that given the literature (racemic product).[15] Colourless oil (0.263 g, 1.79 mmol., 77% yield). TLC (EtOAc: hexane = 20:80 v/v, visualised with $KMnO_4$): R_f = 0.42; ^{1}H NMR (300 MHz, $CDCl_3$,): δ 4.53 (1H, dd, *J* 13.0 & 2.2, 1H), 4.37 (1H, dd, *J* 13.0 & 10.1, CH*H*NO_2), 4.07-4.01 (1H, m, C*H*OH), 2.38-2.42 (1H, br s, O*H*), 0.98 (9H, s, *t*-Bu); ^{13}C NMR (101 MHz, PENDANT, $CDCl_3$): δ 78.3 (CH_2, (-), CH*C*H_2NO_2), 76.2 (CH, (+), *C*HCH_2), 34.3 (C, (-), *t*-Bu), 25.6 (CH_3, (+), *t*-Bu); IR (neat) ν_{max} = 3479 (broad, m), 2962 (m), 2875 (m), 1551 (s) 1480 (m), 1382 (m), 1348 (m), 1191 (m), 1085 (s), 1007 (m), 924 (m), 881 (m), 791 (w), 699 (s) cm^{-1} [lit:[15] (racemic, film): 3554, 2963, 2874, 1560, 1480, 1382, 1347 cm^{-1}]; MS TOF AP+ (*m/z*): 148.1 $[M+H]^+$.

Synthesis of (S)-3,3-dimethyl-1-nitrobutan-2-ol ***(S)-6j****.*

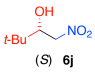

Prepared using *General Procedure G* with trimethylacetaldehyde (43.1 mg, 1.0 equiv., 0.5 mmol), nitromethane (0.27 mL, 10 equiv., 5.0 mmol), $Cu(OAc)_2{\cdot}H_2O$ (4.99 mg, 5 mol%, 0.025 mmol), *N*-benzyl-1-((*2R*, *4S*)-1-benzyl-4-(4-chlorophenyl)azetidin-2-yl)methanamine (9.42 mg, 5 mol%, 0.025 mmol) and ethanol (1 mL). Purified using a *Combiflash Rf 200i* (silica 4 g column, 0-30% EtOAc/*n*-hexane). Colourless oil (45.0 mg, 61% yield); >99% *e.e.* was determined by HPLC with a chiral stationary phase (Eurocel 01, 5 µm, OD, 0.8 % IPA/*n*-hexane, 0.8 mL/min, UV 210 nm), minor enantiomer (*R*) t_r = 14.2 min, major enantiomer (*S*) t_r = 15.3 min; $[\alpha]_D^{21}$ +20.9 (*c* 1.00, CH_2Cl_2); TLC (EtOAc:*n*-hexane= 20:80 v/v, visualised with $KMnO_4$): R_f = 0.47; ^{1}H NMR (400 MHz, $CDCl_3$,): δ 4.76-4.12 (2H, m, CHC*H*$_2$$NO_2$), 4.04 (1H, dq, *J* 10.2 & 2.1, C*H*OH), 2.40 (1H, br s, O*H*), 0.98 (9H, s, *t*-Bu); ^{13}C NMR (101 MHz, PENDANT, $CDCl_3$): δ 78.3 (CH_2, (-), CH*C*H_2NO_2), 76.2 (CH, (+), *C*HCH_2), 34.3 (C, (-), *t*-Bu), 25.6 (CH_3, (+), *t*-Bu); IR (neat) ν_{max} = 3500 (broad, m), 2963 (m), 2875 (m), 1551 (s) 1480 (m), 1382 (m), 1348 (m), 1191 (m), 1084 (s), 1007 (m), 923 (m), 880 (m), 791 (w), 699 (s) cm^{-1}; MS TOF AP- (*m/z*): 146.1 $[M-H]^-$. The absolution configuration was assigned as (*S*) by comparison of the optical rotation with the following literature values: [lit:[16b] $[\alpha]_D^{21}$ -35.9 (*c* 1.01, CH_2Cl_2, 94% *e.e.*, (*R*)-isomer)], [lit $[\alpha]_D^{25}$ +33.02 (*c* 3.71, CH_2Cl_2, 92% *e.e.*, (*S*)-isomer)].

Synthesis of (rac)-N-benzyl-1-((cis)-1-benzyl-4-phenylazetidin-2-yl)methanamine platinum(II)chloride **(rac)-7***.*

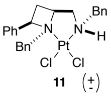

Under nitrogen atmosphere, K_2PtCl_4 (0.26 mmol, 110 mg) was added to a solution of methanol and *rac*-**1r** (0.29 mol, 100 mg). The mixture was stirred for 16 hours at 60 °C after which the solvent was removed *in vacuo* and crystalisation from $CH_3CN:Et_2O$ afforded a small number of yellow crystals, suitable for analysis by XRD, see later. M/z: (ES^+) $[M-Cl]^+$ 572.4, $[M-2Cl]^+$ 536.1.

Preliminary organocatalysis experiment

To further exemplify the potential of the 2,4-*cis*-substituted azetidine structure as a platform for asymmetric catalysis a preliminary organocatalysis experiment was conducted.[22] Menguy and Couty had already demonstrated an *in situ* preparation of amino azetidine thiourea derivatives and applied this scaffold to catalytic asymmetric Michael additions of diethyl malonate to nitrovinyl Michael acceptors.[23]

To give a preliminary indication of the stereoinduction capability of 2,4-*cis-substituted* azetidines in organocatalysis the Menguy and Couty protocol was attempted. An *in situ* prepared thiourea catalyst **S7** was able to deliver product **S8** in 89% e.e. albeit in only 20% yield (**Scheme S 6a**).[24] Catalyst **S7** was prepared according to the Menguy and Couty procedure from homochiral azetidine derivative **1s** which was accessed from **1r** by the same method, previously reported by Feula and Fossey.[14,25]

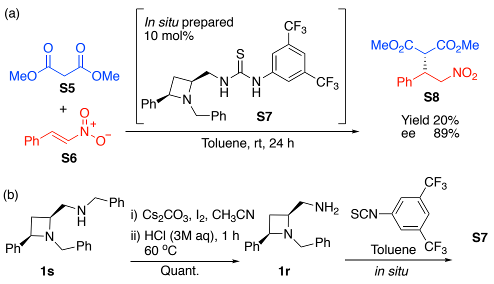

***Scheme S 6.** Preliminary experiment which demonstrates the* in situ *generation and use of a thiourea derivative S7 as a promising organocatalyst for Michael-type reactions of malonates with nitroalkenes.*

This single, un-optimised, attempt to prepare **S8** under control of an organocatalytic azetidine derivative (**Scheme S 6a**) demonstrates the plural capacity for the azetidine scaffold to engender asymmetry across a range of reaction manifolds. Exploration of the organocatalytic potential of *cis*-disubstituted azetidine scaffold will be the subject of further study emerging from the laboratories of this team.

*Synthesis of (S)-dimethyl 2-(2-nitro-1-phenylethyl)malonate **S8**.*

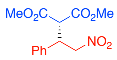

S8

1-Isothiocyanato-3,5-bis(trifluoromethyl)benzene (0.04 mmol, 11 mg) was stirred with (2*S*,4*R*)-**1r** (0.04 mmol, 10 mg) for 30 minutes in dry toluene under argon atmosphere after which (*E*)-(2-nitrovinyl)benzene (0.40 mmol, 59 mg) and dimethyl malonate (0.80 mmol, 106 mg) were added and the mixture was stirred at rt temperature for 48 hours. Toluene was removed *in vacuo* and the product was purified by flash chromatography (silica, hexane:ethyl acetate 7:3). Yield = 23 mg, 20%. $[\alpha]_D^{25}$= +5.5 (*c* 1.0, $CHCl_3$). Absolute stereochemistry (*S*) and *e.e.* were assigned by comparison of the specific optical rotation with the literature ($[\alpha]_D^{25}$= + 5.9; 96% *e..e*).[24] ^{1}H NMR (δ; 300 MHz, $CDCl_3$); 3.49 (3H, s, OC*H*$_3$), 3.70 (3H, s, OC*H*$_3$), 3.80 (1H, d, *J* 9.0, MeO(O)CC*H*), 4.14-4.22 (1H, m, PhC*H*CH_2NO_2), 4.80-4.90 (2H, m, C*H*$_2NO_2$), 7.14-7.29 (5H, m, Ph*H*); ^{13}C NMR (δ; 100 MHz, $CDCl_3$), 45.34 (CH), 53.69 (CH_3), 54.01 (CH_3), 56.46 (CH), 79.41 (CH_2), 129.92 (CH), 130.11 (CH), 130.39 (CH), 138.69 (C), 169.65 (C), 170.12 (C). M/z: (EI$^+$) 281 [M]$^+$.

HPLC Traces

*HPLC Trace (rac)-**6a***

Operator:Shimadzu Timebase:LC_System1 Sequence:15%MeCN v 1

Page 1-1
28/1/2017 9:27 PM

34 AY-Cat 010

Sample Name:	**AY-Cat 010**	*Injection Volume:*	**5.0**
Vial Number:	**1_1**	*Channel:*	**UV_VIS_3**
Sample Type:	**unknown**	*Wavelength:*	**254**
Control Program:	**15% MeCN v 1**	*Bandwidth:*	**8**
Quantif. Method:	**15% MeCN v 1**	*Dilution Factor:*	**1.0000**
Recording Time:	**3/12/2016 15:10**	*Sample Weight:*	**1.0000**
Run Time (min):	**60.01**	*Sample Amount:*	**1.0000**

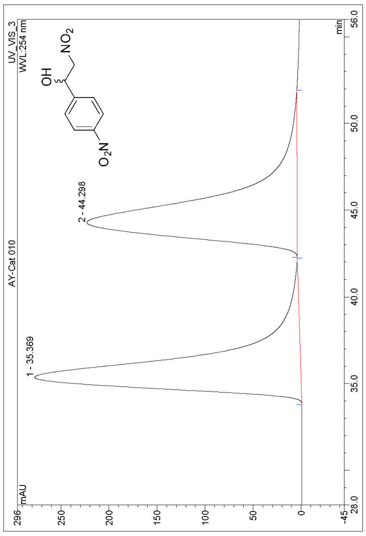

No.	Ret.Time min	Peak Name	Height mAU	Area mAU*min	Rel.Area %	Amount	Type
1	35.37	n.a.	277.244	553.276	50.43	n.a.	BMB
2	44.30	n.a.	218.998	543.747	49.57	n.a.	BMB
**Total:**			496.242	1097.024	100.00	0.000	

DEFAULT/Integration

Chromeleon (c) Dionex 1996-2006
Version 6.80 SR15 Build 4656 (243203)

*HPLC Trace (S)-**6a***

Operator:Shimadzu Timebase:LC_System1 Sequence:15%MeCN v 1

Page 1-1
28/1/2017 9:31 PM

36 AY-Cat 106

Sample Name:	**AY-Cat 106**	*Injection Volume:*	**5.0**
Vial Number:	**1_3**	*Channel:*	**UV_VIS_3**
Sample Type:	**unknown**	*Wavelength:*	**254**
Control Program:	**15% MeCN v 1**	*Bandwidth:*	**8**
Quantif. Method:	**15% MeCN v 1**	*Dilution Factor:*	**1.0000**
Recording Time:	**3/12/2016 17:11**	*Sample Weight:*	**1.0000**
Run Time (min):	**60.01**	*Sample Amount:*	**1.0000**

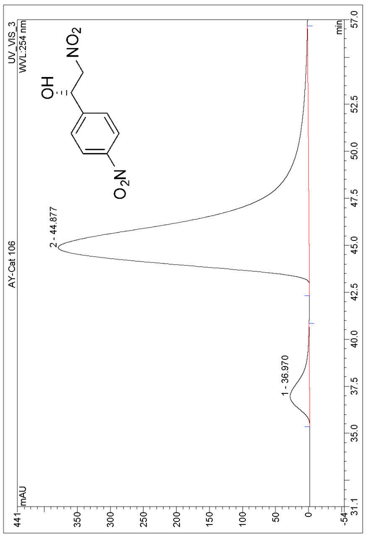

No.	Ret.Time min	Peak Name	Height mAU	Area mAU*min	Rel.Area %	Amount	Type
1	36.97	n.a.	29.427	53.595	4.89	n.a.	BMB*
2	44.88	n.a.	379.571	1042.560	95.11	n.a.	BMB*
**Total:**			408.998	1096.154	100.00	0.000	

DEFAULT/Integration

Chromeleon (c) Dionex 1996-2006
Version 6.80 SR15 Build 4656 (243203)

HPLC Trace (rac)-***6b***

60 AY-Cat 005

Sample Name:	**AY-Cat 005**	*Injection Volume:*	**5.0**
Vial Number:	**1_17**	*Channel:*	**UV_VIS_1**
Sample Type:	**unknown**	*Wavelength:*	**220**
Control Program:	**ISOCRATIC 95 5 Hexane IPA**	*Bandwidth:*	**n.a.**
Quantif. Method:	**ISOCRATIC 95 5 Hexane IPA**	*Dilution Factor:*	**1.0000**
Recording Time:	**5/12/2016 5:48**	*Sample Weight:*	**1.0000**
Run Time (min):	**50.10**	*Sample Amount:*	**1.0000**

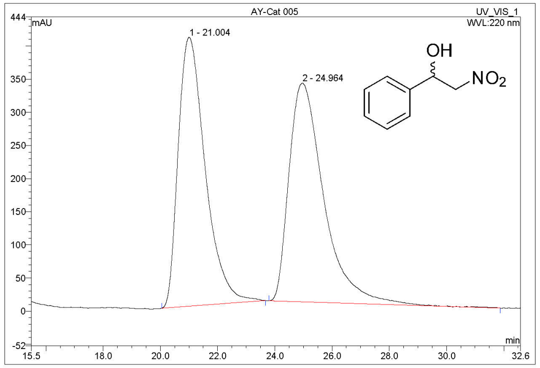

No.	Ret.Time min	Peak Name	Height mAU	Area mAU*min	Rel.Area %	Amount	Type
1	21.00	n.a.	405.733	444.217	48.87	n.a.	BMB*
2	24.96	n.a.	330.167	464.704	51.13	n.a.	BMB*
**Total:**			735.900	908.921	100.00	0.000	

HPLC Trace (S)-***6b***

61 AY-Cat 123

Sample Name:	**AY-Cat 123**	*Injection Volume:*	**5.0**
Vial Number:	**1_18**	*Channel:*	**UV_VIS_1**
Sample Type:	**unknown**	*Wavelength:*	**220**
Control Program:	**ISOCRATIC 95 5 Hexane IPA**	*Bandwidth:*	**n.a.**
Quantif. Method:	**ISOCRATIC 95 5 Hexane IPA**	*Dilution Factor:*	**1.0000**
Recording Time:	**5/12/2016 6:39**	*Sample Weight:*	**1.0000**
Run Time (min):	**50.10**	*Sample Amount:*	**1.0000**

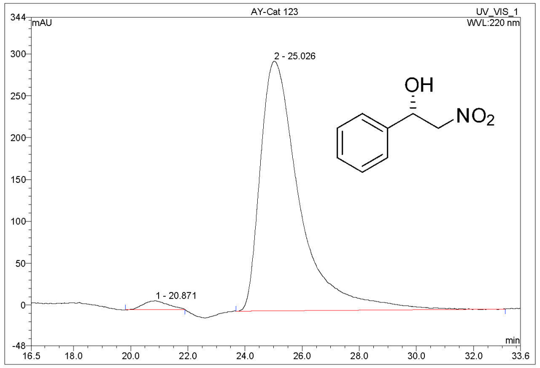

No.	Ret.Time min	Peak Name	Height mAU	Area mAU*min	Rel.Area %	Amount	Type
1	20.87	n.a.	10.398	10.567	2.18	n.a.	BMB*
2	25.03	n.a.	298.191	474.361	97.82	n.a.	BMB*
**Total:**			308.589	484.927	100.00	0.000	

*HPLC Trace (rac)-**6c***

Operator:Shimadzu Timebase:LC_System2 Sequence:ISOCRATIC 95 5 Hexane IPA   Page 1-1
11/12/2016 11:23 PM

93 AY-Cat 012

Sample Name:	**AY-Cat 012**	*Injection Volume:*	**5.0**
Vial Number:	**1_11**	*Channel:*	**UV_VIS_2**
Sample Type:	**unknown**	*Wavelength:*	**230**
Control Program:	**ISOCRATIC 95 5 Hexane IPA**	*Bandwidth:*	**n.a.**
Quantif. Method:	**ISOCRATIC 95 5 Hexane IPA**	*Dilution Factor:*	**1.0000**
Recording Time:	**6/12/2016 14:03**	*Sample Weight:*	**1.0000**
Run Time (min):	**50.10**	*Sample Amount:*	**1.0000**

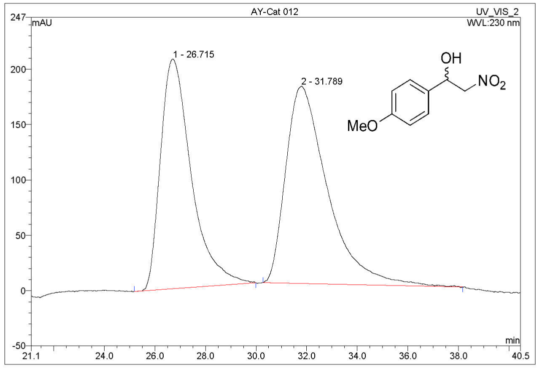

No.	Ret.Time min	Peak Name	Height mAU	Area mAU*min	Rel.Area %	Amount	Type
1	26.72	n.a.	207.279	292.992	46.20	n.a.	BMB*
2	31.79	n.a.	178.121	341.236	53.80	n.a.	BMB*
**Total:**			385.401	634.228	100.00	0.000	

DEFAULT/Integration   Chromeleon (c) Dionex 1996-2006
Version 6.80 SR15 Build 4656 (243203)

*HPLC Trace (S)-**6c***

Operator:Shimadzu Timebase:LC_System2 Sequence:ISOCRATIC 95 5 Hexane IPA   Page 1-1
28/1/2017 9:50 PM

119 AY-Cat 111

Sample Name:	**AY-Cat 111**	*Injection Volume:*	**5.0**
Vial Number:	**1_20**	*Channel:*	**UV_VIS_2**
Sample Type:	**unknown**	*Wavelength:*	**230**
Control Program:	**ISOCRATIC 95 5 Hexane IPA**	*Bandwidth:*	**n.a.**
Quantif. Method:	**ISOCRATIC 95 5 Hexane IPA**	*Dilution Factor:*	**1.0000**
Recording Time:	**11/12/2016 22:35**	*Sample Weight:*	**1.0000**
Run Time (min):	**50.10**	*Sample Amount:*	**1.0000**

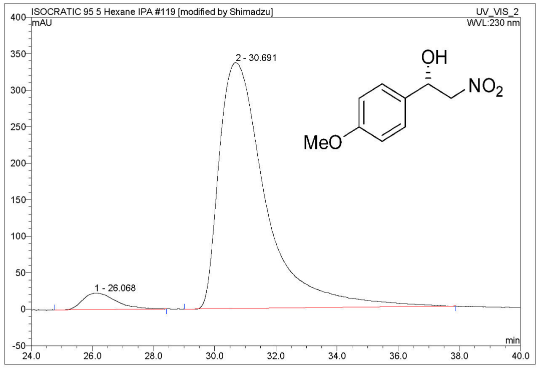

No.	Ret.Time min	Peak Name	Height mAU	Area mAU*min	Rel.Area %	Amount	Type
1	26.07	n.a.	22.410	27.895	4.47	n.a.	BMB*
2	30.69	n.a.	336.771	596.000	95.53	n.a.	BMB*
**Total:**			359.181	623.895	100.00	0.000	

DEFAULT/Integration   Chromeleon (c) Dionex 1996-2006
Version 6.80 SR15 Build 4656 (243203)

HPLC Trace (rac)-**6d**

Operator:Shimadzu Timebase:LC_System2 Sequence:ISOCRATIC 95 5 Hexane IPA Page 1-1
11/12/2016 11:30 PM

68 AY-Cat 006

Sample Name:	**AY-Cat 006**	*Injection Volume:*	**5.0**
Vial Number:	**1_14**	*Channel:*	**UV_VIS_1**
Sample Type:	**unknown**	*Wavelength:*	**220**
Control Program:	**ISOCRATIC 95 5 Hexane IPA**	*Bandwidth:*	**n.a.**
Quantif. Method:	**ISOCRATIC 95 5 Hexane IPA**	*Dilution Factor:*	**1.0000**
Recording Time:	**5/12/2016 12:33**	*Sample Weight:*	**1.0000**
Run Time (min):	**50.10**	*Sample Amount:*	**1.0000**

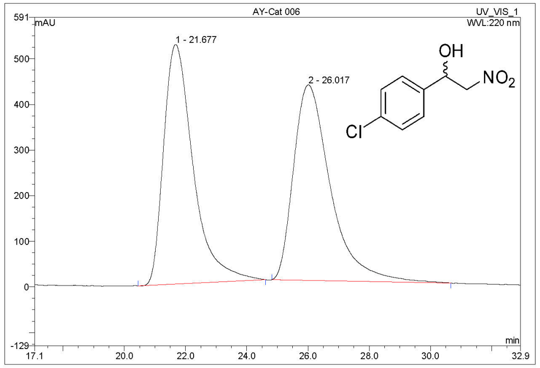

No.	Ret.Time min	Peak Name	Height mAU	Area mAU*min	Rel.Area %	Amount	Type
1	21.68	n.a.	524.784	588.703	49.55	n.a.	BMB*
2	26.02	n.a.	428.475	599.308	50.45	n.a.	BMB*
**Total:**			953.259	1188.012	100.00	0.000	

DEFAULT/Integration Chromeleon (c) Dionex 1996-2006
Version 6.80 SR15 Build 4656 (243203)

HPLC Trace (S)-**6d**

Operator:Shimadzu Timebase:LC_System2 Sequence:ISOCRATIC 95 5 Hexane IPA Page 1-1
11/12/2016 11:34 PM

80 AY-Cat 108

Sample Name:	**AY-Cat 108**	*Injection Volume:*	**5.0**
Vial Number:	**1_16**	*Channel:*	**UV_VIS_1**
Sample Type:	**unknown**	*Wavelength:*	**220**
Control Program:	**ISOCRATIC 95 5 Hexane IPA**	*Bandwidth:*	**n.a.**
Quantif. Method:	**ISOCRATIC 95 5 Hexane IPA**	*Dilution Factor:*	**1.0000**
Recording Time:	**6/12/2016 1:07**	*Sample Weight:*	**1.0000**
Run Time (min):	**50.11**	*Sample Amount:*	**1.0000**

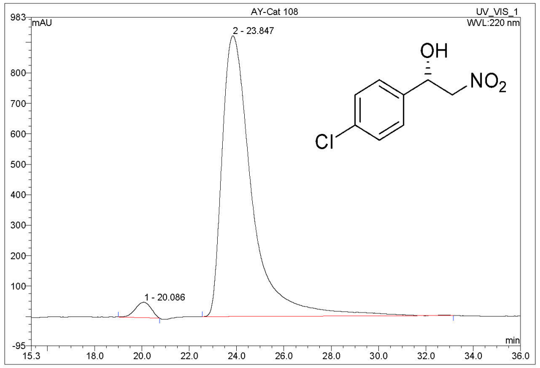

No.	Ret.Time min	Peak Name	Height mAU	Area mAU*min	Rel.Area %	Amount	Type
1	20.09	n.a.	50.721	39.141	2.82	n.a.	BMB*
2	23.85	n.a.	921.451	1347.089	97.18	n.a.	BMB*
**Total:**			972.171	1386.230	100.00	0.000	

DEFAULT/Integration Chromeleon (c) Dionex 1996-2006
Version 6.80 SR15 Build 4656 (243203)

HPLC Trace (rac)-***6e***

Operator:Shimadzu Timebase:LC_System2 Sequence:ISOCRATIC 95 5 Hexane IPA | Page 1-1 | 11/12/2016 11:41 PM

90 AY-Cat 037

Sample Name:	**AY-Cat 037**	*Injection Volume:*	**5.0**
Vial Number:	**1_5**	*Channel:*	**UV_VIS_2**
Sample Type:	**unknown**	*Wavelength:*	**230**
Control Program:	**ISOCRATIC 95 5 Hexane IPA**	*Bandwidth:*	**n.a.**
Quantif. Method:	**ISOCRATIC 95 5 Hexane IPA**	*Dilution Factor:*	**1.0000**
Recording Time:	**6/12/2016 11:32**	*Sample Weight:*	**1.0000**
Run Time (min):	**50.10**	*Sample Amount:*	**1.0000**

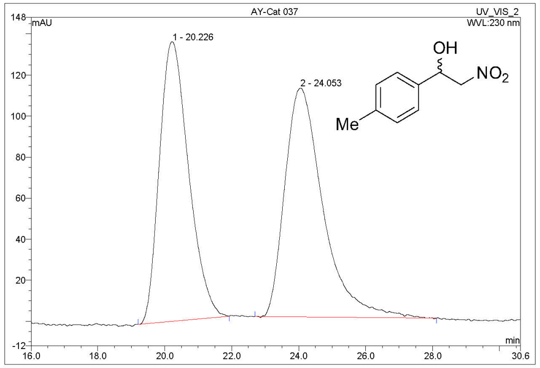

No.	Ret.Time min	Peak Name	Height mAU	Area mAU*min	Rel.Area %	Amount	Type
1	20.23	n.a.	136.175	137.915	48.39	n.a.	BMB*
2	24.05	n.a.	111.520	147.096	51.61	n.a.	BMB*
**Total:**			247.696	285.011	100.00	0.000	

DEFAULT/Integration

Chromeleon (c) Dionex 1996-2006
Version 6.80 SR15 Build 4656 (243203)

HPLC Trace (S)-***6e***

Operator:Shimadzu Timebase:LC_System2 Sequence:ISOCRATIC 95 5 Hexane IPA | Page 1-1 | 11/12/2016 11:42 PM

92 AY-Cat 114

Sample Name:	**AY-Cat 114**	*Injection Volume:*	**5.0**
Vial Number:	**1_7**	*Channel:*	**UV_VIS_2**
Sample Type:	**unknown**	*Wavelength:*	**230**
Control Program:	**ISOCRATIC 95 5 Hexane IPA**	*Bandwidth:*	**n.a.**
Quantif. Method:	**ISOCRATIC 95 5 Hexane IPA**	*Dilution Factor:*	**1.0000**
Recording Time:	**6/12/2016 13:13**	*Sample Weight:*	**1.0000**
Run Time (min):	**50.10**	*Sample Amount:*	**1.0000**

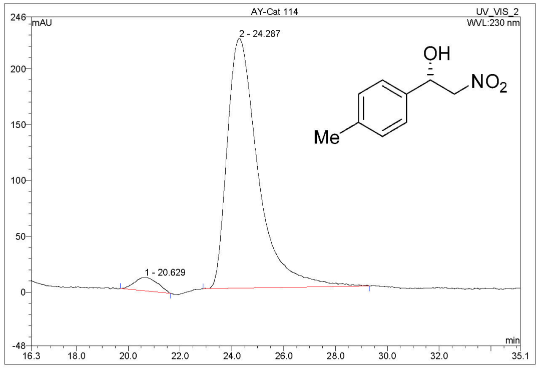

No.	Ret.Time min	Peak Name	Height mAU	Area mAU*min	Rel.Area %	Amount	Type
1	20.63	n.a.	12.016	12.469	3.87	n.a.	BMB*
2	24.29	n.a.	223.448	309.626	96.13	n.a.	BMB*
**Total:**			235.464	322.096	100.00	0.000	

DEFAULT/Integration

Chromeleon (c) Dionex 1996-2006
Version 6.80 SR15 Build 4656 (243203)

HPLC Trace (rac)-6f

Operator:Shimadzu Timebase:LC_System2 Sequence:ISOCRATIC 95 5 Hexane IPA

Page 1-1
28/1/2017 9:08 PM

66 AY-Cat 007

Sample Name:	**AY-Cat 007**	*Injection Volume:*	**3.0**
Vial Number:	**1_2**	*Channel:*	**UV_VIS_1**
Sample Type:	**unknown**	*Wavelength:*	**220**
Control Program:	**ISOCRATIC 95 5 Hexane IPA**	*Bandwidth:*	**n.a.**
Quantif. Method:	**ISOCRATIC 95 5 Hexane IPA**	*Dilution Factor:*	**1.0000**
Recording Time:	**5/12/2016 10:52**	*Sample Weight:*	**1.0000**
Run Time (min):	**50.10**	*Sample Amount:*	**1.0000**

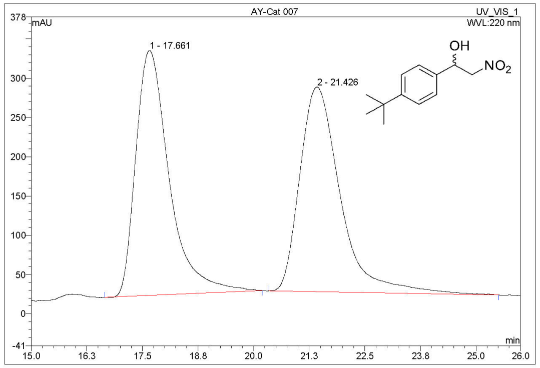

No.	Ret.Time min	Peak Name	Height mAU	Area mAU*min	Rel.Area %	Amount	Type
1	17.66	n.a.	311.867	271.678	49.75	n.a.	BMB*
2	21.43	n.a.	260.628	274.406	50.25	n.a.	BMB*
**Total:**			572.495	546.084	100.00	0.000	

DEFAULT/Integration

Chromeleon (c) Dionex 1996-2006
Version 6.80 SR15 Build 4656 (243203)

HPLC Trace (S)-6f

Operator:Shimadzu Timebase:LC_System2 Sequence:ISOCRATIC 95 5 Hexane IPA

Page 1-1
28/1/2017 9:19 PM

72 AY-Cat 121

Sample Name:	**AY-Cat 121**	*Injection Volume:*	**5.0**
Vial Number:	**1_3**	*Channel:*	**UV_VIS_1**
Sample Type:	**unknown**	*Wavelength:*	**220**
Control Program:	**ISOCRATIC 95 5 Hexane IPA**	*Bandwidth:*	**n.a.**
Quantif. Method:	**ISOCRATIC 95 5 Hexane IPA**	*Dilution Factor:*	**1.0000**
Recording Time:	**5/12/2016 15:57**	*Sample Weight:*	**1.0000**
Run Time (min):	**50.10**	*Sample Amount:*	**1.0000**

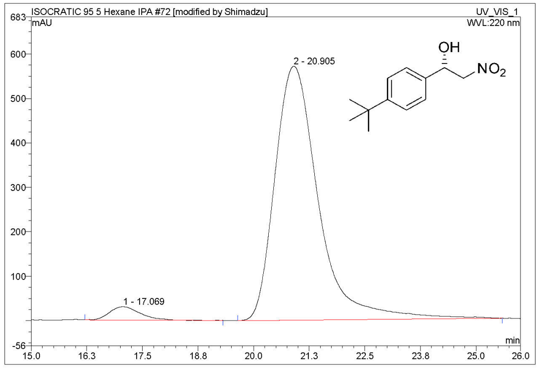

No.	Ret.Time min	Peak Name	Height mAU	Area mAU*min	Rel.Area %	Amount	Type
1	17.07	n.a.	29.932	23.466	3.49	n.a.	BMB*
2	20.91	n.a.	571.556	649.121	96.51	n.a.	BMB*
**Total:**			601.488	672.587	100.00	0.000	

DEFAULT/Integration

Chromeleon (c) Dionex 1996-2006
Version 6.80 SR15 Build 4656 (243203)

HPLC Trace (rac)-**6g**

Operator:Shimadzu Timebase:LC_System1 Sequence:15%MeCN v 1 — Page 1-1 — 3/12/2016 10:02 PM

38 AY-Cat 009

Sample Name:	**AY-Cat 009**	*Injection Volume:*	**5.0**
Vial Number:	**1_4**	*Channel:*	**UV_VIS_2**
Sample Type:	**unknown**	*Wavelength:*	**230**
Control Program:	**15% MeCN v 1**	*Bandwidth:*	**8**
Quantif. Method:	**15% MeCN v 1**	*Dilution Factor:*	**1.0000**
Recording Time:	**3/12/2016 20:14**	*Sample Weight:*	**1.0000**
Run Time (min):	**45.01**	*Sample Amount:*	**1.0000**

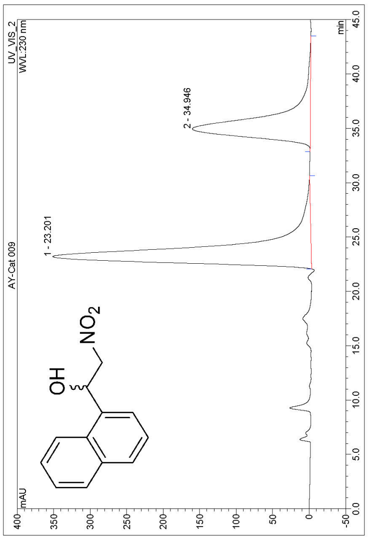

No.	Ret.Time min	Peak Name	Height mAU	Area mAU*min	Rel.Area %	Amount	Type
1	23.20	n.a.	354.215	576.358	63.74	n.a.	BMB*
2	34.95	n.a.	161.552	327.910	36.26	n.a.	BMB*
**Total:**			515.767	904.268	100.00	0.000	

DEFAULT/Integration — Chromeleon (c) Dionex 1996-2006 Version 6.80 SR15 Build 4656 (243203)

HPLC Trace (S)-**6g**

Operator:Shimadzu Timebase:LC_System1 Sequence:15%MeCN v 1 — Page 1-1 — 3/12/2016 10:04 PM

39 AY-Cat 109

Sample Name:	**AY-Cat 109**	*Injection Volume:*	**5.0**
Vial Number:	**1_5**	*Channel:*	**UV_VIS_2**
Sample Type:	**unknown**	*Wavelength:*	**230**
Control Program:	**15% MeCN v 1**	*Bandwidth:*	**8**
Quantif. Method:	**15% MeCN v 1**	*Dilution Factor:*	**1.0000**
Recording Time:	**3/12/2016 21:00**	*Sample Weight:*	**1.0000**
Run Time (min):	**45.01**	*Sample Amount:*	**1.0000**

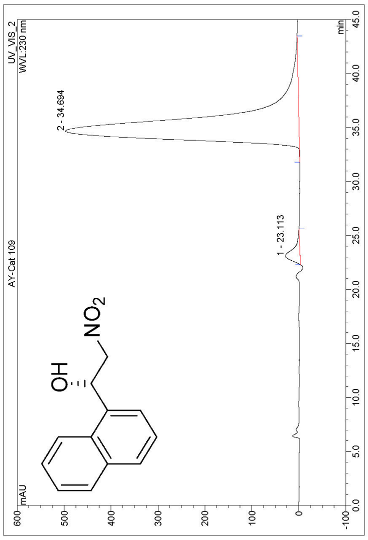

No.	Ret.Time min	Peak Name	Height mAU	Area mAU*min	Rel.Area %	Amount	Type
1	23.11	n.a.	31.190	38.363	3.34	n.a.	BMB*
2	34.69	n.a.	497.834	1111.088	96.66	n.a.	BMB*
**Total:**			529.024	1149.451	100.00	0.000	

DEFAULT/Integration — Chromeleon (c) Dionex 1996-2006 Version 6.80 SR15 Build 4656 (243203)

*HPLC Trace (rac)-**6h***

Operator:Shimadzu Timebase:LC_System2 Sequence:ISOCRATIC 95 5 Hexane IPA — Page 1-1 — 11/12/2016 6:17 PM

117 AY-Cat 038

Sample Name:	**AY-Cat 038**	*Injection Volume:*	**4.0**
Vial Number:	**1_11**	*Channel:*	**UV_VIS_1**
Sample Type:	**unknown**	*Wavelength:*	**220**
Control Program:	**ISOCRATIC 95 5 Hexane IPA**	*Bandwidth:*	**n.a.**
Quantif. Method:	**ISOCRATIC 95 5 Hexane IPA**	*Dilution Factor:*	**1.0000**
Recording Time:	**11/12/2016 14:49**	*Sample Weight:*	**1.0000**
Run Time (min):	**40.10**	*Sample Amount:*	**1.0000**

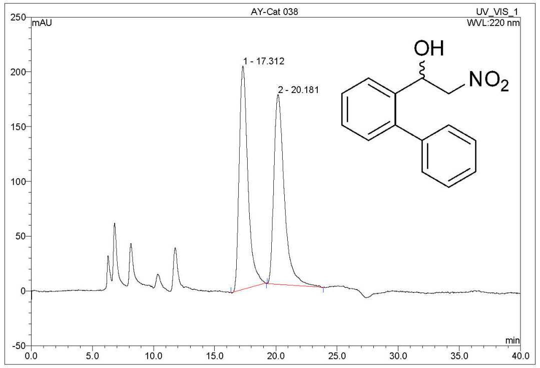

No.	Ret.Time min	Peak Name	Height mAU	Area mAU*min	Rel.Area %	Amount	Type
1	17.31	n.a.	204.003	167.974	49.57	n.a.	BMB*
2	20.18	n.a.	173.277	170.888	50.43	n.a.	BMB*
**Total:**			377.281	338.862	100.00	0.000	

DEFAULT/Integration — Chromeleon (c) Dionex 1996-2006 Version 6.80 SR15 Build 4656 (243203)

*HPLC Trace (S)-**6h***

Operator:Shimadzu Timebase:LC_System2 Sequence:ISOCRATIC 95 5 Hexane IPA — Page 1-1 — 11/12/2016 6:19 PM

118 AY-Cat 199

Sample Name:	**AY-Cat 199**	*Injection Volume:*	**4.0**
Vial Number:	**1_12**	*Channel:*	**UV_VIS_1**
Sample Type:	**unknown**	*Wavelength:*	**220**
Control Program:	**ISOCRATIC 95 5 Hexane IPA**	*Bandwidth:*	**n.a.**
Quantif. Method:	**ISOCRATIC 95 5 Hexane IPA**	*Dilution Factor:*	**1.0000**
Recording Time:	**11/12/2016 15:29**	*Sample Weight:*	**1.0000**
Run Time (min):	**40.10**	*Sample Amount:*	**1.0000**

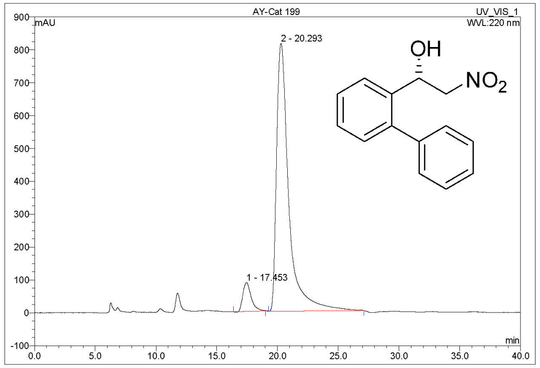

No.	Ret.Time min	Peak Name	Height mAU	Area mAU*min	Rel.Area %	Amount	Type
1	17.45	n.a.	87.555	69.369	6.79	n.a.	BMB*
2	20.29	n.a.	815.309	951.710	93.21	n.a.	BMB*
**Total:**			902.864	1021.080	100.00	0.000	

DEFAULT/Integration — Chromeleon (c) Dionex 1996-2006 Version 6.80 SR15 Build 4656 (243203)

*HPLC Trace (rac)-**6i***

Operator:Shimadzu Timebase:LC_System2 Sequence:ISOCRATIC 95 5 Hexane IPA — Page 1-1, 28/1/2017 10:05 PM

125 AY-Cat 011

Sample Name:	**AY-Cat 011**	*Injection Volume:*	**5.0**
Vial Number:	**1_2**	*Channel:*	**UV_VIS_1**
Sample Type:	**unknown**	*Wavelength:*	**220**
Control Program:	**ISOCRATIC 95 5 Hexane IPA**	*Bandwidth:*	**n.a.**
Quantif. Method:	**ISOCRATIC 95 5 Hexane IPA**	*Dilution Factor:*	**1.0000**
Recording Time:	**14/12/2016 12:54**	*Sample Weight:*	**1.0000**
Run Time (min):	**50.10**	*Sample Amount:*	**1.0000**

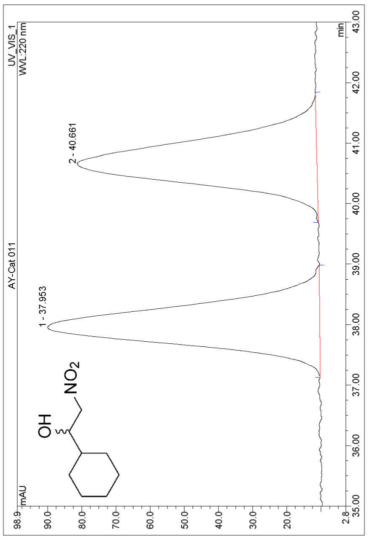

No.	Ret.Time min	Peak Name	Height mAU	Area mAU*min	Rel.Area %	Amount	Type
1	37.95	n.a.	79.594	54.986	50.27	n.a.	BMB*
2	40.66	n.a.	70.083	54.406	49.73	n.a.	BMB*
**Total:**			149.676	109.392	100.00	0.000	

DEFAULT/Integration — Chromeleon (c) Dionex 1996-2006 Version 6.80 SR15 Build 4656 (243203)

*HPLC Trace (S)-**6i***

Operator:Shimadzu Timebase:LC_System2 Sequence:ISOCRATIC 95 5 Hexane IPA — Page 1-1, 28/1/2017 10:02 PM

134 AY-Cat 118

Sample Name:	**AY-Cat 118**	*Injection Volume:*	**5.0**
Vial Number:	**1_4**	*Channel:*	**UV_VIS_1**
Sample Type:	**unknown**	*Wavelength:*	**220**
Control Program:	**ISOCRATIC 95 5 Hexane IPA**	*Bandwidth:*	**n.a.**
Quantif. Method:	**ISOCRATIC 95 5 Hexane IPA**	*Dilution Factor:*	**1.0000**
Recording Time:	**15/12/2016 16:46**	*Sample Weight:*	**1.0000**
Run Time (min):	**52.46**	*Sample Amount:*	**1.0000**

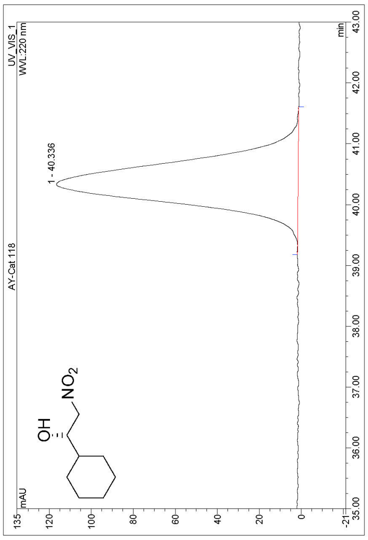

No.	Ret.Time min	Peak Name	Height mAU	Area mAU*min	Rel.Area %	Amount	Type
1	40.34	n.a.	115.062	84.429	100.00	n.a.	BMB*
**Total:**			115.062	84.429	100.00	0.000	

DEFAULT/Integration — Chromeleon (c) Dionex 1996-2006 Version 6.80 SR15 Build 4656 (243203)

*HPLC Trace (rac)-**6j***

Operator:Shimadzu Timebase:LC_System2 Sequence:ISOCRATIC 95 5 Hexane IPA Page 1-1 12/10/2017 7:25 PM

116 AY-Cat 032

Sample Name:	**AY-Cat 032**	*Injection Volume:*	**5.0**
Vial Number:	**1_2**	*Channel:*	**UV_VIS_1**
Sample Type:	**unknown**	*Wavelength:*	**210**
Control Program:	**ISOCRATIC 95 5 Hexane IPA**	*Bandwidth:*	**n.a.**
Quantif. Method:	**ISOCRATIC 95 5 Hexane IPA**	*Dilution Factor:*	**1.0000**
Recording Time:	**11/12/2016 19:39**	*Sample Weight:*	**1.0000**
Run Time (min):	**40.10**	*Sample Amount:*	**1.0000**

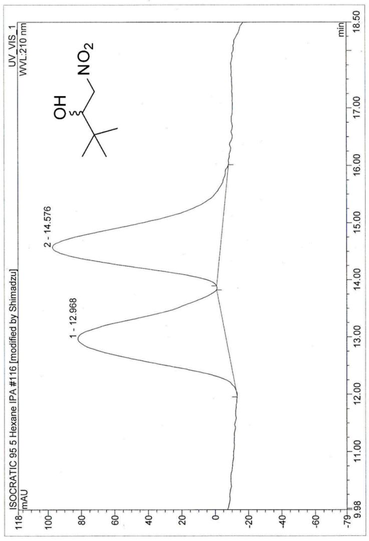

No.	Ret.Time min	Peak Name	Height mAU	Area mAU*min	Rel.Area %	Amount	Type
1	12.97	n.a.	88.463	75.196	48.15	n.a.	BMB*
2	14.58	n.a.	100.389	80.966	51.85	n.a.	BMB*
Total:			188.852	156.162	100.00	0.000	

*HPLC Trace (S)-**6j***

Operator:Shimadzu Timebase:LC_System2 Sequence:ISOCRATIC 95 5 Hexane IPA Page 1-1 12/10/2017 7:29 PM

117 AY-Cat 116

Sample Name:	**AY-Cat 116**	*Injection Volume:*	**5.0**
Vial Number:	**1_4**	*Channel:*	**UV_VIS_1**
Sample Type:	**unknown**	*Wavelength:*	**210**
Control Program:	**ISOCRATIC 95 5 Hexane IPA**	*Bandwidth:*	**n.a.**
Quantif. Method:	**ISOCRATIC 95 5 Hexane IPA**	*Dilution Factor:*	**1.0000**
Recording Time:	**11/12/2016 20:20**	*Sample Weight:*	**1.0000**
Run Time (min):	**26.51**	*Sample Amount:*	**1.0000**

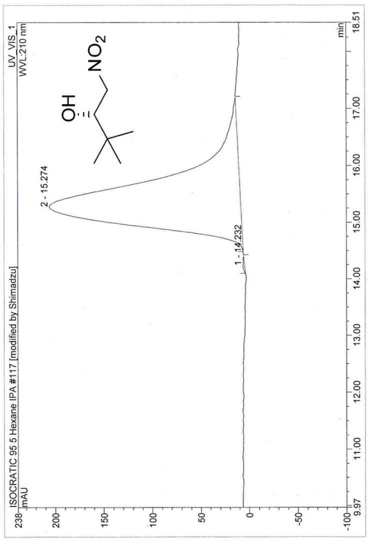

No.	Ret.Time min	Peak Name	Height mAU	Area mAU*min	Rel.Area %	Amount	Type
1	14.23	n.a.	1.679	0.313	0.18	n.a.	BMB*
2	15.27	n.a.	197.191	172.132	99.82	n.a.	BMB*
Total:			198.870	172.445	100.00	0.000	

NMR Spectrums

Proton NMR Spectrum of ***S1a***

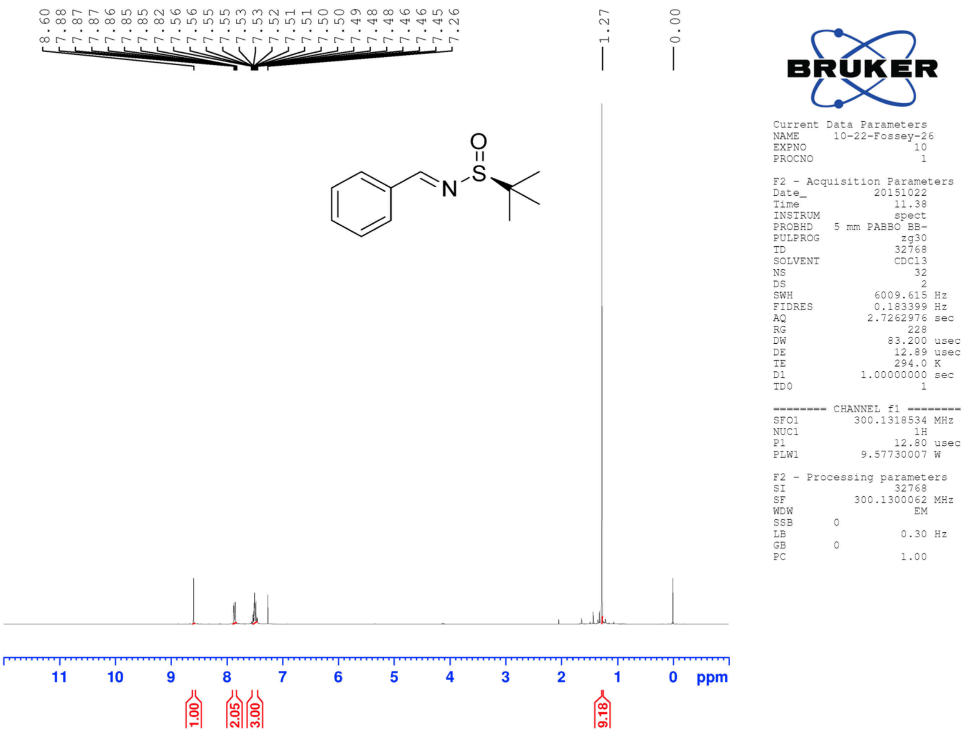

Carbon NMR Spectrum of ***S1a***

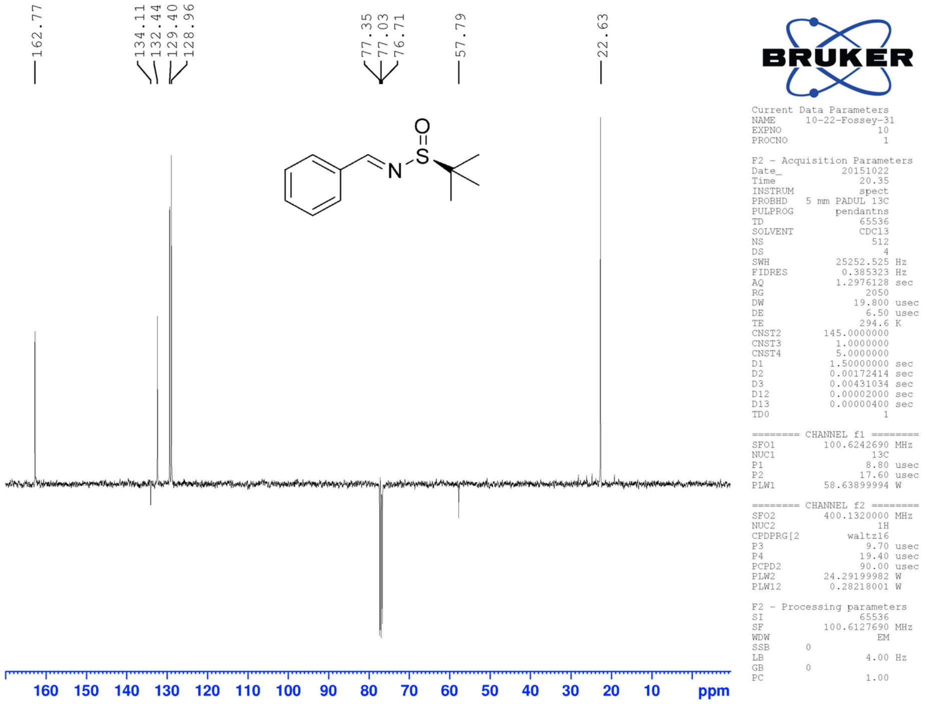

Proton NMR Spectrum of ***S1b***

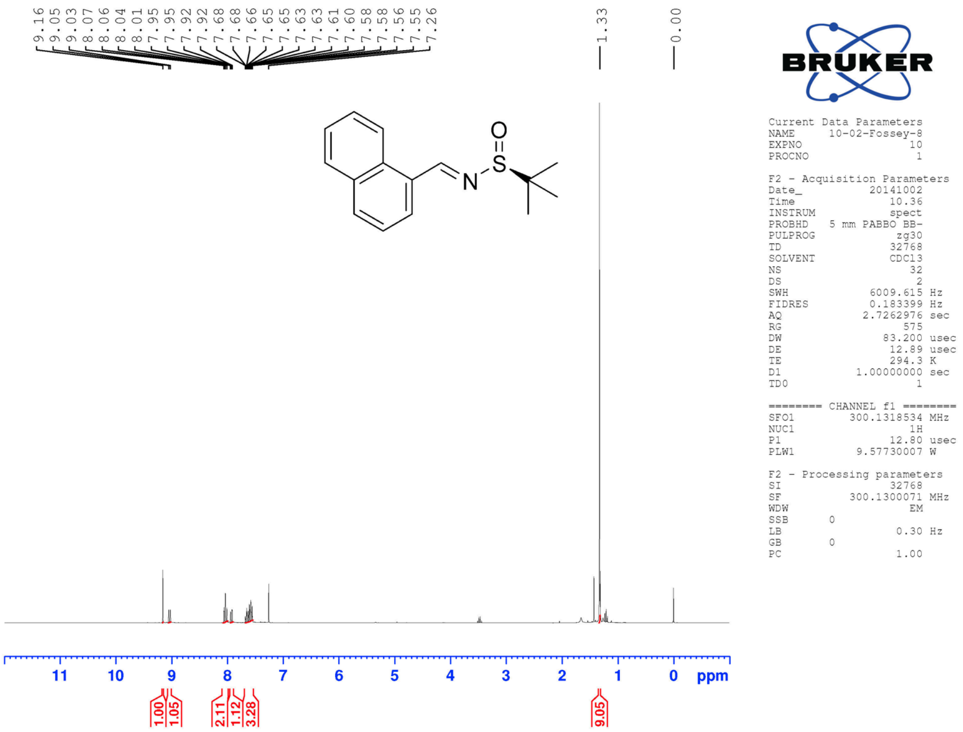

Carbon NMR Spectrum of ***S1b***

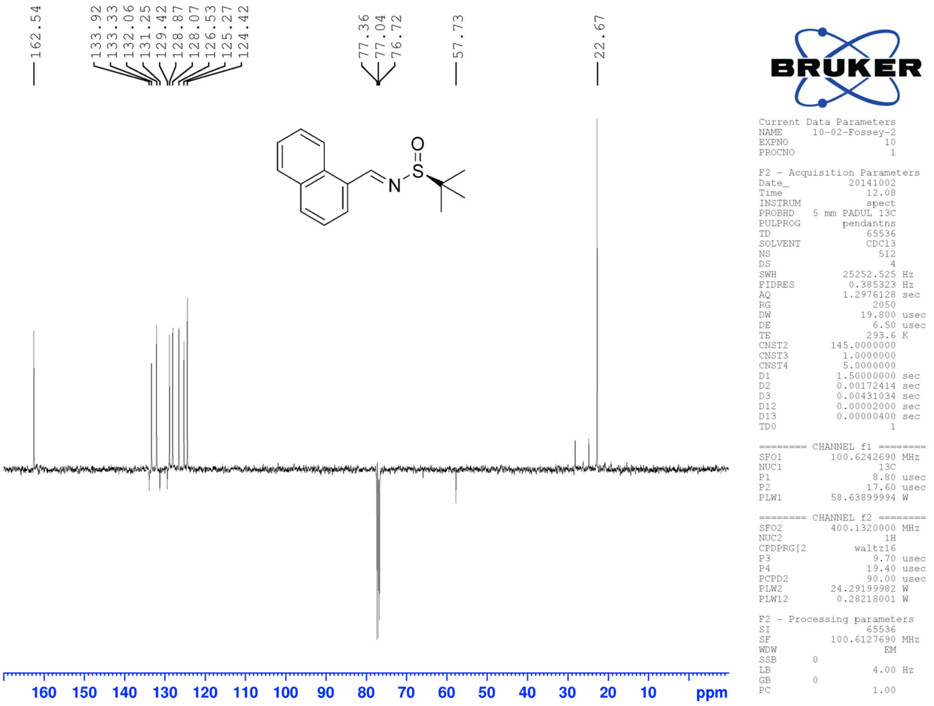

*Proton NMR Spectrum of **S1c***

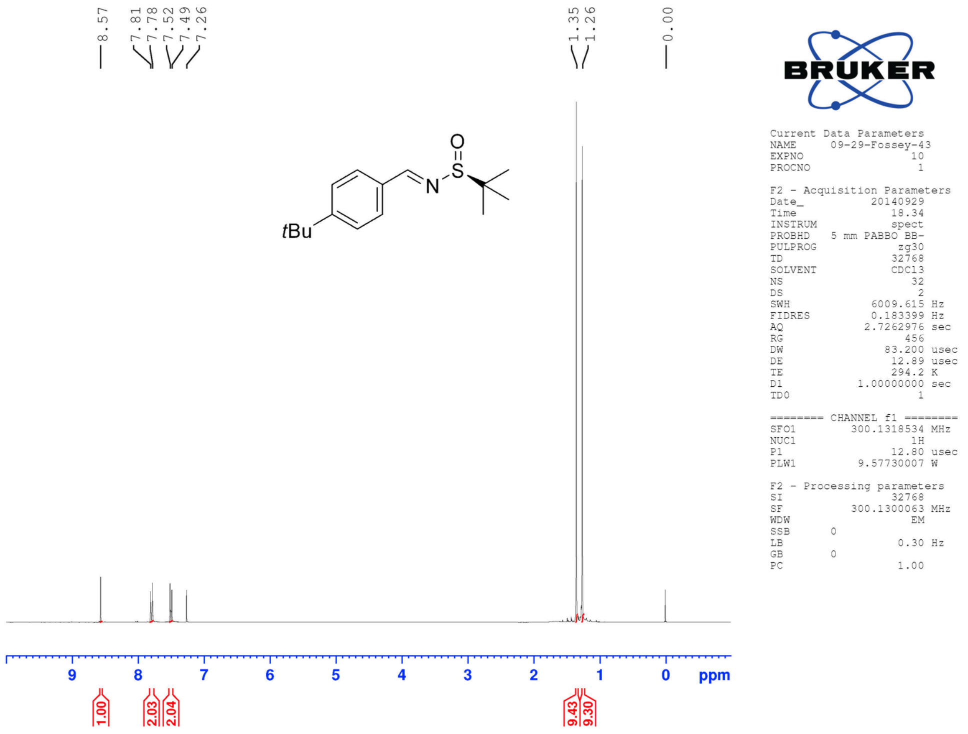

*Carbon NMR Spectrum of **S1c***

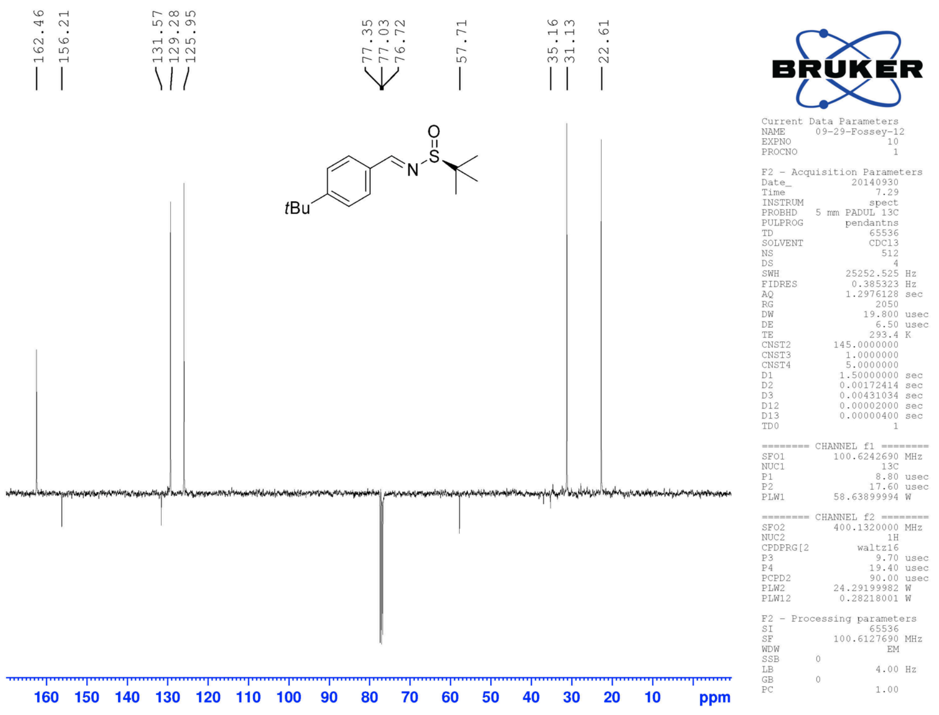

*Proton NMR Spectrum of **S1d***

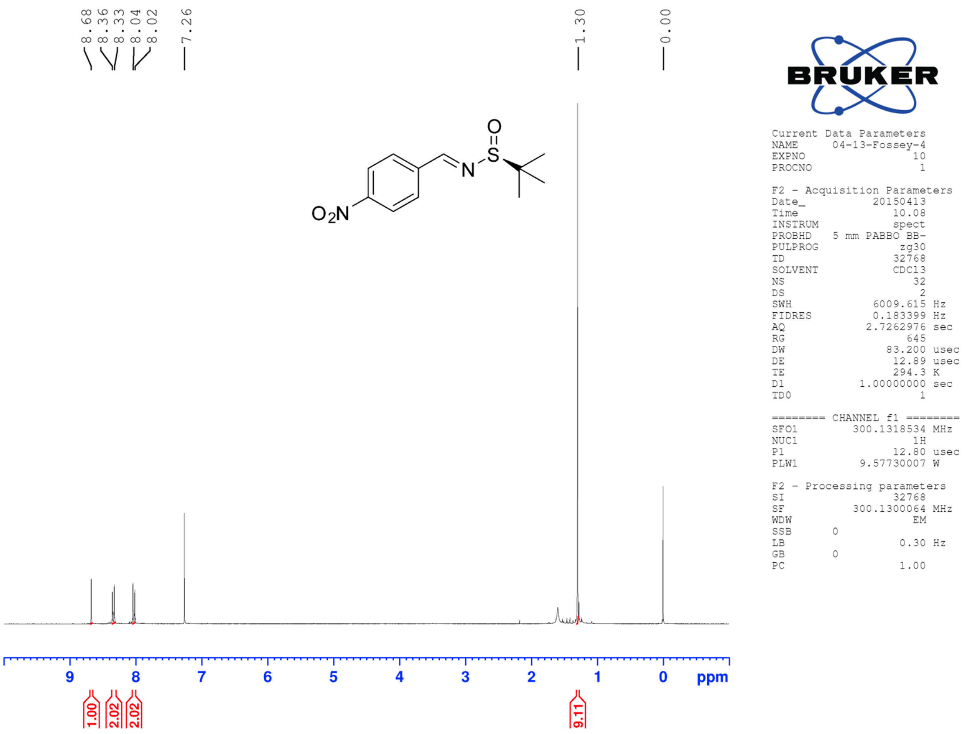

*Carbon NMR Spectrum of **S1d***

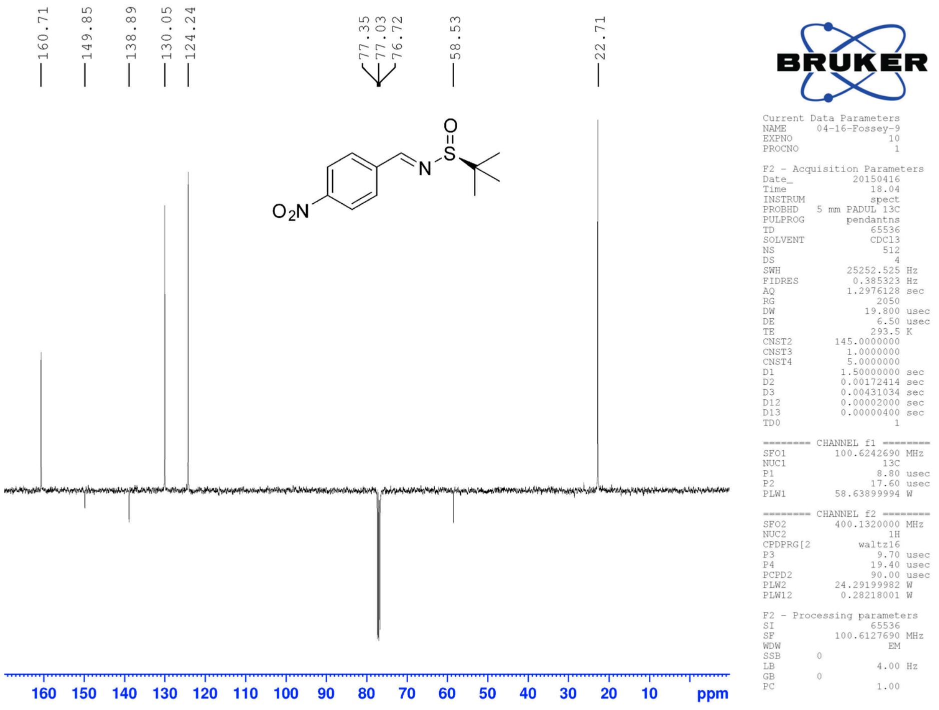

Proton NMR Spectrum of ***S1e***

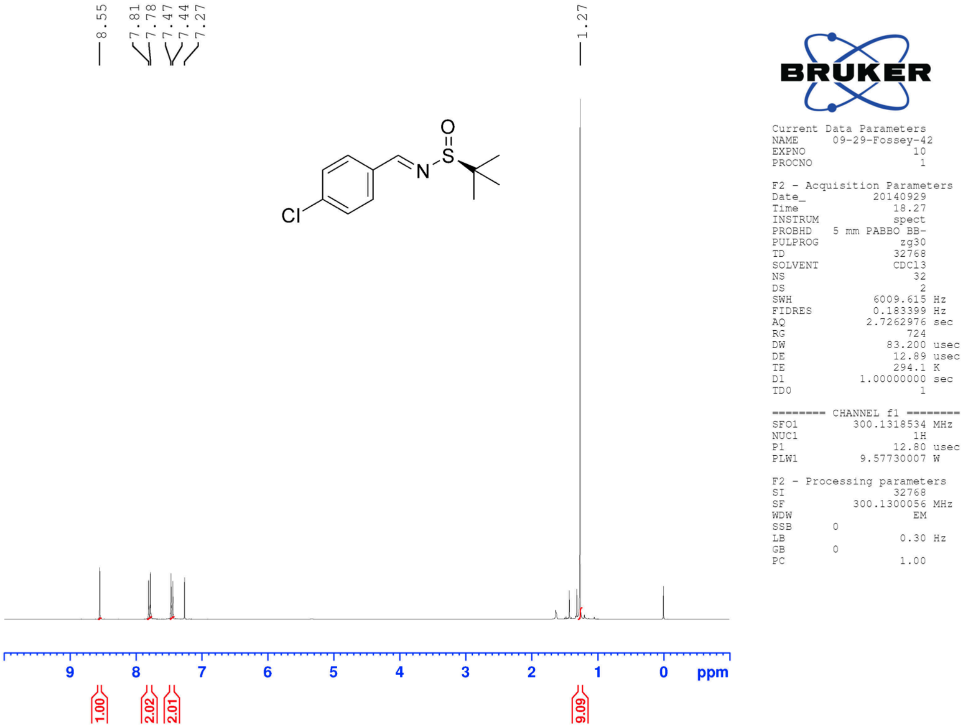

Carbon NMR Spectrum of ***S1e***

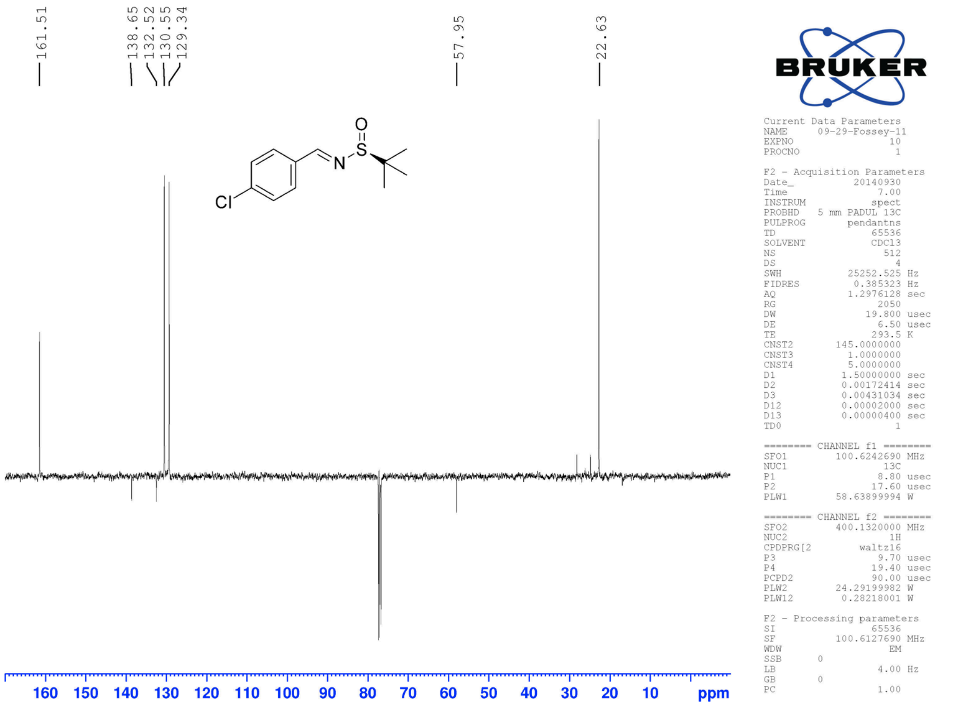

*Proton NMR Spectrum of **S2a***

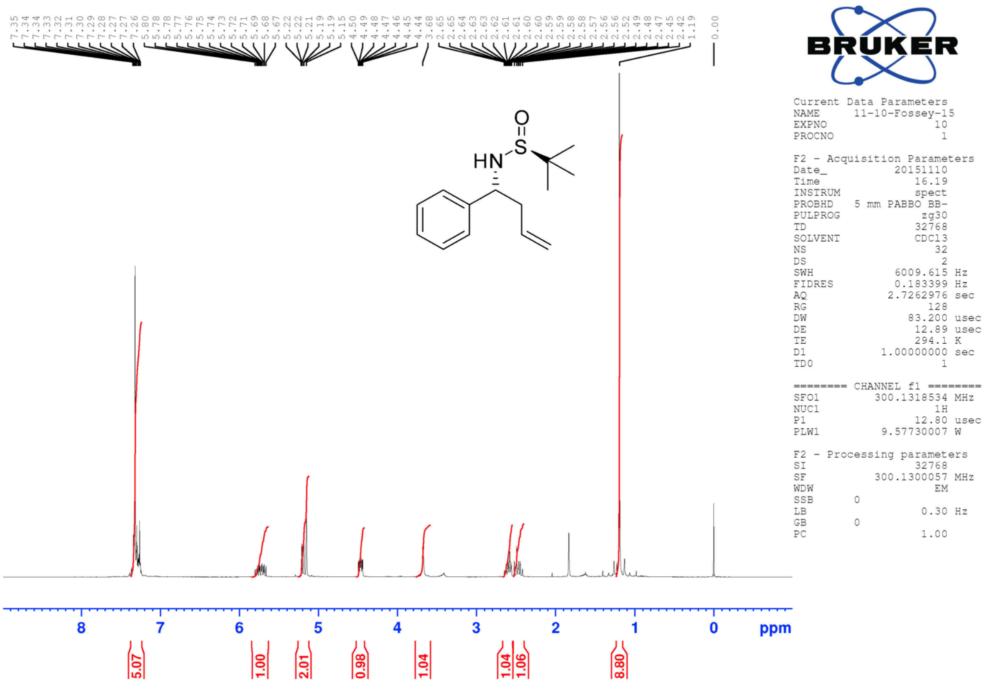

*Carbon NMR Spectrum of **S2a***

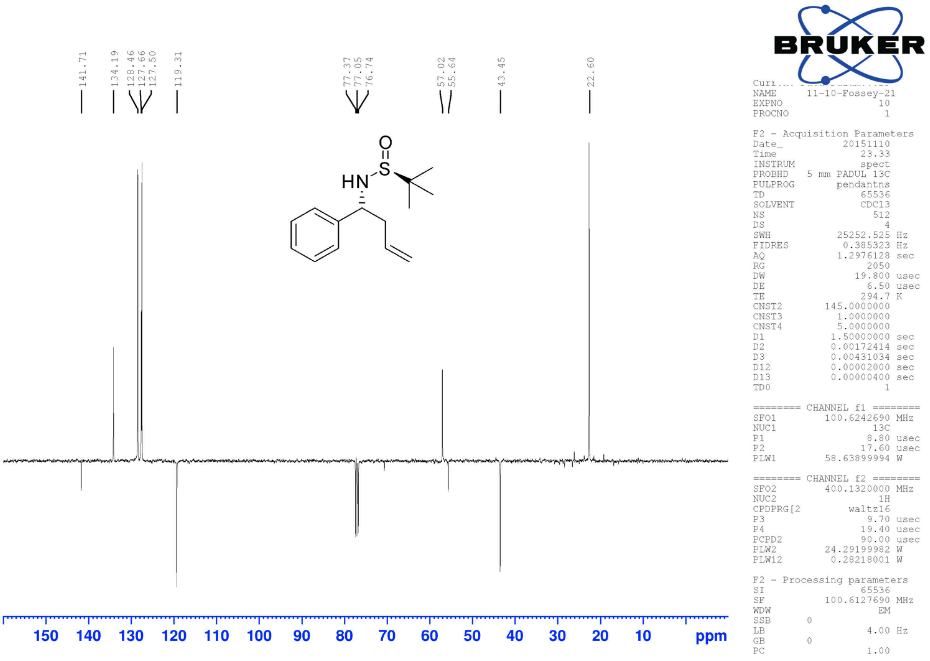

*Proton NMR Spectrum of **S2b***

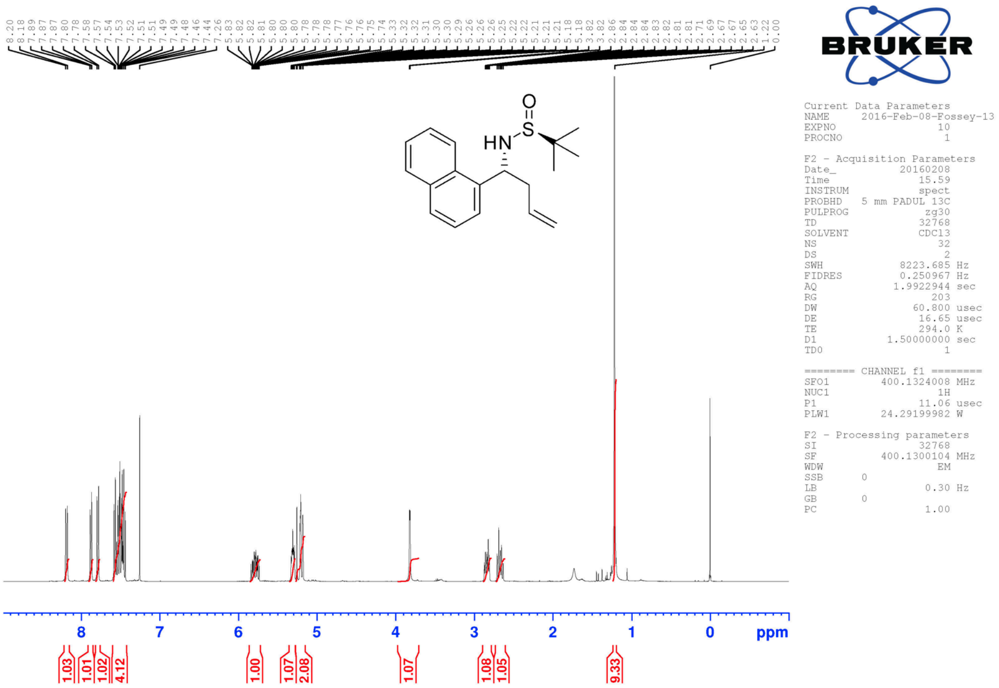

*Carbon NMR Spectrum of **S2b***

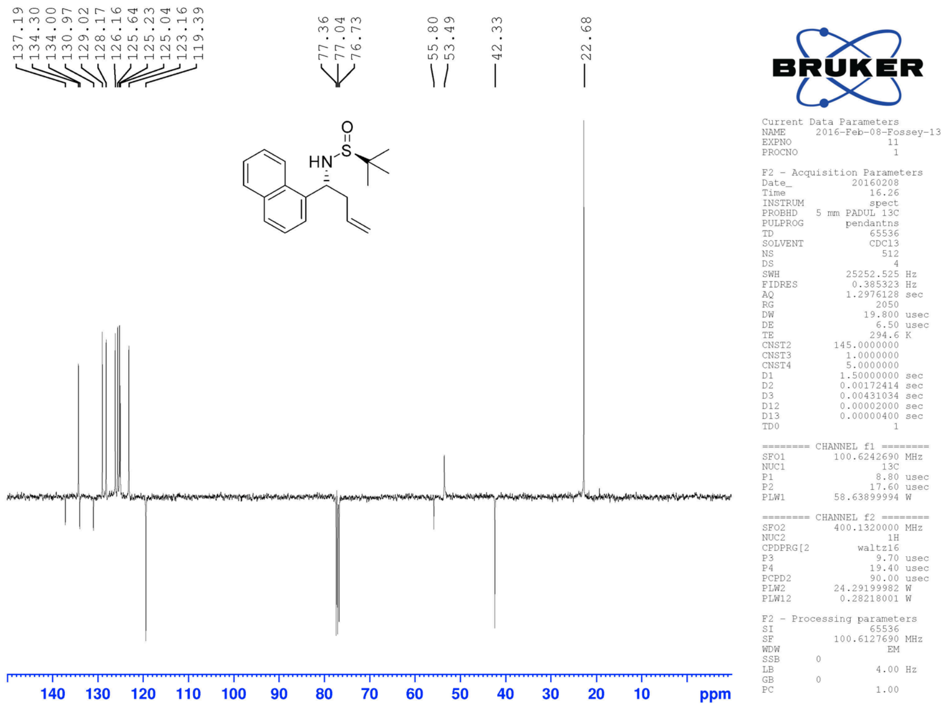

Proton NMR Spectrum of ***S2c***

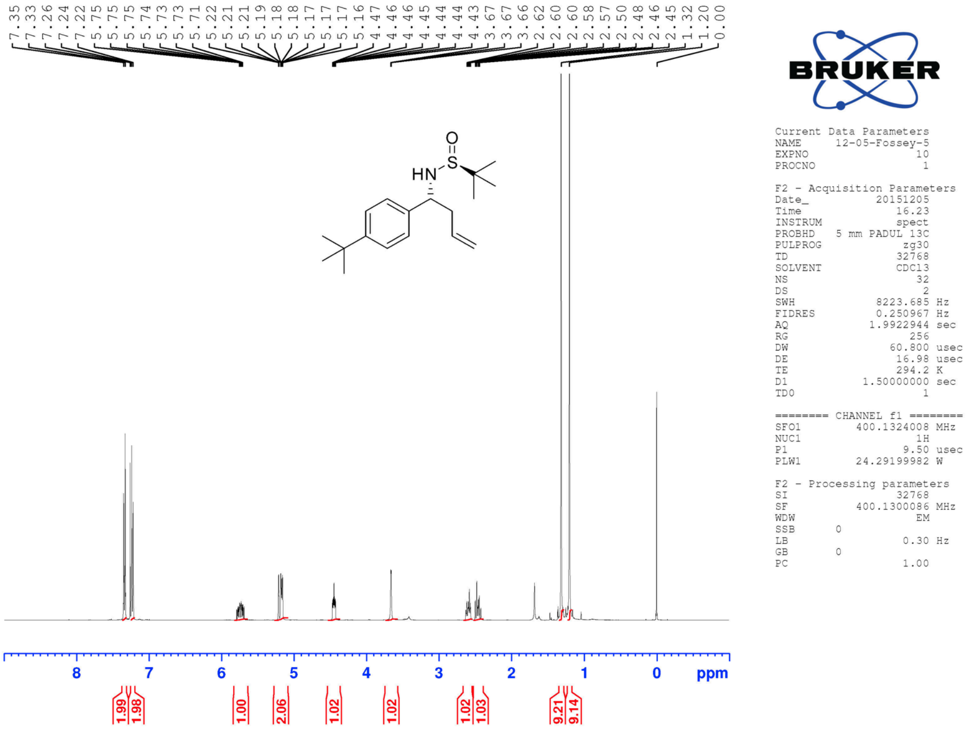

Carbon NMR Spectrum of ***S2c***

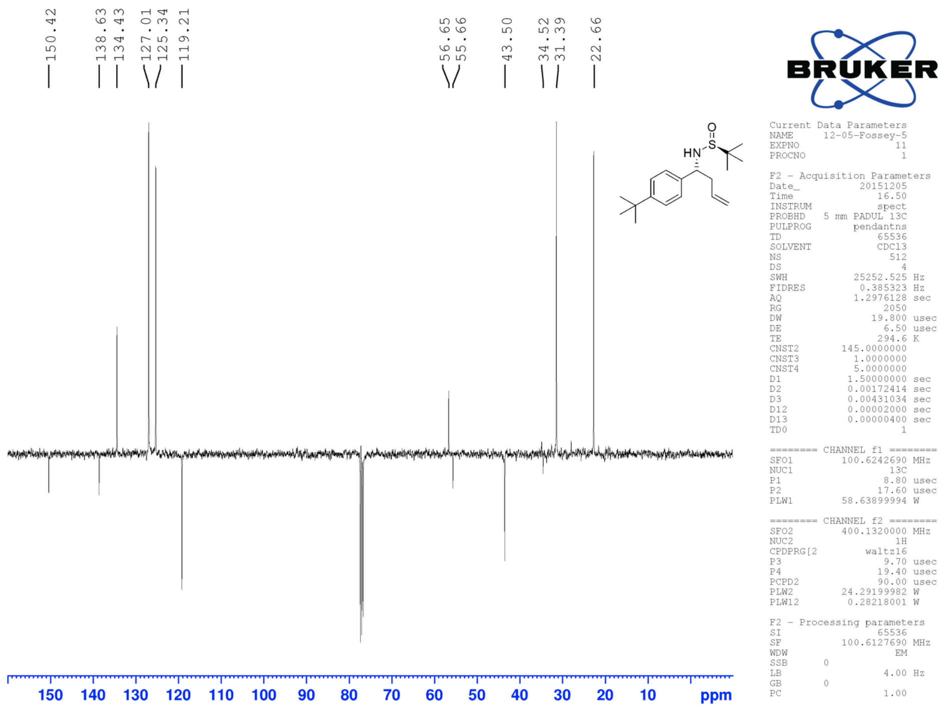

Proton NMR Spectrum of ***S2d***

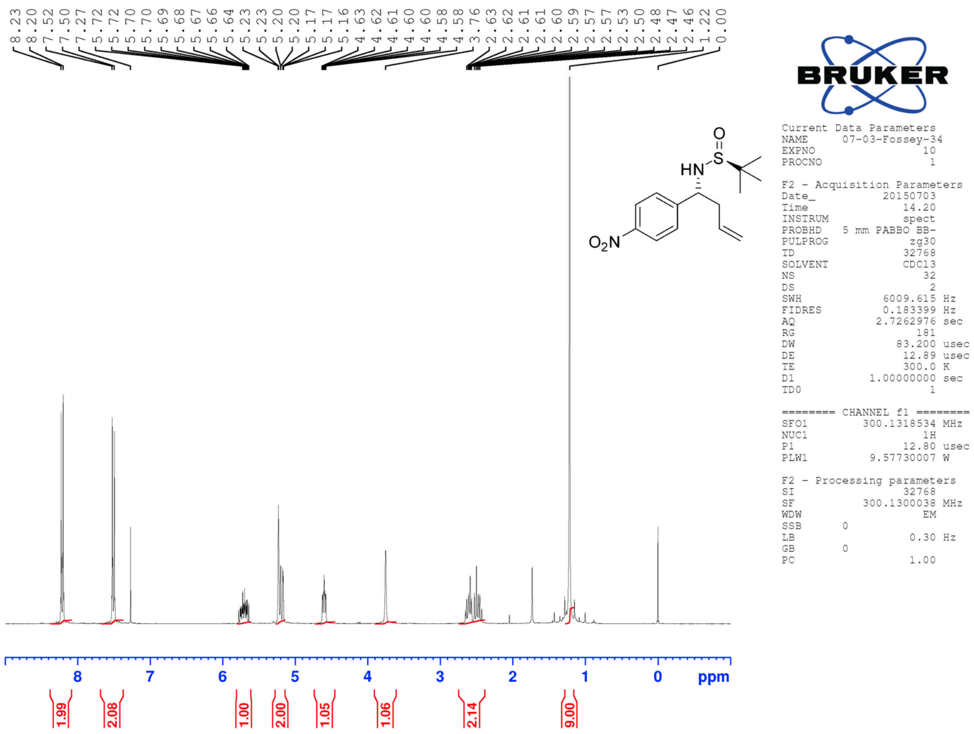

Carbon NMR Spectrum of ***S2d***

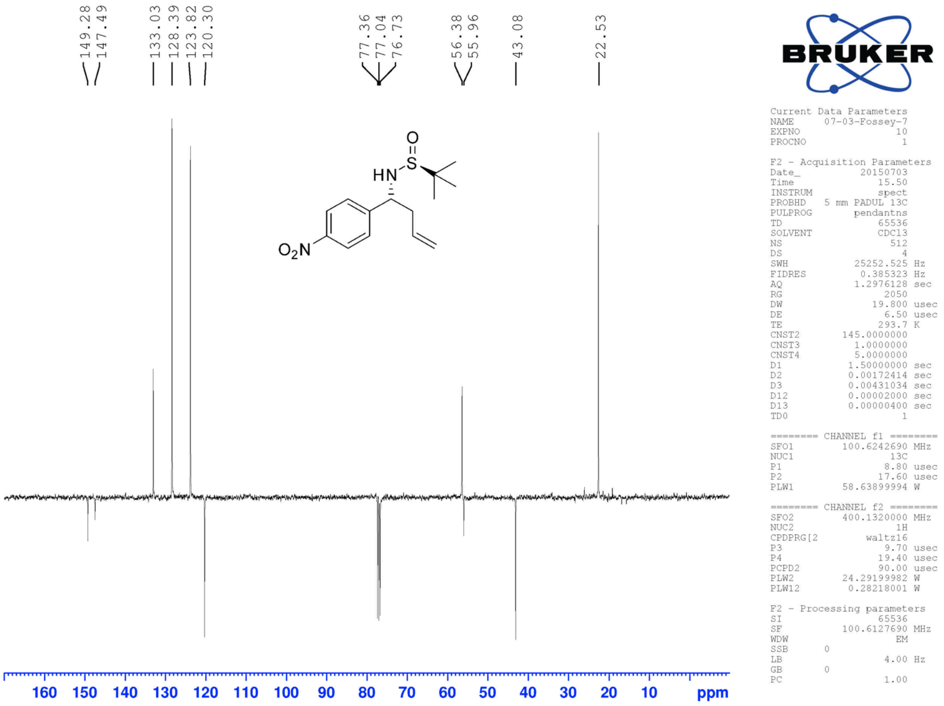

Proton NMR Spectrum of ***S2e***

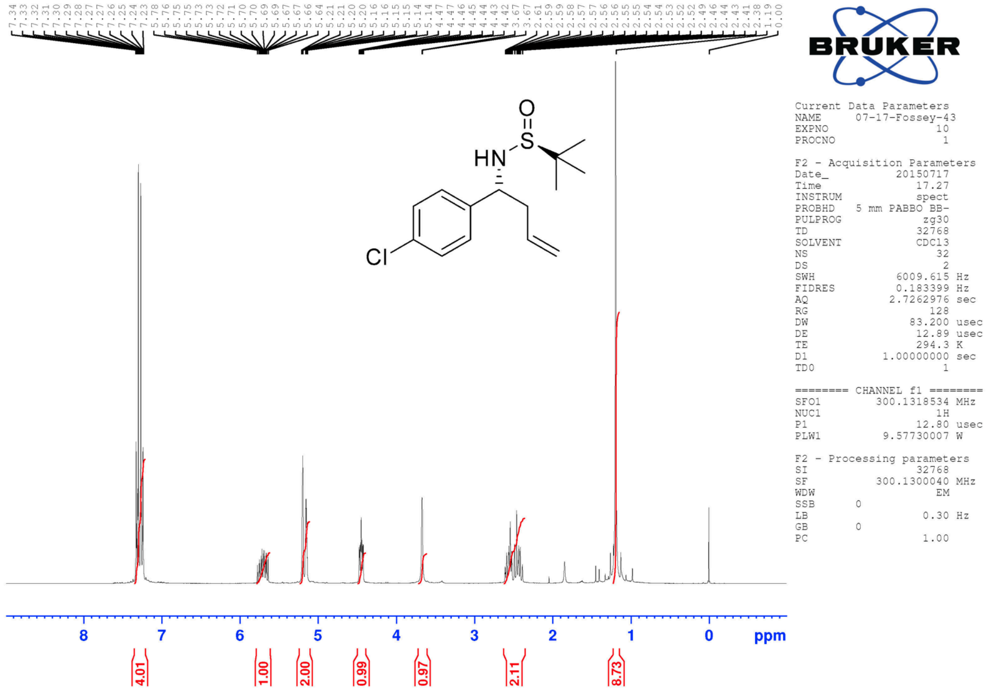

Carbon NMR Spectrum of ***S2e***

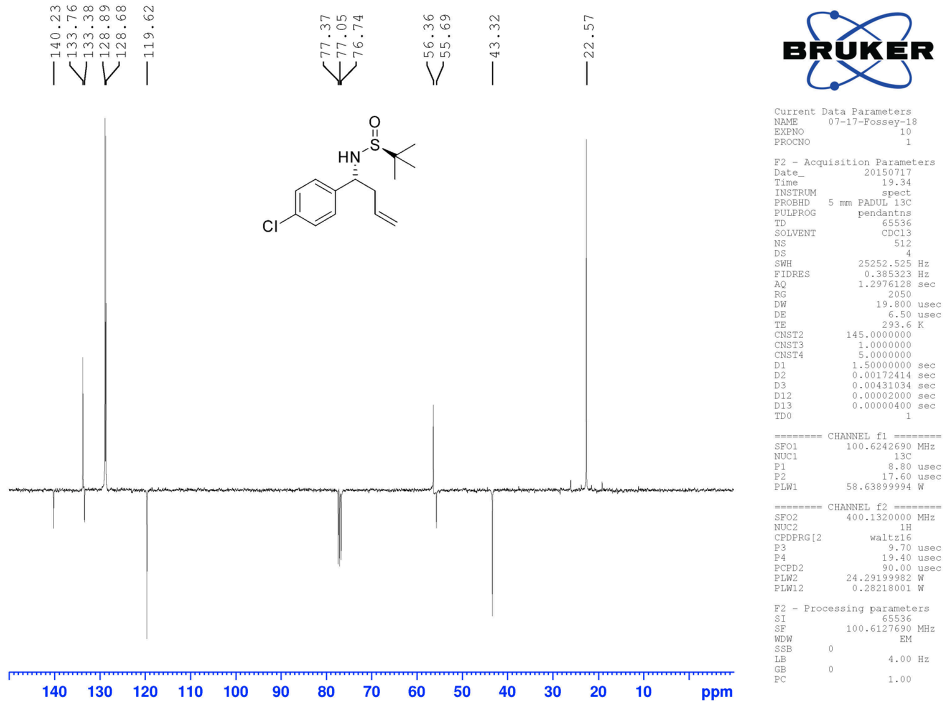

Proton NMR Spectrum of ***S3a***

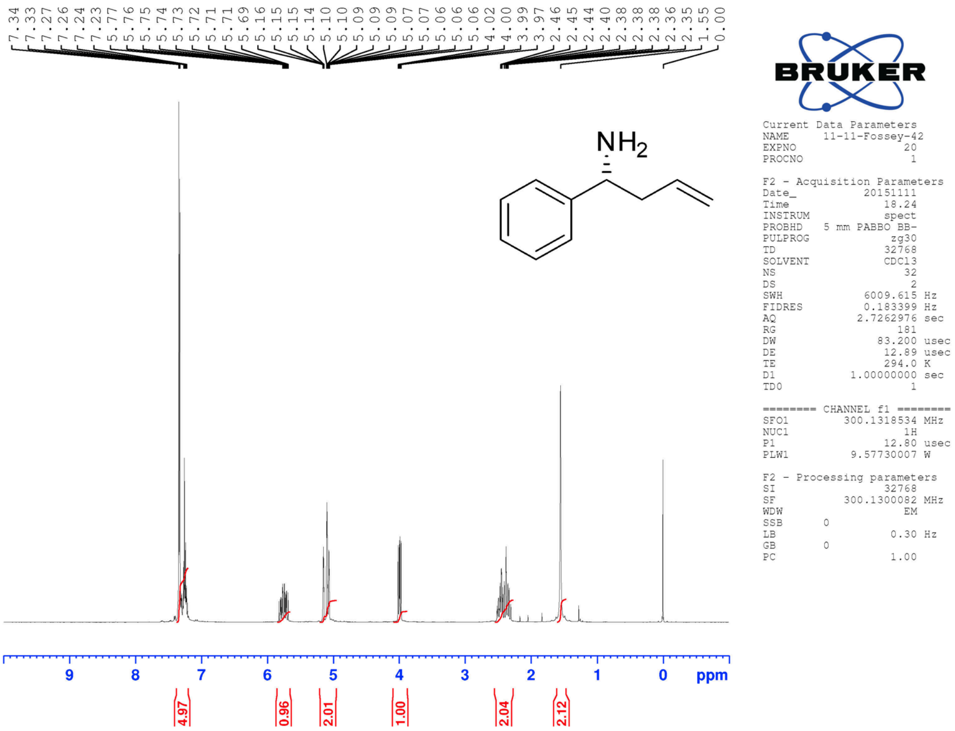

Carbon NMR Spectrum of ***S3a***

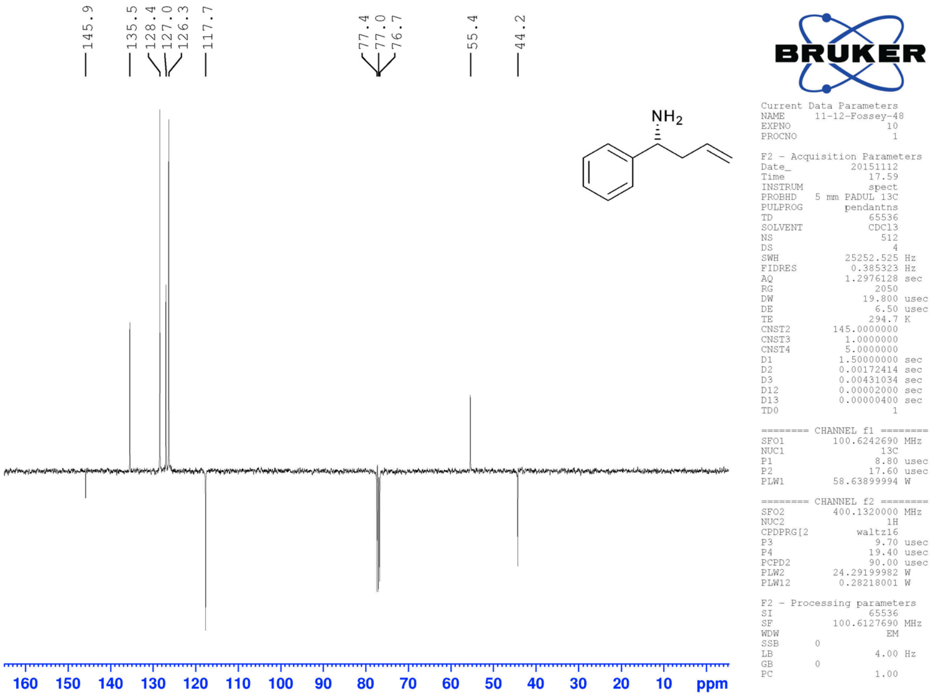

Proton NMR Spectrum of ***S3b***

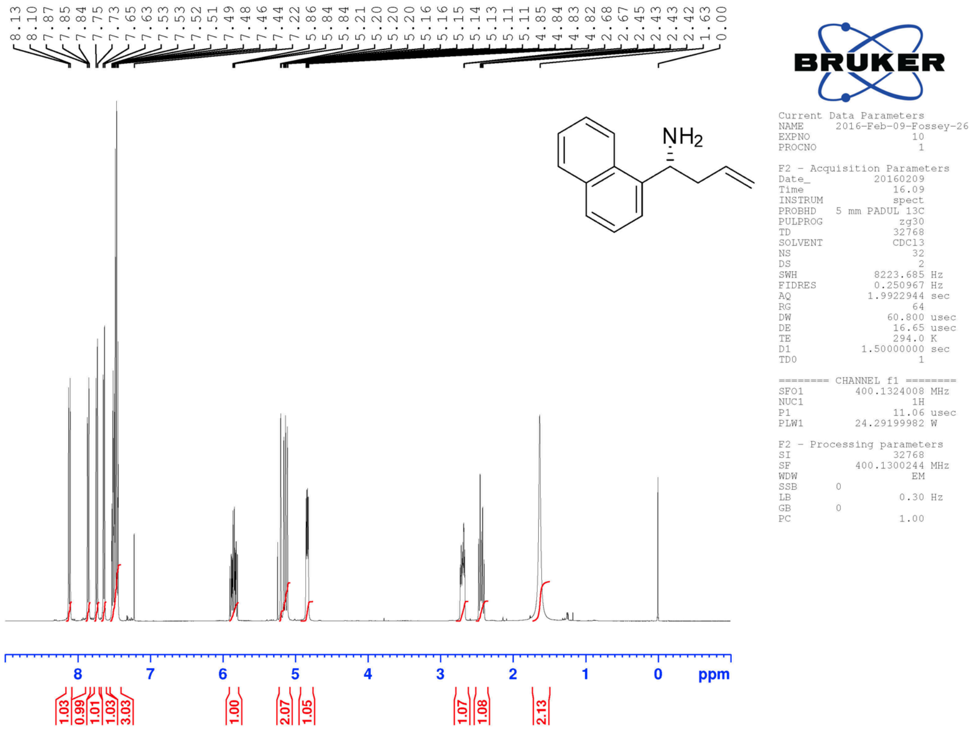

Carbon NMR Spectrum of ***S3b***

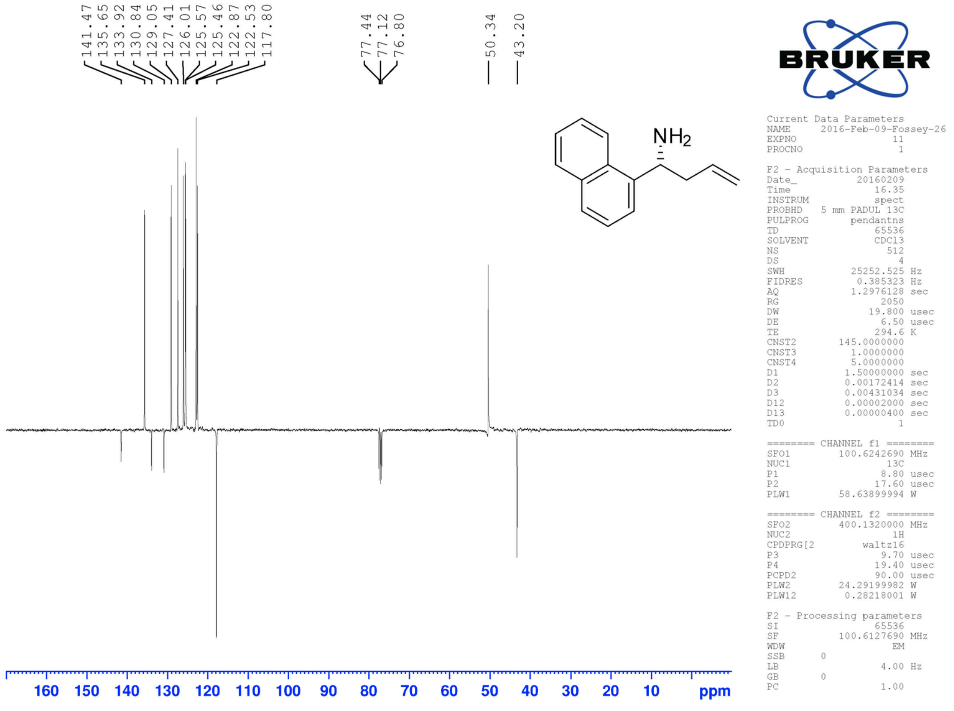

Proton NMR Spectrum of ***S3c***

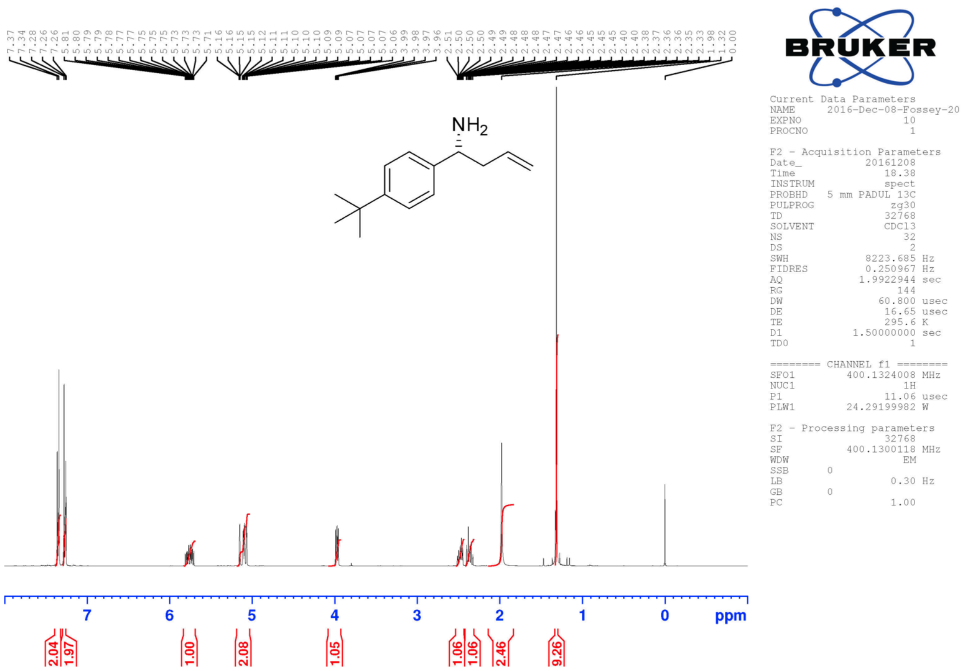

Carbon NMR Spectrum of ***S3c***

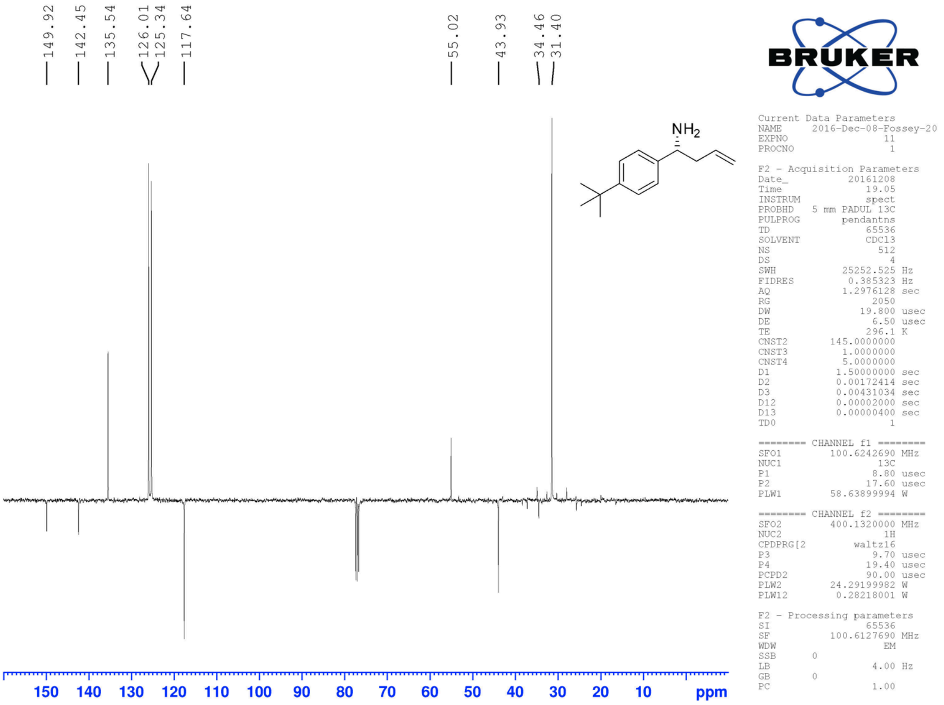

*Proton NMR Spectrum of **S3d***

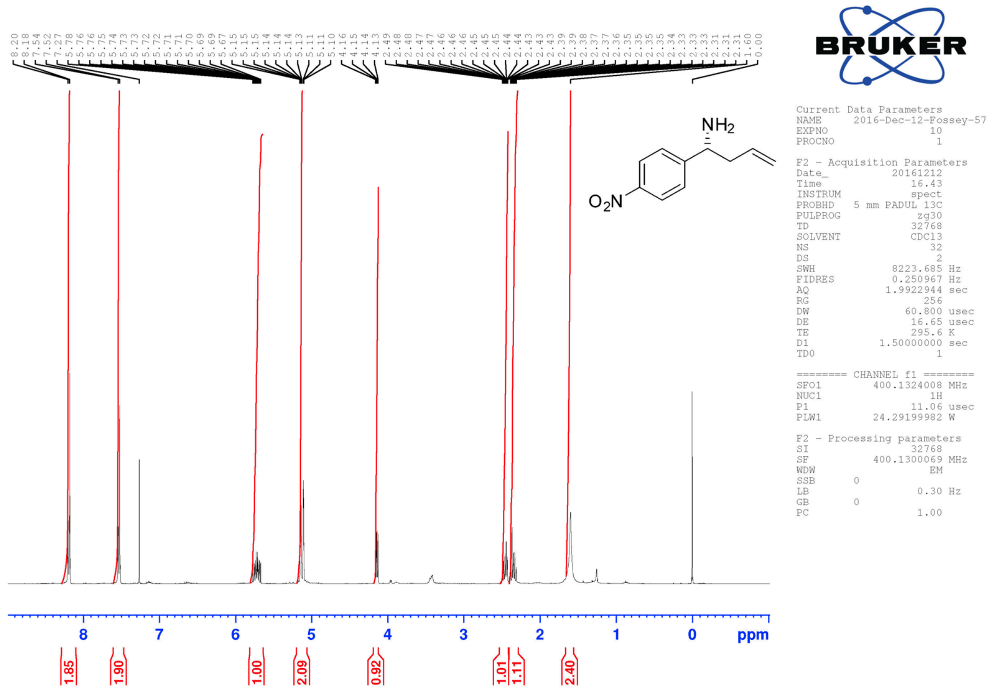

*Carbon NMR Spectrum of **S3d***

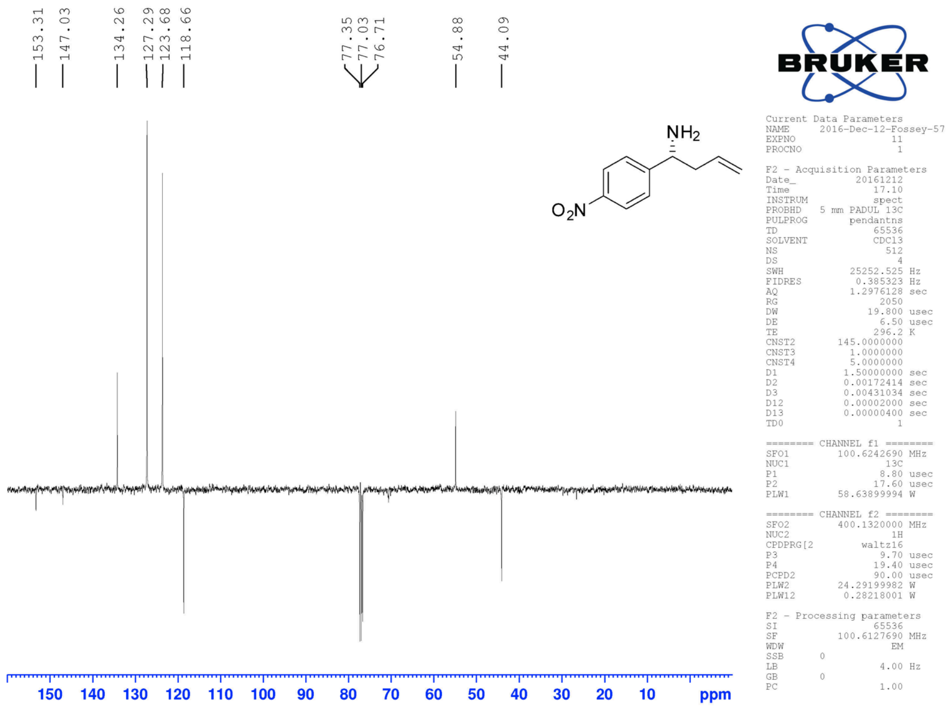

*Proton NMR Spectrum of **S3e***

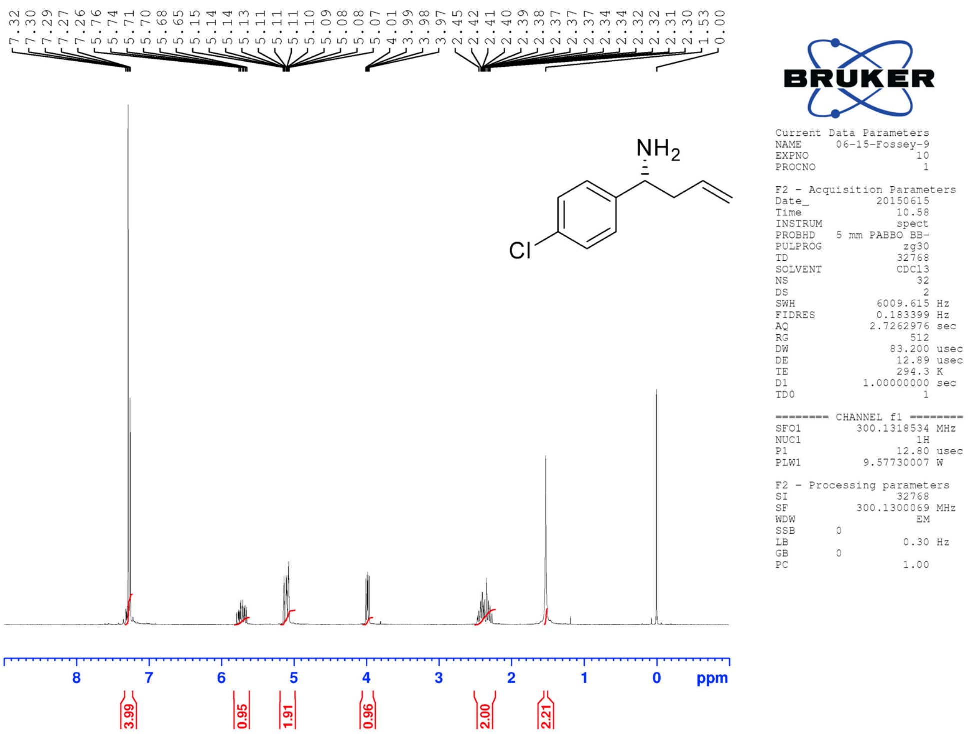

*Carbon NMR Spectrum of **S3e***

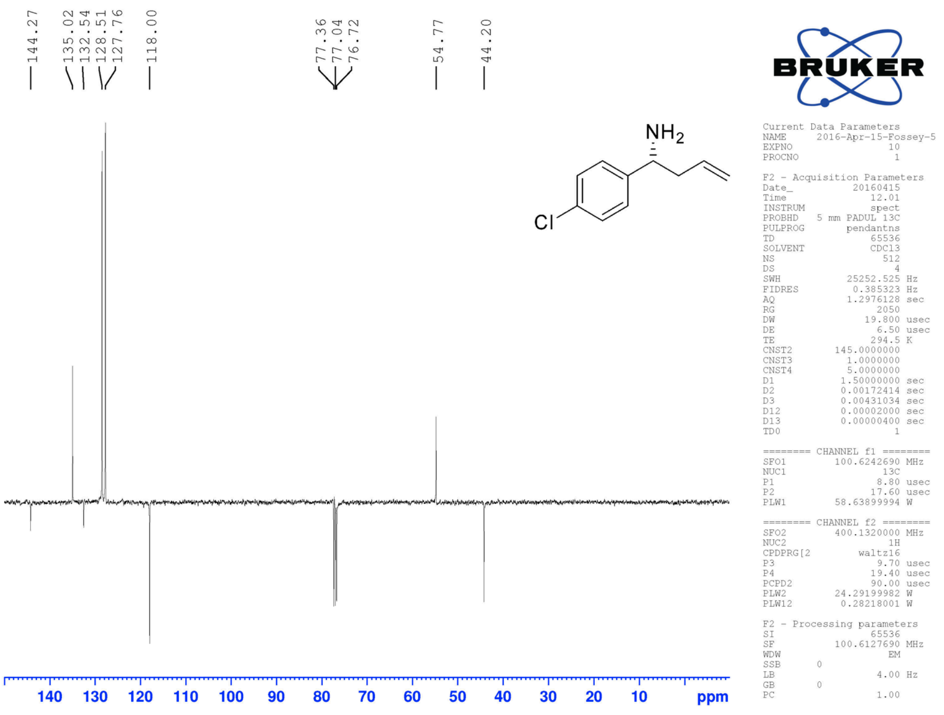

Proton NMR Spectrum of ***S4a***

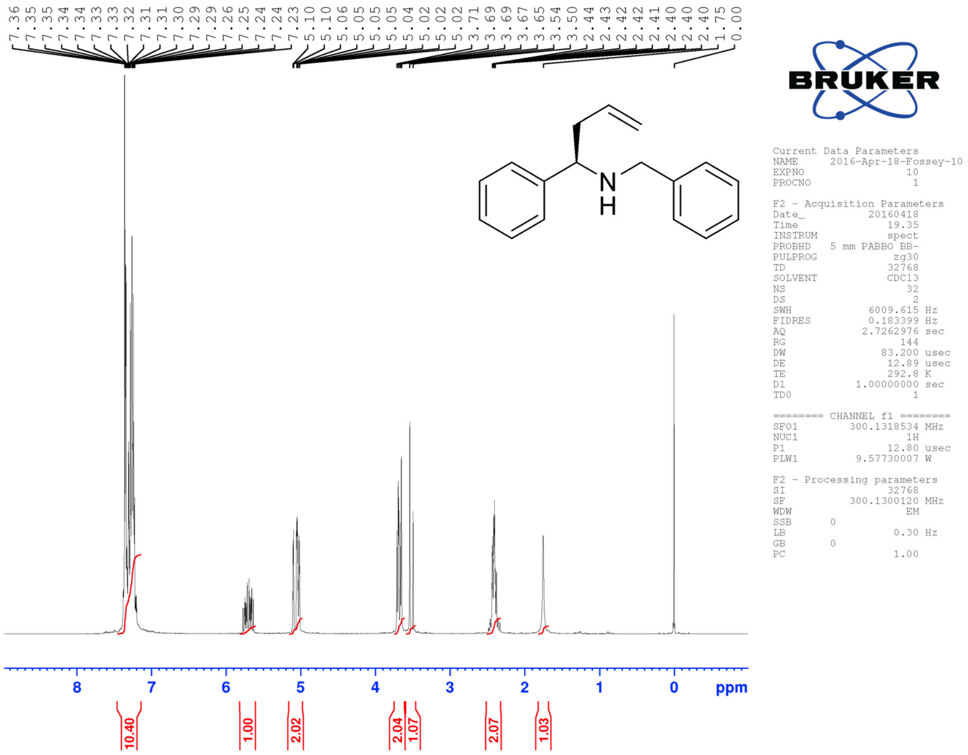

Carbon NMR Spectrum of ***S4a***

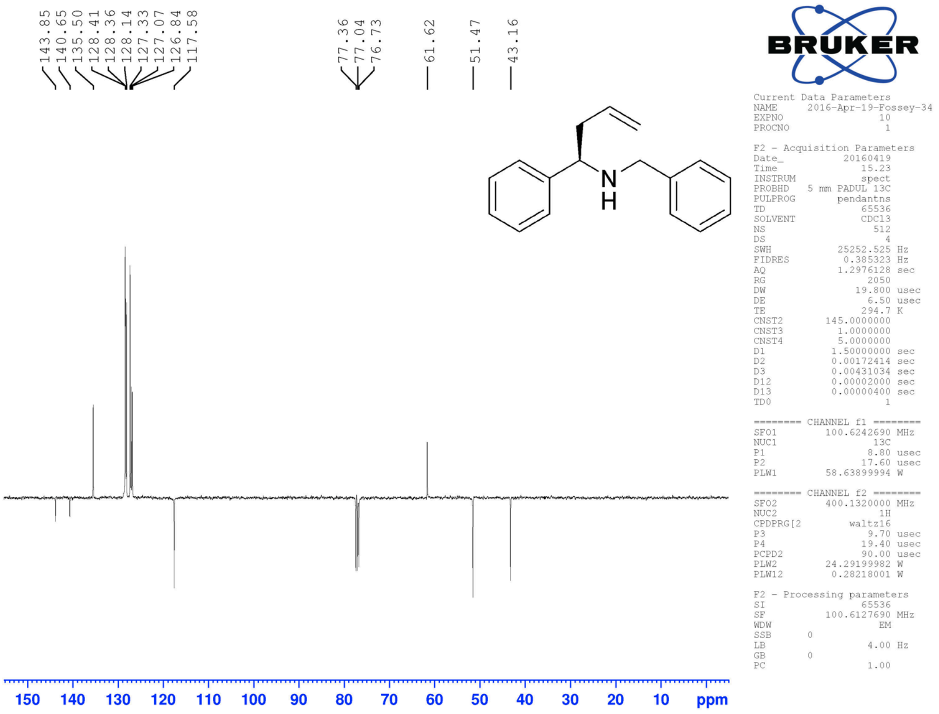

Proton NMR Spectrum of ***S4b***

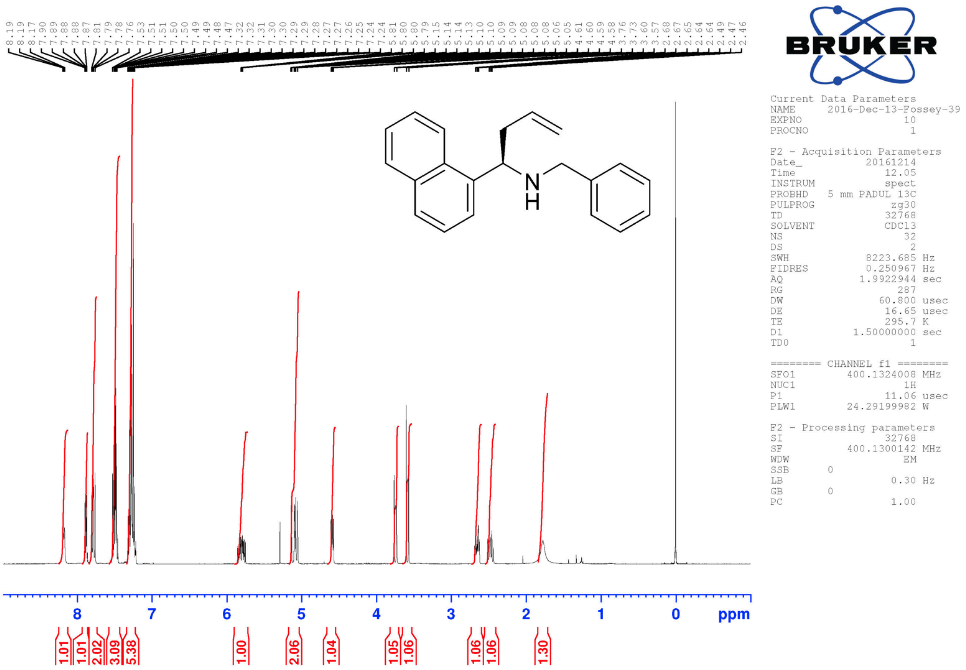

Carbon NMR Spectrum of ***S4b***

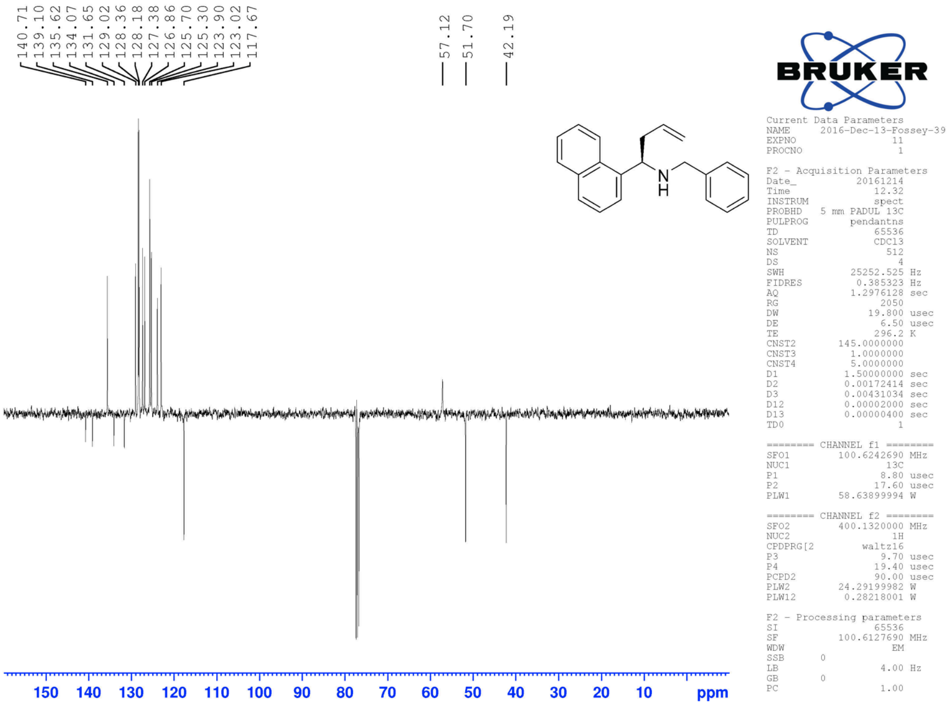

Proton NMR Spectrum of ***S4c***

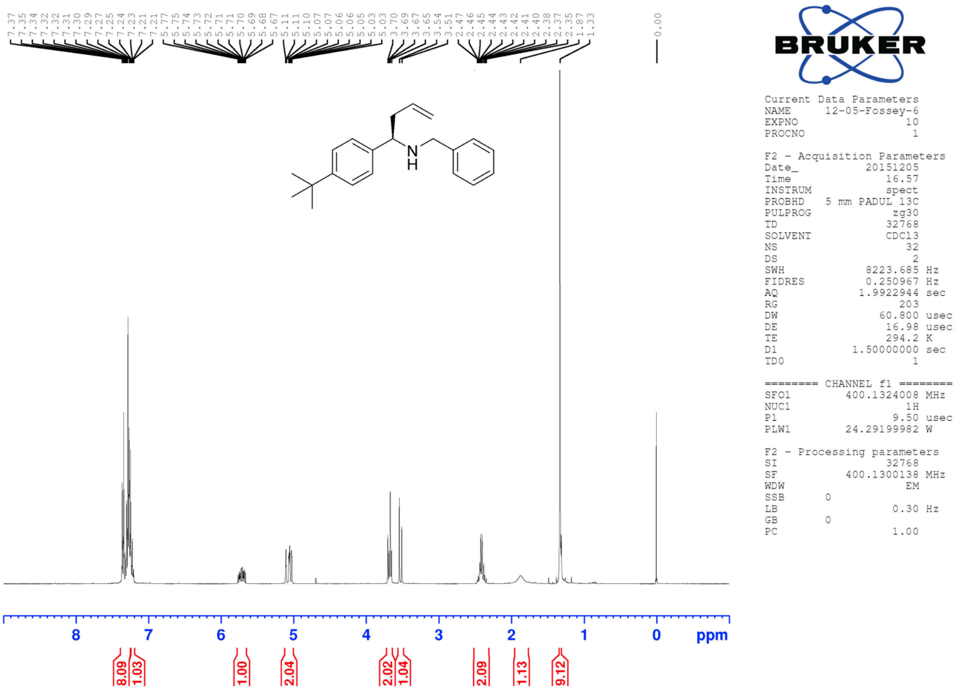

Carbon NMR Spectrum of ***S4c***

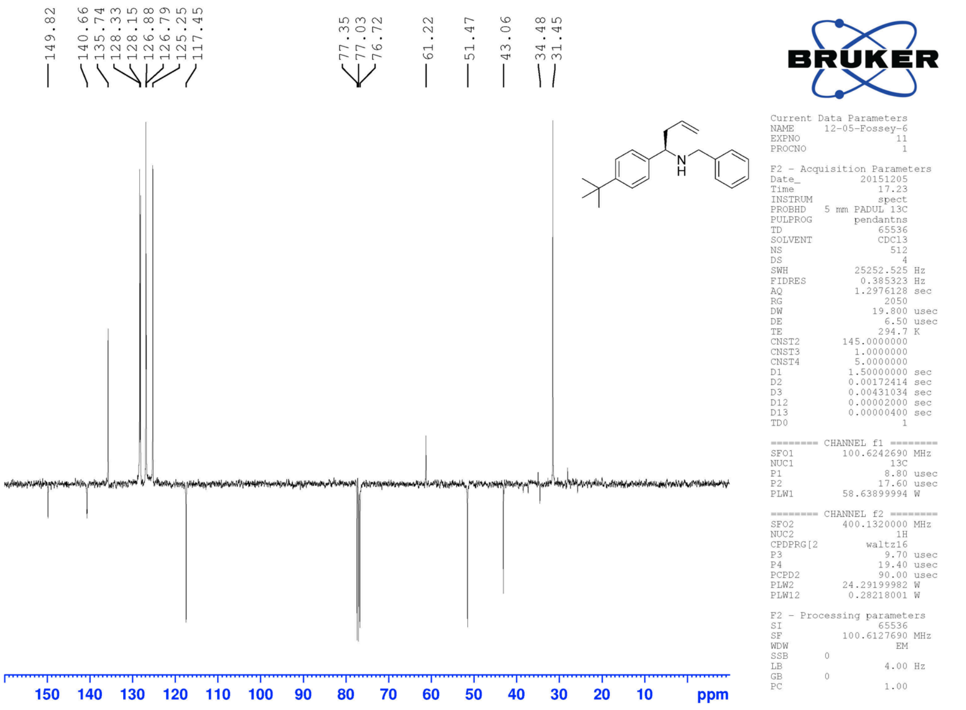

Proton NMR Spectrum of ***S4d***

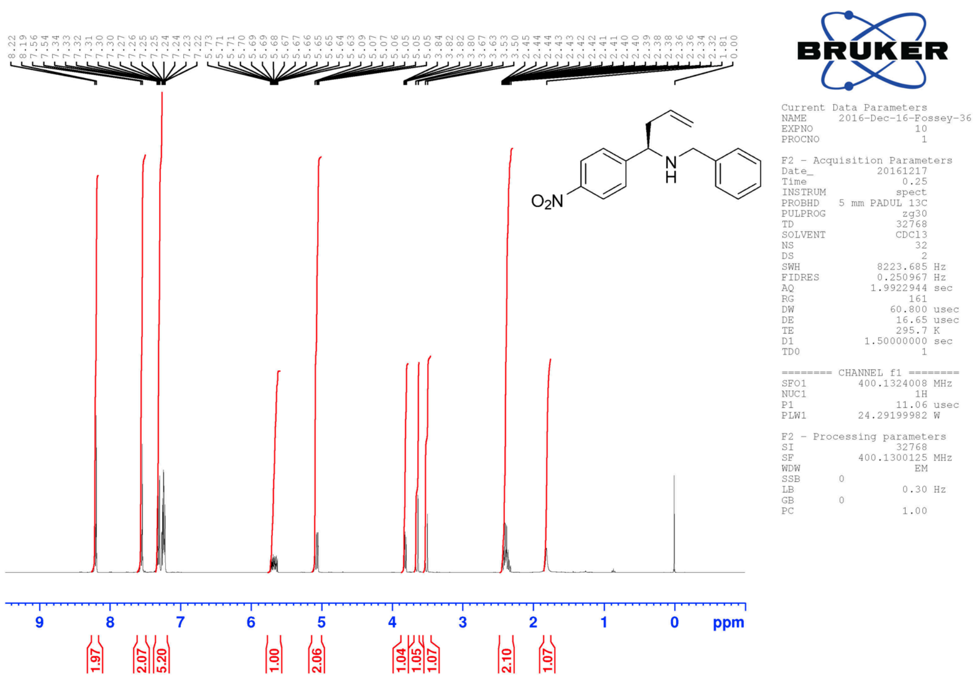

Carbon NMR Spectrum of ***S4d***

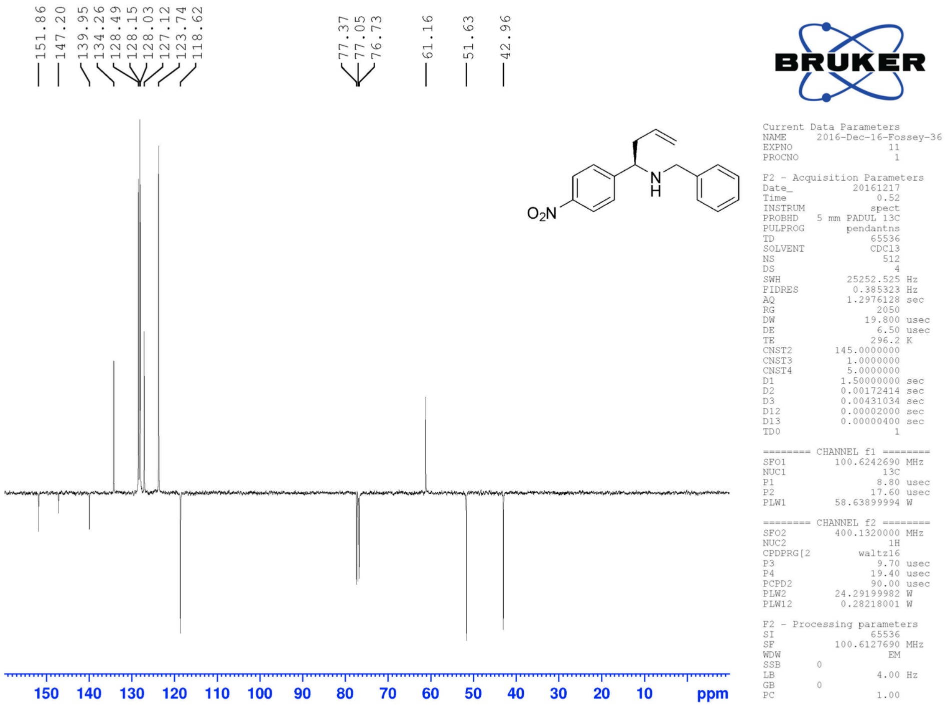

Proton NMR Spectrum of ***S4e***

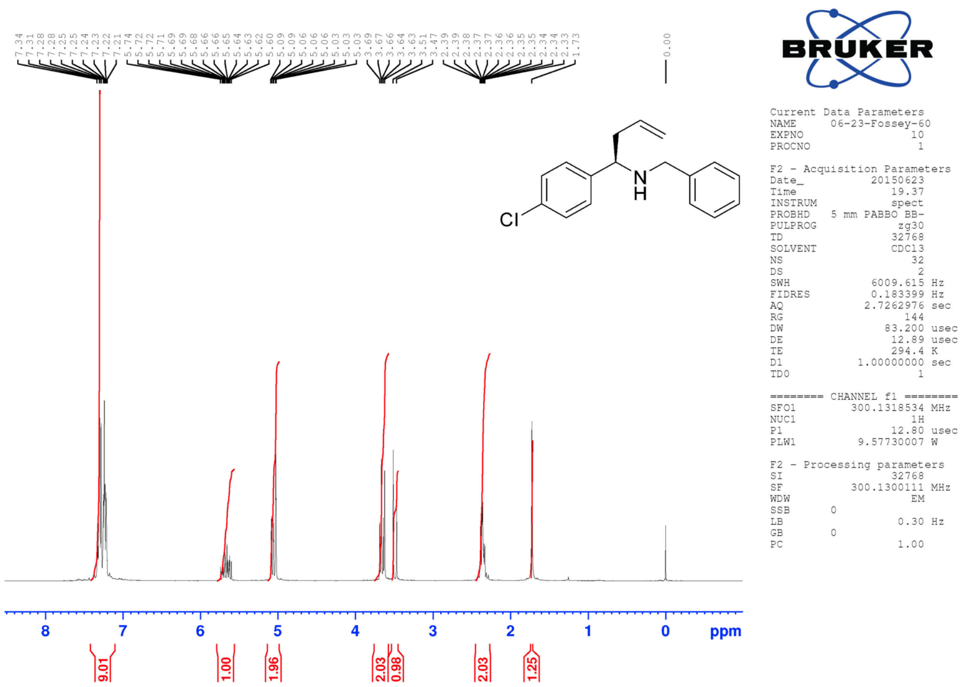

Carbon NMR Spectrum of ***S4e***

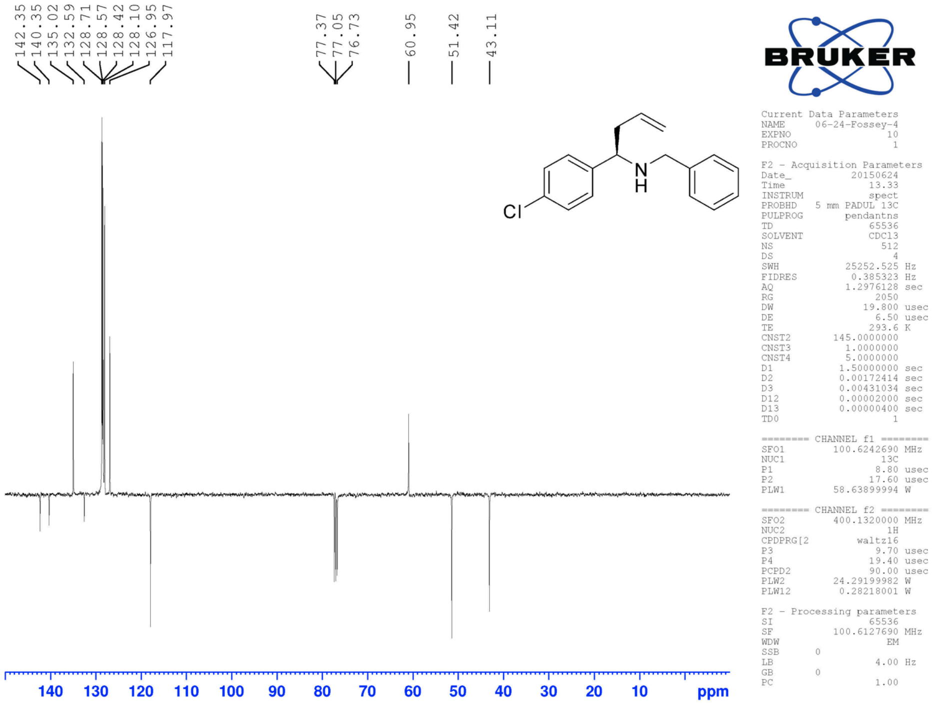

Proton NMR Spectrum of ***S4f***

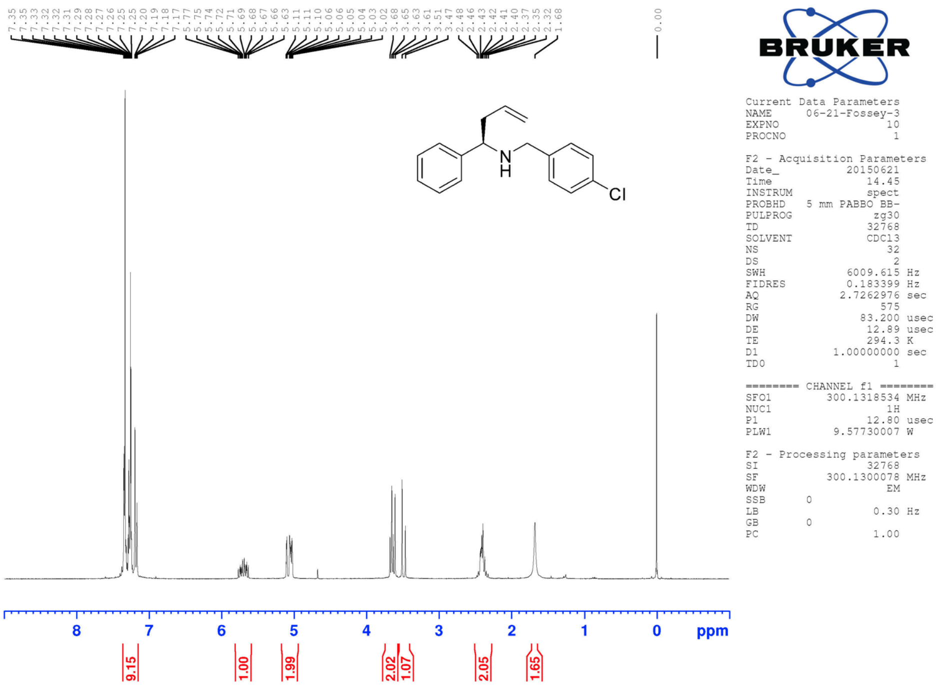

Carbon NMR Spectrum of ***S4f***

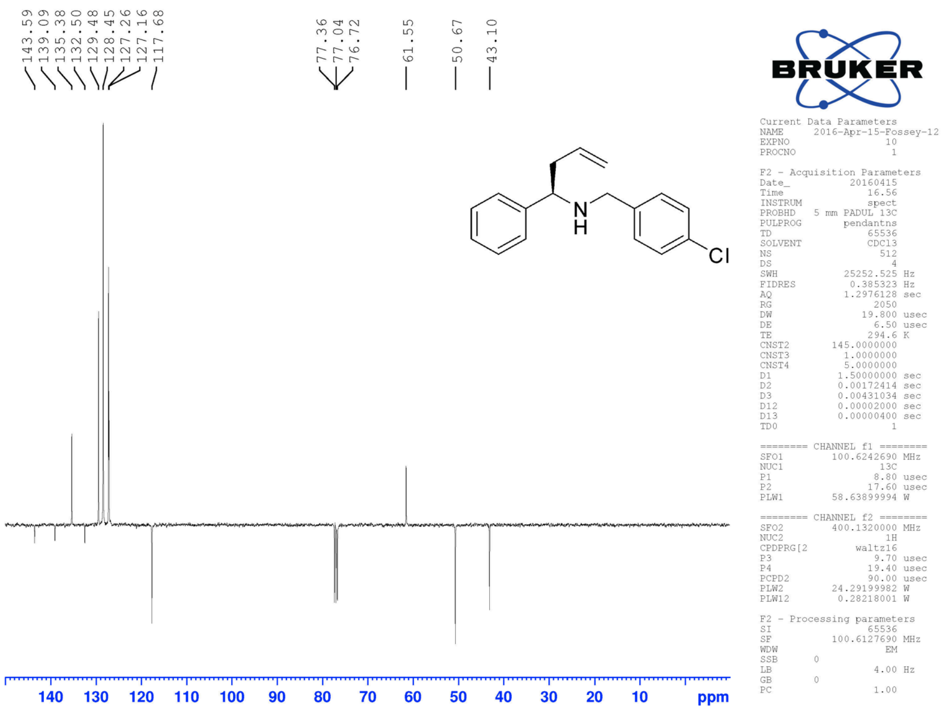

*Proton NMR Spectrum of **S4g***

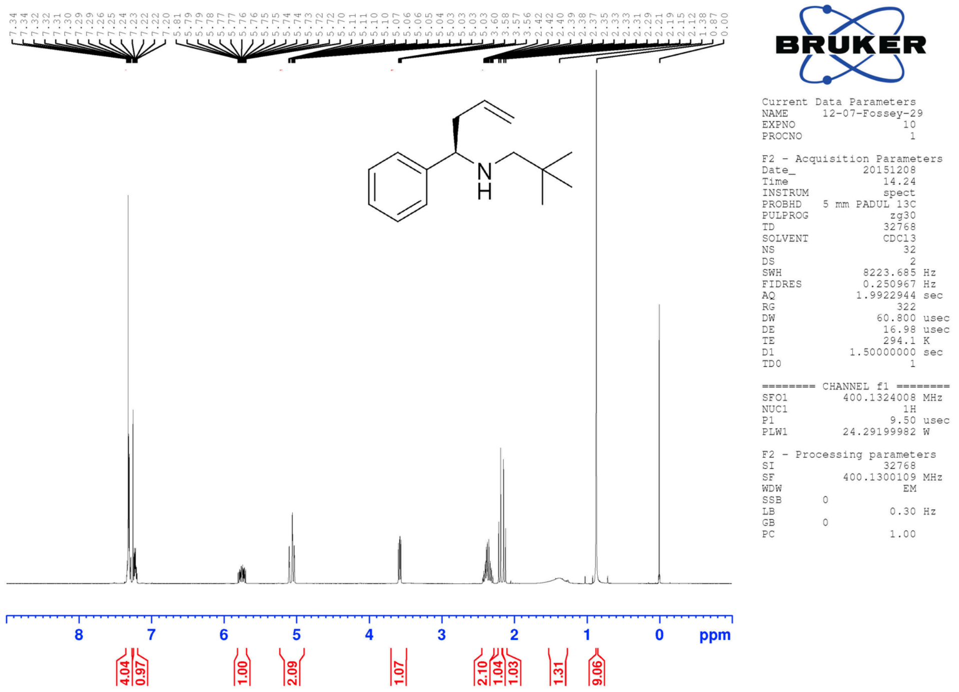

*Carbon NMR Spectrum of **S4g***

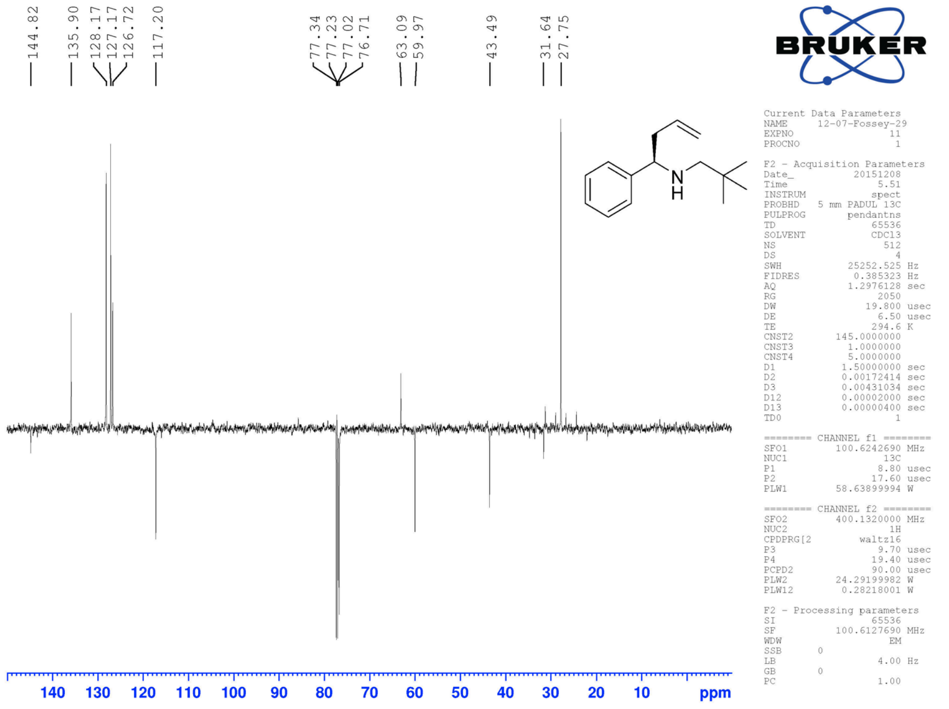

Proton NMR Spectrum of ***S4h***

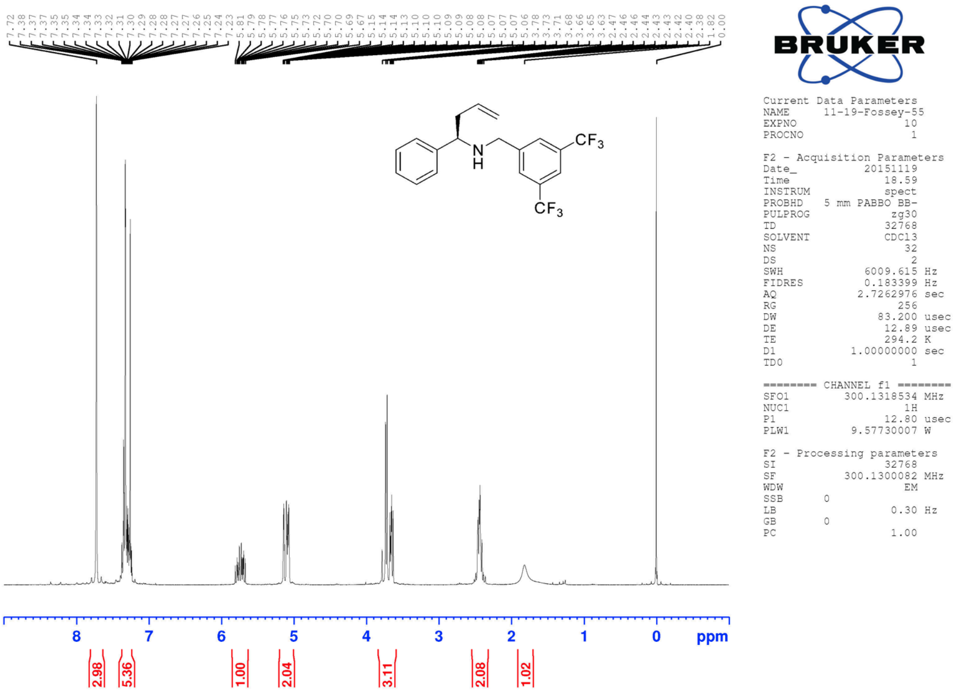

Carbon NMR Spectrum of ***S4h***

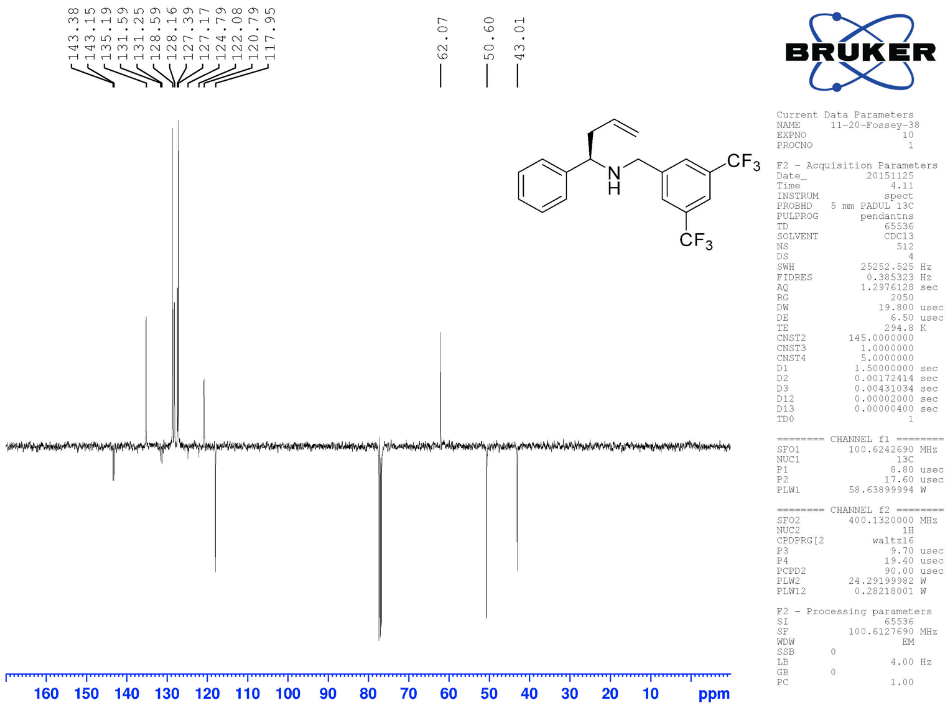

Fluorine NMR Spectrum of ***S4h***

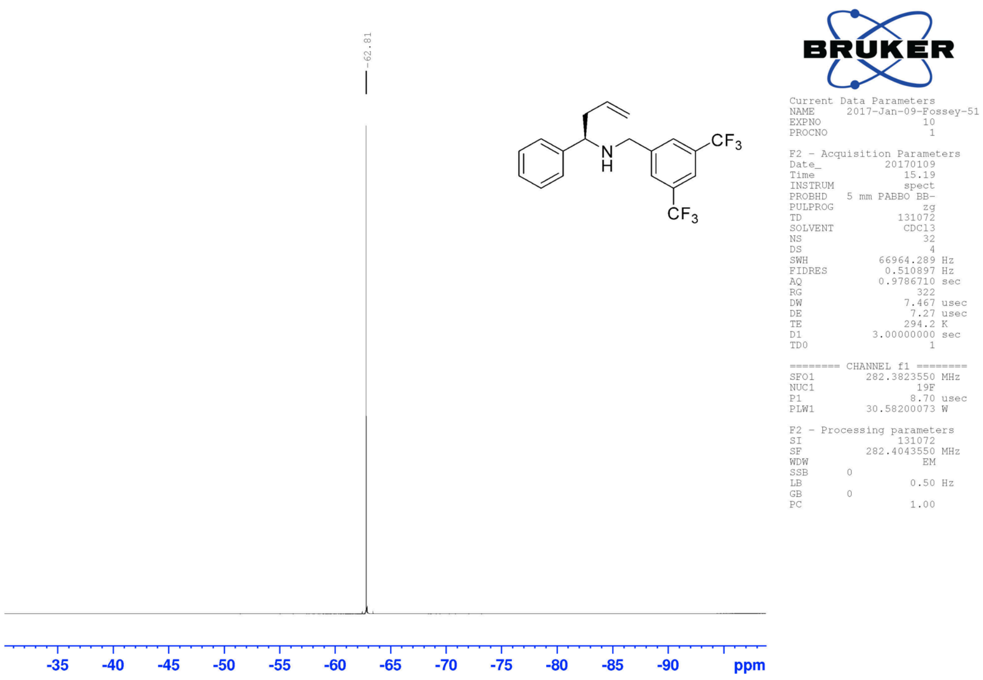

Proton NMR Spectrum of ***S4i***

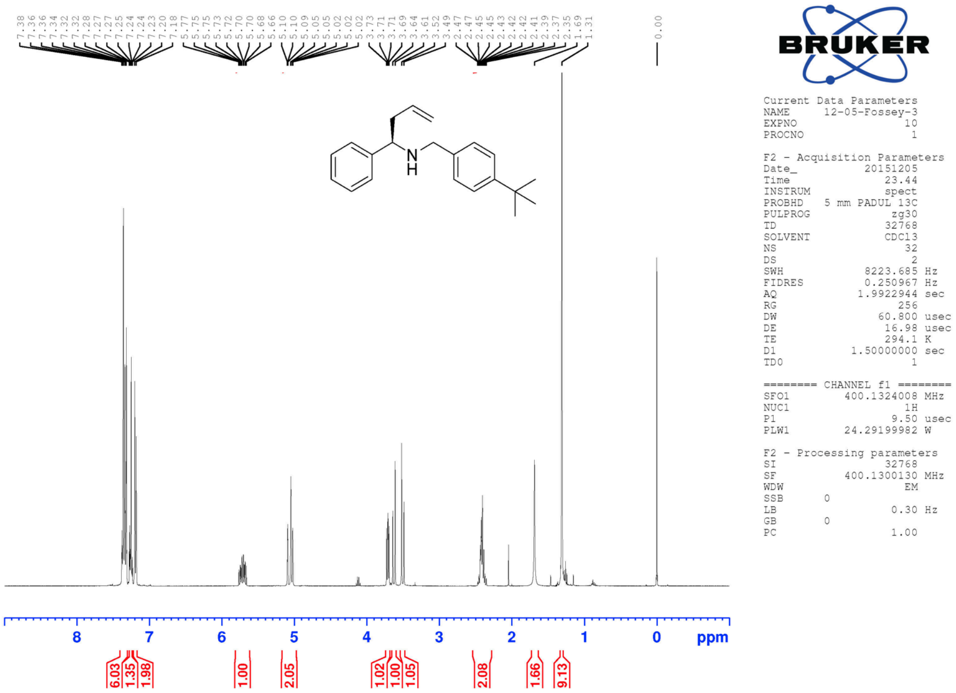

Carbon NMR Spectrum of ***S4i***

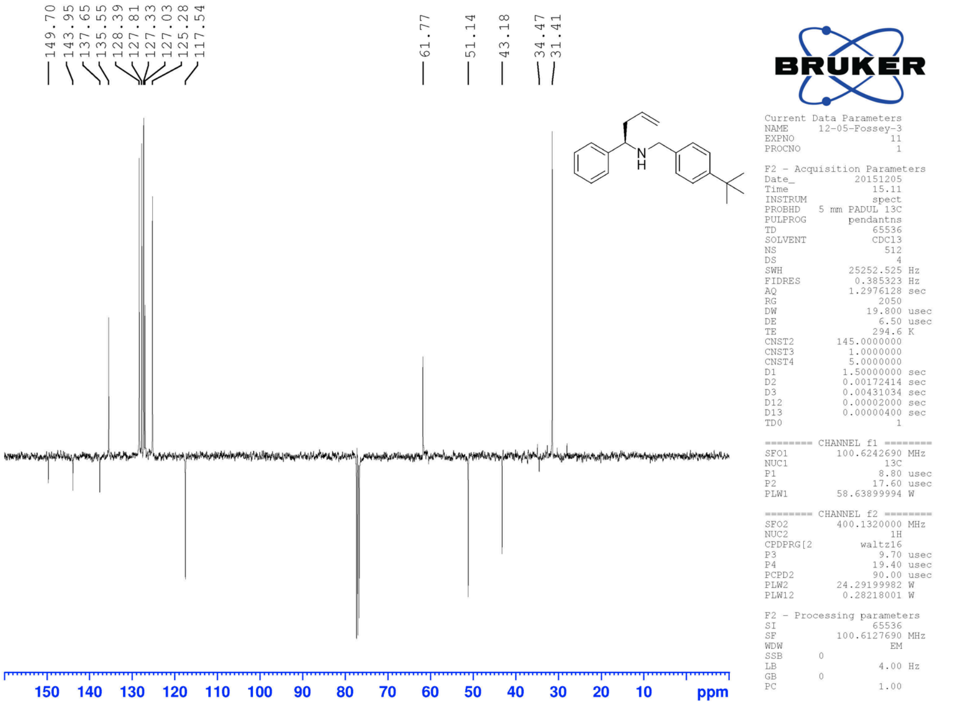

*Proton NMR Spectrum of **S4j***

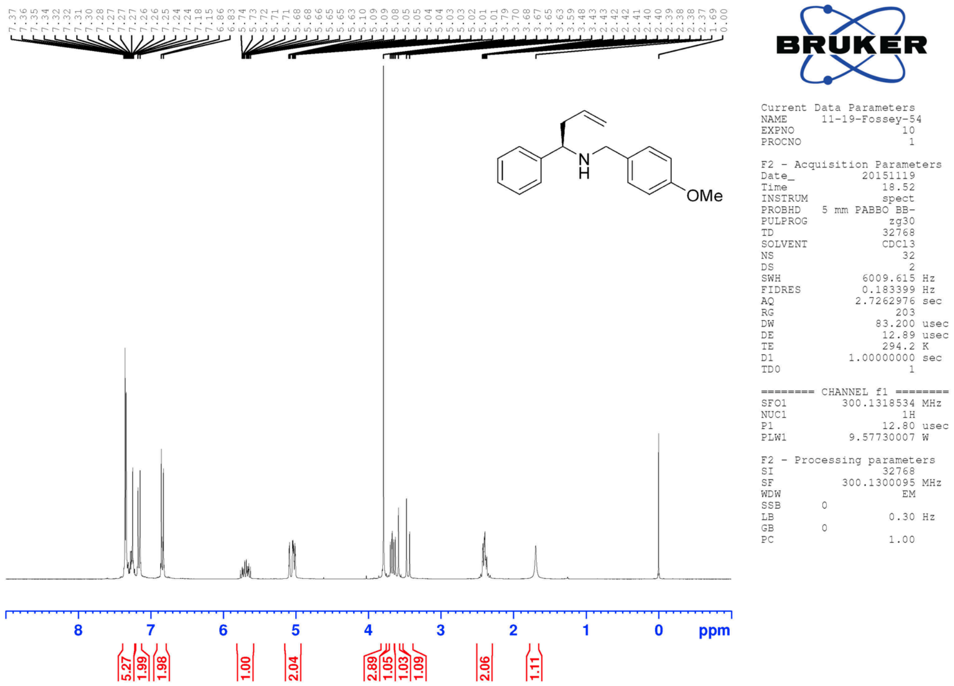

*Carbon NMR Spectrum of **S4j***

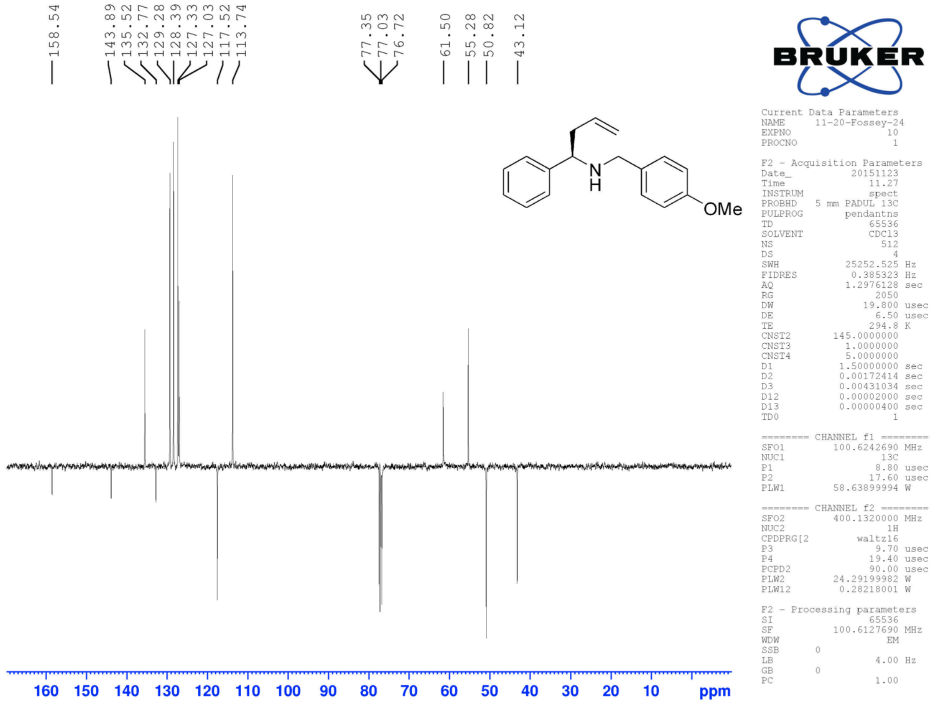

*Proton NMR Spectrum of **1a***

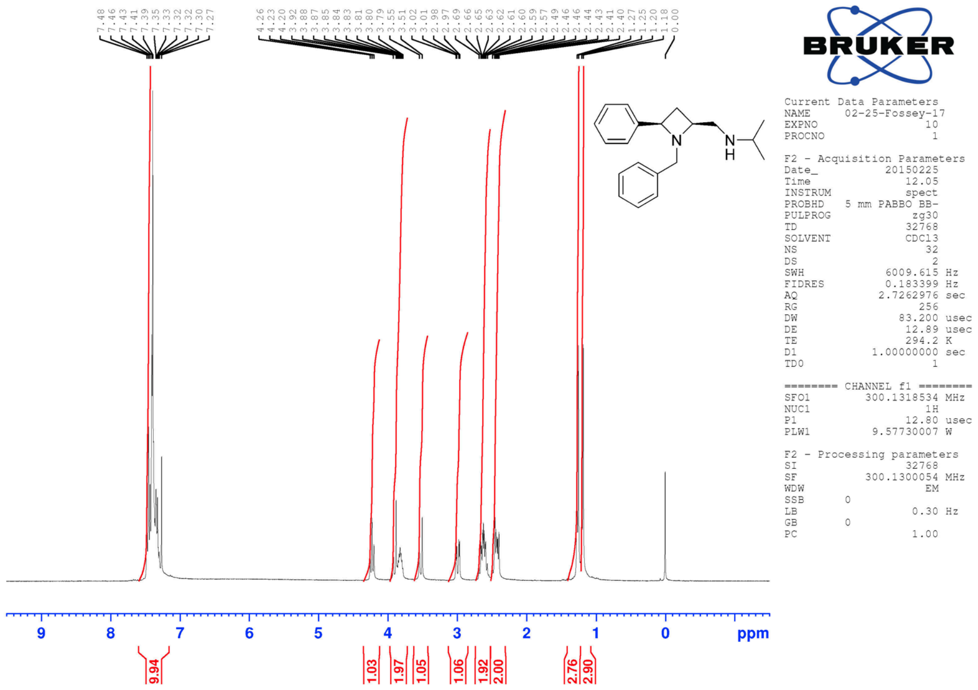

*Carbon NMR Spectrum of **1a***

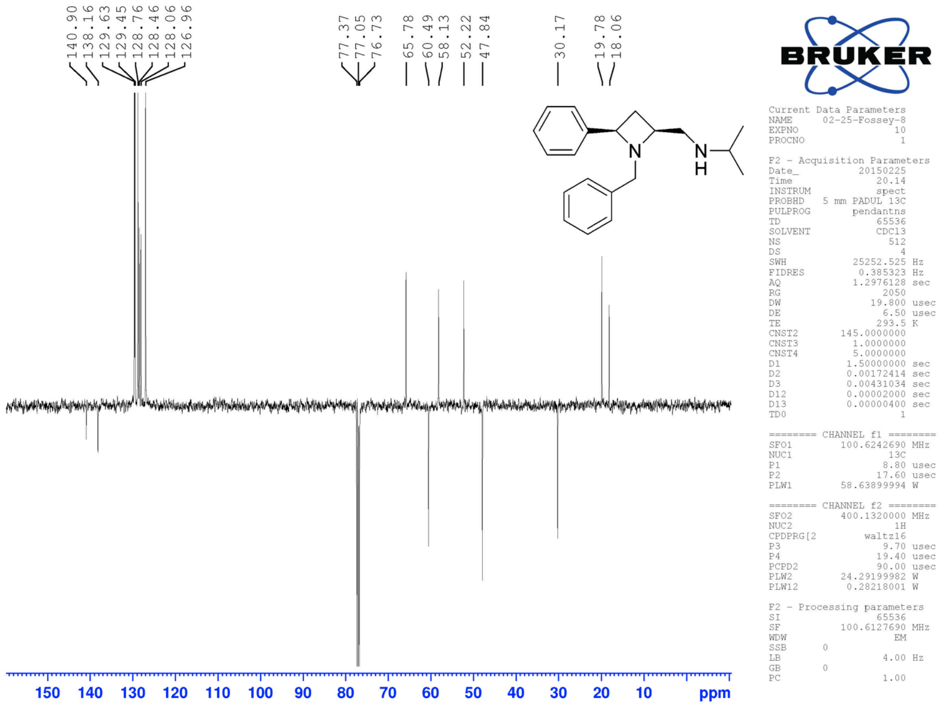

*Proton NMR Spectrum of **1b***

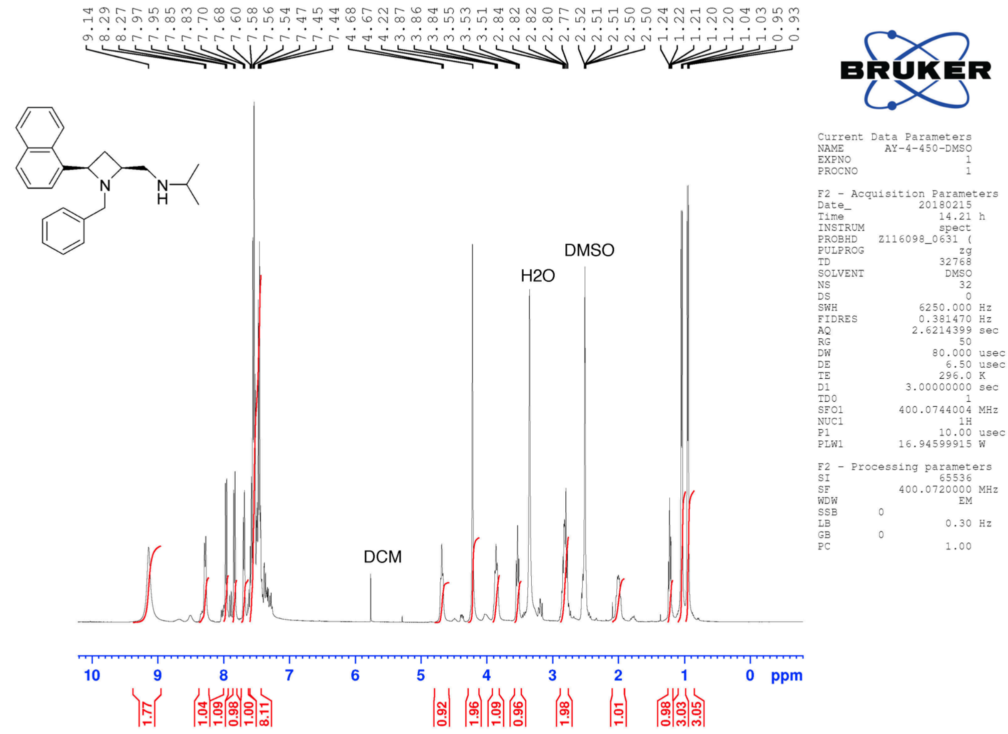

*Carbon NMR Spectrum of **1b***

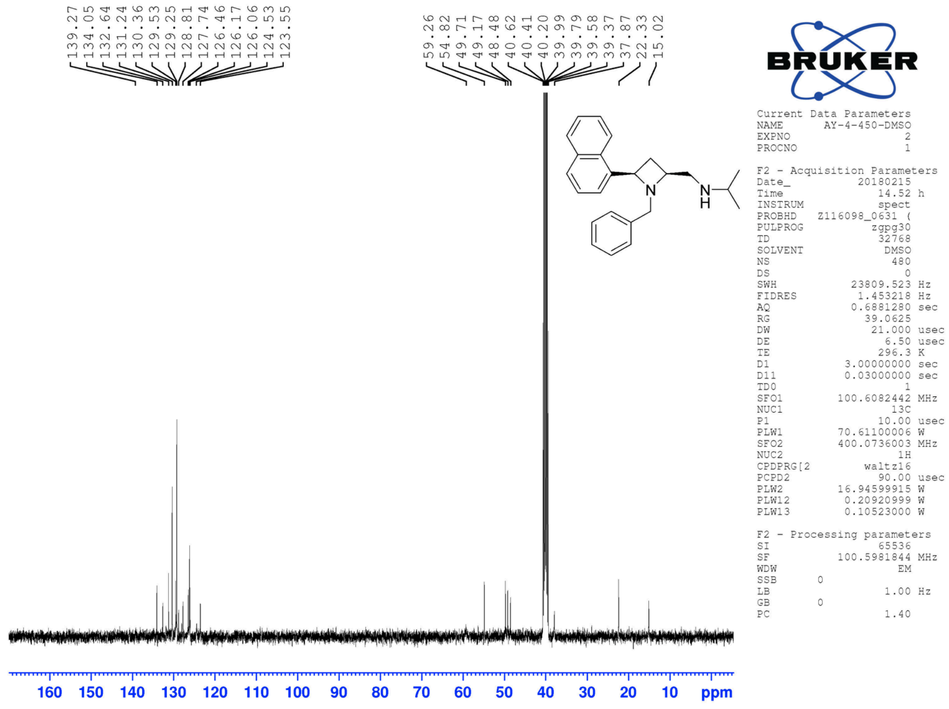

*Proton NMR Spectrum of **1c***

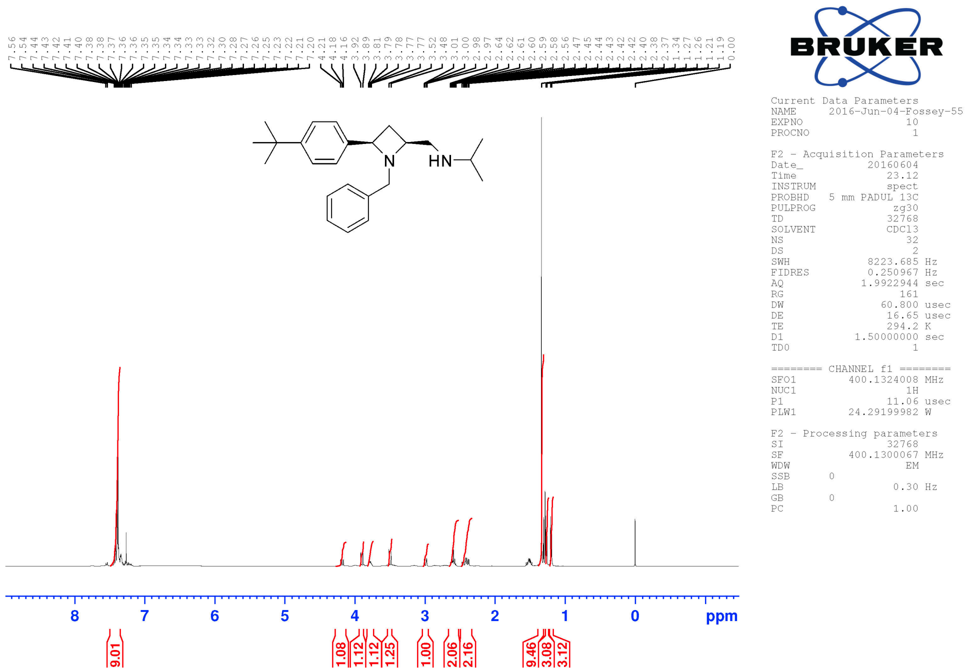

*Carbon NMR Spectrum of **1c***

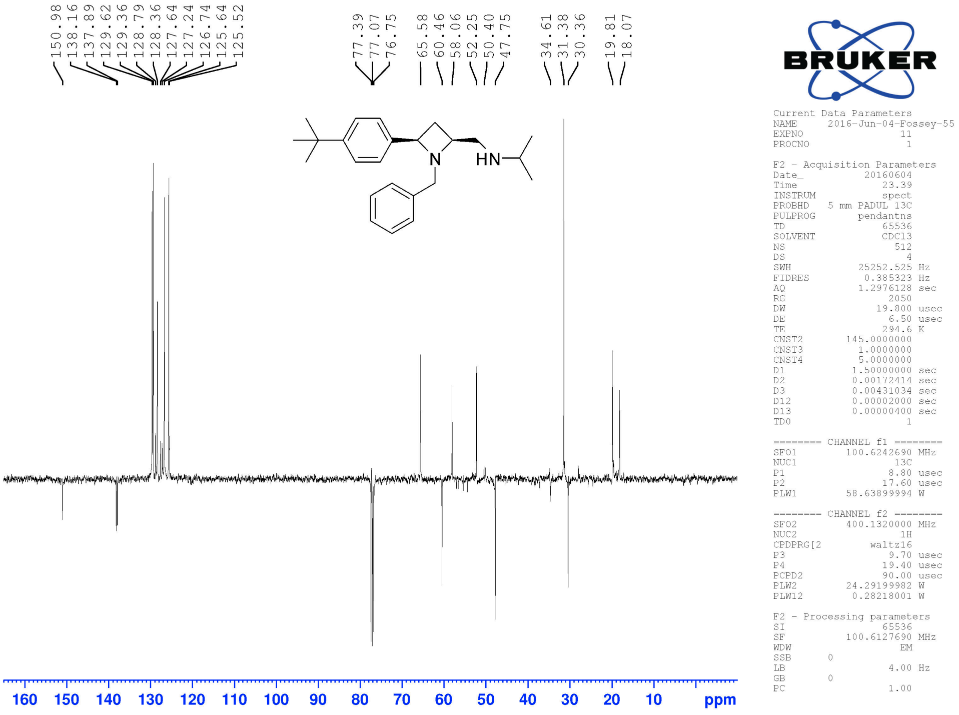

*Proton NMR Spectrum of **1d***

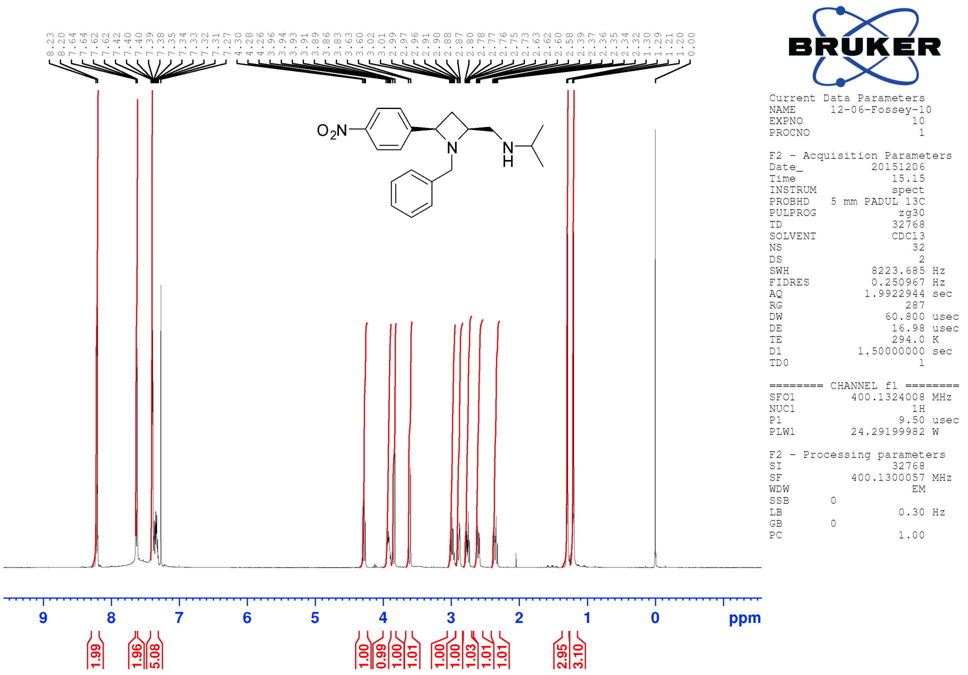

*Carbon NMR Spectrum of **1d***

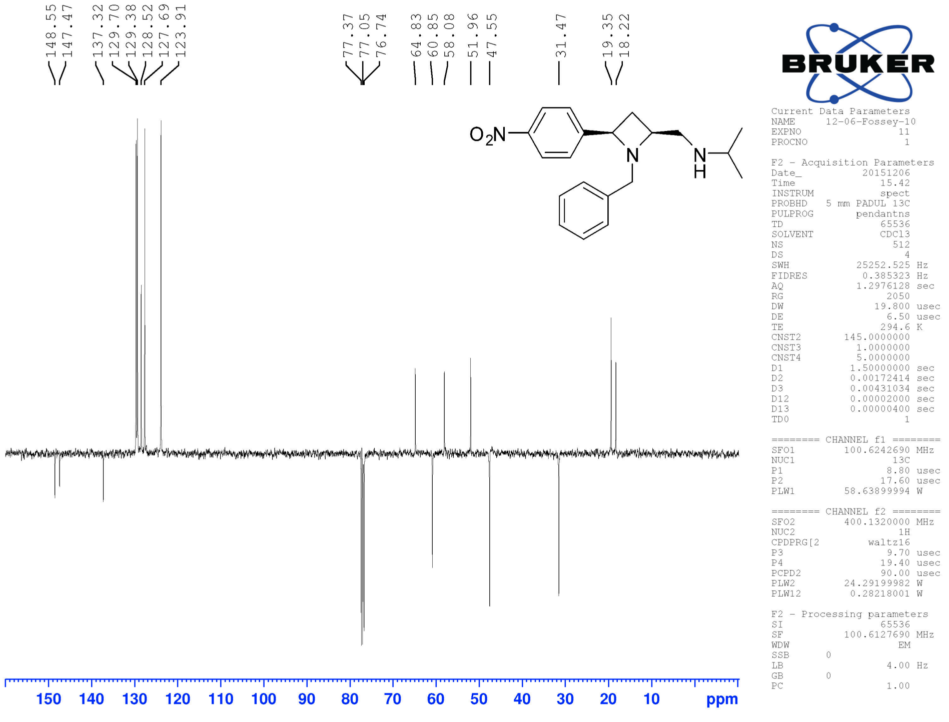

*Proton NMR Spectrum of **1e***

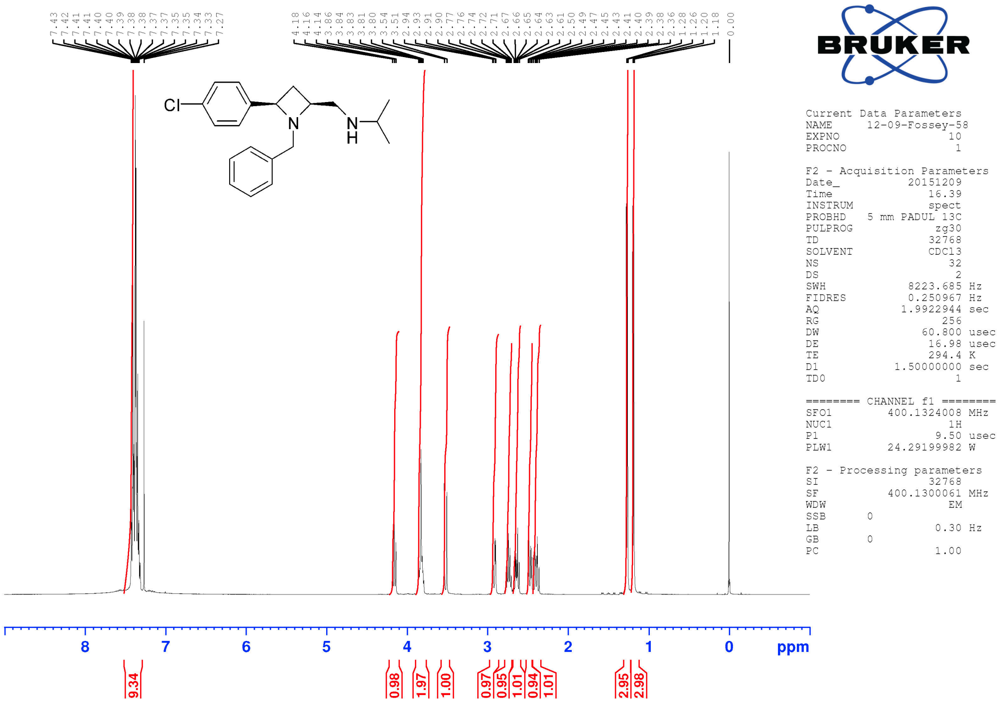

*Carbon NMR Spectrum of **1e***

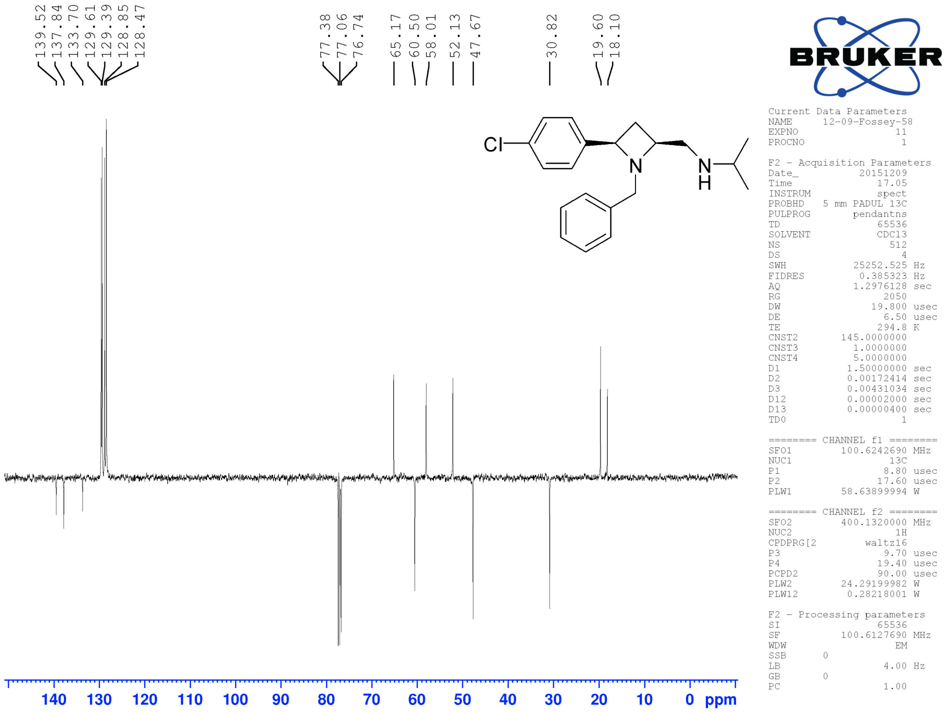

*Proton NMR Spectrum of **1f***

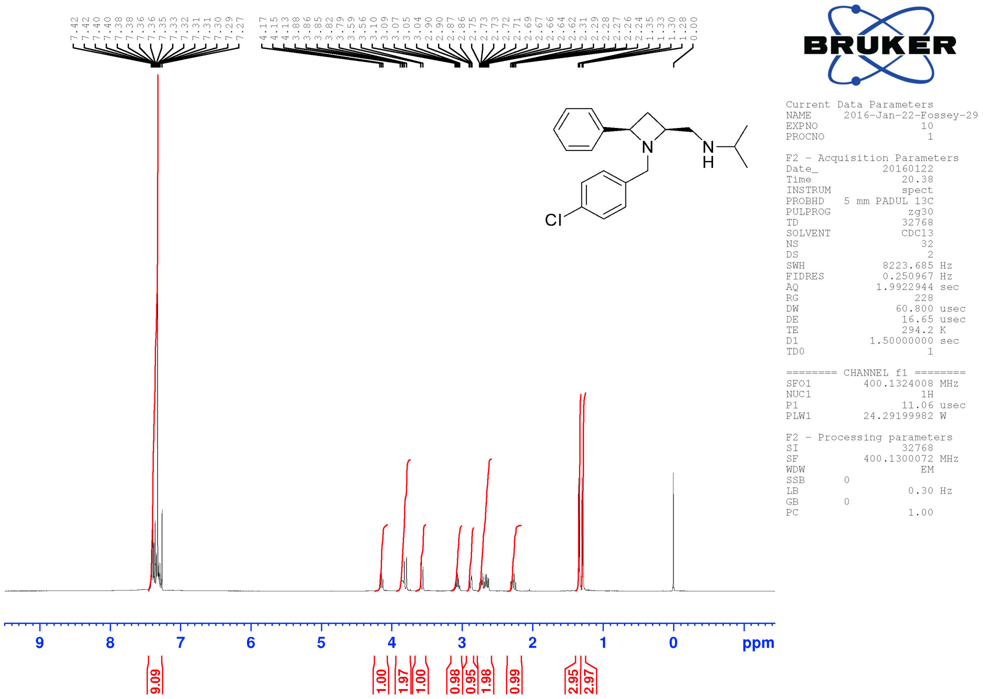

*Carbon NMR Spectrum of **1f***

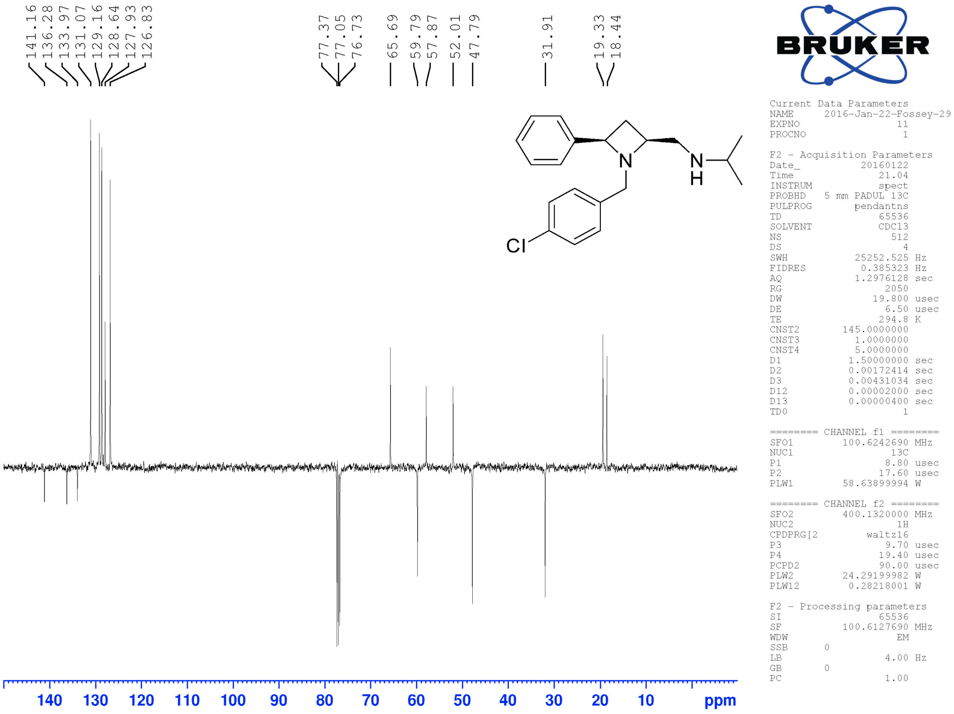

Proton NMR Spectrum of ***1g***

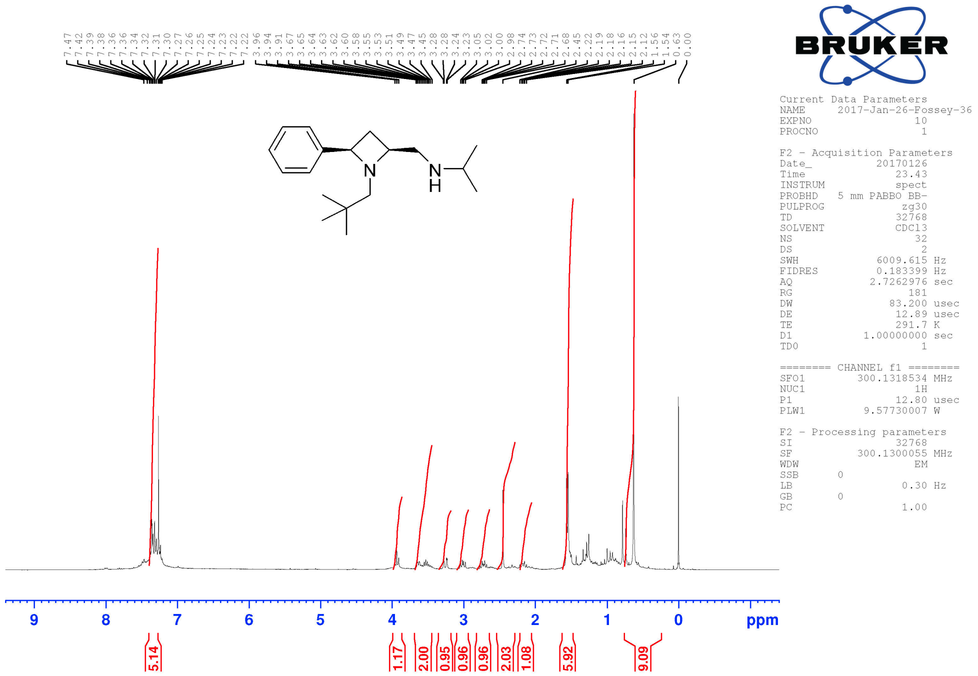

Carbon NMR Spectrum of ***1g***

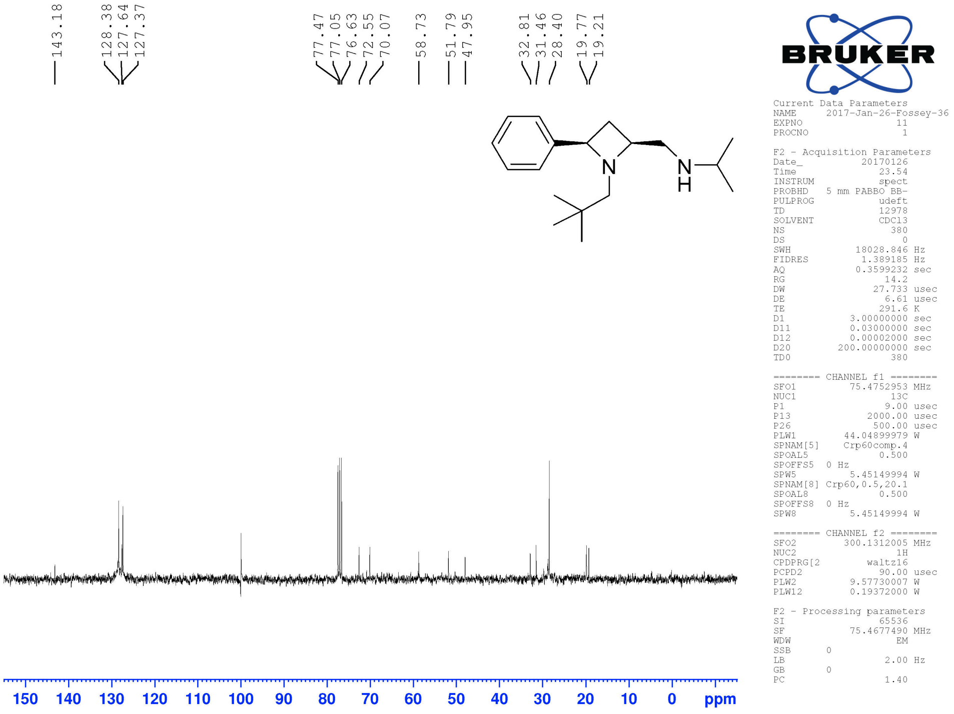

*Proton NMR Spectrum of **1h***

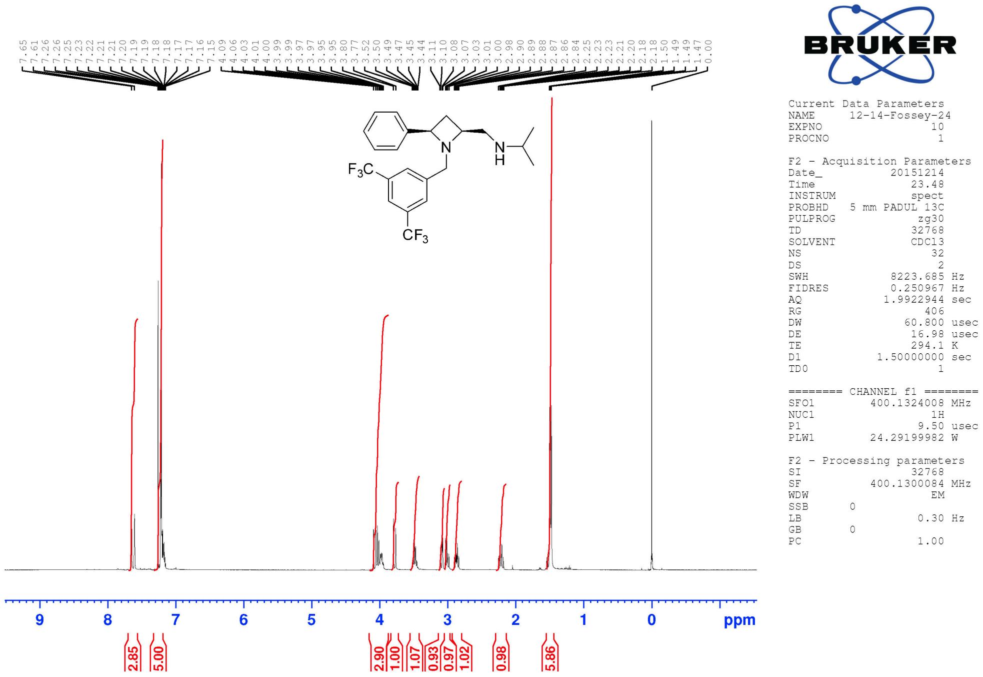

*Carbon NMR Spectrum of **1h***

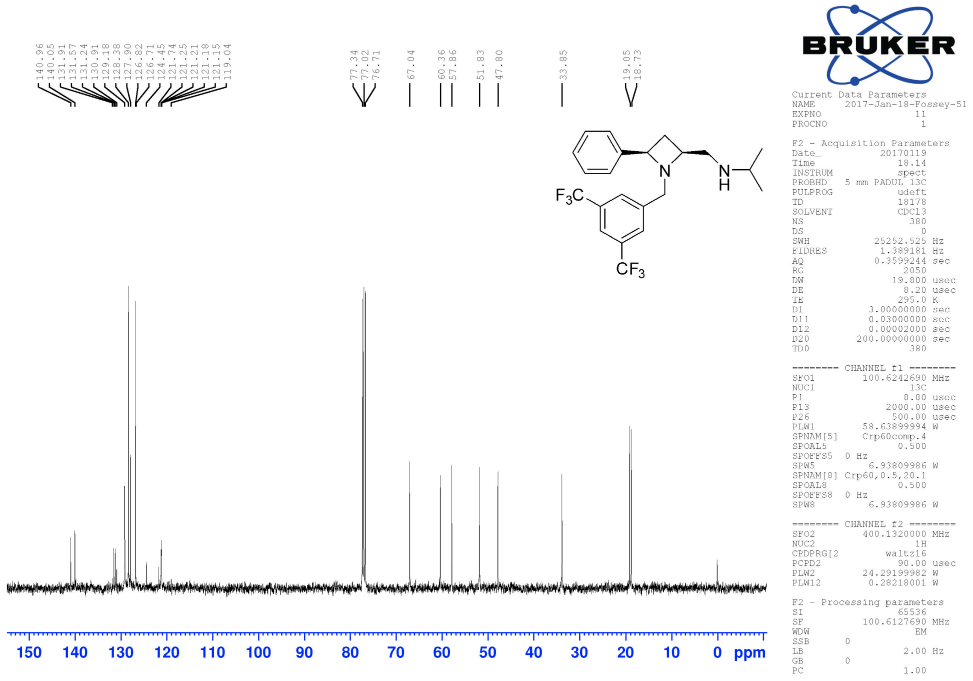

*Fluorine NMR Spectrum of **1h***

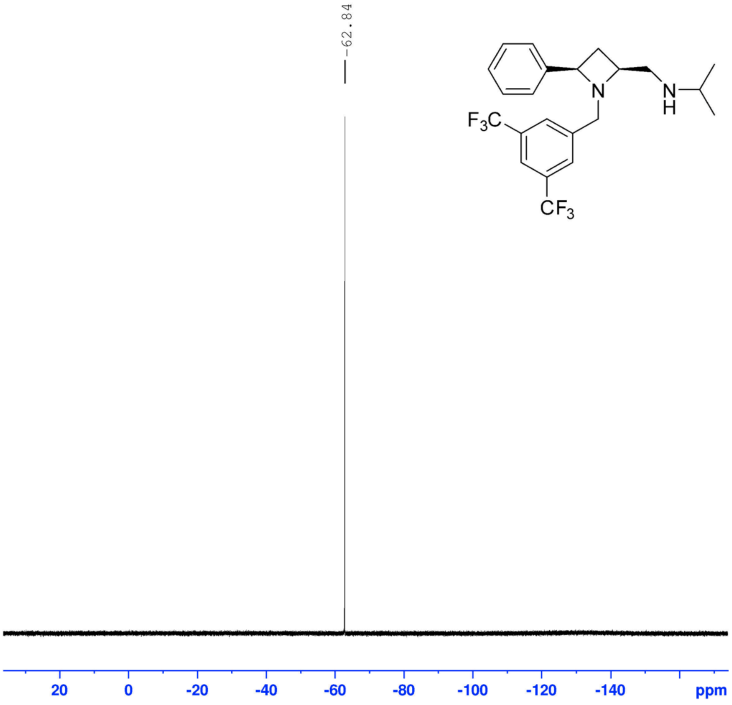


```
Current Data Parameters
NAME      2018-Feb-07-Fossey-46
EXPNO                     10
PROCNO                     1

F2 - Acquisition Parameters
Date_               20180207
Time                   10.04
INSTRUM                spect
PROBHD    5 mm PABBO BB-
PULPROG                   zg
TD                    131072
SOLVENT                CDCl3
NS                        32
DS                         4
SWH                66964.289 Hz
FIDRES              0.510897 Hz
AQ                 0.9786710 sec
RG                      2050
DW                     7.467 usec
DE                      7.27 usec
TE                     292.6 K
D1                3.00000000 sec
TD0                        1

======== CHANNEL f1 ========
SFO1             282.3823550 MHz
NUC1                     19F
P1                      8.70 usec
PLW1             30.58200073 W

F2 - Processing parameters
SI                    131072
SF               282.4043550 MHz
WDW                       EM
SSB      0
LB                      0.50 Hz
GB       0
PC                      1.00
```

*Proton NMR Spectrum of **1i***

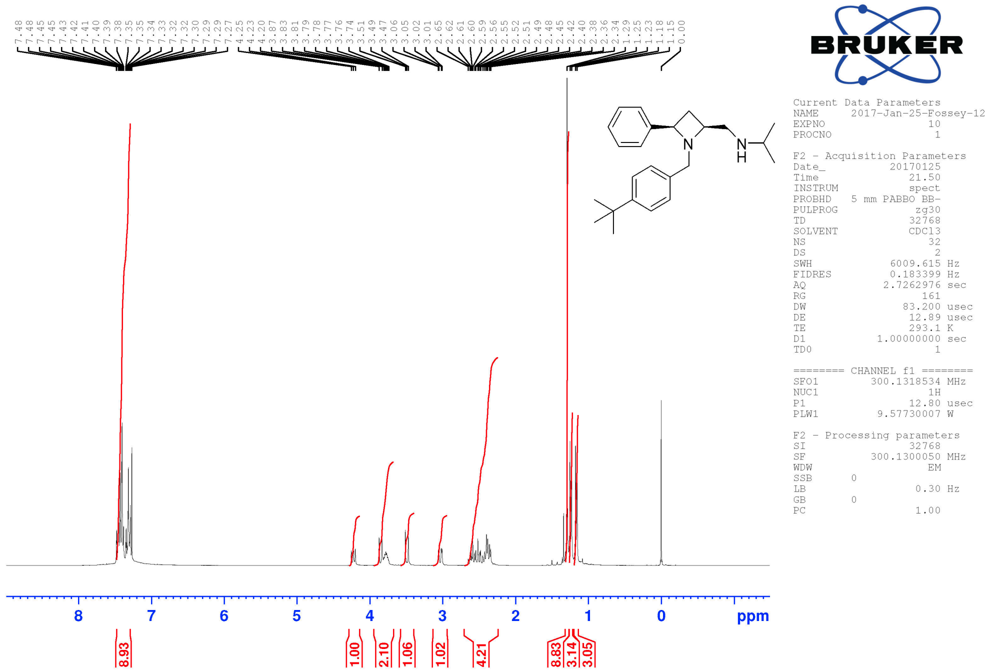

*Carbon NMR Spectrum of **1i***

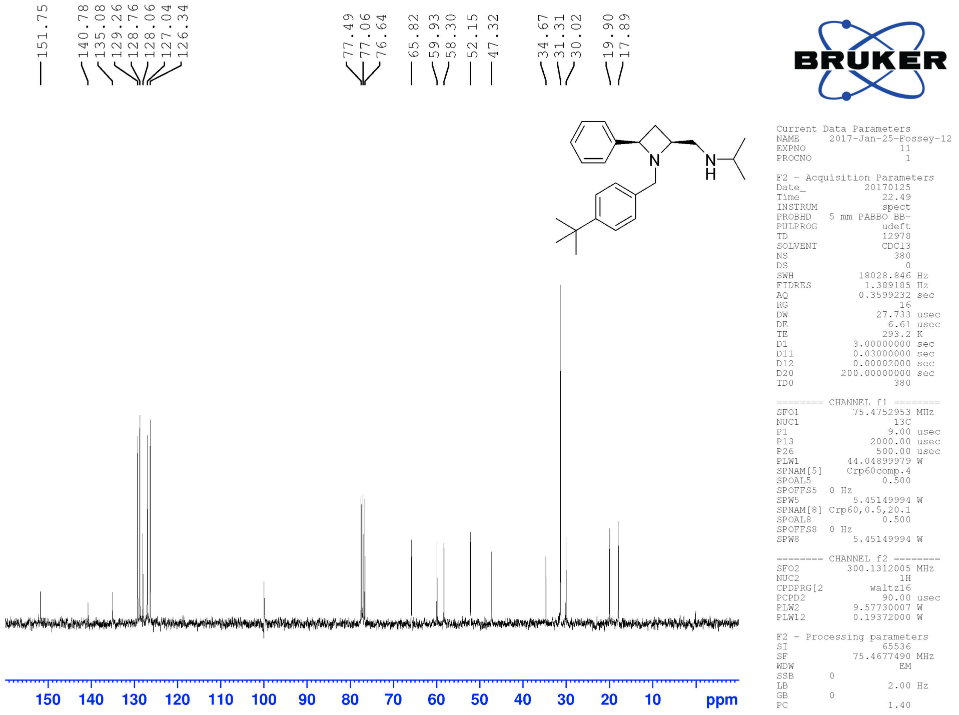

Proton NMR Spectrum of ***1j***

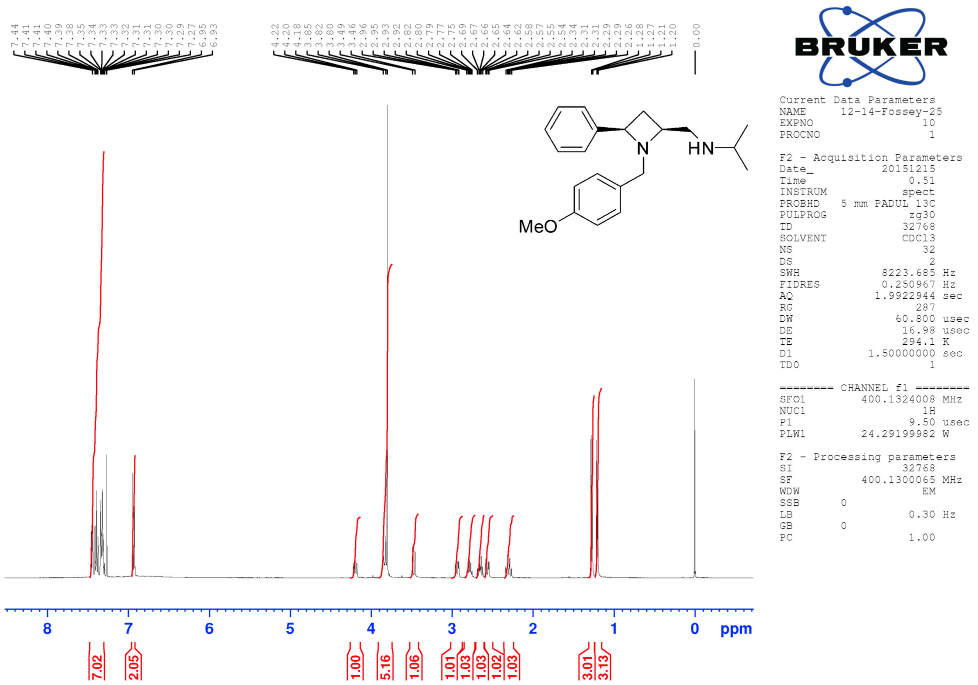

Carbon NMR Spectrum of ***1j***

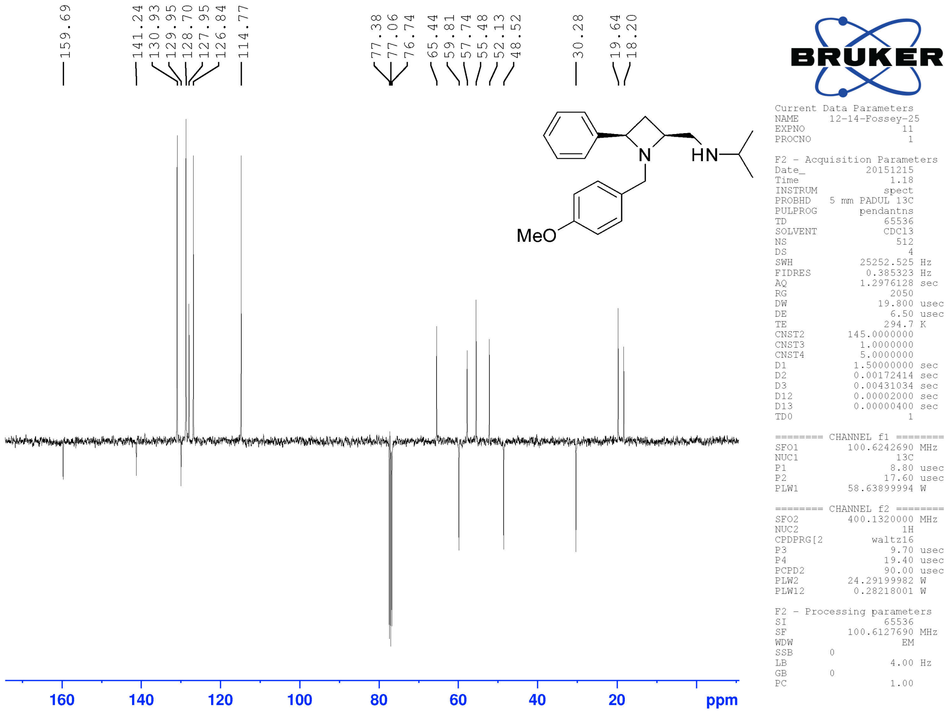

*Proton NMR Spectrum of **1k***

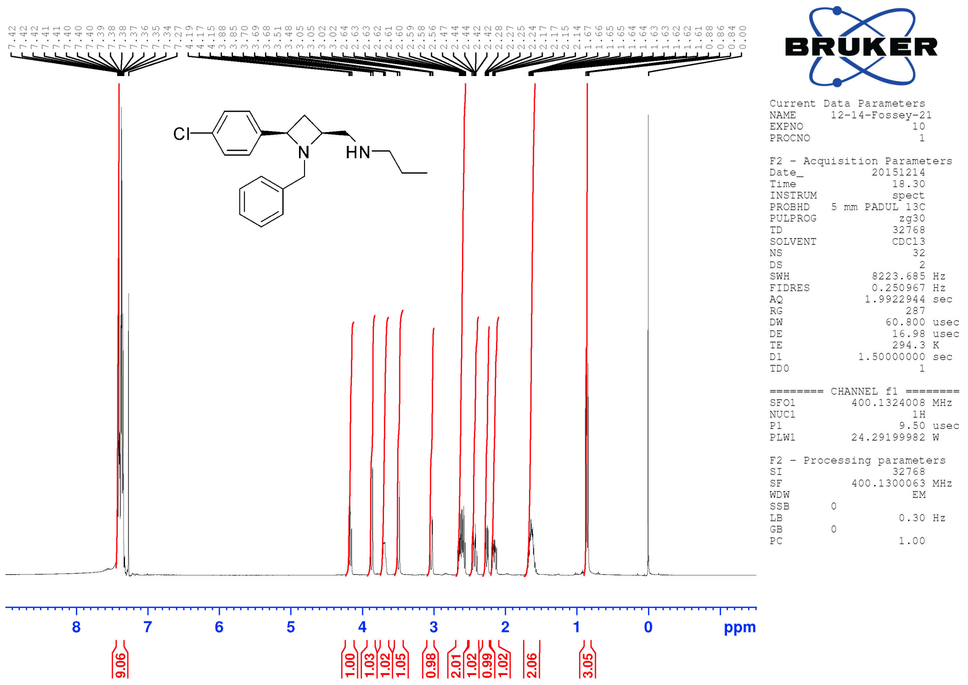

*Carbon NMR Spectrum of **1k***

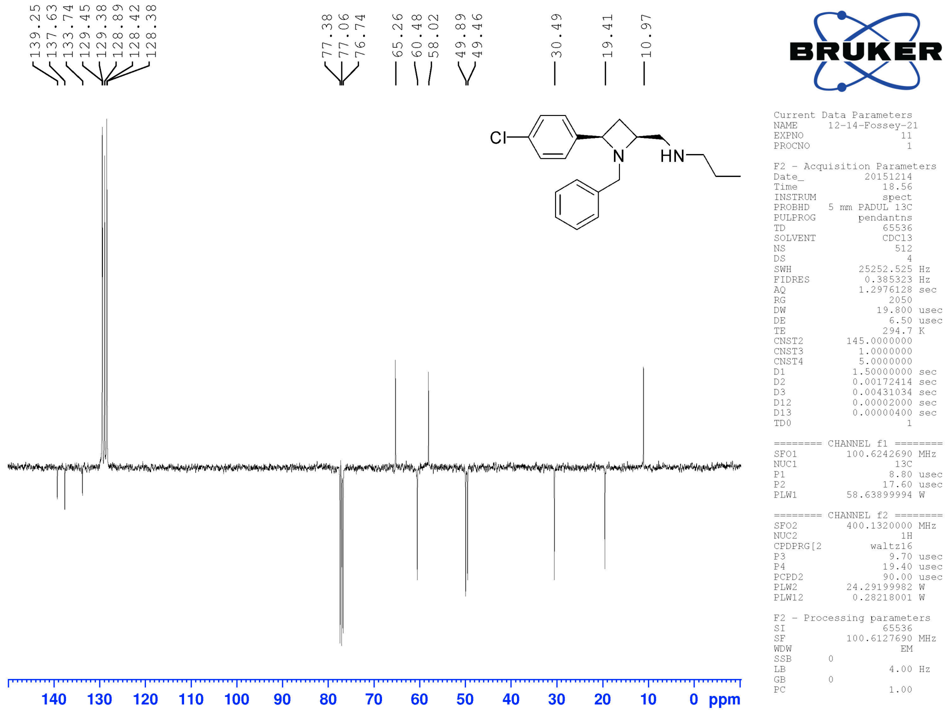

Proton NMR Spectrum of ***1l***

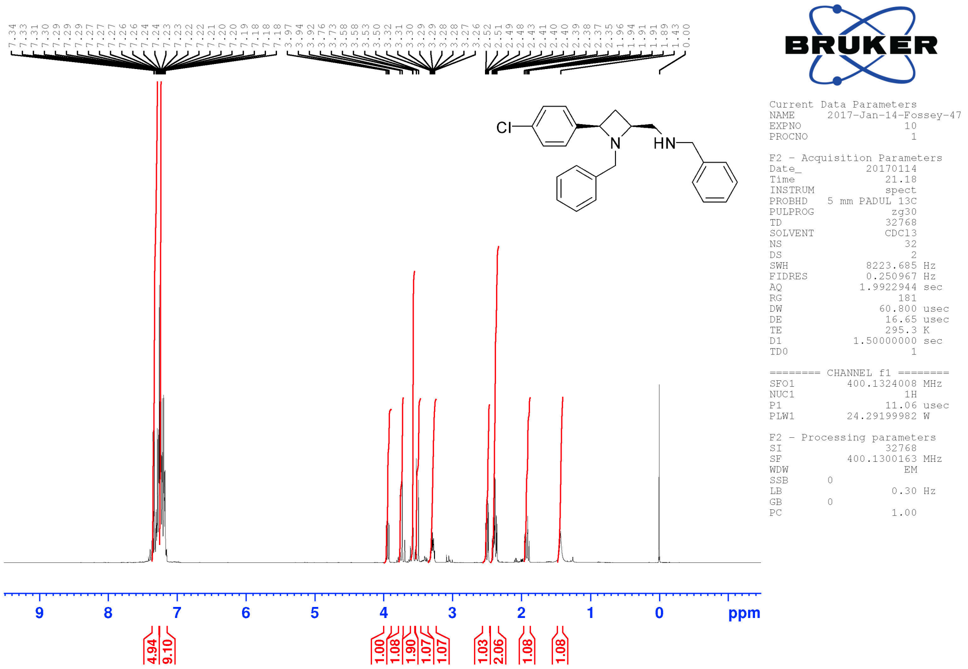

Carbon NMR Spectrum of ***1l***

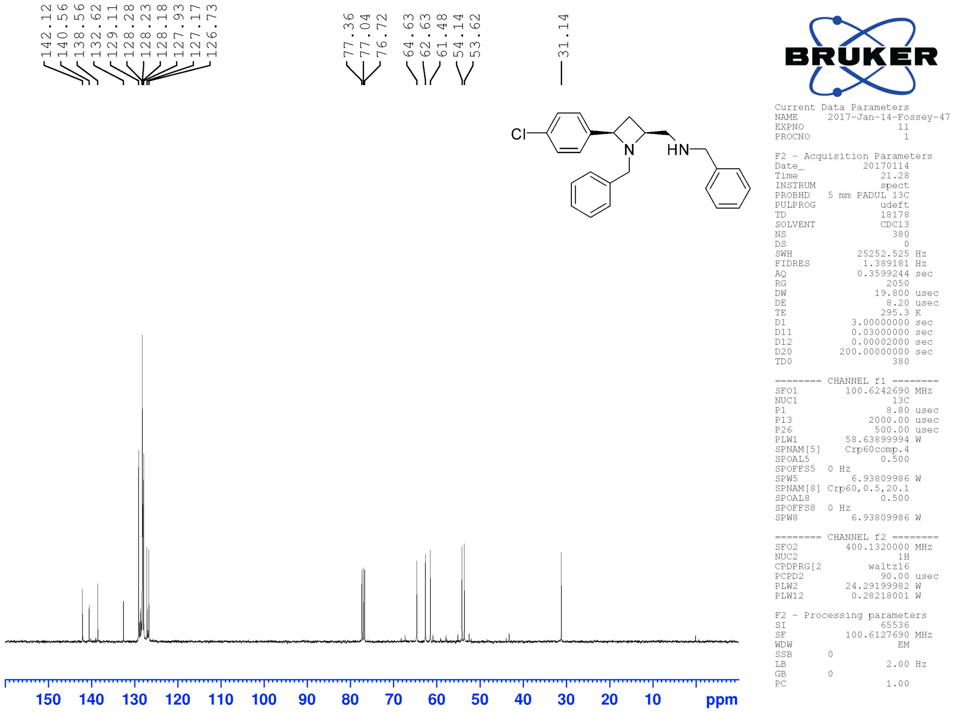

Proton NMR Spectrum of ***1m***

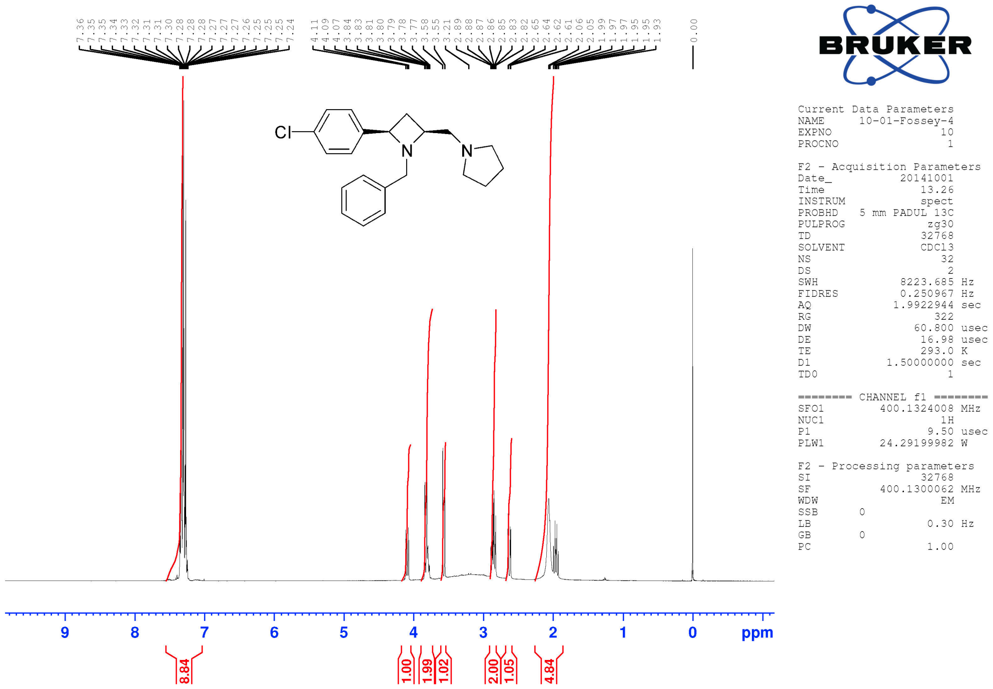

Carbon NMR Spectrum of ***1m***

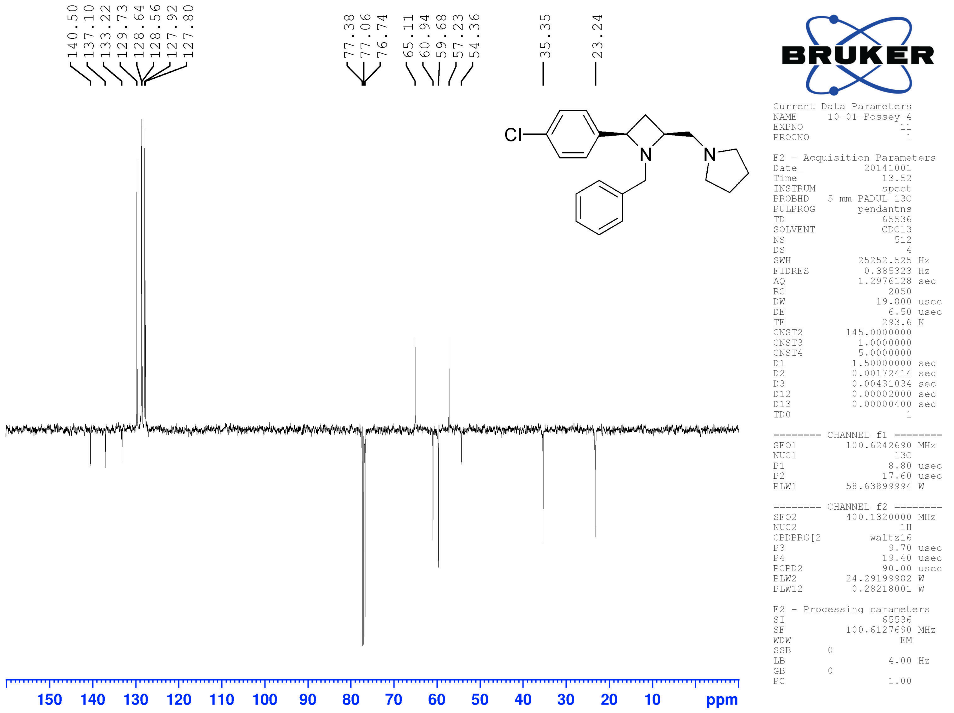

*Proton NMR Spectrum of **1n***

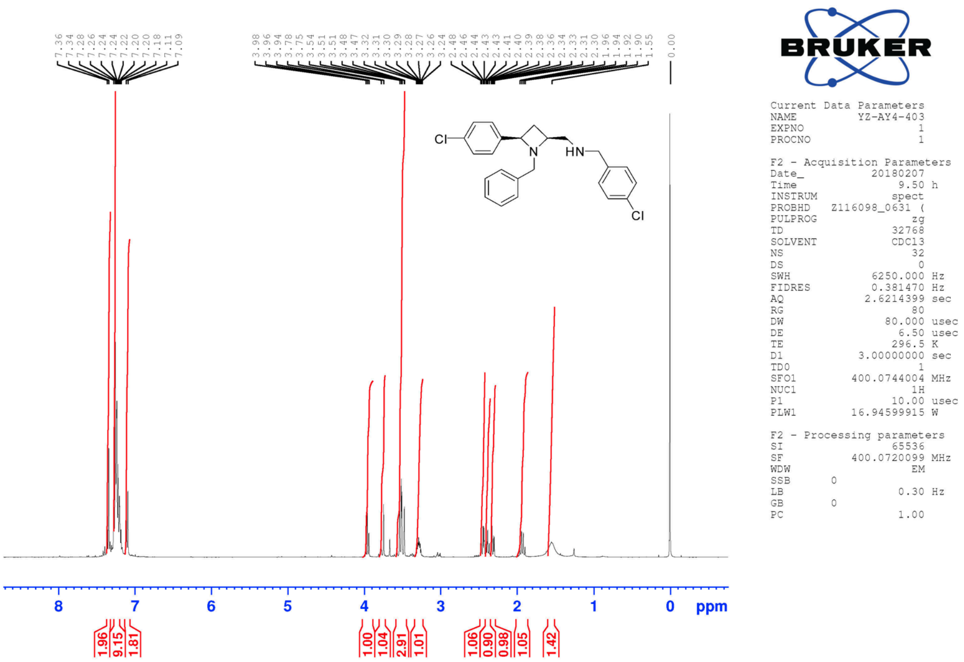

*Carbon NMR Spectrum of **1n***

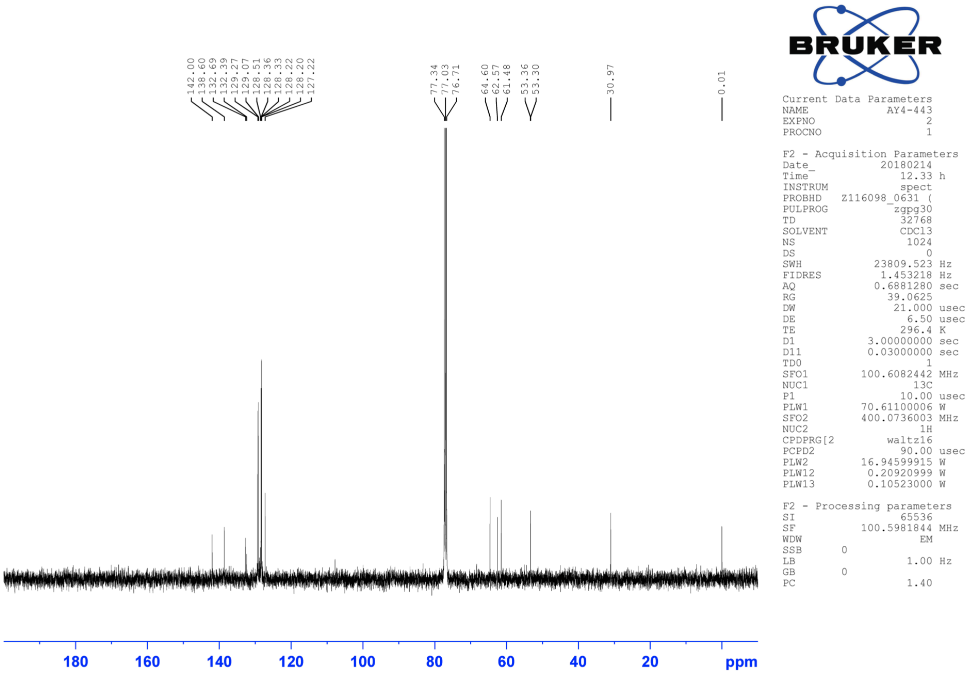

*Proton NMR Spectrum of **1o***

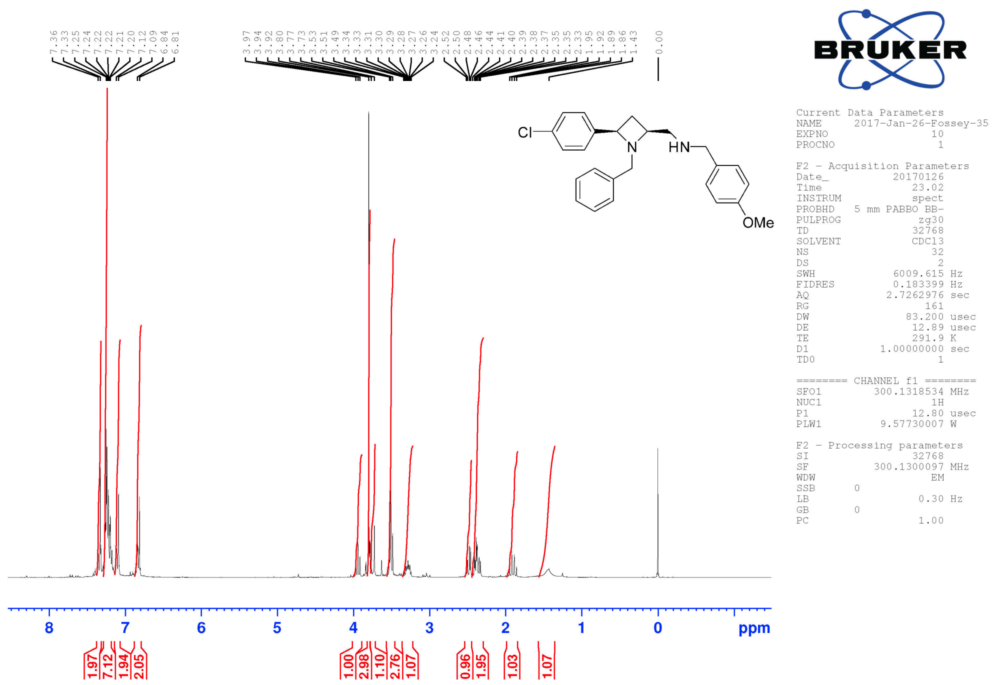

*Carbon NMR Spectrum of **1o***

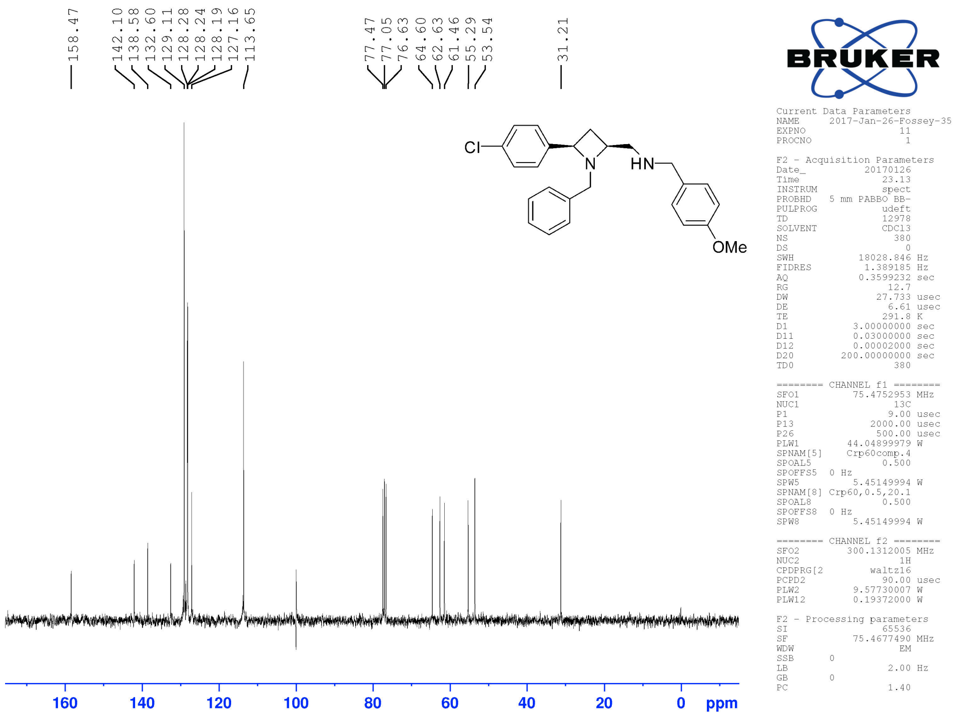

Proton NMR Spectrum of **(rac)-*6a***

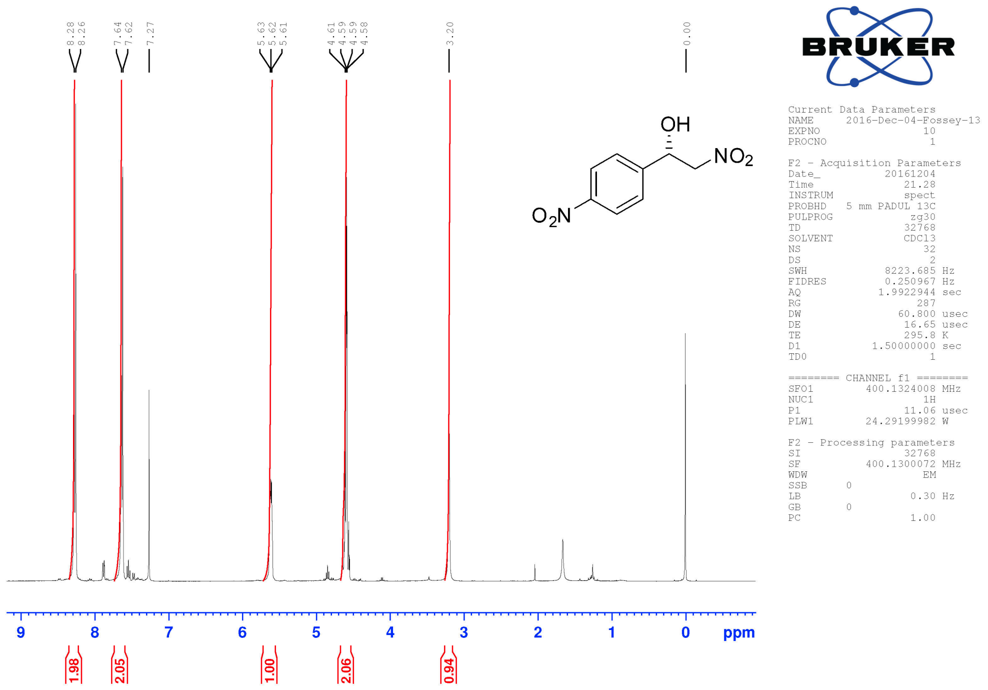

Carbon NMR Spectrum of **(rac)-*6a***

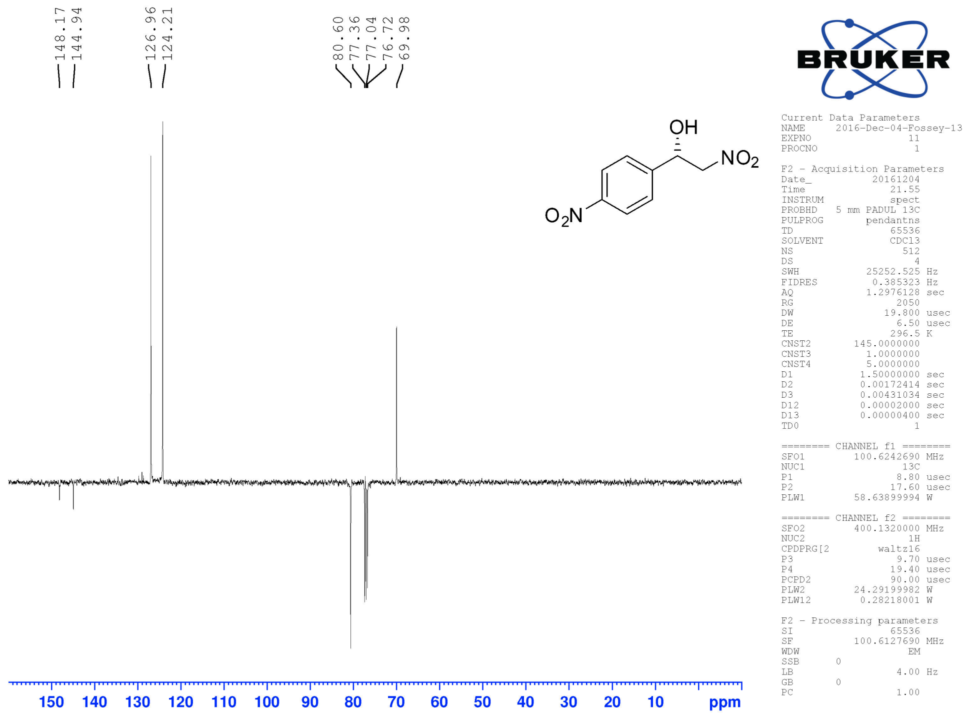

Proton NMR Spectrum of **(rac)-*6b***

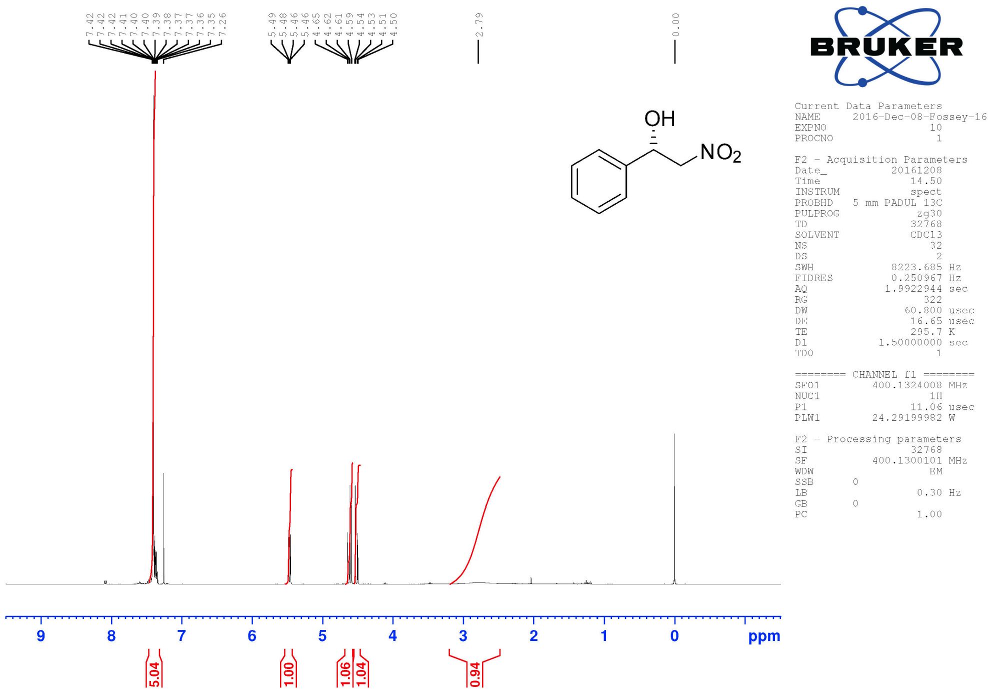

Carbon NMR Spectrum of **(rac)-*6b***

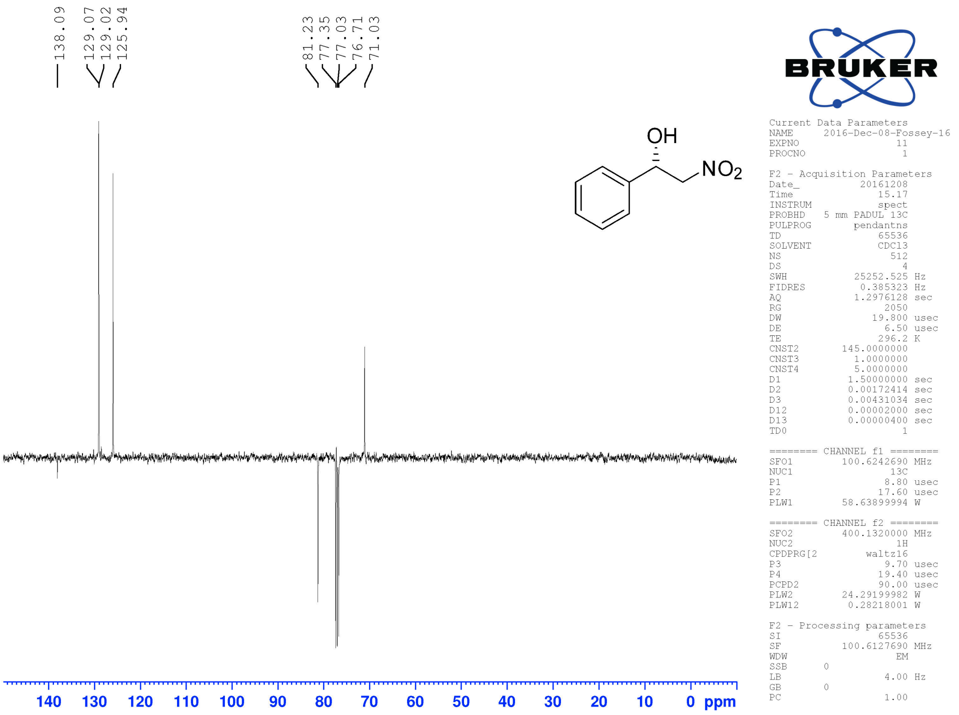

Proton NMR Spectrum of **(rac)-*6c***

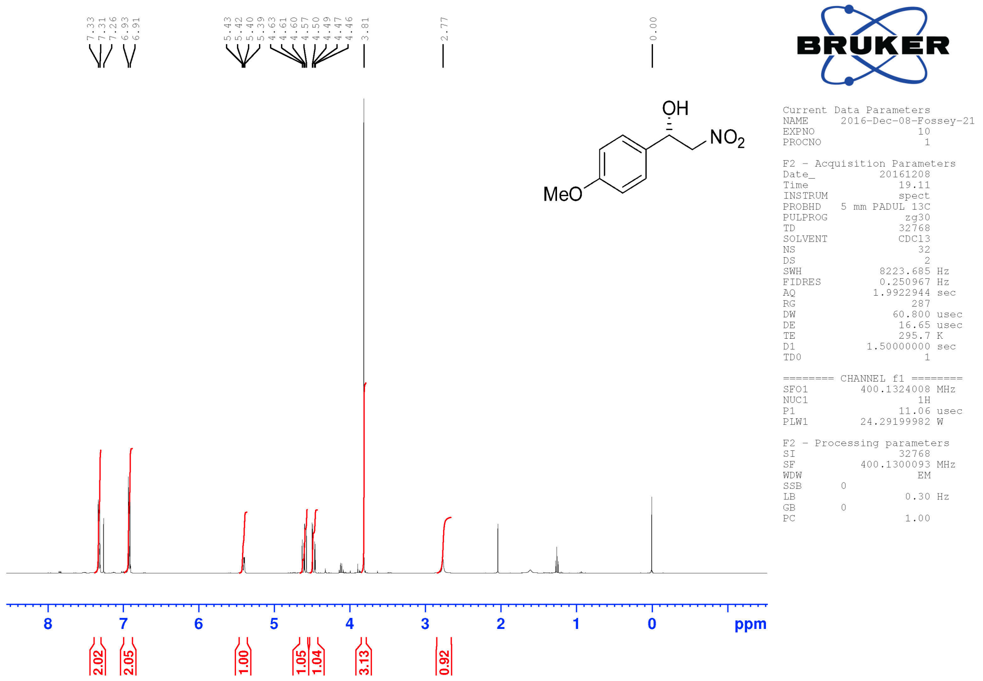

Carbon NMR Spectrum of **(rac)-*6c***

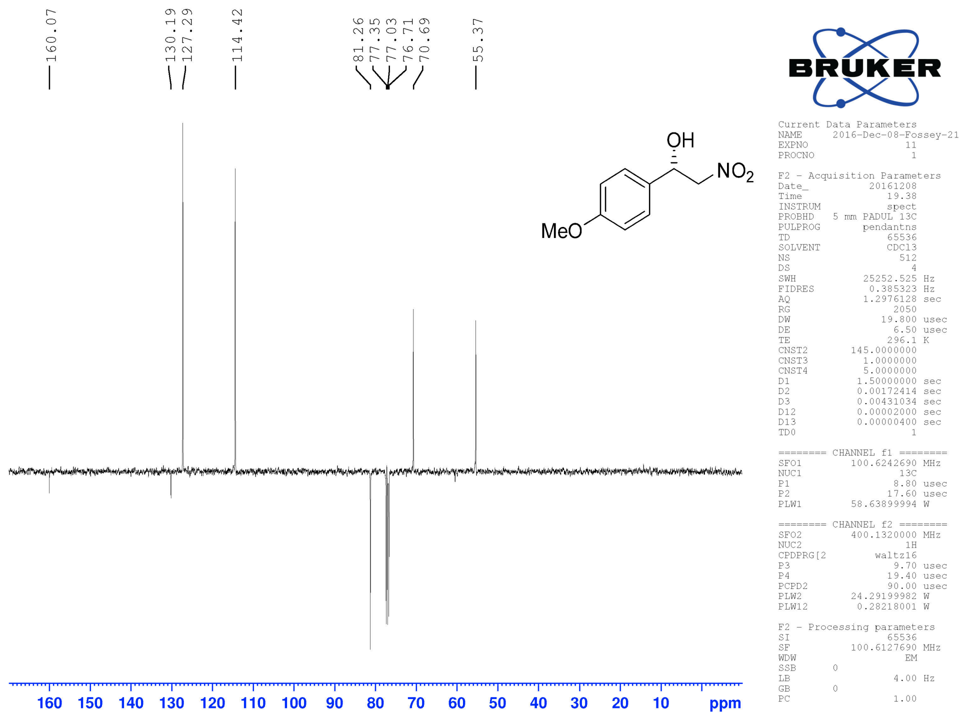

Proton NMR Spectrum of **(rac)-*6d***

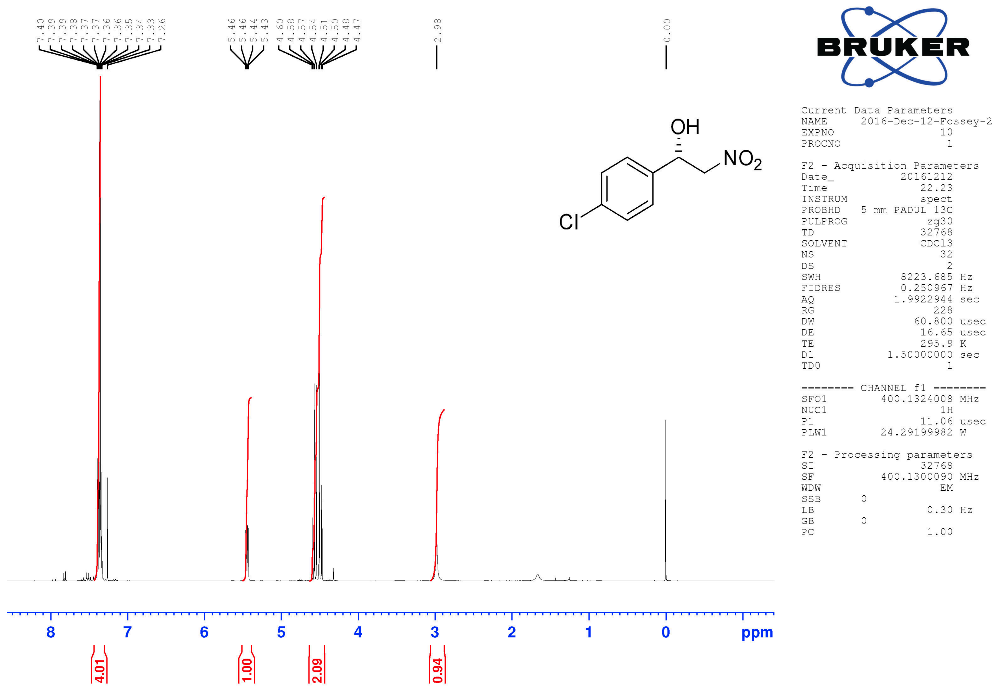

Carbon NMR Spectrum of **(rac)-*6d***

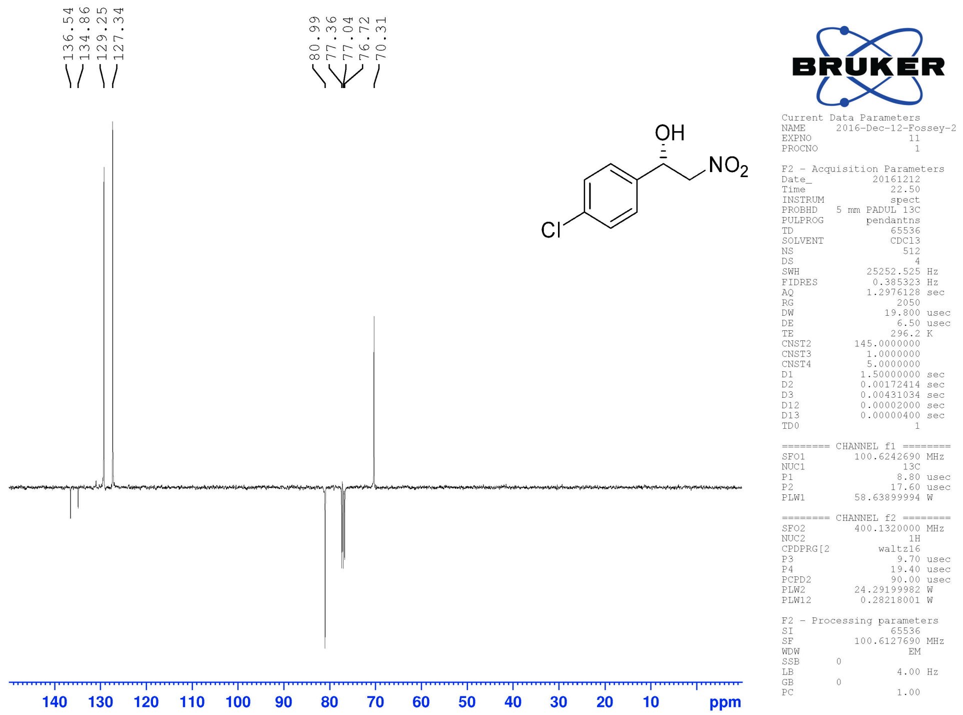

Proton NMR Spectrum of **(rac)-*6e***

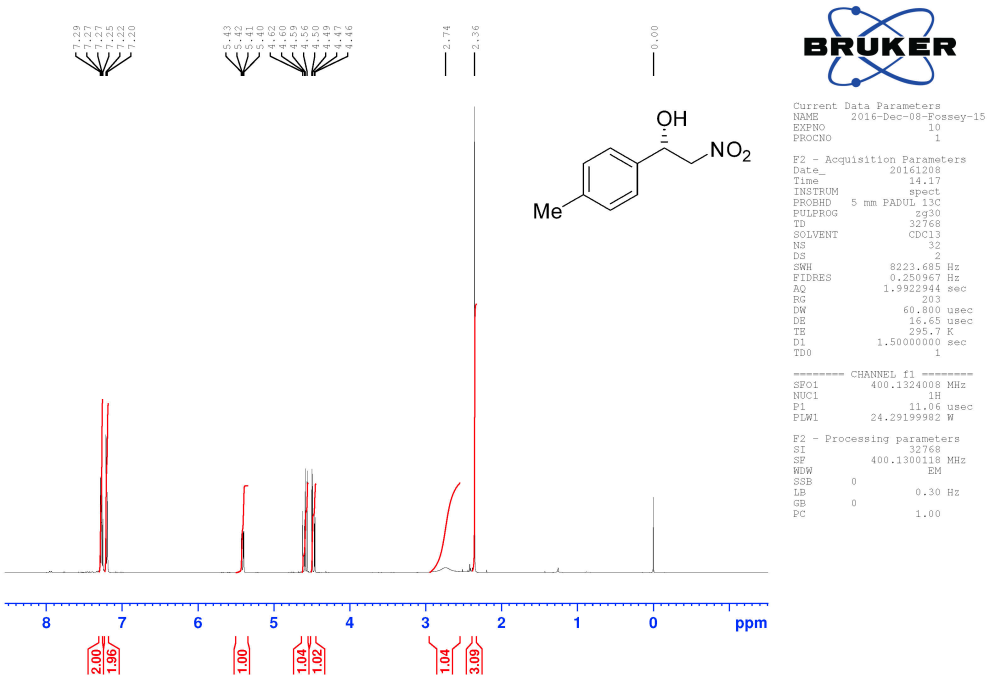

Carbon NMR Spectrum of **(rac)-*6e***

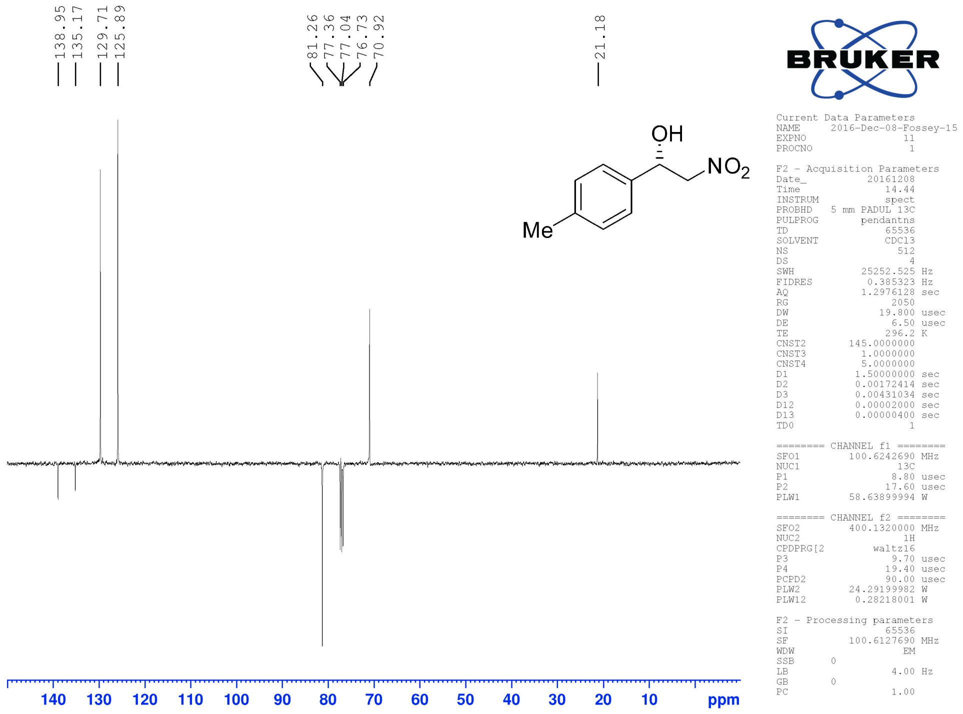

Proton NMR Spectrum of **(rac)-*6f***

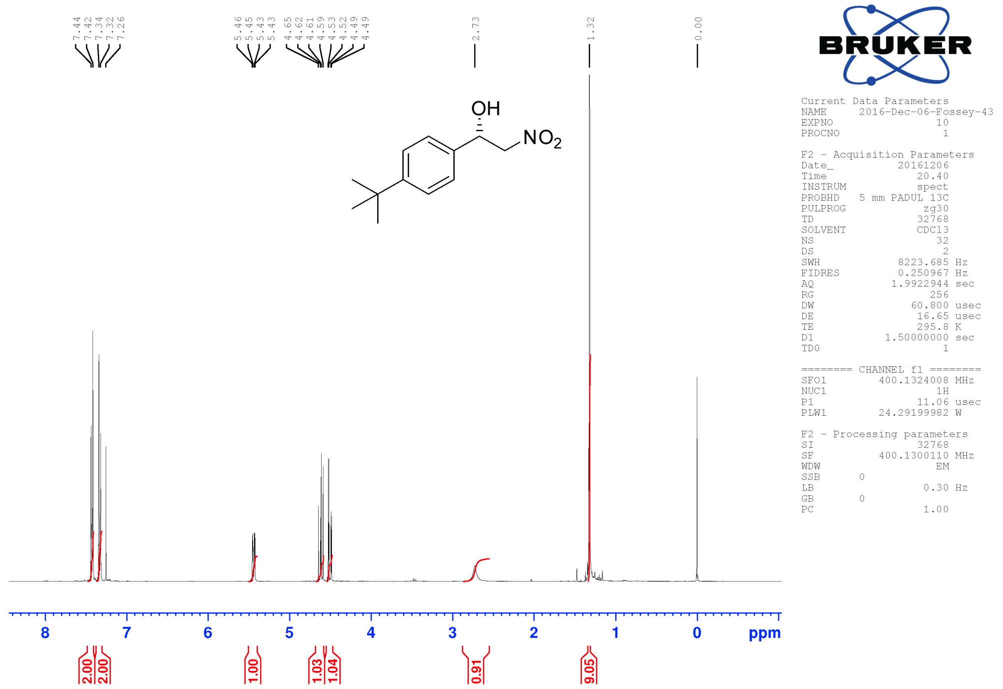

Carbon NMR Spectrum of **(rac)-*6f***

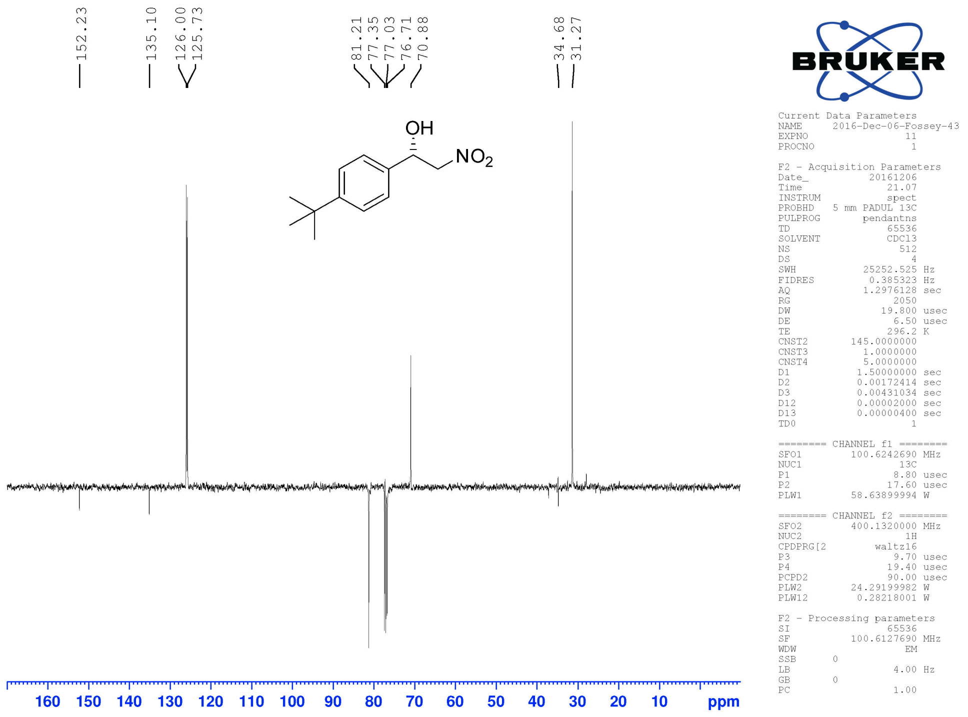

Proton NMR Spectrum of **(rac)-6g**

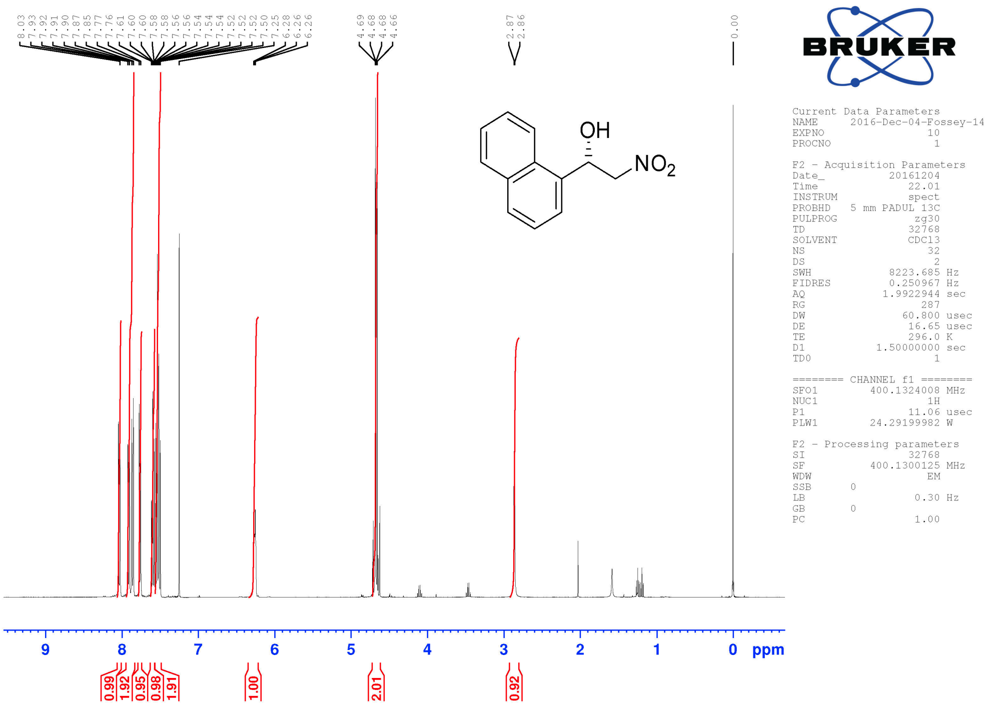

Carbon NMR Spectrum of **(rac)-6g**

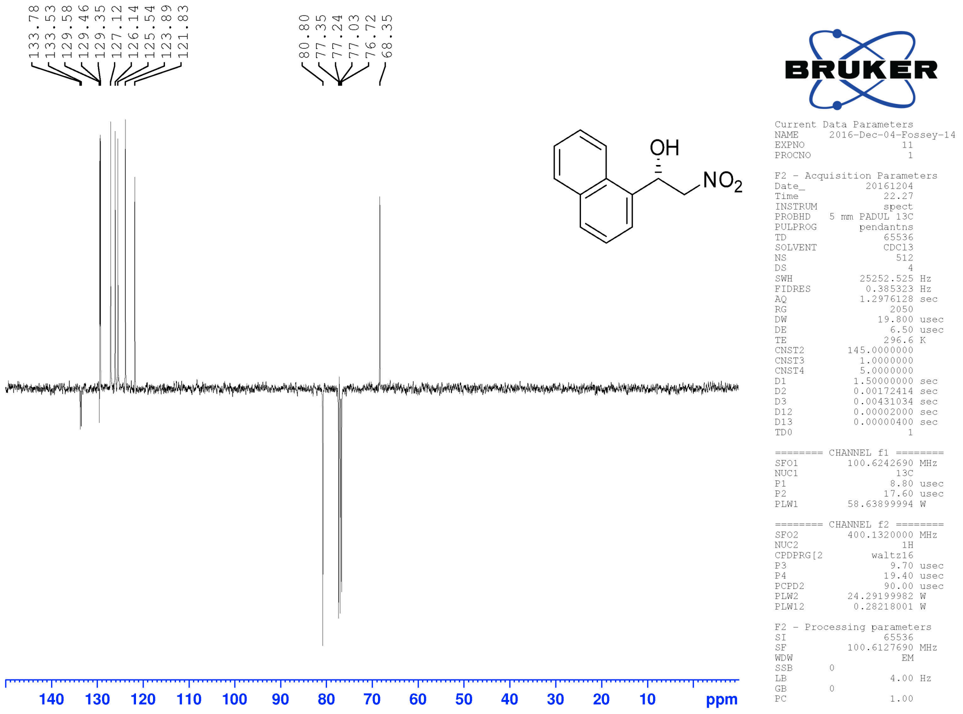

Proton NMR Spectrum of **(rac)-*6h***

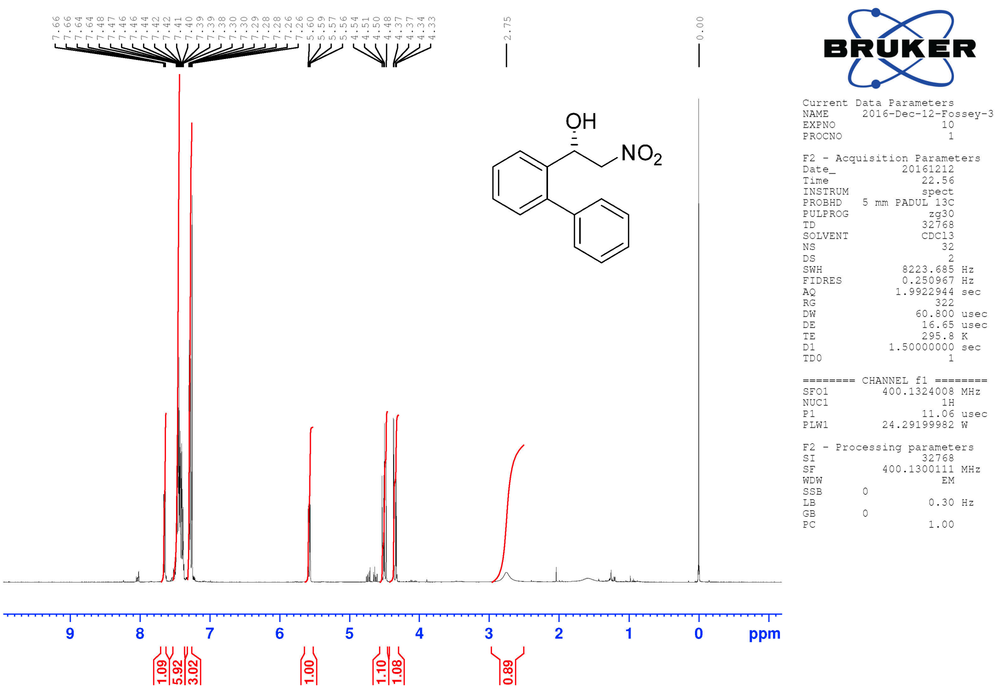

Carbon NMR Spectrum of **(rac)-*6h***

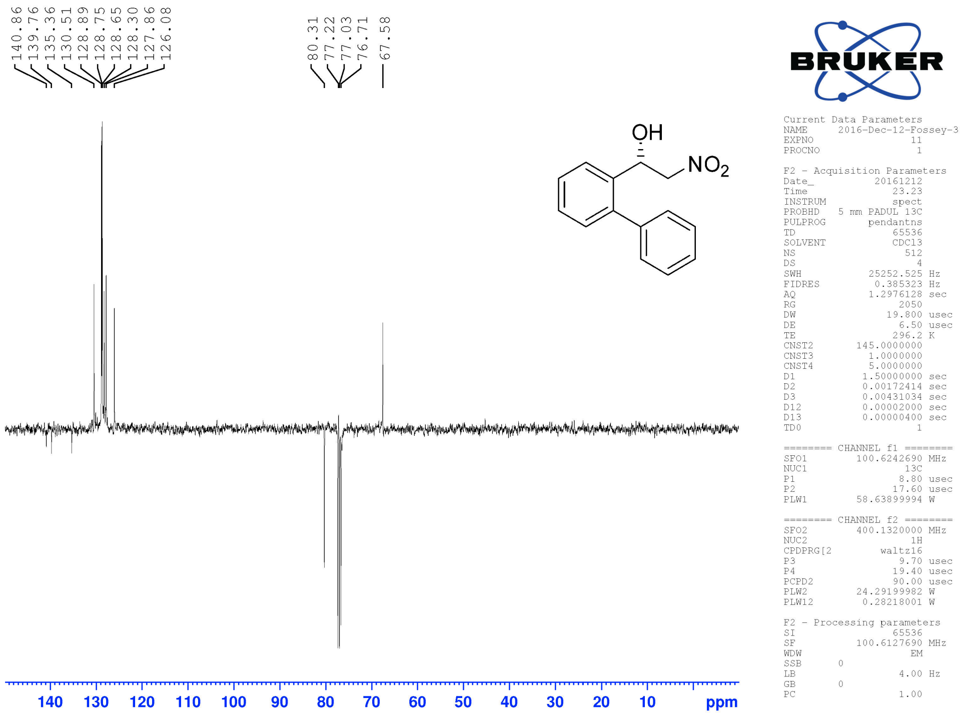

Proton NMR Spectrum of **(rac)-*6i***

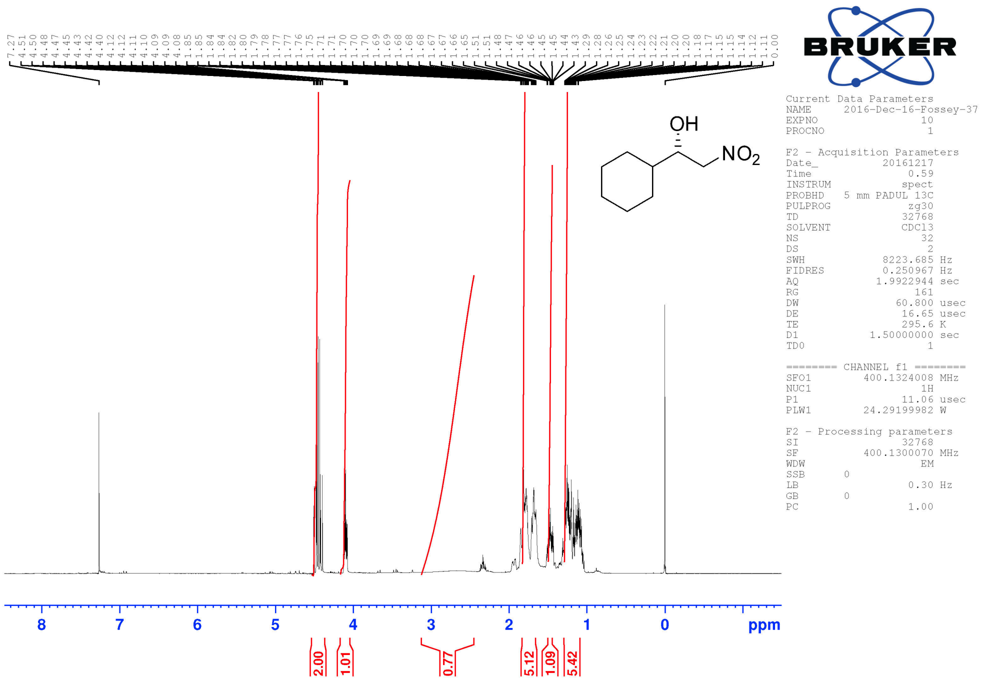

Carbon NMR Spectrum of **(rac)-*6i***

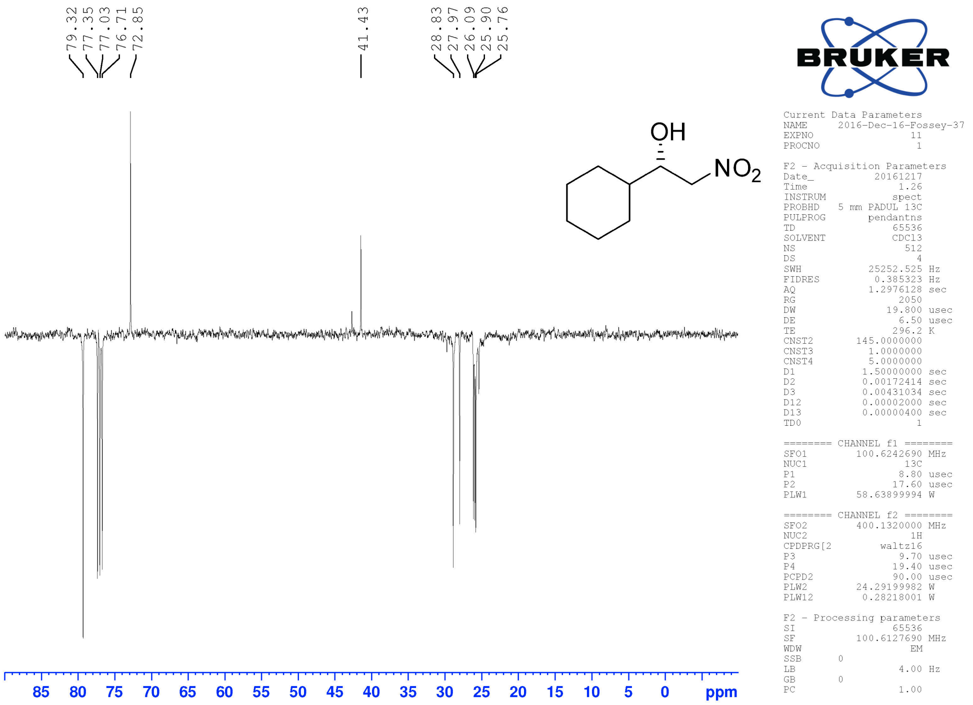

Proton NMR Spectrum of **(rac)-6j**

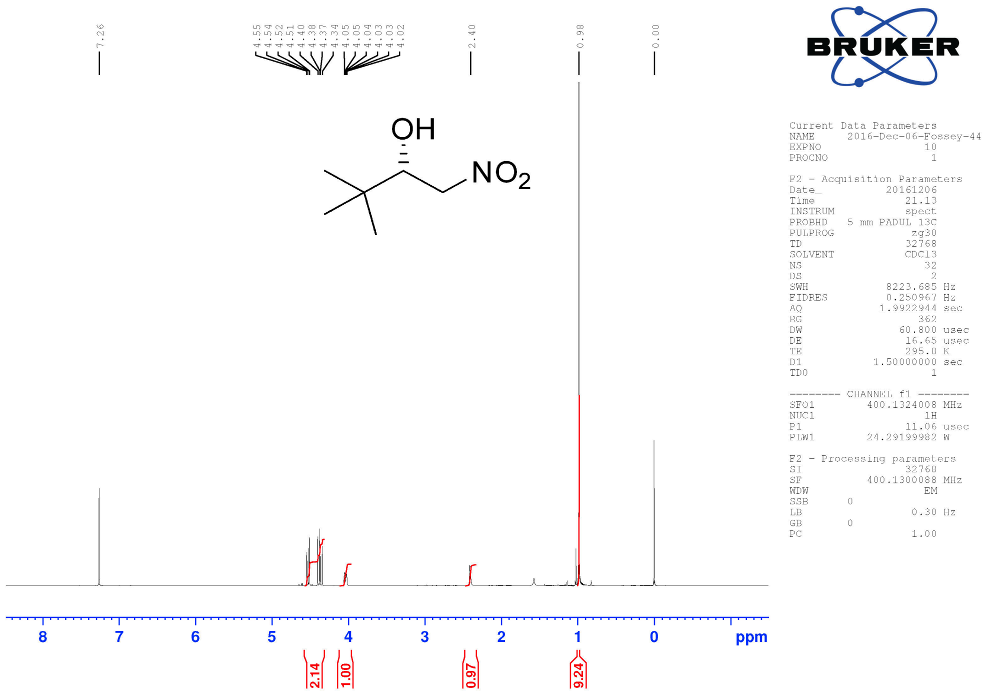

Carbon NMR Spectrum of **(rac)-6j**

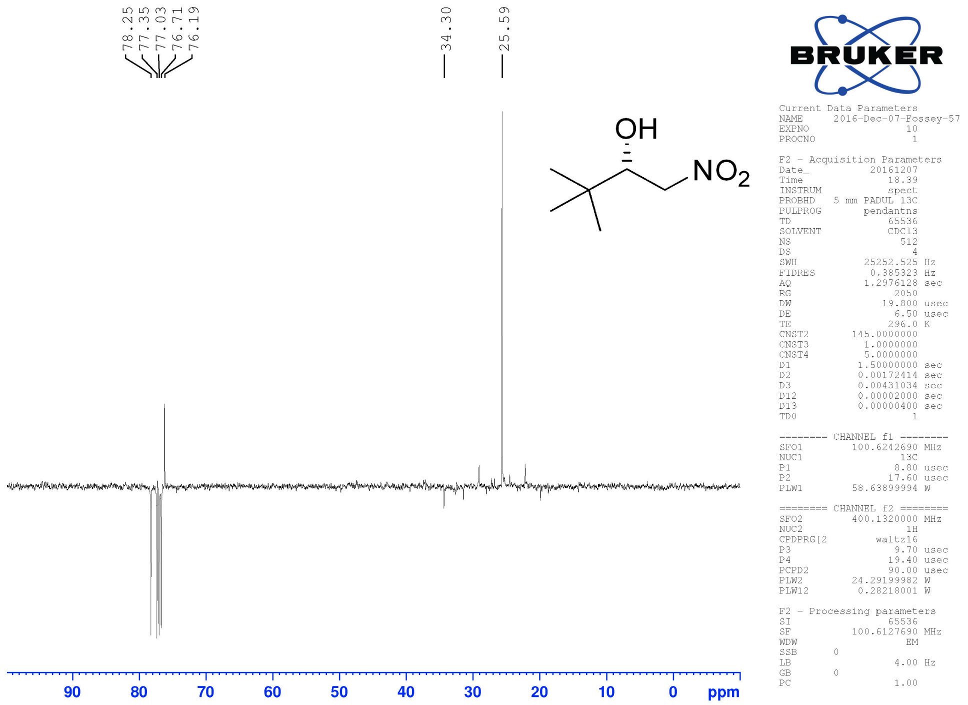

Computational data tables

This section includes the geometry of all structures reported in the main text. In addition, where alternative conformations have been explored and found to be higher in energy, those structures are included here. Where complexes of alternative ligand geometries led to dissociation from the metal, they were high in energy and were terminated before completion and are therefore not included.

Acetate anion

SCF energy= -228.401395147
Zero-point correction= 0.049059
Thermal correction to Energy= 0.053465
Thermal correction to Enthalpy= 0.054409
Thermal correction to Gibbs Free Energy= 0.021712
SCF energy+Solvation free energy= -228.492104536

Coordinates:
8 -0.693327 1.15978 -0.000879
6 -0.21917 0.001691 0.003895
8 -0.802185 -1.10401 -0.000955
6 1.3479 -0.05345 0.001292
1 1.72047 -1.07832 0.094554
1 1.74344 0.559525 0.820039
1 1.72783 0.383199 -0.931049

Benzaldehyde

SCF energy= -345.419894224
Zero-point correction= 0.111324
Thermal correction to Energy= 0.117589
Thermal correction to Enthalpy= 0.118533
Thermal correction to Gibbs Free Energy= 0.080818
SCF energy+Solvation free energy= -345.424933960

Coordinates:
6 -1.73279 -1.05617 0.000023
6 -0.361093 -1.29039 0.000012
6 0.528747 -0.216803 -0.00002
6 0.050211 1.09602 -0.000033
6 -1.31819 1.32903 -0.000012
6 -2.20815 0.25307 0.000013
1 -2.42858 -1.8891 0.000042
1 0.024576 -2.30768 0.000022
1 0.768526 1.91049 -0.000058
1 -1.69755 2.34606 -0.000019
1 -3.27817 0.438064 0.000028
6 1.98984 -0.470579 -0.000045
1 2.27908 -1.54279 -0.000262
8 2.83007 0.397482 0.000077

Nitromethane anion

SCF energy= -244.301524468

Zero-point correction= 0.036834
Thermal correction to Energy= 0.040701
Thermal correction to Enthalpy= 0.041645
Thermal correction to Gibbs Free Energy= 0.010915
SCF energy+Solvation free energy= -244.390635638

Coordinates:
6 -1.28536 -1.6e-05 0.000004
1 -1.78911 0.953148 0.000065
1 -1.78908 -0.953192 -0.000043
7 0.046699 0.0 -0.000024
8 0.68523 -1.10136 0.000013
8 0.685199 1.10137 0.000002

*Free Product **6b***

SCF energy= -589.751417985
Zero-point correction= 0.152302
Thermal correction to Energy= 0.162524
Thermal correction to Enthalpy= 0.163468
Thermal correction to Gibbs Free Energy= 0.115151

Coordinates:
6 3.04121 -1.01636 0.326127
6 1.69422 -1.16513 0.006065
6 0.907717 -0.056499 -0.29918
6 1.49886 1.20816 -0.293045
6 2.84686 1.36565 0.022633
6 3.6236 0.250775 0.337548
1 3.64649 -1.8892 0.563815
1 1.18569 -2.12546 -0.041346
1 0.895726 2.07895 -0.552067
1 3.2959 2.35693 0.019695
1 4.67663 0.370305 0.581897
6 -0.586049 -0.323276 -0.642301
1 -0.829359 0.392425 -1.48327
6 -1.3666 0.374092 0.599802
1 -1.18373 -0.21774 1.49503
1 -1.12303 1.43046 0.72232
7 -2.79864 0.301254 0.30613
8 -3.25874 1.13852 -0.466506
8 -3.44913 -0.612177 0.786441
8 -0.908717 -1.56759 -0.794294

*Cu[**1pC**-(OAc)$_2$]*

SCF energy= -1230.37463809
Zero-point correction= 0.395809
Thermal correction to Energy= 0.420540
Thermal correction to Enthalpy= 0.421484
Thermal correction to Gibbs Free Energy= 0.340814
SCF energy+Solvation free energy= =
SCF energy+Solvation free energy= =
SCF energy+Solvation free energy= =
SCF energy+Solvation free energy= =

SCF energy+Solvation free energy= =
SCF energy+Solvation free energy= =

Coordinates:
6 -1.41423 -2.5272 0.330615
1 -0.792093 -2.76275 1.20004
6 -0.508246 -2.32516 -0.89946
7 -2.15752 -1.30974 0.664631
1 -2.82522 -1.01661 -0.061399
7 -0.222381 -0.91116 -1.27692
29 -0.815472 0.274155 0.635542
8 -1.8809 1.6422 -0.184083
8 0.536724 1.48323 1.61086
1 -0.911655 -2.86261 -1.76981
6 -3.07967 1.53523 -0.644839
8 -3.73007 0.485797 -0.738538
6 -3.67967 2.84971 -1.11004
1 -3.03805 3.28708 -1.87998
1 -3.70405 3.55238 -0.272921
1 -4.68459 2.69491 -1.50174
6 -0.979846 -0.393605 -2.41381
1 -2.03961 -0.328464 -2.15311
1 -0.859484 -1.04269 -3.29555
1 -0.627793 0.611474 -2.66202
6 -2.88036 -1.41495 1.93555
1 -3.48887 -0.516406 2.06168
1 -2.15881 -1.48506 2.75526
1 -3.5359 -2.29482 1.96062
1 -2.10069 -3.36939 0.168056
6 1.00585 -2.57117 -0.741697
6 1.19382 -1.27096 -1.5472
1 1.3278 -2.44623 0.294424
1 1.4092 -3.48815 -1.17217
1 1.31227 -1.50265 -2.61852
6 2.24875 -0.282334 -1.12816
6 3.55508 -0.730461 -0.908621
6 1.96517 1.07494 -0.986426
6 4.56183 0.164129 -0.560798
1 3.7806 -1.78974 -1.0117
6 2.96893 1.97046 -0.619984
1 0.952673 1.44378 -1.13419
6 4.26902 1.52044 -0.413922
1 5.57222 -0.197564 -0.395215
1 2.7237 3.01906 -0.486206
1 5.05034 2.21891 -0.130697
6 1.10991 0.432573 2.00645
8 0.56646 -0.695205 1.75206
6 2.44319 0.482003 2.69135
1 2.51192 -0.300364 3.44892
1 2.61166 1.4668 3.12698
1 3.20767 0.29481 1.92807

*Cu[**1p**C-(OAc)(O_2NCH_2)] – lowest energy conformation*

SCF energy= -1246.27584727
Zero-point correction= 0.383170
Thermal correction to Energy= 0.406548
Thermal correction to Enthalpy= 0.407493
Thermal correction to Gibbs Free Energy= 0.329897
SCF energy+Solvation free energy= -1246.28830458

Coordinates:
6 1.36606 -2.51984 -0.408913
1 0.713633 -2.71835 -1.26638
6 0.503721 -2.34895 0.85684
7 2.11413 -1.30097 -0.724627
1 2.79712 -1.03149 -0.003446
7 0.230248 -0.943632 1.26956
29 0.786443 0.291312 -0.631599
8 1.87926 1.63218 0.200315
8 -0.557266 1.52543 -1.5142
1 0.938978 -2.90505 1.69949
6 3.08446 1.50185 0.63593
8 3.72724 0.444614 0.689601
6 3.70227 2.79408 1.13944
1 3.61047 3.56825 0.374057
1 4.74789 2.63944 1.4044
1 3.145 3.1368 2.01648
6 1.00704 -0.455737 2.40557
1 2.06331 -0.39253 2.12981
1 0.895757 -1.12133 3.27637
1 0.666929 0.546828 2.67936
6 2.81131 -1.37599 -2.01183
1 3.42072 -0.476483 -2.12592
1 2.07327 -1.4206 -2.81859
1 3.46233 -2.25753 -2.07288
1 2.04438 -3.3773 -0.299474
6 -1.01377 -2.59563 0.738333
6 -1.18758 -1.29527 1.54653
1 -1.36309 -2.47393 -0.289359
1 -1.40451 -3.51323 1.17905
1 -1.30634 -1.52206 2.61859
6 -2.23271 -0.299884 1.11729
6 -3.51919 -0.747855 0.804119
6 -1.95275 1.06366 1.04649
6 -4.51099 0.152502 0.430997
1 -3.74074 -1.81197 0.850739
6 -2.94089 1.96576 0.656805
1 -0.951436 1.42989 1.26127
6 -4.22175 1.51456 0.353989
1 -5.50656 -0.208364 0.190434
1 -2.69807 3.02083 0.579789
1 -4.99144 2.21868 0.052376
8 -0.612382 -0.643176 -1.72968
6 -2.39484 0.574691 -2.49311
1 -2.86782 -0.346771 -2.79193

1 -2.82847 1.55557 -2.60122
7 -1.23372 0.497825 -1.94576

*Cu[**1pC**-(OAc)(O_2NCH_2)] – higher energy conformation*

SCF energy= -1246.26960401
Zero-point correction= 0.383875
Thermal correction to Energy= 0.407899
Thermal correction to Enthalpy= 0.408843
Thermal correction to Gibbs Free Energy= 0.330061
SCF energy+Solvation free energy= -1246.28444419

Coordinates:
6 1.13227 -2.34399 -1.26628
1 0.519674 -2.01215 -2.1075
6 0.215216 -2.60344 -0.054257
7 2.09694 -1.27464 -0.995084
7 -0.019766 -1.44739 0.86116
29 0.918899 0.35594 -0.29981
8 2.0712 0.996688 1.11647
8 -0.278671 1.96376 -0.637229
1 0.561047 -3.47303 0.523008
6 3.06649 1.53438 0.488864
8 3.22008 1.43821 -0.734714
6 4.04614 2.30133 1.35305
1 4.33165 1.69964 2.21945
1 3.55296 3.20245 1.72905
1 4.92591 2.58216 0.774451
6 0.614815 -1.49434 2.17781
1 1.64596 -1.1418 2.12406
1 0.574045 -2.5083 2.60717
1 0.078073 -0.813894 2.84495
1 1.63887 -3.27621 -1.55467
6 -1.29699 -2.66792 -0.349558
6 -1.47707 -1.74854 0.870671
1 -1.54906 -2.14963 -1.27805
1 -1.77343 -3.64781 -0.308133
1 -1.71962 -2.34479 1.76522
6 -2.41675 -0.575748 0.774521
6 -3.67615 -0.75088 0.194338
6 -2.07092 0.675255 1.28225
6 -4.57827 0.305549 0.128473
1 -3.94946 -1.72376 -0.208848
6 -2.96718 1.73889 1.20255
1 -1.08572 0.839268 1.71088
6 -4.22371 1.55602 0.632773
1 -5.55417 0.156209 -0.323678
1 -2.67095 2.71321 1.5781
1 -4.92258 2.38501 0.574547
8 -0.42189 0.105207 -1.77028
6 -1.99997 1.72398 -2.1252
1 -2.47053 1.07256 -2.8437
1 -2.35656 2.70377 -1.85235
7 -0.938947 1.29503 -1.53745

1 2.42706 -0.86722 -1.86957
6 3.26316 -1.70531 -0.21837
1 3.93224 -0.853054 -0.098516
1 3.79453 -2.52408 -0.720897
1 2.94222 -2.05398 0.76536

*Cu[**1p**C-(OAc)(O_2NCH_2)] – higher energy conformation*

SCF energy= -1246.24344268
Zero-point correction= 0.383450
Thermal correction to Energy= 0.407915
Thermal correction to Enthalpy= 0.408859
Thermal correction to Gibbs Free Energy= 0.326021
SCF energy+Solvation free energy= =
SCF energy+Solvation free energy= =

Coordinates:
6 -0.808244 1.45652 -1.18966
7 -1.2024 1.62193 0.220163
29 1.90222 -0.025516 0.00109
8 1.4325 -1.74821 0.793651
8 2.69328 1.04618 1.52327
6 2.52044 -2.36274 0.479255
8 3.39115 -1.81539 -0.22226
6 2.68665 -3.77056 0.999003
1 1.81393 -4.36931 0.725913
1 2.73379 -3.74144 2.091
1 3.59654 -4.21843 0.600955
6 -0.773504 0.73611 1.2796
1 0.263624 0.956135 1.56005
1 -0.868887 -0.340157 1.05879
1 -1.3824 0.939896 2.16597
6 -2.19011 2.03104 -1.58058
6 -2.63529 1.76801 -0.126186
1 -2.14671 3.09682 -1.8073
1 -2.73573 1.51742 -2.37452
8 2.48359 1.79829 -0.511317
6 3.45433 3.15674 1.05626
1 3.5744 3.91077 0.29526
1 3.76485 3.25806 2.08373
7 2.90257 2.04734 0.712118
1 -3.0811 2.63185 0.38325
6 -3.51994 0.563909 0.140724
6 -4.12697 0.459376 1.39704
6 -3.72862 -0.469132 -0.777084
6 -4.90242 -0.644879 1.73554
1 -3.98986 1.26239 2.11795
6 -4.50659 -1.57649 -0.445615
1 -3.29473 -0.41608 -1.77131
6 -5.09245 -1.67052 0.812944
1 -5.36166 -0.703202 2.71757
1 -4.65817 -2.36422 -1.17726
1 -5.6982 -2.53331 1.07113
6 -0.558934 0.032284 -1.71043

1 -0.995263 -0.073601 -2.71606
1 -1.03487 -0.715846 -1.06767
7 0.878188 -0.303448 -1.77801
1 0.056299 2.08082 -1.45537
6 1.09157 -1.65185 -2.32947
1 2.16209 -1.84427 -2.40645
1 0.609785 -1.76005 -3.30879
1 0.660183 -2.37115 -1.62966
1 1.33532 0.382211 -2.38339

*Cu[**1p**C-(OAc)(O_2NCH_2)] – higher energy conformation*

SCF energy= -1246.24383978
Zero-point correction= 0.383476
Thermal correction to Energy= 0.407923
Thermal correction to Enthalpy= 0.408867
Thermal correction to Gibbs Free Energy= 0.326315
SCF energy+Solvation free energy= -1246.25866679

Coordinates:
6 -0.756846 1.41262 -1.13013
7 -1.1827 1.52951 0.277092
29 1.88615 -0.108338 0.150997
8 1.50968 -1.88049 0.869296
8 2.74533 0.843685 1.69543
6 2.39086 -2.55927 0.220677
8 3.08991 -2.04 -0.669367
6 2.54949 -4.0121 0.604409
1 1.57064 -4.47148 0.755393
1 3.08765 -4.06162 1.55573
1 3.11715 -4.54682 -0.156735
6 -0.799597 0.596332 1.31242
1 0.219732 0.808833 1.66064
1 -0.871497 -0.469556 1.03673
1 -1.45412 0.749517 2.176
6 -2.1167 2.02823 -1.53238
6 -2.60212 1.73101 -0.097169
1 -2.04915 3.09866 -1.72921
1 -2.65395 1.54682 -2.35204
8 2.45774 1.74617 -0.265649
6 3.45822 2.99824 1.3718
1 3.53941 3.81289 0.670326
1 3.79995 3.02369 2.39405
7 2.91301 1.9102 0.957501
1 -3.03057 2.59332 0.429722
6 -3.53727 0.553356 0.110911
6 -4.17467 0.431967 1.35068
6 -3.77263 -0.436473 -0.847451
6 -5.00403 -0.64796 1.63501
1 -4.02017 1.2043 2.10102
6 -4.60472 -1.51907 -0.570487
1 -3.31795 -0.366229 -1.83124
6 -5.21945 -1.63139 0.672808
1 -5.48583 -0.719659 2.60527

1 -4.77565 -2.2736 -1.3323
1 -5.86767 -2.4749 0.888361
6 -0.53167 0.000605 -1.68866
1 -0.868302 -0.033065 -2.73655
1 -1.12137 -0.728989 -1.12575
7 0.882466 -0.420834 -1.62413
1 0.938092 -1.43685 -1.68879
6 1.71025 0.141567 -2.70611
1 1.27333 -0.076546 -3.68844
1 2.70621 -0.300394 -2.63501
1 1.79497 1.22222 -2.57722
1 0.125101 2.02814 -1.34771

*Cu[**1p**C-(OAc)(O_2NCH_2)] – higher energy conformation*

SCF energy= -1246.26804547
Zero-point correction= 0.384330
Thermal correction to Energy= 0.408119
Thermal correction to Enthalpy= 0.409063
Thermal correction to Gibbs Free Energy= 0.330818
SCF energy+Solvation free energy= =
SCF energy+Solvation free energy= =
SCF energy+Solvation free energy= =
SCF energy+Solvation free energy= =

Coordinates:
6 1.50853 -2.50187 0.135786
1 0.884913 -2.90719 -0.66738
6 0.611395 -2.08087 1.31549
7 2.22398 -1.35356 -0.424683
1 2.86097 -0.900956 0.238589
7 0.292907 -0.627548 1.39988
29 0.850522 0.167448 -0.729901
8 1.69528 1.79286 -0.144466
8 -0.557608 1.10147 -1.89348
1 1.03762 -2.42247 2.27004
8 3.62666 0.822724 0.428376
6 3.23652 2.98779 0.963326
1 2.54701 3.81272 0.896641
1 4.21473 3.03789 1.41256
6 1.05521 0.139985 2.3818
1 2.11317 0.148993 2.10169
1 0.949685 -0.292012 3.3893
1 0.70155 1.17447 2.39508
6 2.97418 -1.67697 -1.64186
1 3.55376 -0.796568 -1.9271
1 2.26938 -1.92941 -2.44008
1 3.65678 -2.52269 -1.48857
1 2.21311 -3.28542 0.44676
6 -0.89685 -2.38816 1.23248
6 -1.10874 -0.957165 1.76398
1 -1.23115 -2.48196 0.196516
1 -1.27313 -3.2091 1.8436
1 -1.20655 -0.97296 2.8622

6 -2.19755 -0.102211 1.1739
6 -3.4944 -0.620194 1.09346
6 -1.95657 1.19807 0.73438
6 -4.53473 0.150918 0.586607
1 -3.68539 -1.63687 1.43061
6 -2.99582 1.96627 0.210117
1 -0.951307 1.61344 0.768895
6 -4.28562 1.44928 0.141113
1 -5.53706 -0.262837 0.529554
1 -2.78616 2.96717 -0.153248
1 -5.09328 2.05011 -0.265367
6 -1.08861 -0.028344 -2.06752
8 -0.48261 -1.06239 -1.62519
6 -2.43795 -0.168477 -2.70625
1 -2.46564 -1.05568 -3.34132
1 -2.68758 0.728261 -3.27294
1 -3.16939 -0.297775 -1.8998
7 2.88756 1.85815 0.427391

*Cu[**1p**C-(OAc)(O_2NCH_2)] – higher energy conformation*

SCF energy= -1246.26725131
Zero-point correction= 0.383840
Thermal correction to Energy= 0.407856
Thermal correction to Enthalpy= 0.408801
Thermal correction to Gibbs Free Energy= 0.329848
SCF energy+Solvation free energy= -1246.28161774

Coordinates:
6 1.2313 -2.26758 -1.33289
1 0.653587 -1.91512 -2.18969
6 0.261209 -2.56104 -0.172384
7 2.17824 -1.20133 -0.9966
7 -0.021778 -1.44184 0.776549
29 1.01328 0.418436 -0.285883
8 2.11725 0.888391 1.26459
8 -0.271959 2.01635 -0.502587
1 0.577618 -3.45128 0.390786
8 3.10403 1.51313 -0.600176
6 4.01915 2.11683 1.40029
1 3.91252 2.07405 2.47145
1 4.80979 2.63223 0.880217
6 0.567896 -1.53983 2.11229
1 1.60538 -1.20314 2.10522
1 0.498817 -2.56713 2.50508
1 0.016421 -0.875253 2.78373
1 1.75484 -3.19045 -1.62086
6 -1.2356 -2.61668 -0.536113
6 -1.46935 -1.78601 0.735768
1 -1.45016 -2.03264 -1.43453
1 -1.70628 -3.59885 -0.587293
1 -1.70321 -2.45395 1.58139
6 -2.45781 -0.651468 0.711557
6 -3.73057 -0.869369 0.174692

6 -2.15327 0.596425 1.25129
6 -4.68567 0.142085 0.184756
1 -3.9712 -1.83979 -0.254426
6 -3.10431 1.61534 1.24951
1 -1.15906 0.794259 1.64261
6 -4.3731 1.38947 0.724723
1 -5.6701 -0.038984 -0.236146
1 -2.84113 2.58945 1.64926
1 -5.1131 2.18391 0.72493
6 -0.803918 1.38178 -1.45096
8 -0.293997 0.263903 -1.80763
6 -2.05297 1.87223 -2.12174
1 -1.97843 1.73034 -3.20181
1 -2.23357 2.91878 -1.87745
1 -2.88771 1.26575 -1.7516
7 3.11347 1.53572 0.678395
6 3.32181 -1.63395 -0.188597
1 2.96779 -2.02778 0.766077
1 3.96337 -0.771298 -0.003774
1 3.89473 -2.41958 -0.698284
1 2.53318 -0.76959 -1.84932

*Cu[**1pC**-(OAc)(O_2NCH_2)] – higher energy conformation*

SCF energy= -1246.24231877
Zero-point correction= 0.383133
Thermal correction to Energy= 0.407535
Thermal correction to Enthalpy= 0.408480
Thermal correction to Gibbs Free Energy= 0.326071
SCF energy+Solvation free energy= =
SCF energy+Solvation free energy= =

Coordinates:
6 -0.737081 1.37174 -1.20203
7 -1.13237 1.56855 0.205526
29 1.92557 -0.100655 0.027075
8 1.38074 -1.76503 0.924572
8 2.67979 1.14411 1.49309
8 3.31474 -1.83065 -0.111272
6 2.59505 -3.68802 1.00829
1 1.8128 -4.10853 1.61864
1 3.4992 -4.20093 0.723934
6 -0.764299 0.664506 1.27305
1 0.273699 0.840435 1.5807
1 -0.899616 -0.406614 1.04819
1 -1.38419 0.892183 2.14572
6 -2.08476 2.01072 -1.60912
6 -2.55342 1.78887 -0.15509
1 -1.98559 3.06985 -1.84925
1 -2.65067 1.51508 -2.40042
6 2.8921 1.97884 0.573733
8 2.55402 1.67197 -0.62083
6 3.54823 3.30158 0.845026

1 4.5669 3.27344 0.447359
1 3.58101 3.49653 1.91628
1 3.00742 4.09329 0.322923
7 2.45713 -2.46549 0.61298
1 -2.95453 2.6815 0.341512
6 -3.50557 0.638256 0.115559
6 -4.13943 0.58422 1.36159
6 -3.75151 -0.396447 -0.791113
6 -4.97652 -0.473875 1.70144
1 -3.97421 1.39021 2.0733
6 -4.59066 -1.45744 -0.45832
1 -3.29822 -0.380711 -1.77784
6 -5.20219 -1.50274 0.79061
1 -5.45573 -0.493028 2.67548
1 -4.76919 -2.24754 -1.18137
1 -5.85525 -2.33 1.0499
6 -0.551621 -0.068503 -1.70037
1 -0.967799 -0.166807 -2.71527
1 -1.08442 -0.779812 -1.0609
7 0.866504 -0.47795 -1.7199
1 0.158822 1.94924 -1.46803
1 1.36397 0.134485 -2.37002
6 1.02363 -1.87773 -2.14892
1 2.08617 -2.1221 -2.1877
1 0.550899 -2.05053 -3.12338
1 0.552101 -2.51354 -1.39561

*Cu[**1pC**-(OAc)(O_2NCH_2)] – higher energy conformation*

SCF energy= -1246.24111109
Zero-point correction= 0.383228
Thermal correction to Energy= 0.407685
Thermal correction to Enthalpy= 0.408629
Thermal correction to Gibbs Free Energy= 0.326587
SCF energy+Solvation free energy= -1246.25533406

Coordinates:
6 -0.724164 1.31229 -1.14158
7 -1.146 1.45059 0.266725
29 1.90083 -0.168406 0.13235
8 1.45957 -1.91695 0.926561
8 2.67793 0.942176 1.66791
8 3.17997 -1.93542 -0.439234
6 2.67188 -3.83271 0.729227
1 2.00738 -4.2786 1.45085
1 3.5086 -4.33143 0.268101
6 -0.811634 0.497427 1.30172
1 0.211898 0.663602 1.66179
1 -0.927065 -0.562995 1.02042
1 -1.46641 0.675522 2.16028
6 -2.05802 1.97792 -1.54852
6 -2.55567 1.71221 -0.111253
1 -1.94774 3.04298 -1.7551
1 -2.61408 1.51116 -2.36416

6 2.83021 1.87619 0.833378
8 2.46543 1.6776 -0.373946
6 3.43574 3.19235 1.22489
1 4.40281 3.29775 0.725333
1 3.57085 3.24315 2.3045
1 2.79225 4.00419 0.878709
7 2.46358 -2.59967 0.402903
1 -2.94574 2.59589 0.409842
6 -3.54133 0.577667 0.102611
6 -4.19701 0.497125 1.33605
6 -3.8049 -0.413979 -0.846342
6 -5.07169 -0.545995 1.62328
1 -4.02066 1.27191 2.07908
6 -4.68171 -1.45972 -0.566358
1 -3.3371 -0.37416 -1.82563
6 -5.31445 -1.53233 0.670778
1 -5.56754 -0.586131 2.5883
1 -4.87313 -2.21635 -1.3212
1 -5.99814 -2.34685 0.888208
6 -0.546503 -0.110727 -1.68722
1 -0.879425 -0.143981 -2.73615
1 -1.1626 -0.815036 -1.12102
7 0.853676 -0.574313 -1.60896
1 0.873449 -1.59363 -1.59552
6 1.68566 -0.108789 -2.7314
1 1.24349 -0.392695 -3.69439
1 2.67618 -0.557845 -2.63063
1 1.78642 0.976881 -2.67941
1 0.18167 1.89135 -1.3609

TS-1p*C*-II *– lowest energy conformation*

SCF energy= -1591.69252316
Zero-point correction= 0.496793
Thermal correction to Energy= 0.527769
Thermal correction to Enthalpy= 0.528714
Thermal correction to Gibbs Free Energy= 0.432821
SCF energy+Solvation free energy= -1591.70766979

Coordinates:
6 3.12232 -1.38501 -1.24864
1 2.36015 -1.48797 -2.03075
6 2.73998 -2.24114 -0.045305
7 3.12635 0.026917 -0.864983
1 3.76095 0.235013 -0.086515
7 1.64793 -1.63142 0.781382
29 1.29106 0.383339 -0.038505
8 1.75407 2.25309 0.46335
6 -0.741334 2.16822 -1.81775
7 -0.714729 0.858653 -2.07346
8 0.423819 0.232667 -1.9505
8 -1.73612 0.205691 -2.3297
1 3.61375 -2.4689 0.579734
6 2.84476 2.45676 1.11887

8 3.68392 1.5882 1.39504
6 3.0612 3.89596 1.55385
1 4.01965 4.00254 2.06098
1 2.25043 4.19713 2.22324
1 3.02176 4.55226 0.680166
6 2.02698 -1.20984 2.13926
1 2.69611 -0.343053 2.09844
1 2.52548 -2.0279 2.68031
1 1.11497 -0.930028 2.6754
6 3.40988 0.937608 -1.98134
1 3.38759 1.96171 -1.60368
1 2.62186 0.820722 -2.72839
1 4.08848 -1.70377 -1.66437
6 1.85125 -3.46399 -0.328622
6 0.897678 -2.93675 0.764552
1 1.3997 -3.42912 -1.32322
1 2.28343 -4.4493 -0.154086
1 1.09244 -3.4504 1.71606
6 -1.08649 1.68171 0.526442
8 -0.525465 0.571052 0.679236
1 0.213916 2.66156 -1.71111
1 -1.66145 2.67899 -2.054
6 -0.579974 -2.94006 0.499421
6 -1.42925 -3.63248 1.36472
6 -1.11024 -2.33554 -0.642854
6 -2.78895 -3.74354 1.08598
1 -1.02037 -4.09669 2.25943
6 -2.46857 -2.44957 -0.924558
1 -0.484005 -1.7452 -1.30842
6 -3.30801 -3.15943 -0.066457
1 -3.4378 -4.28897 1.76452
1 -2.86073 -1.95315 -1.80621
1 -4.36672 -3.25072 -0.292149
6 -2.55134 1.77772 0.487328
6 -3.17267 3.0294 0.542851
6 -3.31411 0.612612 0.382654
6 -4.55722 3.11537 0.499948
1 -2.56452 3.92876 0.613577
6 -4.70057 0.704057 0.336498
1 -2.80585 -0.344243 0.341758
6 -5.32052 1.95047 0.394475
1 -5.04526 4.08364 0.547519
1 -5.29659 -0.199733 0.253178
1 -6.40365 2.01904 0.357353
1 -0.501634 2.60078 0.648472
1 4.3895 0.730325 -2.4284

*Product arising from **TS-1pC-II** – lowest energy conformation*

SCF energy= -1591.71873258
Zero-point correction= 0.500883
Thermal correction to Energy= 0.531389
Thermal correction to Enthalpy= 0.532333
Thermal correction to Gibbs Free Energy= 0.438749

Coordinates:
6 3.03866 -1.89242 -0.762397
1 2.33675 -2.2693 -1.51571
6 2.58521 -2.33017 0.630692
7 3.03203 -0.432422 -0.859384
1 3.69076 -0.014808 -0.190044
7 1.44696 -1.4975 1.12939
29 1.32302 0.348098 0.004467
8 2.09595 2.20216 -0.103615
6 -0.762332 2.39382 -1.00446
7 -0.831014 1.24337 -1.93427
8 0.200054 0.575555 -2.08059
8 -1.87987 0.978482 -2.47187
1 3.42405 -2.32164 1.3361
6 3.1287 2.32567 0.651013
8 3.70647 1.37726 1.21082
6 3.63274 3.7454 0.833615
1 4.55099 3.75451 1.41998
1 2.86283 4.33334 1.34152
1 3.80136 4.20494 -0.143741
6 1.65169 -0.904584 2.46291
1 2.44902 -0.156683 2.40595
1 1.92118 -1.67668 3.19889
1 0.719177 -0.41364 2.75181
6 3.3054 0.073065 -2.20963
1 3.2481 1.16297 -2.18399
1 2.53508 -0.296943 -2.89138
1 4.02517 -2.31758 -0.995895
6 1.70669 -3.59554 0.66672
6 0.542972 -2.69913 1.1711
1 1.53015 -4.05306 -0.309775
1 2.02159 -4.36339 1.37429
1 0.276597 -2.92199 2.21021
6 -1.04691 1.88047 0.455512
8 -0.341826 0.75384 0.765204
1 0.254761 2.77735 -1.06812
1 -1.51415 3.10805 -1.33738
6 -0.709913 -2.64113 0.343976
6 -1.9052 -3.15107 0.851458
6 -0.69588 -2.11848 -0.949817
6 -3.06524 -3.13905 0.07805
1 -1.93189 -3.5535 1.8612
6 -1.85552 -2.07577 -1.71519
1 0.220228 -1.70767 -1.36383
6 -3.04439 -2.59309 -1.20191
1 -3.98869 -3.53925 0.485991
1 -1.83049 -1.61481 -2.69667
1 -3.95332 -2.55661 -1.79401
6 -2.54659 1.66735 0.620133
6 -3.41214 2.75702 0.726985
6 -3.06795 0.376833 0.641981
6 -4.78391 2.56201 0.848539
1 -3.00739 3.76833 0.724571

6 -4.44167 0.179166 0.761306
1 -2.37803 -0.458146 0.578979
6 -5.30296 1.26819 0.864392
1 -5.44798 3.41696 0.93616
1 -4.83655 -0.8332 0.77429
1 -6.3734 1.11269 0.961355
1 -0.75584 2.75461 1.07049
1 4.29164 -0.243608 -2.57036

TS-1p*C*-I *– lowest energy conformation*

SCF energy= -1591.69250351
Zero-point correction= 0.497680
Thermal correction to Energy= 0.528136
Thermal correction to Enthalpy= 0.529080
Thermal correction to Gibbs Free Energy= 0.435249
SCF energy+Solvation free energy= -1591.70610939

Coordinates:
6 -1.54062 -1.95537 -2.15991
1 -1.30576 -1.10544 -2.80563
6 -2.70469 -1.57505 -1.22632
7 -0.320307 -2.25892 -1.41028
1 -0.410571 -3.11426 -0.841589
7 -2.32271 -1.08015 0.131479
29 -0.01405 -0.826781 0.028807
8 0.774695 -2.06212 1.31025
6 0.528032 2.57563 -0.881356
7 0.567319 1.41237 -1.55752
8 1.54034 1.04971 -2.22371
8 -0.415691 0.589406 -1.3537
1 -3.41407 -2.40849 -1.12807
6 0.677772 -3.34385 1.397
8 -0.00068 -4.07406 0.662027
6 1.47913 -3.94899 2.53619
1 1.40288 -5.0359 2.52102
1 1.10085 -3.56134 3.48626
1 2.52439 -3.63954 2.45425
6 -2.57436 -2.02537 1.21912
1 -1.9514 -2.91399 1.07969
1 -3.63382 -2.32641 1.24836
1 -2.31016 -1.56721 2.17681
6 0.861293 -2.3693 -2.27585
1 1.71058 -2.68671 -1.66449
1 1.07918 -1.38612 -2.70437
1 -1.8237 -2.80414 -2.79709
6 -3.42225 -0.241488 -1.50901
6 -3.3078 0.03581 -0.00068
1 -2.7959 0.435276 -2.0944
1 -4.42758 -0.297111 -1.92784
1 -4.23928 -0.250299 0.513716
6 1.30861 1.64573 0.939003
8 0.53135 0.690371 1.19132
1 1.26242 3.30612 -1.19002

1 -0.442618 2.85294 -0.492709
6 -2.8868 1.40685 0.460924
6 -2.15428 1.57713 1.6346
6 -3.27378 2.53547 -0.264668
6 -1.80549 2.85258 2.0717
1 -1.82287 0.710256 2.19682
6 -2.93661 3.81353 0.17478
1 -3.84076 2.41442 -1.18448
6 -2.19858 3.97545 1.34576
1 -1.22615 2.96535 2.98371
1 -3.24391 4.68124 -0.400975
1 -1.93088 4.97005 1.68923
6 2.70466 1.42261 0.483694
6 3.54748 2.51152 0.242535
6 3.18562 0.121467 0.321192
6 4.85224 2.30658 -0.180369
1 3.16829 3.52189 0.382517
6 4.49637 -0.081115 -0.105087
1 2.55314 -0.723832 0.584151
6 5.32574 1.0056 -0.361613
1 5.5028 3.15428 -0.370879
1 4.87306 -1.09189 -0.227699
1 6.34636 0.843389 -0.694537
1 1.11482 2.6037 1.44613
1 0.707695 -3.10179 -3.07828

Product arising from ***TS-1pC-I*** *– lowest energy conformation*

SCF energy= -1591.71980564
Zero-point correction= 0.500228
Thermal correction to Energy= 0.530850
Thermal correction to Enthalpy= 0.531794
Thermal correction to Gibbs Free Energy= 0.437280

Coordinates:
6 -2.91354 -2.25758 -0.312533
1 -3.3721 -1.47971 -0.935309
6 -2.8877 -1.79695 1.14861
7 -1.55271 -2.44463 -0.820035
1 -1.05094 -3.17414 -0.301834
7 -1.74001 -0.882714 1.42113
29 -0.366132 -0.856578 -0.220454
8 1.24821 -1.72188 -1.06548
6 1.42318 1.19824 -1.66416
7 0.075968 1.35809 -2.26618
8 -0.712529 0.412931 -2.17645
8 -0.203683 2.40906 -2.79765
1 -2.88864 -2.65409 1.83197
6 1.58183 -2.73333 -0.347673
8 0.864534 -3.21093 0.550804
6 2.95068 -3.31623 -0.6353
1 3.0851 -4.26499 -0.1162
1 3.70282 -2.59725 -0.292936

1 3.08667 -3.44352 -1.71187
6 -0.836416 -1.28371 2.51043
1 -0.27288 -2.17218 2.2118
1 -1.40032 -1.48538 3.43328
1 -0.137255 -0.457507 2.66844
6 -1.49944 -2.731 -2.25861
1 -0.452649 -2.83408 -2.54874
1 -1.92612 -1.88367 -2.80207
1 -3.52407 -3.165 -0.422747
6 -3.88773 -0.678201 1.50954
6 -2.67262 0.231809 1.81643
1 -4.51182 -0.343954 0.67751
1 -4.52306 -0.879425 2.37257
1 -2.56992 0.420922 2.89106
6 1.35474 1.44606 -0.119429
8 0.412241 0.693624 0.512048
1 1.72985 0.169283 -1.85375
1 2.0605 1.92846 -2.16012
6 -2.48518 1.51961 1.06857
6 -1.96352 2.63289 1.72487
6 -2.7645 1.60737 -0.295181
6 -1.71963 3.81455 1.03118
1 -1.72895 2.56739 2.78438
6 -2.52262 2.78436 -0.994619
1 -3.15211 0.744097 -0.832305
6 -1.99611 3.89078 -0.331248
1 -1.30555 4.67191 1.553
1 -2.71611 2.82793 -2.06074
1 -1.79583 4.80612 -0.879315
1 -2.054 -3.64234 -2.51404
6 2.77865 1.19358 0.375358
6 3.06295 0.040936 1.1062
6 3.81189 2.08574 0.084458
6 4.36466 -0.220295 1.52855
1 2.24847 -0.63437 1.35158
6 5.11349 1.82688 0.504242
1 3.59363 2.99906 -0.467098
6 5.39396 0.668045 1.22489
1 4.57538 -1.1194 2.10123
1 5.90754 2.53142 0.27493
1 6.40779 0.463772 1.5557
1 1.12749 2.52234 0.006667

*Cu[**1q**C-(OAc)$_2$] – lowest energy conformation*

SCF energy= -1461.33379042
Zero-point correction= 0.478461
Thermal correction to Energy= 0.507431
Thermal correction to Enthalpy= 0.508375
Thermal correction to Gibbs Free Energy= 0.416922
SCF energy+Solvation free energy= -1461.34705552

Coordinates:

6 -0.239081 -2.09796 1.94142
1 0.13604 -1.44437 2.73587
6 0.945059 -2.51757 1.04881
7 -1.23965 -1.32279 1.20463
1 -1.71854 -1.86289 0.4655
7 1.10269 -1.78815 -0.243337
29 -0.278392 0.092096 0.024135
8 -1.29415 -0.064003 -1.59937
8 0.574218 1.95544 -0.273144
1 0.920981 -3.59984 0.856941
6 -2.25823 -0.879612 -1.84579
8 -2.53699 -1.89616 -1.19053
6 -3.08747 -0.512627 -3.06208
1 -2.43557 -0.394682 -3.93163
1 -3.56721 0.452974 -2.87725
1 -3.84228 -1.27532 -3.2539
6 0.662815 -2.51803 -1.43137
1 1.16882 -3.49431 -1.49993
1 -0.418722 -2.66979 -1.39142
6 -2.25326 -0.705024 2.0841
1 -2.9118 -1.47318 2.51213
1 -1.71443 -0.222365 2.90726
1 -0.690824 -2.9798 2.41584
6 2.35593 -2.05544 1.46234
6 2.5732 -1.74041 -0.028131
1 2.3208 -1.14007 2.05723
1 3.01237 -2.79626 1.91997
1 3.03848 -2.6043 -0.530982
6 3.30542 -0.485582 -0.419622
6 4.5294 -0.201833 0.194484
6 2.82535 0.374551 -1.4054
6 5.26669 0.917562 -0.177135
1 4.90563 -0.86768 0.968167
6 3.55548 1.50636 -1.7679
1 1.86332 0.186401 -1.87581
6 4.77884 1.77618 -1.16252
1 6.21699 1.12416 0.305852
1 3.1551 2.18013 -2.51856
1 5.347 2.6559 -1.44838
6 1.14284 1.80784 0.84098
8 0.839314 0.793948 1.55859
6 2.20678 2.75649 1.30939
1 2.08193 2.96888 2.37312
1 2.18718 3.67378 0.721291
1 3.17403 2.26041 1.16965
1 0.894435 -1.93471 -2.32651
6 -3.04242 0.323067 1.3123
6 -2.55321 1.62854 1.21064
6 -4.1941 -0.031698 0.609671
6 -3.19466 2.56315 0.403042
1 -1.65381 1.90398 1.75935
6 -4.84342 0.904914 -0.189582
1 -4.56459 -1.0514 0.665331
6 -4.34169 2.20085 -0.29798

1 -2.79416 3.56809 0.317938
1 -5.73561 0.619162 -0.738533
1 -4.84421 2.92677 -0.92991

*Cu[**1q**C-(OAc)₂] – higher energy conformation*

SCF energy= -1461.33324018
Zero-point correction= 0.477998
Thermal correction to Energy= 0.506973
Thermal correction to Enthalpy= 0.507917
Thermal correction to Gibbs Free Energy= 0.417339
SCF energy+Solvation free energy= -1461.34645880

Coordinates:
6 1.01459 -0.447442 -2.44254
1 0.849724 0.634342 -2.41345
6 -0.342746 -1.17512 -2.46094
7 1.76933 -0.797727 -1.23829
1 1.94698 -1.80363 -1.13745
7 -0.865875 -1.6095 -1.13081
29 0.461419 -0.417391 0.363761
8 1.02199 -1.80406 1.57081
8 -0.367278 0.871847 1.74438
1 -0.307229 -2.03927 -3.14015
6 1.86183 -2.75697 1.35216
8 2.40258 -3.01751 0.268967
6 2.16404 -3.60347 2.57626
1 1.23244 -4.0229 2.9657
1 2.58429 -2.96842 3.3609
1 2.8612 -4.40273 2.32627
6 -0.745709 -3.03492 -0.833762
1 -1.19786 -3.6451 -1.63176
1 0.30745 -3.30668 -0.720394
6 3.06693 -0.103452 -1.09794
1 3.66354 -0.721354 -0.419988
1 3.58304 -0.059191 -2.0675
1 1.58613 -0.686298 -3.3506
6 -1.6176 -0.335336 -2.67646
6 -2.22534 -1.14468 -1.51423
1 -1.46625 0.709716 -2.39922
1 -2.10704 -0.418826 -3.6473
1 -2.80268 -1.99786 -1.90645
6 -3.03489 -0.432051 -0.463887
6 -4.04425 0.447815 -0.864726
6 -2.82286 -0.64588 0.896864
6 -4.83357 1.09752 0.078344
1 -4.20843 0.625647 -1.92531
6 -3.60223 0.016291 1.84425
1 -2.02505 -1.30546 1.23051
6 -4.61285 0.882691 1.43889
1 -5.61479 1.77782 -0.247347
1 -3.40429 -0.140452 2.89968
1 -5.22019 1.3963 2.17775
6 -0.6292 1.64741 0.783285

8 -0.270534 1.31128 -0.392618
6 -1.39987 2.91719 0.993875
1 -1.04342 3.6877 0.308172
1 -1.31624 3.24528 2.03033
1 -2.45213 2.70693 0.770416
1 -1.25472 -3.25279 0.109601
6 2.87495 1.27946 -0.526857
6 2.73007 2.39213 -1.35567
6 2.72959 1.43657 0.854072
6 2.41329 3.63616 -0.818559
1 2.85553 2.27865 -2.43039
6 2.4073 2.67766 1.39468
1 2.83792 0.572435 1.50937
6 2.24145 3.7782 0.556578
1 2.2948 4.49377 -1.47381
1 2.27319 2.779 2.46701
1 1.98716 4.74723 0.975572

*Cu[**1q**C-(OAc)$_2$] – higher energy conformation*

SCF energy= -1461.33100338
Zero-point correction= 0.478233
Thermal correction to Energy= 0.507418
Thermal correction to Enthalpy= 0.508362
Thermal correction to Gibbs Free Energy= 0.416040
SCF energy+Solvation free energy= -1461.34374548

Coordinates:
6 -1.24761 1.36465 0.706732
1 -0.924967 2.08921 -0.048594
6 -0.132073 1.18573 1.75189
7 -1.50518 0.107016 -0.000587
1 -1.83586 -0.650965 0.61219
7 0.771393 0.012606 1.55606
29 0.315249 -0.641194 -0.633376
8 -0.004832 -2.51991 -0.38292
8 1.85275 -0.759433 -1.98938
1 -0.55347 1.15662 2.7671
6 -1.0499 -3.0923 0.104586
8 -2.00525 -2.52002 0.650062
6 -1.03927 -4.60523 -0.015931
1 -0.171288 -5.00313 0.517223
1 -0.928666 -4.88469 -1.06687
1 -1.95611 -5.02805 0.393771
6 0.519765 -1.13099 2.43032
1 0.533059 -0.829161 3.4895
1 -0.452306 -1.57315 2.19372
6 -2.47065 0.227754 -1.10869
1 -2.04874 0.920596 -1.84589
1 -2.53823 -0.76475 -1.56797
1 -2.16036 1.74723 1.17958
6 1.09068 2.1203 1.67598
6 1.9406 0.858169 1.91414
1 1.2363 2.5111 0.666322

1 1.15844 2.91577 2.41868
1 2.15566 0.742497 2.98913
6 3.20881 0.668251 1.12619
6 4.12737 1.7206 1.06326
6 3.51249 -0.535025 0.492357
6 5.33277 1.57009 0.385845
1 3.89287 2.66411 1.55139
6 4.71275 -0.682401 -0.202091
1 2.80326 -1.35912 0.510975
6 5.62808 0.364398 -0.250553
1 6.03799 2.39486 0.347128
1 4.92128 -1.61764 -0.711599
1 6.56417 0.2469 -0.787592
6 1.8694 0.501707 -2.00501
8 0.967303 1.1335 -1.35873
6 2.95797 1.26181 -2.70293
1 2.54053 2.13029 -3.21614
1 3.49164 0.614501 -3.39843
1 3.65493 1.61831 -1.93588
1 1.28775 -1.89235 2.26872
6 -3.83745 0.686638 -0.657306
6 -4.61076 -0.151766 0.152558
6 -4.32609 1.94538 -1.00201
6 -5.85667 0.269529 0.605771
1 -4.22628 -1.13527 0.420012
6 -5.57595 2.36658 -0.551555
1 -3.72402 2.5987 -1.62935
6 -6.34169 1.52879 0.253518
1 -6.45444 -0.386658 1.23092
1 -5.94903 3.34769 -0.829011
1 -7.31532 1.85474 0.606052

*Cu[**1q**C-(OAc)(O_2NCH_2)] – lowest energy conformation*

SCF energy= -1477.23514498
Zero-point correction= 0.466178
Thermal correction to Energy= 0.494438
Thermal correction to Enthalpy= 0.495382
Thermal correction to Gibbs Free Energy= 0.406121

Coordinates:
6 -0.202837 -2.1486 1.89052
1 0.190181 -1.51377 2.69245
6 0.96487 -2.56614 0.974435
7 -1.20316 -1.34802 1.18161
1 -1.69637 -1.86821 0.437892
7 1.10829 -1.81102 -0.302752
29 -0.241708 0.081504 0.015594
8 -1.27097 -0.034152 -1.60315
8 0.577085 1.92974 -0.24251
1 0.926052 -3.64439 0.764445
6 -2.2458 -0.83588 -1.8523
8 -2.5265 -1.8616 -1.21236
6 -3.08882 -0.437194 -3.049

1 -3.57558 0.516983 -2.82633
1 -3.83973 -1.19918 -3.25815
1 -2.44712 -0.2835 -3.92039
6 0.662151 -2.51691 -1.50163
1 -0.418651 -2.67297 -1.45461
1 1.17139 -3.48934 -1.59776
1 0.881337 -1.91273 -2.38601
6 -2.19857 -0.736009 2.08512
1 -0.658388 -3.03264 2.35734
6 2.38378 -2.12113 1.38043
6 2.57988 -1.74283 -0.098547
1 2.361 -1.23247 2.01537
1 3.04656 -2.88099 1.7957
1 3.06134 -2.57095 -0.643743
6 3.26909 -0.447893 -0.435935
6 4.44097 -0.106259 0.24465
6 2.79069 0.400465 -1.43297
6 5.13026 1.05931 -0.071218
1 4.81446 -0.76235 1.02802
6 3.47144 1.57747 -1.74002
1 1.86678 0.165337 -1.95547
6 4.64486 1.9055 -1.06744
1 6.04106 1.31131 0.46356
1 3.07291 2.24023 -2.50168
1 5.17682 2.81978 -1.31268
8 0.875781 0.732008 1.55572
6 2.13962 2.62186 1.27978
1 2.62616 2.40629 2.21722
1 2.37598 3.45562 0.638677
7 1.22956 1.80552 0.87821
1 -2.85553 -1.50544 2.51359
1 -1.64346 -0.267932 2.90601
6 -2.99389 0.307393 1.34037
6 -4.16523 -0.029693 0.661971
6 -2.49509 1.6093 1.24062
6 -4.82652 0.921218 -0.110072
1 -4.54326 -1.04679 0.715858
6 -3.14885 2.55818 0.460034
1 -1.57531 1.86956 1.76261
6 -4.3171 2.21452 -0.21483
1 -5.73417 0.648932 -0.640128
1 -2.74056 3.56002 0.375369
1 -4.82977 2.95248 -0.824301

*Cu[**1q**C-(OAc)(O_2NCH_2)] – higher energy conformation*

SCF energy= -1477.23224607
Zero-point correction= 0.466099
Thermal correction to Energy= 0.494502
Thermal correction to Enthalpy= 0.495446
Thermal correction to Gibbs Free Energy= 0.405128

Coordinates:
6 1.23377 1.37233 -0.696812

1 0.889166 2.0886 0.057918
6 0.143773 1.19223 -1.76879
7 1.48419 0.110713 0.005737
1 1.81614 -0.645301 -0.608753
7 -0.76365 0.022852 -1.58274
29 -0.342656 -0.634305 0.621979
8 -0.025579 -2.51473 0.371527
8 -1.87676 -0.766472 1.93085
1 0.590477 1.15789 -2.77278
6 1.01981 -3.08739 -0.11363
8 1.98111 -2.51525 -0.649273
6 1.00299 -4.60134 -0.004606
1 0.884259 -4.88858 1.04336
1 1.92099 -5.02453 -0.411366
1 0.137223 -4.9919 -0.546776
6 -0.512298 -1.11623 -2.46128
1 0.458361 -1.56108 -2.22336
1 -0.522267 -0.811531 -3.51993
1 -1.2812 -1.87763 -2.30471
6 2.44153 0.221495 1.1218
1 2.49912 -0.773353 1.57736
1 2.01739 0.913051 1.85891
1 2.15375 1.76764 -1.14462
6 -1.07608 2.13272 -1.71952
6 -1.93463 0.871412 -1.93018
1 -1.22299 2.5516 -0.721527
1 -1.13686 2.90997 -2.4819
1 -2.17439 0.743434 -2.99828
6 -3.18132 0.6932 -1.10507
6 -4.05187 1.77412 -0.942066
6 -3.50512 -0.532281 -0.525508
6 -5.23088 1.63164 -0.218572
1 -3.80046 2.73471 -1.38671
6 -4.67864 -0.673144 0.212918
1 -2.82672 -1.3772 -0.616764
6 -5.54664 0.404314 0.362625
1 -5.90005 2.4789 -0.101875
1 -4.90311 -1.62748 0.678896
1 -6.46321 0.291968 0.934006
8 -0.994284 1.13185 1.32288
6 -2.92341 1.17165 2.55566
1 -2.90587 2.24839 2.50651
1 -3.69 0.587799 3.03865
7 -1.97061 0.534315 1.97342
6 3.81441 0.674224 0.683037
6 4.30826 1.92829 1.03737
6 4.58857 -0.164881 -0.125313
6 5.56379 2.34418 0.597862
1 3.70571 2.58211 1.66373
6 5.84019 0.251113 -0.567585
1 4.20002 -1.14465 -0.400428
6 6.33023 1.50572 -0.205867
1 5.94076 3.3216 0.882916
1 6.43843 -0.405656 -1.19167

1 7.30828 1.82744 -0.549943

*Cu[**1q**C-(OAc)(O_2NCH_2)] – higher energy conformation*

SCF energy= -1477.23386363
Zero-point correction= 0.465329
Thermal correction to Energy= 0.493755
Thermal correction to Enthalpy= 0.494699
Thermal correction to Gibbs Free Energy= 0.404916

Coordinates:
6 1.07747 -0.356924 -2.4029
1 0.912733 0.724225 -2.33966
6 -0.277857 -1.08343 -2.50311
7 1.78583 -0.75561 -1.18572
1 1.95063 -1.76716 -1.1144
7 -0.843067 -1.57328 -1.2116
29 0.423282 -0.444577 0.382494
8 0.951357 -1.88053 1.54749
8 -0.427644 0.741737 1.7917
1 -0.215256 -1.91597 -3.21849
6 1.7859 -2.83267 1.30849
8 2.35844 -3.0464 0.23124
6 2.02995 -3.75045 2.49333
1 2.3499 -3.15976 3.35538
1 2.78048 -4.50124 2.2475
1 1.0902 -4.23874 2.76747
6 -0.74773 -3.01198 -0.981391
1 0.299509 -3.30097 -0.855743
1 -1.18612 -3.5787 -1.81841
1 -1.28246 -3.26968 -0.062658
6 3.08566 -0.08289 -0.979425
1 3.63424 -0.707855 -0.267752
1 1.68368 -0.55753 -3.29779
6 -1.54386 -0.232138 -2.72668
6 -2.18639 -1.06547 -1.60076
1 -1.39767 0.805856 -2.42159
1 -2.00915 -0.289995 -3.71121
1 -2.77945 -1.89195 -2.02468
6 -2.98639 -0.360782 -0.537035
6 -3.91031 0.617732 -0.912707
6 -2.84688 -0.679217 0.812426
6 -4.6853 1.26492 0.043652
1 -4.01835 0.875526 -1.96408
6 -3.61027 -0.019787 1.77398
1 -2.11527 -1.41922 1.12859
6 -4.53468 0.94781 1.39277
1 -5.39949 2.02347 -0.2629
1 -3.46719 -0.259007 2.82301
1 -5.13011 1.45901 2.14318
8 -0.318984 1.29089 -0.315028
6 -1.44941 2.67303 1.11345
1 -1.67395 3.31638 0.278262
1 -1.78207 2.83415 2.126

7 -0.757427 1.61381 0.878982
1 3.65434 -0.055136 -1.91981
6 2.88853 1.30807 -0.43116
6 2.87293 2.42167 -1.26937
6 2.60492 1.47513 0.92749
6 2.55119 3.68005 -0.766226
1 3.1043 2.29937 -2.32543
6 2.27008 2.72719 1.4314
1 2.62085 0.61169 1.59253
6 2.23888 3.83172 0.581799
1 2.53711 4.54036 -1.42833
1 2.02613 2.83587 2.48355
1 1.9761 4.81018 0.972244

*TS-**1q**C-**I** – lowest energy conformation*

SCF energy= -1822.64883257
Zero-point correction= 0.579388
Thermal correction to Energy= 0.614680
Thermal correction to Enthalpy= 0.615624
Thermal correction to Gibbs Free Energy= 0.507147
SCF energy+Solvation free energy= -1822.66286994

Coordinates:
6 1.65548 1.88281 0.831946
1 1.27719 1.55253 1.80337
6 0.699406 2.93532 0.239961
7 1.72242 0.693109 -0.02065
1 2.17289 0.889579 -0.927998
7 -0.311711 2.43221 -0.740663
29 -0.155622 0.124754 -0.605116
8 0.432932 -0.52814 -2.34817
6 -2.57903 -0.915292 1.79168
7 -1.24444 -0.740866 1.82098
8 -0.453662 -1.62128 2.17058
8 -0.793797 0.35934 1.30173
1 1.26449 3.7634 -0.210052
6 1.51279 -0.307944 -3.0124
8 2.42448 0.46693 -2.68559
6 1.60922 -1.08308 -4.31455
1 2.57963 -0.923562 -4.7839
1 0.812518 -0.751937 -4.98686
1 1.44816 -2.14688 -4.12215
6 -0.03369 2.76975 -2.13608
1 0.888373 2.27259 -2.45369
1 0.077645 3.85765 -2.26985
1 -0.849756 2.41623 -2.77307
6 2.4202 -0.448065 0.606403
1 2.37052 -1.27369 -0.11463
1 1.84369 -0.744186 1.48993
1 2.65347 2.31211 0.980721
6 -0.438142 3.4448 1.14457
6 -1.36762 3.24432 -0.064332
1 -0.659224 2.73528 1.94511

1 -0.345653 4.45742 1.53828
1 -1.49497 4.19175 -0.612507
6 -2.53029 -1.59402 -0.287685
8 -1.94119 -0.684576 -0.926319
1 -2.9423 -1.74972 2.3745
1 -3.15678 -0.01374 1.63589
6 -2.71497 2.60528 0.151297
6 -3.30492 1.81628 -0.834758
6 -3.41247 2.83802 1.33845
6 -4.56531 1.2586 -0.633845
1 -2.7681 1.60549 -1.75388
6 -4.67832 2.29262 1.53853
1 -2.96051 3.44914 2.11586
6 -5.25759 1.49708 0.5515
1 -5.0044 0.636045 -1.40819
1 -5.20779 2.48374 2.46695
1 -6.24246 1.06654 0.705186
6 3.85881 -0.144446 0.953124
6 4.28821 -0.118826 2.27904
6 4.77083 0.144091 -0.06762
6 5.61363 0.182762 2.58775
1 3.57908 -0.339943 3.07325
6 6.09245 0.449251 0.2399
1 4.43485 0.129138 -1.10375
6 6.51693 0.46756 1.5685
1 5.93819 0.196077 3.62381
1 6.79545 0.66863 -0.558
1 7.54993 0.702603 1.80615
1 -3.63088 -1.56669 -0.268085
6 -1.90901 -2.92448 -0.063413
6 -2.61169 -3.9279 0.610783
6 -0.625001 -3.18183 -0.547889
6 -2.02937 -5.16964 0.819318
1 -3.61674 -3.7256 0.976167
6 -0.041881 -4.42894 -0.335191
1 -0.111118 -2.4266 -1.13809
6 -0.737704 -5.41844 0.35126
1 -2.57643 -5.94596 1.34474
1 0.955334 -4.62887 -0.71509
1 -0.281053 -6.38965 0.515243

TS-1q*C*-I *– higher energy conformation*

SCF energy= -1822.63332074
Zero-point correction= 0.580155
Thermal correction to Energy= 0.614923
Thermal correction to Enthalpy= 0.615867
Thermal correction to Gibbs Free Energy= 0.511851
SCF energy+Solvation free energy= -1822.65053262

Coordinates:
6 1.77085 -1.87624 1.61478
1 2.06113 -0.889952 1.99762
6 2.7199 -2.29637 0.506771

7 0.42221 -1.74635 1.06818
1 0.121109 -2.63633 0.666528
7 2.6818 -1.29571 -0.615825
29 0.735249 -0.41291 -0.645658
8 0.495604 -0.428236 -2.61295
6 -0.488535 2.87066 0.32684
7 0.63199 2.1995 0.004812
8 0.993951 1.23052 0.761668
8 1.16466 2.34903 -1.11333
1 2.48549 -3.30873 0.156491
6 -0.122749 -1.53257 -2.82165
8 -0.246857 -2.41961 -1.95544
6 -0.698649 -1.71866 -4.21133
1 -1.46195 -0.955049 -4.38565
1 -1.13662 -2.71095 -4.31689
1 0.08648 -1.56879 -4.95691
6 2.89725 -1.9593 -1.91666
1 2.03622 -2.59366 -2.13064
1 3.81079 -2.56998 -1.89683
1 2.97931 -1.19366 -2.68976
1 1.82384 -2.59684 2.44589
6 4.19286 -1.97621 0.788641
6 4.01879 -0.732837 -0.11272
1 4.45734 -1.81217 1.83394
1 4.88535 -2.70427 0.363514
1 4.71642 -0.703596 -0.955399
6 -1.56242 1.33798 -0.858926
8 -1.14814 0.231922 -0.427614
1 -0.856542 2.72819 1.33196
1 -0.67024 3.7775 -0.231491
6 3.98051 0.637049 0.513946
6 3.85545 1.73447 -0.337731
6 4.08195 0.860567 1.88783
6 3.80287 3.0267 0.169558
1 3.75394 1.57318 -1.4078
6 4.03315 2.15363 2.40028
1 4.19994 0.032079 2.57906
6 3.8875 3.23957 1.54246
1 3.67168 3.86182 -0.510541
1 4.10923 2.31055 3.47187
1 3.8436 4.24796 1.94246
6 -2.95432 1.77049 -0.572028
6 -3.48705 2.87474 -1.24156
6 -3.73998 1.06141 0.339154
6 -4.79797 3.27054 -1.00157
1 -2.86964 3.41871 -1.95354
6 -5.05035 1.45962 0.577411
1 -3.31789 0.199231 0.844657
6 -5.58069 2.56273 -0.089922
1 -5.21186 4.12658 -1.52542
1 -5.65832 0.899697 1.28188
1 -6.60565 2.86944 0.095953
1 -1.06353 1.78621 -1.73353
6 -0.55277 -1.28465 2.06801

1 -0.253844 -1.63597 3.06763
1 -0.508004 -0.188709 2.07266
6 -1.96768 -1.74433 1.8029
6 -2.9025 -1.60205 2.83287
6 -2.38795 -2.25065 0.571134
6 -4.23798 -1.9382 2.63716
1 -2.57963 -1.21045 3.7953
6 -3.72443 -2.60028 0.381451
1 -1.69904 -2.33014 -0.266831
6 -4.65356 -2.4406 1.40488
1 -4.95172 -1.81344 3.44598
1 -4.03985 -2.98327 -0.584144
1 -5.69442 -2.7051 1.24534

TS-1q*C*-I *– higher energy conformation*

SCF energy= -1822.65206163
Zero-point correction= 0.581055
Thermal correction to Energy= 0.615389
Thermal correction to Enthalpy= 0.616334
Thermal correction to Gibbs Free Energy= 0.515059
SCF energy+Solvation free energy= -1822.66579683

Coordinates:
6 -0.325971 2.85498 1.6437
1 -0.6483 2.22482 2.47764
6 -1.53108 3.1271 0.728869
7 0.75254 2.1225 0.970746
1 1.21305 2.69027 0.241347
7 -1.68105 2.22926 -0.453095
29 -0.046413 0.594042 -0.148507
8 1.25457 0.719158 -1.58502
6 -1.80714 -2.10922 1.35312
7 -0.855255 -1.24659 1.76086
8 0.23994 -1.61194 2.1999
8 -1.06106 0.007279 1.5122
1 -1.54756 4.17662 0.403251
6 2.0271 1.70328 -1.88887
8 1.92631 2.86181 -1.45756
6 3.1335 1.34348 -2.86171
1 3.73775 2.22025 -3.09468
1 2.70037 0.927516 -3.77535
1 3.75822 0.568601 -2.40728
6 -1.33996 2.83971 -1.73838
1 -0.28024 3.11092 -1.74059
1 -1.94938 3.73852 -1.92666
1 -1.50852 2.11993 -2.54448
6 1.7426 1.63515 1.95624
1 1.2442 0.859989 2.54885
1 2.01838 2.46205 2.6279
1 0.055942 3.79621 2.0619
6 -2.90483 2.66401 1.24776
6 -3.14184 2.10031 -0.160847
1 -2.79714 1.86677 1.98751

1 -3.5947 3.42991 1.60346
1 -3.68732 2.83446 -0.776357
6 -1.05121 -2.15471 -0.661076
8 -0.916006 -0.953895 -1.01711
1 -1.70005 -3.11525 1.73272
1 -2.77267 -1.66347 1.15344
6 -3.78919 0.74908 -0.317368
6 -3.56049 -0.020801 -1.45862
6 -4.67992 0.27719 0.647959
6 -4.19906 -1.24576 -1.62517
1 -2.85743 0.325236 -2.20867
6 -5.32815 -0.946358 0.48131
1 -4.87169 0.869336 1.53912
6 -5.08445 -1.71383 -0.654556
1 -4.00456 -1.8346 -2.51708
1 -6.01903 -1.29949 1.24085
1 -5.5852 -2.66805 -0.787195
6 2.98218 1.07895 1.30175
6 3.91212 1.94002 0.710442
6 3.2289 -0.294154 1.30736
6 5.07386 1.4375 0.135629
1 3.71901 3.00967 0.69146
6 4.40621 -0.794987 0.751755
1 2.50076 -0.967764 1.75369
6 5.32812 0.066298 0.164883
1 5.78566 2.11586 -0.324763
1 4.60586 -1.86103 0.794745
1 6.24565 -0.325925 -0.263587
6 0.124131 -3.04345 -0.46794
6 -0.060716 -4.39056 -0.141108
6 1.41023 -2.5509 -0.697669
6 1.0299 -5.23931 -0.023652
1 -1.06841 -4.76993 0.017917
6 2.50141 -3.4117 -0.5942
1 1.54873 -1.51706 -1.01016
6 2.31655 -4.7472 -0.251181
1 0.882124 -6.2835 0.233298
1 3.49738 -3.03524 -0.802924
1 3.17123 -5.41257 -0.174417
1 -1.98659 -2.66061 -0.947516

TS-1q*C*-I *– higher energy conformation*

SCF energy= -1822.63095462
Zero-point correction= 0.579383
Thermal correction to Energy= 0.614252
Thermal correction to Enthalpy= 0.615196
Thermal correction to Gibbs Free Energy= 0.510706
SCF energy+Solvation free energy= -1822.64604153

Coordinates:
6 0.473519 1.75071 1.74998
1 -0.201316 0.995011 2.16594
6 -0.284527 2.64113 0.783802

7 1.59641 1.0623 1.10274
1 2.07869 1.72826 0.475711
7 -1.09136 1.92864 -0.232781
29 1.06644 -0.382638 -0.243985
8 2.22944 0.214337 -1.64037
6 -1.01276 -2.13967 1.03113
8 -0.170575 -1.18264 1.1115
6 -0.160109 -3.75294 -0.005245
1 0.144056 -4.52246 0.692686
1 -0.886624 -3.96826 -0.774973
7 0.862084 -2.94853 -0.404103
8 1.8821 -2.83166 0.279359
8 0.617331 -2.08335 -1.31296
1 0.389966 3.3837 0.331913
6 2.79187 1.36589 -1.79254
8 2.53784 2.38409 -1.13442
6 3.84852 1.39525 -2.87783
1 4.19704 2.41503 -3.04162
1 3.44802 0.970978 -3.8014
1 4.68307 0.763557 -2.55773
6 -0.699201 2.03755 -1.62937
1 0.175514 1.42186 -1.85554
1 -0.464017 3.07595 -1.91733
1 -1.53632 1.68717 -2.24506
6 2.55297 0.47723 2.06944
1 2.08739 -0.410851 2.51157
1 2.72831 1.20478 2.87563
1 0.849798 2.37526 2.57576
6 -1.5682 3.25552 1.37173
6 -2.28864 2.738 0.107313
1 -1.89977 2.75437 2.28569
1 -1.57873 4.33483 1.52453
1 -2.42758 3.55346 -0.620486
1 -1.13424 -2.74613 1.94031
6 -3.57563 1.97496 0.259609
6 -3.64669 0.896179 1.14666
6 -4.70812 2.32017 -0.475032
6 -4.8298 0.187742 1.30557
1 -2.74929 0.592805 1.68297
6 -5.8976 1.60757 -0.324343
1 -4.65924 3.15506 -1.17045
6 -5.9605 0.543836 0.569144
1 -4.86688 -0.658636 1.98458
1 -6.77236 1.88609 -0.904201
1 -6.88422 -0.015098 0.686516
6 -2.23763 -2.04496 0.187028
6 -3.29944 -2.91964 0.438114
6 -2.34078 -1.1132 -0.845272
6 -4.45275 -2.86437 -0.333908
1 -3.21392 -3.64725 1.24341
6 -3.49307 -1.06391 -1.62411
1 -1.54035 -0.400159 -1.00386
6 -4.54827 -1.93557 -1.37104
1 -5.27736 -3.53993 -0.128053

1 -3.57845 -0.324297 -2.41445
1 -5.4516 -1.88376 -1.97104
6 3.85455 0.1311 1.38754
6 4.65392 1.15421 0.867389
6 4.26362 -1.1931 1.24404
6 5.83575 0.85373 0.200008
1 4.34177 2.19102 0.968898
6 5.44982 -1.49561 0.577773
1 3.64312 -1.99097 1.63857
6 6.23482 -0.47481 0.052709
1 6.44528 1.65556 -0.205654
1 5.75351 -2.53152 0.465126
1 7.1569 -0.710192 -0.469984

TS-1q**C*****-II*** *– lowest energy conformation*

SCF energy= -1822.64937938
Zero-point correction= 0.579558
Thermal correction to Energy= 0.614846
Thermal correction to Enthalpy= 0.615790
Thermal correction to Gibbs Free Energy= 0.509297
SCF energy+Solvation free energy= -1822.66467018

Coordinates:
6 -2.27949 -1.67103 0.360738
1 -1.72364 -1.73116 1.30477
6 -1.56304 -2.48562 -0.712107
7 -2.27524 -0.259574 -0.031398
1 -2.71408 -0.097278 -0.946767
7 -0.36623 -1.78235 -1.27819
29 -0.338239 0.242498 -0.399213
8 -0.811777 2.03686 -1.14346
6 1.12496 2.20588 1.75058
7 1.1016 0.896492 2.01601
8 0.047878 0.217133 1.65702
8 2.0722 0.295025 2.49341
1 -2.25077 -2.77834 -1.5169
6 -1.7974 2.18917 -1.95527
8 -2.57634 1.29233 -2.31634
6 -1.97555 3.60401 -2.4789
1 -2.85265 3.66595 -3.12245
1 -1.08271 3.89735 -3.03816
1 -2.07472 4.29576 -1.63795
6 -0.470845 -1.3705 -2.6859
1 -1.20494 -0.563684 -2.79455
1 -0.770068 -2.21624 -3.323
1 0.508598 -1.00651 -3.01064
6 -2.91964 0.642142 0.945927
1 -2.6962 1.66264 0.617244
1 -2.42302 0.488355 1.90831
1 -3.29592 -2.04908 0.531393
6 -0.664186 -3.63534 -0.227895
6 0.464051 -3.02159 -1.08514
1 -0.449506 -3.57715 0.842068

1 -0.965545 -4.65 -0.48812
1 0.533382 -3.54083 -2.05104
6 1.99444 1.73099 -0.435482
8 1.57141 0.573692 -0.673854
1 0.188795 2.65374 1.45021
1 1.94241 2.76096 2.18356
6 1.83745 -2.90243 -0.490486
6 2.91716 -3.51209 -1.13235
6 2.04058 -2.26787 0.737817
6 4.18121 -3.5139 -0.548784
1 2.76441 -3.99873 -2.09304
6 3.30236 -2.27403 1.32492
1 1.23175 -1.73881 1.23805
6 4.37188 -2.90378 0.688186
1 5.01112 -3.99571 -1.05658
1 3.44028 -1.75866 2.26964
1 5.35405 -2.91103 1.15242
6 3.40773 1.94453 -0.094443
6 3.93381 3.23913 -0.038226
6 4.21672 0.839493 0.177112
6 5.27017 3.42666 0.28721
1 3.28985 4.09088 -0.246924
6 5.55351 1.03237 0.505895
1 3.78187 -0.151869 0.119128
6 6.079 2.32156 0.561411
1 5.68545 4.42853 0.329508
1 6.18403 0.175128 0.721392
1 7.1235 2.47103 0.818067
1 1.38174 2.59477 -0.716759
6 -4.41036 0.419908 1.03328
6 -4.99578 -0.137425 2.16952
6 -5.21762 0.748564 -0.061209
6 -6.37065 -0.361201 2.21904
1 -4.37121 -0.394128 3.02202
6 -6.589 0.522576 -0.013765
1 -4.75903 1.18066 -0.950143
6 -7.16838 -0.031547 1.12745
1 -6.81641 -0.792658 3.11001
1 -7.20853 0.783881 -0.866135
1 -8.23952 -0.204208 1.16459

***TS-1q*C*-II** – higher energy conformation*

SCF energy= -1822.63008073
Zero-point correction= 0.580359
Thermal correction to Energy= 0.614456
Thermal correction to Enthalpy= 0.615400
Thermal correction to Gibbs Free Energy= 0.512534
SCF energy+Solvation free energy= -1822.64668351

Coordinates:
6 2.46488 1.30917 -1.60975
1 2.51342 0.244786 -1.87177
6 3.29416 1.55933 -0.360488

7 1.06071 1.62823 -1.3852
1 0.981274 2.59664 -1.05659
7 2.77882 0.749539 0.799535
29 0.572012 0.564135 0.552388
8 0.196139 2.01899 1.77603
6 -1.15351 -2.4726 -0.193448
7 0.057605 -2.00526 0.261165
8 0.693505 -1.19956 -0.505106
8 0.334412 -2.06564 1.45029
1 3.33025 2.62687 -0.124047
6 0.400627 3.25888 1.5212
8 1.02051 3.70064 0.540565
6 -0.169965 4.21242 2.55689
1 -1.24967 4.05768 2.63348
1 0.045011 5.24595 2.28641
1 0.258196 3.9827 3.5368
6 3.03723 1.45536 2.07146
1 2.53422 2.41833 2.05541
1 4.11353 1.62492 2.21651
1 2.65184 0.856576 2.90026
6 0.256267 1.36782 -2.57637
1 0.460665 0.333863 -2.88042
1 0.571517 2.0146 -3.41253
1 2.90272 1.88107 -2.44489
6 4.614 0.775333 -0.363158
6 3.9268 -0.246002 0.571002
1 4.96378 0.418781 -1.33361
1 5.43433 1.29923 0.130272
1 4.45272 -0.384859 1.52044
6 -2.02214 -0.739298 0.100921
8 -1.31988 -0.018101 0.898542
1 -1.18735 -2.62512 -1.265
1 -1.58053 -3.2255 0.457587
6 3.58046 -1.61907 0.051019
6 3.12051 -2.56417 0.970775
6 3.74241 -2.00839 -1.27939
6 2.78476 -3.85103 0.567197
1 2.99632 -2.27749 2.01178
6 3.41231 -3.29746 -1.68885
1 4.12964 -1.31117 -2.01528
6 2.92362 -4.21995 -0.768646
1 2.40747 -4.56164 1.29591
1 3.54194 -3.58046 -2.72896
1 2.66494 -5.22502 -1.08744
6 -3.36936 -1.18458 0.577732
6 -4.32057 -1.64051 -0.336555
6 -3.67655 -1.14027 1.93743
6 -5.57288 -2.05501 0.105024
1 -4.07682 -1.65235 -1.39739
6 -4.92928 -1.55648 2.37785
1 -2.92543 -0.764792 2.62514
6 -5.87772 -2.01529 1.46485
1 -6.31266 -2.40496 -0.608713
1 -5.16994 -1.51978 3.43612

1 -6.85484 -2.33769 1.81143
1 -1.98408 -0.496744 -0.971286
6 -1.22892 1.51147 -2.34193
6 -1.74715 2.22066 -1.25985
6 -2.11206 0.878811 -3.22367
6 -3.12359 2.25892 -1.03876
1 -1.08291 2.72848 -0.566723
6 -3.48512 0.929221 -3.01293
1 -1.71319 0.325612 -4.07163
6 -3.99445 1.6092 -1.90624
1 -3.50918 2.78466 -0.170952
1 -4.15793 0.42511 -3.70073
1 -5.06364 1.6248 -1.71981

TS-1q*C*-II *– higher energy conformation*

SCF energy= -1822.64970786
Zero-point correction= 0.579576
Thermal correction to Energy= 0.614552
Thermal correction to Enthalpy= 0.615496
Thermal correction to Gibbs Free Energy= 0.510563
SCF energy+Solvation free energy= -1822.66537161

Coordinates:
6 -1.4664 -2.94153 0.9192
1 -0.808982 -2.71892 1.76982
6 -0.630801 -3.484 -0.234464
7 -2.11373 -1.67607 0.555824
1 -2.70904 -1.76122 -0.2835
7 0.148015 -2.41076 -0.922016
29 -0.632295 -0.467094 -0.193595
8 -1.76924 0.674779 -1.3675
6 0.09368 2.40481 1.17406
7 0.536521 1.29283 1.77389
8 -0.211773 0.225442 1.71929
8 1.65734 1.21244 2.28684
1 -1.25002 -4.04312 -0.946915
6 -2.80511 0.212057 -1.9669
8 -3.17574 -0.975322 -1.94727
6 -3.58861 1.23589 -2.76843
1 -2.98479 1.56293 -3.62071
1 -3.78739 2.10949 -2.14312
1 -4.52359 0.805456 -3.12795
6 -0.153629 -2.23952 -2.35105
1 -1.18674 -1.89889 -2.47155
1 -0.019117 -3.18672 -2.89508
1 0.528403 -1.48824 -2.75924
6 -2.8919 -1.13121 1.68986
1 -2.1828 -0.943823 2.50087
1 -3.60982 -1.89435 2.02532
1 -2.19856 -3.69161 1.25119
6 0.673442 -4.19523 0.173295
6 1.43329 -3.15309 -0.685036
1 0.896408 -4.11666 1.24031

1 0.783726 -5.23177 -0.146118
1 1.76308 -3.59716 -1.63282
6 0.976869 1.69809 -0.883205
8 0.991709 0.446443 -0.787161
1 -0.945312 2.41277 0.875553
1 0.667082 3.29817 1.36719
6 2.59184 -2.4262 -0.061766
6 3.86756 -2.59633 -0.604108
6 2.44163 -1.65254 1.09198
6 4.98304 -2.02732 0.005216
1 3.98894 -3.18665 -1.50964
6 3.55514 -1.08349 1.70359
1 1.45842 -1.45241 1.51365
6 4.82801 -1.27824 1.16823
1 5.96799 -2.17067 -0.428589
1 3.40943 -0.462081 2.57992
1 5.69408 -0.834336 1.65051
6 2.23944 2.44896 -0.791683
6 2.28988 3.801 -1.14456
6 3.38637 1.79212 -0.339066
6 3.48855 4.49505 -1.04896
1 1.38719 4.3008 -1.48975
6 4.58305 2.49278 -0.242058
1 3.32048 0.74207 -0.074528
6 4.63508 3.8398 -0.595089
1 3.53427 5.54344 -1.32625
1 5.47545 1.9851 0.111281
1 5.57152 4.38433 -0.518862
1 0.092067 2.19301 -1.30242
6 -3.61837 0.141821 1.33742
6 -4.73686 0.114324 0.500913
6 -3.18413 1.36146 1.8561
6 -5.39987 1.2932 0.174781
1 -5.07908 -0.830067 0.085178
6 -3.85005 2.54225 1.53491
1 -2.30911 1.377 2.49972
6 -4.95728 2.51016 0.691778
1 -6.26375 1.26249 -0.482233
1 -3.50237 3.48577 1.94542
1 -5.4787 3.42884 0.440405

TS-1q*C*-II *– higher energy conformation*

SCF energy= -1822.64664357
Zero-point correction= 0.580510
Thermal correction to Energy= 0.615218
Thermal correction to Enthalpy= 0.616162
Thermal correction to Gibbs Free Energy= 0.512117
SCF energy+Solvation free energy= -1822.66277014

Coordinates:
6 -0.627407 -0.237837 -2.60161
1 0.175038 -0.968062 -2.46094
6 -0.026655 1.18006 -2.67007

7 -1.54579 -0.390159 -1.4695
1 -2.37435 0.219966 -1.54943
7 -0.109722 1.9907 -1.42231
29 -0.639453 0.381411 0.254794
8 -2.13476 1.35316 0.965238
6 2.12323 -0.677234 0.336367
8 0.999895 -0.704677 -0.250881
6 1.8396 -1.23757 2.425
1 2.11427 -2.27507 2.55114
1 2.37548 -0.440015 2.9199
7 0.526054 -1.01642 2.22088
8 -0.215859 -1.92319 1.82587
8 0.118282 0.203609 2.17695
1 -0.468896 1.7428 -3.50433
6 -3.32346 1.48577 0.47925
8 -3.65461 1.28644 -0.696023
6 -4.35184 1.95232 1.49264
1 -4.38832 1.23296 2.31489
1 -5.33321 2.03895 1.02642
1 -4.04277 2.91495 1.91017
6 -1.11915 3.04795 -1.42478
1 -2.11523 2.60765 -1.49709
1 -0.957187 3.73832 -2.26812
1 -1.06278 3.61122 -0.489043
6 -1.99672 -1.79085 -1.28449
1 -1.20109 -2.33008 -0.756372
1 -2.13962 -2.26303 -2.26711
1 -1.13878 -0.47628 -3.54447
6 1.50947 1.32633 -2.65042
6 1.28544 2.45282 -1.62035
1 1.99982 0.453338 -2.21413
1 1.99162 1.60471 -3.58803
1 1.28622 3.43365 -2.12238
6 2.14188 2.50844 -0.384855
6 3.52961 2.39299 -0.500016
6 1.57121 2.63038 0.882635
6 4.33411 2.38354 0.635696
1 3.97877 2.28565 -1.4848
6 2.37445 2.60822 2.02222
1 0.491775 2.70799 0.995255
6 3.75613 2.48201 1.90254
1 5.4112 2.2866 0.533625
1 1.90923 2.67091 3.0008
1 4.38174 2.46357 2.78969
6 3.15298 -1.67923 -0.029805
6 4.48662 -1.45057 0.316643
6 2.79982 -2.83185 -0.734605
6 5.46545 -2.37079 -0.039678
1 4.74529 -0.544816 0.861596
6 3.7804 -3.75317 -1.08488
1 1.75663 -2.98787 -0.992619
6 5.11198 -3.52398 -0.739541
1 6.5029 -2.1909 0.224361
1 3.50921 -4.65182 -1.63009

1 5.87557 -4.24397 -1.0176
1 2.49034 0.268633 0.744754
6 -3.2958 -1.81708 -0.517103
6 -4.50582 -1.82704 -1.21178
6 -3.30833 -1.73657 0.876954
6 -5.71579 -1.74699 -0.531151
1 -4.49824 -1.88336 -2.29818
6 -4.52041 -1.6552 1.55876
1 -2.37121 -1.72829 1.42719
6 -5.72416 -1.65524 0.858254
1 -6.6498 -1.75011 -1.08441
1 -4.52119 -1.59025 2.643
1 -6.66661 -1.58815 1.39353

Product arising from ***TS-1q*****C-*****I***

SCF energy= -1822.67015161
Zero-point correction= 0.582813
Thermal correction to Energy= 0.617775
Thermal correction to Enthalpy= 0.618719
Thermal correction to Gibbs Free Energy= 0.513020

Coordinates:
6 1.07589 1.78376 1.13976
1 0.830042 1.1957 2.02882
6 -0.125259 2.66648 0.754101
7 1.38892 0.842192 0.058787
1 1.78666 1.33095 -0.763405
7 -0.969367 2.16036 -0.370126
29 -0.28309 0.010946 -0.801072
8 0.561179 -0.060236 -2.60599
6 -2.6059 -1.85679 0.800905
7 -1.82498 -0.990225 1.7391
8 -2.20268 -0.909378 2.8819
8 -0.838303 -0.378522 1.32293
1 0.20366 3.69075 0.529359
6 1.58093 0.556423 -3.07148
8 2.23013 1.44595 -2.48815
6 1.99126 0.130943 -4.47159
1 2.87007 0.685506 -4.79999
1 1.15957 0.300906 -5.16049
1 2.19986 -0.942728 -4.47588
6 -0.754121 2.83572 -1.65207
1 0.271528 2.65629 -1.9904
1 -0.921185 3.92026 -1.55879
1 -1.43633 2.43199 -2.40503
6 2.34026 -0.220849 0.440995
1 2.49885 -0.828534 -0.457586
1 1.85569 -0.862603 1.18825
1 1.94857 2.40197 1.38016
6 -1.36305 2.66878 1.67252
6 -2.19638 2.54919 0.384164
1 -1.41535 1.76944 2.29157
1 -1.52506 3.55718 2.28365

1 -2.5165 3.54934 0.049501
6 -1.85327 -2.19928 -0.490793
8 -1.72895 -1.09953 -1.30502
1 -2.91769 -2.70742 1.40894
1 -3.4816 -1.25158 0.551504
6 -3.37853 1.61715 0.335383
6 -3.78939 1.04116 -0.869393
6 -4.12315 1.37478 1.49167
6 -4.94386 0.265165 -0.921819
1 -3.19426 1.17396 -1.76691
6 -5.27555 0.591759 1.44349
1 -3.80147 1.80161 2.4384
6 -5.69508 0.04564 0.232155
1 -5.24607 -0.180529 -1.86421
1 -5.84215 0.410231 2.35143
1 -6.59526 -0.559911 0.190841
6 3.65596 0.312409 0.958288
6 4.05986 0.081217 2.27181
6 4.47045 1.07639 0.114875
6 5.26626 0.596619 2.74319
1 3.42605 -0.510068 2.92868
6 5.67142 1.5956 0.586743
1 4.15188 1.26138 -0.910559
6 6.07288 1.35526 1.90083
1 5.57232 0.407032 3.76759
1 6.29935 2.18704 -0.072657
1 7.0125 1.75879 2.26547
1 -2.51689 -2.94305 -0.974236
6 -0.515235 -2.90794 -0.229078
6 -0.177581 -3.53742 0.972375
6 0.421335 -2.89844 -1.26854
6 1.07003 -4.13751 1.1354
1 -0.87887 -3.56443 1.80376
6 1.6647 -3.50578 -1.10835
1 0.167376 -2.38337 -2.19085
6 1.99614 -4.12295 0.095672
1 1.31743 -4.61441 2.0792
1 2.37928 -3.48842 -1.92671
1 2.96846 -4.58825 0.224735

*Product arising from **TS-1q**C-II*

SCF energy= -1822.67503821
Zero-point correction= 0.582702
Thermal correction to Energy= 0.617719
Thermal correction to Enthalpy= 0.618663
Thermal correction to Gibbs Free Energy= 0.512696

Coordinates:
6 -2.27459 -1.72075 -0.258227
1 -1.78361 -2.16325 0.617422
6 -1.52997 -2.11517 -1.5328
7 -2.2185 -0.266213 -0.102585
1 -2.65392 0.206546 -0.908676

7 -0.279247 -1.31325 -1.71618
29 -0.322 0.449239 -0.460776
8 -1.0123 2.34385 -0.465977
6 1.52721 2.29723 1.15243
7 1.33336 1.07955 1.97244
8 0.27513 0.456699 1.81406
8 2.21423 0.718912 2.71522
1 -2.18638 -2.03012 -2.40683
6 -1.91961 2.59971 -1.33713
8 -2.50915 1.74336 -2.02272
6 -2.27251 4.0681 -1.48973
1 -3.03285 4.20242 -2.25853
1 -1.37256 4.63208 -1.74835
1 -2.6358 4.4549 -0.533331
6 -0.17821 -0.614122 -3.00856
1 -0.948483 0.164006 -3.06006
1 -0.312098 -1.31364 -3.847
1 0.807956 -0.145893 -3.05885
6 -2.85106 0.243159 1.12881
1 -2.6082 1.30988 1.17724
1 -2.36684 -0.243344 1.98238
1 -3.30715 -2.09371 -0.281114
6 -0.711592 -3.41747 -1.45803
6 0.564882 -2.55631 -1.65706
1 -0.758969 -3.92527 -0.491722
1 -0.900725 -4.13757 -2.25477
1 1.03167 -2.73043 -2.63299
6 2.13904 1.87363 -0.234207
8 1.48794 0.810646 -0.790263
1 0.537678 2.72616 1.00212
1 2.19934 2.94706 1.71109
6 1.61593 -2.62197 -0.585333
6 2.87342 -3.15081 -0.874011
6 1.33871 -2.2127 0.720503
6 3.83595 -3.27563 0.126942
1 3.10383 -3.46428 -1.8893
6 2.30306 -2.3093 1.717
1 0.369629 -1.78691 0.965501
6 3.55425 -2.84974 1.42157
1 4.8106 -3.69129 -0.111321
1 2.08205 -1.94148 2.71318
1 4.31076 -2.92439 2.1965
6 3.62499 1.59199 -0.050198
6 4.53359 2.63848 0.115695
6 4.08386 0.277924 -0.015747
6 5.88474 2.37691 0.31962
1 4.18122 3.66847 0.076829
6 5.43576 0.013797 0.190468
1 3.36763 -0.522711 -0.1657
6 6.33911 1.05963 0.359561
1 6.58453 3.1984 0.441779
1 5.78115 -1.01618 0.217358
1 7.39337 0.852735 0.517821
1 2.03601 2.80265 -0.828508

6 -4.34539 0.019714 1.14971
6 -4.94271 -0.78828 2.11575
6 -5.14485 0.612723 0.166457
6 -6.32056 -0.999245 2.11001
1 -4.32482 -1.25302 2.88062
6 -6.51879 0.398149 0.156072
1 -4.68315 1.24246 -0.593785
6 -7.10987 -0.407118 1.12945
1 -6.77438 -1.62851 2.86951
1 -7.13136 0.863678 -0.609945
1 -8.18293 -0.571002 1.1217

Model complex with N-Bn on azetidine nitrogen

SCF energy= -1461.32752570
Zero-point correction= 0.478616
Thermal correction to Energy= 0.506956
Thermal correction to Enthalpy= 0.507900
Thermal correction to Gibbs Free Energy= 0.418655
SCF energy+Solvation free energy= -1461.34087427

Coordinates:
6 -0.399342 1.27222 2.4622
1 0.524828 1.08083 3.01716
6 -0.891727 -0.049179 1.84007
7 -0.075534 2.27653 1.44683
1 -0.886017 2.59236 0.898002
7 -0.591122 -0.248869 0.390432
29 1.07835 1.36346 -0.005834
8 0.378135 2.19606 -1.58751
8 2.90829 0.78908 -0.753325
1 -1.9618 -0.195624 2.03092
6 -0.592701 3.03974 -1.67057
8 -1.35124 3.3654 -0.747212
6 -0.782224 3.63194 -3.05507
1 -1.01321 2.82885 -3.76081
1 0.150945 4.09484 -3.38571
1 -1.58883 4.36462 -3.04841
6 -1.6679 0.032656 -0.58779
1 -1.86321 1.1095 -0.548444
1 -1.25705 -0.178527 -1.58111
6 0.584637 3.4588 2.00879
1 0.69883 4.20159 1.21595
1 1.57115 3.17295 2.38655
1 0.002752 3.89989 2.82824
1 -1.14228 1.66405 3.17059
6 -0.09833 -1.33122 2.15915
6 -0.236436 -1.66784 0.66313
1 0.937546 -1.11074 2.42458
1 -0.548078 -2.02856 2.86674
1 -1.11348 -2.31345 0.514688
6 0.940705 -2.24932 -0.071827
6 1.5693 -3.37663 0.468044
6 1.40082 -1.73327 -1.28196

6 2.63451 -3.97992 -0.190624
1 1.21629 -3.78124 1.41417
6 2.47967 -2.32809 -1.93728
1 0.942762 -0.844756 -1.71047
6 3.09353 -3.45438 -1.39919
1 3.11164 -4.85476 0.240689
1 2.84157 -1.89734 -2.86537
1 3.93091 -3.91751 -1.91174
6 3.16024 0.306098 0.383617
8 2.32857 0.507445 1.33224
6 4.36472 -0.555741 0.617705
1 4.78645 -0.358751 1.60493
1 5.10654 -0.397701 -0.164893
1 4.02939 -1.59914 0.587176
6 -2.95798 -0.728675 -0.387529
6 -3.99132 -0.182747 0.37913
6 -3.14635 -1.98975 -0.962599
6 -5.17406 -0.886676 0.588621
1 -3.87026 0.813234 0.800296
6 -4.32698 -2.69773 -0.756719
1 -2.3597 -2.41334 -1.5844
6 -5.34162 -2.14873 0.024069
1 -5.96797 -0.446107 1.1838
1 -4.45806 -3.67471 -1.21157
1 -6.2645 -2.69773 0.183049

Model complex with N-Bn on azetidine nitrogen

SCF energy= -1461.33022122
Zero-point correction= 0.478794
Thermal correction to Energy= 0.507911
Thermal correction to Enthalpy= 0.508855
Thermal correction to Gibbs Free Energy= 0.417285
SCF energy+Solvation free energy= -1461.34384869

Coordinates:
6 1.69443 2.14616 -1.80294
1 1.231 3.02891 -1.35182
6 0.598112 1.19402 -2.29924
7 2.51456 1.52339 -0.765628
1 3.00453 0.673512 -1.08145
7 0.125169 0.134627 -1.34506
29 1.23641 0.772462 0.647565
8 2.43732 -0.615352 1.2756
8 -0.107236 0.793669 2.16189
1 0.900833 0.72015 -3.24331
6 3.41437 -1.17119 0.64228
8 3.77609 -0.905422 -0.51191
6 4.11108 -2.27245 1.41951
1 3.4598 -3.15311 1.4318
1 4.26759 -1.96418 2.45503
1 5.05601 -2.53473 0.944015
6 0.588227 -1.22643 -1.7028
1 0.464145 -1.37184 -2.78942

1 1.66003 -1.2848 -1.47668
6 3.50075 2.43744 -0.18175
1 4.10946 1.87961 0.533551
1 2.97932 3.24586 0.339639
1 4.15584 2.87027 -0.948022
1 2.31373 2.47984 -2.64739
6 -0.826071 1.76191 -2.37558
6 -1.29022 0.442926 -1.73671
1 -0.958827 2.60076 -1.68721
1 -1.21713 2.00537 -3.36414
1 -1.59989 -0.266551 -2.52012
6 -2.37755 0.518666 -0.693165
6 -3.44898 1.38516 -0.937028
6 -2.40534 -0.277313 0.451999
6 -4.53345 1.44491 -0.066731
1 -3.43727 2.01616 -1.82296
6 -3.48657 -0.21144 1.32909
1 -1.57532 -0.934907 0.679171
6 -4.55625 0.640565 1.07126
1 -5.35671 2.12144 -0.275633
1 -3.48355 -0.832597 2.21955
1 -5.39819 0.686014 1.75516
6 -0.547508 1.89257 1.71646
8 0.038992 2.42644 0.718614
6 -1.74972 2.55804 2.31746
1 -1.49789 3.58589 2.5909
1 -2.10067 2.00355 3.18705
1 -2.53648 2.59153 1.5566
6 -0.178429 -2.30365 -0.975885
6 -1.27028 -2.91985 -1.5914
6 0.150493 -2.66545 0.334743
6 -2.03725 -3.8607 -0.908837
1 -1.52329 -2.66216 -2.61745
6 -0.613148 -3.60844 1.01684
1 0.982228 -2.17972 0.837604
6 -1.71152 -4.20416 0.399923
1 -2.88673 -4.32498 -1.39995
1 -0.351441 -3.87246 2.03693
1 -2.30748 -4.93672 0.935505

Single crystal X-ray diffraction data

CDCC Deposition Numbers

CCDC 1824096-1824097

XRD Data tables for compound (2S, 4R)-1d

The dataset was measured on an Agilent SuperNova diffractometer using an Atlas detector. The data collection was driven and processed and an absorption correction was applied using CrysAlisPro.[26] The structure was solved using ShelXS[2] and refined by a full-matrix least-squares procedure on F^2 in ShelXL.[27] All non-hydrogen atoms were refined with anisotropic displacement parameters. All hydrogen atoms were added at calculated positions and refined by use of a riding model with isotropic displacement parameters based on the equivalent isotropic displacement parameter (U_{eq}) of the parent atom. Reports were produced using OLEX2.[28]

Table 1 Crystal data and structure refinement for (2*S*, 4*R*)-1d.

Identification code	**(2*S*, 4*R*)-1d**
Empirical formula	$C_{20}H_{26}IN_3O_2$
Formula weight	467.34
Temperature/K	99.9(4)
Crystal system	monoclinic
Space group	$P2_1$
a/Å	8.26967(7)
b/Å	7.63280(6)
c/Å	16.69601(11)
α/°	90
β/°	96.4510(7)
γ/°	90
Volume/Å^3	1047.191(14)
Z	2
ρ_{calc}g/cm^3	1.482
μ/mm^{-1}	12.155
F(000)	472.0
Crystal size/mm^3	0.3448 × 0.1981 × 0.0956
Radiation	CuKα (λ = 1.54184)
2Θ range for data collection/°	5.326 to 148.828
Index ranges	-9 ≤ h ≤ 10, -9 ≤ k ≤ 9, -20 ≤ l ≤ 20
Reflections collected	19506
Independent reflections	4208 [R_{int} = 0.0237, R_{sigma} = 0.0151]
Data/restraints/parameters	4208/1/237
Goodness-of-fit on F^2	1.068
Final R indexes [I>=2σ (I)]	R_1 = 0.0151, wR_2 = 0.0386
Final R indexes [all data]	R_1 = 0.0153, wR_2 = 0.0388
Largest diff. peak/hole / e Å^{-3}	0.21/-0.58
Flack parameter	-0.017(2)

Table 2 Fractional Atomic Coordinates (×10[4]) and Equivalent Isotropic Displacement Parameters (Å[2]×10[3]) for (2*S*, 4*R*)-1d. U_{eq} is defined as 1/3 of of the trace of the orthogonalised U_{IJ} tensor.

Atom	*x*	*y*	*z*	U(eq)
C(1)	7564(3)	7308(5)	2586.1(14)	17.6(5)
C(2)	6873(3)	7786(4)	1702.8(15)	21.0(6)
C(3)	5228(3)	8067(3)	2038.6(15)	18.1(5)
C(4)	3721(3)	7251(4)	1605.6(14)	18.7(6)
C(5)	8689(3)	5772(3)	2701.3(14)	16.6(5)
C(6)	10354(3)	6050(3)	2895.0(16)	19.7(5)
C(7)	11458(4)	4680(4)	2930.5(17)	21.0(6)
C(8)	10856(3)	3003(3)	2779.2(15)	19.3(5)
C(9)	9208(3)	2674(5)	2598.6(14)	22.8(5)
C(10)	8125(4)	4059(4)	2561.2(16)	21.3(5)
C(11)	5482(3)	7673(6)	3552.9(14)	22.8(6)
C(12)	6410(3)	6583(5)	4205.6(15)	24.5(6)
C(13)	7722(3)	7286(7)	4691.6(15)	32.3(8)
C(14)	8657(4)	6232(6)	5247(2)	44.8(10)
C(15)	8277(5)	4489(7)	5321(2)	46.9(10)
C(16)	6953(5)	3775(5)	4849(2)	41.0(8)
C(17)	6027(4)	4824(5)	4289.8(16)	30.5(7)
C(18)	1708(3)	7414(6)	348.4(13)	20.7(5)
C(19)	1550(4)	8070(5)	-513.2(17)	33.3(8)
C(20)	357(4)	8130(5)	793.1(18)	33.5(8)
N(1)	5856(3)	7045(3)	2766.5(12)	17.5(5)
N(2)	3340(3)	7967(3)	768.5(12)	17.0(5)
N(3)	12020(3)	1548(3)	2807.6(14)	24.9(5)
O(1)	13452(3)	1837(3)	3041.3(14)	31.2(5)
O(2)	11490(3)	87(3)	2593.6(15)	33.7(5)
I(1)	3796.0(2)	2507.5(3)	739.2(2)	19.92(5)

Table 3 Anisotropic Displacement Parameters (Å[2]×10[3]) for (2*S*, 4*R*)-1d. The Anisotropic displacement factor exponent takes the form: $-2\pi^2[h^2a^{*2}U_{11}+2hka^*b^*U_{12}+\ldots]$.

Atom	U_{11}	U_{22}	U_{33}	U_{23}	U_{13}	U_{12}
C(1)	15(1)	18.3(15)	19.1(9)	-0.9(11)	0.2(8)	-1.8(12)
C(2)	17.6(11)	26.1(19)	19.6(10)	3.4(10)	2.8(8)	3.9(11)
C(3)	17.5(12)	18.5(12)	18.3(11)	0.3(8)	2.0(9)	3.3(9)
C(4)	21.4(12)	17.2(16)	17.3(10)	0.8(10)	0.8(8)	1.9(11)
C(5)	17.4(12)	18.9(12)	13.3(10)	0.2(9)	0.5(9)	1.2(10)
C(6)	21.5(14)	15.7(12)	21.5(12)	-0.4(9)	0.6(10)	-1.2(10)
C(7)	18.5(14)	19.4(14)	24.5(14)	0.3(11)	-1.2(11)	0.8(11)
C(8)	22.6(13)	18.3(14)	17.1(11)	0.9(8)	2.8(9)	4.3(10)
C(9)	28.1(12)	15.6(15)	24.5(10)	-1.1(13)	2.0(9)	-2.8(14)
C(10)	19.5(14)	21.4(14)	22.7(12)	-0.8(10)	1(1)	-1.9(11)
C(11)	21.5(11)	28.5(16)	18.5(10)	-3.8(13)	2.8(8)	3.6(14)
C(12)	20.7(14)	38.4(18)	15.0(12)	-1.2(11)	4.9(10)	4.3(12)
C(13)	26.2(13)	51(2)	19.1(10)	-5.4(15)	0.6(9)	1.0(17)
C(14)	28.3(18)	85(3)	19.8(14)	0.7(16)	-2.8(13)	5.2(19)
C(15)	36(2)	82(3)	22.6(15)	18.2(16)	5.2(13)	20(2)

$C^{(16)}$	41(2)	54(2)	30.1(15)	17.5(15)	11.0(14)	11.4(17)
$C^{(17)}$	28.7(17)	40(2)	22.9(14)	5.7(11)	4.3(12)	4.3(13)
$C^{(18)}$	17.4(11)	21.4(12)	22(1)	-2.1(15)	-3.6(8)	-2.7(15)
$C^{(19)}$	18.5(13)	61(2)	19.5(12)	1.0(12)	-1(1)	-0.9(13)
$C^{(20)}$	15.1(13)	60(2)	24.5(13)	0.4(12)	0.4(11)	-1.2(13)
$N^{(1)}$	14.7(10)	21.9(13)	15.7(9)	0.7(7)	0.5(7)	3.5(8)
$N^{(2)}$	14(1)	19.8(13)	17.0(9)	-1.0(7)	1.0(7)	1.3(8)
$N^{(3)}$	32.2(15)	20.2(13)	23.4(11)	1.5(9)	7.1(10)	7.1(11)
$O^{(1)}$	23.9(11)	28.9(10)	40.2(12)	-0.5(9)	0.7(9)	9.3(8)
$O^{(2)}$	43.1(14)	16.8(11)	41.6(13)	-4(1)	6.8(11)	4.1(10)
$I^{(1)}$	19.74(8)	19.04(8)	21.14(7)	-0.65(8)	2.99(5)	-0.04(9)

Table 4 Bond Lengths for (2*S*, 4*R*)-1d .

Atom	Atom	Length/Å	Atom	Atom	Length/Å
$C^{(1)}$	$C^{(2)}$	1.564(3)	$C^{(11)}$	$C^{(12)}$	1.510(4)
$C^{(1)}$	$C^{(5)}$	1.496(4)	$C^{(11)}$	$N^{(1)}$	1.463(3)
$C^{(1)}$	$N^{(1)}$	1.491(3)	$C^{(12)}$	$C^{(13)}$	1.388(4)
$C^{(2)}$	$C^{(3)}$	1.543(3)	$C^{(12)}$	$C^{(17)}$	1.391(5)
$C^{(3)}$	$C^{(4)}$	1.504(4)	$C^{(13)}$	$C^{(14)}$	1.394(5)
$C^{(3)}$	$N^{(1)}$	1.488(3)	$C^{(14)}$	$C^{(15)}$	1.376(7)
$C^{(4)}$	$N^{(2)}$	1.501(3)	$C^{(15)}$	$C^{(16)}$	1.386(6)
$C^{(5)}$	$C^{(6)}$	1.394(4)	$C^{(16)}$	$C^{(17)}$	1.392(4)
$C^{(5)}$	$C^{(10)}$	1.399(4)	$C^{(18)}$	$C^{(19)}$	1.515(4)
$C^{(6)}$	$C^{(7)}$	1.385(4)	$C^{(18)}$	$C^{(20)}$	1.511(4)
$C^{(7)}$	$C^{(8)}$	1.386(4)	$C^{(18)}$	$N^{(2)}$	1.510(3)
$C^{(8)}$	$C^{(9)}$	1.385(4)	$N^{(3)}$	$O^{(1)}$	1.224(3)
$C^{(8)}$	$N^{(3)}$	1.468(3)	$N^{(3)}$	$O^{(2)}$	1.236(3)
$C^{(9)}$	$C^{(10)}$	1.383(5)			

Table 5 Bond Angles for (2*S*, 4*R*)-1d .

Atom	Atom	Atom	Angle/˚	Atom	Atom	Atom	Angle/˚
$C^{(5)}$	$C^{(1)}$	$C^{(2)}$	117.4(2)	$C^{(13)}$	$C^{(12)}$	$C^{(11)}$	120.6(3)
$N^{(1)}$	$C^{(1)}$	$C^{(2)}$	88.17(17)	$C^{(13)}$	$C^{(12)}$	$C^{(17)}$	119.1(3)
$N^{(1)}$	$C^{(1)}$	$C^{(5)}$	117.3(3)	$C^{(17)}$	$C^{(12)}$	$C^{(11)}$	120.1(3)
$C^{(3)}$	$C^{(2)}$	$C^{(1)}$	86.17(18)	$C^{(12)}$	$C^{(13)}$	$C^{(14)}$	120.3(4)
$C^{(4)}$	$C^{(3)}$	$C^{(2)}$	119.0(2)	$C^{(15)}$	$C^{(14)}$	$C^{(13)}$	120.2(4)
$N^{(1)}$	$C^{(3)}$	$C^{(2)}$	89.04(19)	$C^{(14)}$	$C^{(15)}$	$C^{(16)}$	120.1(3)
$N^{(1)}$	$C^{(3)}$	$C^{(4)}$	111.7(2)	$C^{(15)}$	$C^{(16)}$	$C^{(17)}$	119.7(4)
$N^{(2)}$	$C^{(4)}$	$C^{(3)}$	111.9(2)	$C^{(12)}$	$C^{(17)}$	$C^{(16)}$	120.6(3)
$C^{(6)}$	$C^{(5)}$	$C^{(1)}$	119.6(2)	$C^{(20)}$	$C^{(18)}$	$C^{(19)}$	111.0(3)
$C^{(6)}$	$C^{(5)}$	$C^{(10)}$	118.9(2)	$N^{(2)}$	$C^{(18)}$	$C^{(19)}$	109.0(2)
$C^{(10)}$	$C^{(5)}$	$C^{(1)}$	121.3(2)	$N^{(2)}$	$C^{(18)}$	$C^{(20)}$	110.0(2)
$C^{(7)}$	$C^{(6)}$	$C^{(5)}$	121.6(3)	$C^{(3)}$	$N^{(1)}$	$C^{(1)}$	90.89(19)
$C^{(6)}$	$C^{(7)}$	$C^{(8)}$	117.8(3)	$C^{(11)}$	$N^{(1)}$	$C^{(1)}$	116.0(2)
$C^{(7)}$	$C^{(8)}$	$N^{(3)}$	118.1(2)	$C^{(11)}$	$N^{(1)}$	$C^{(3)}$	118.2(2)
$C^{(9)}$	$C^{(8)}$	$C^{(7)}$	122.1(3)	$C^{(4)}$	$N^{(2)}$	$C^{(18)}$	114.4(2)

$C^{(9)}$	$C^{(8)}$	$N^{(3)}$	119.7(3)	$O^{(1)}$	$N^{(3)}$	$C^{(8)}$	118.8(2)
$C^{(10)}$	$C^{(9)}$	$C^{(8)}$	119.2(3)	$O^{(1)}$	$N^{(3)}$	$O^{(2)}$	123.6(3)
$C^{(9)}$	$C^{(10)}$	$C^{(5)}$	120.3(3)	$O^{(2)}$	$N^{(3)}$	$C^{(8)}$	117.6(3)
$N^{(1)}$	$C^{(11)}$	$C^{(12)}$	109.0(3)				

Table 6 Hydrogen Atom Coordinates (Å×10^4) and Isotropic Displacement Parameters (Å^2×10^3) for (2*S*, 4*R*)-1d.

Atom	*x*	*y*	*z*	U(eq)
$H^{(1)}$	8056	8361	2875	21
$H^{(2A)}$	7344	8862	1493	25
$H^{(2B)}$	6879	6804	1316	25
$H^{(3)}$	5052	9326	2170	22
$H^{(4A)}$	3874	5967	1576	22
$H^{(4B)}$	2789	7474	1915	22
$H^{(6)}$	10739	7207	3005	24
$H^{(7)}$	12592	4883	3054	25
$H^{(9)}$	8826	1510	2502	27
$H^{(10)}$	6992	3847	2440	26
$H^{(11A)}$	4298	7576	3589	27
$H^{(11B)}$	5797	8920	3622	27
$H^{(13)}$	7984	8491	4646	39
$H^{(14)}$	9558	6719	5575	54
$H^{(15)}$	8924	3772	5696	56
$H^{(16)}$	6680	2577	4906	49
$H^{(17)}$	5125	4333	3963	37
$H^{(18)}$	1647	6106	344	25
$H^{(19A)}$	1680	9346	-515	50
$H^{(19B)}$	474	7759	-783	50
$H^{(19C)}$	2394	7529	-799	50
$H^{(20A)}$	420	7597	1330	50
$H^{(20B)}$	-697	7852	491	50
$H^{(20C)}$	473	9403	847	50
$H^{(2C)}$	4130	7615	466	20
$H^{(2D)}$	3371	9158	793	20

Experimental

Single crystals of $C_{20}H_{26}IN_3O_2$ **[(2*S*, 4*R*)-1d]** mounted on a **SuperNova, Dual, Cu at zero, Atlas** diffractometer. The crystal was kept at 99.9(4) K during data collection. Using Olex2,[28] the structure was solved with the ShelXS[27] structure solution program using Patterson Method and refined with the ShelXL[29] refinement package using Least Squares minimisation.

Crystal structure determination of [(2*S*, 4*R*)-1d]

Crystal Data for $C_{20}H_{26}IN_3O_2$ (M =467.34 g/mol): monoclinic, space group $P2_1$ (no. 4), a = 8.26967(7) Å, b = 7.63280(6) Å, c = 16.69601(11) Å, β = 96.4510(7)°, V = 1047.191(14) Å^3, Z = 2, T = 99.9(4) K, μ(CuKα) = 12.155 mm^{-1}, $Dcalc$ = 1.482 g/cm^3, 19506 reflections measured (5.326° ≤ 2Θ ≤ 148.828°), 4208 unique (R_{int} = 0.0237, R_{sigma} = 0.0151) which were used in all calculations. The final R_1 was 0.0151 (I > 2σ(I)) and wR_2 was 0.0388 (all data).

Refinement model description

Number of restraints - 1, number of constraints - unknown.

Details:

1. Fixed Uiso

At 1.2 times of:

All C(H) groups, All C(H,H) groups, All N(H,H) groups

At 1.5 times of:
All C(H,H,H) groups
2.a Ternary CH refined with riding coordinates:
C1(H1), C3(H3), C18(H18)
2.b Secondary CH2 refined with riding coordinates:
C2(H2A,H2B), C4(H4A,H4B), C11(H11A,H11B), N2(H2C,H2D)
2.c Aromatic/amide H refined with riding coordinates:
C6(H6), C7(H7), C9(H9), C10(H10), C13(H13), C14(H14), C15(H15), C16(H16), C17(H17)
2.d Idealised Me refined as rotating group:
C19(H19A,H19B,H19C), C20(H20A,H20B,H20C)

This report has been created with Olex2, compiled on 2017.08.10 svn.r3458 for OlexSys. Please let us know if there are any errors or if you would like to have additional features.

*XRD Data tables for (*rac*)-7*

7: The dataset was measured by the EPSRC UK National Crystallography Service[30] on a Bruker-Nonius Roper CCD diffractometer at the window of a Bruker-Nonius FR591 rotating anode. The data collection was driven by COLLECT[31] and processed by DENZO[32] and an absorption correction was applied using SADABS[33] The structure was solved in Sir2004,[34] and was refined by a full-matrix least-squares procedure on F^2 in SHELXL-97.[27] All non-hydrogen atoms were refined with anisotropic displacement parameters. The hydrogen atoms bonded to N(2), N(102), N(202) and N(302) (one per crystallographically-independent platinum complex) were located in the electron density and their positions refined subject to suitable distance restraints, (N-H = 0.91 (2) Å and Pd … H = 2.48 (4) Å and C(104)-H(12C) = C(112)-H(12C) = 1.92 (4) Å). The isotropic thermal parameters (U_{iso}) of these hydrogen atoms were set at 1.2 times the equivalent isotropic thermal parameter (U_{eq}) of the parent nitrogen atom. All remaining hydrogen atoms were added at calculated positions and refined by use of a riding model with isotropic displacement parameters based on the equivalent isotropic displacement parameter (U_{eq}) of the parent atom. The structure contains four crystallographically-independent platinum complexes with one acetonitrile molecule per platinum complex.

Table 1. Crystal data and structure refinement for **(*rac*)-7**.

Identification code	**(*rac*)-7**, 2010src0915	
Empirical formula	$C_{24}H_{26}Cl_2N_2Pt$, C_2H_3N	
Formula weight	649.51	
Temperature	120(2) K	
Wavelength	0.71073 Å	
Crystal system	Monoclinic	
Space group	P 2(1)	
Unit cell dimensions	a = 10.6958(2) Å	α= 90°.
	b = 10.4600(2) Å	β= 93.9630(10)°.
	c = 43.9267(10) Å	χ = 90°.
Volume	4902.68(17) Å^3	
Z, Z'	8, 4	
Density (calculated)	1.760 Mg/m^3	
Absorption coefficient	5.961 mm^{-1}	
F(000)	2544	
Crystal size	0.16 x 0.12 x 0.10 mm^3	
Theta range for data collection	2.92 to 25.03°.	

Index ranges	-12<=h<=12, -12<=k<=12, -52<=l<=52
Reflections collected	44832
Independent reflections	16268 [R(int) = 0.1064]
Completeness to theta = 25.03°	97.1 %
Absorption correction	Semi-empirical from equivalents
Max. and min. transmission	0.5871 and 0.4489
Refinement method	Full-matrix least-squares on F^2
Data / restraints / parameters	16268 / 277 / 1165
Goodness-of-fit on F^2	1.043
Final R indices [I>2sigma(I)]	R1 = 0.0623, wR2 = 0.0856
R indices (all data)	R1 = 0.0871, wR2 = 0.0908
Absolute structure parameter	0.041(7)
Largest diff. peak and hole	1.236 and -1.882 e.Å^{-3}

Notes:
The structure contains four crystallographically-independent platinum complexes with one acetonitrile molecule per platinum complex.
The hydrogen atoms bonded to N(2), N(102), N(202) and N(302) (one per crystallographically-independent platinum complex) were located in the electron density and their positions refined subject to suitable distance restraints, (N-H = 0.91 (2) Å and Pd … H = 2.48 (4) Å and C(104)-H(12C) = C(112)-H(12C) = 1.92 (4) Å). The isotropic thermal parameters (U_{iso}) of these hydrogen atoms were set at 1.2 times the equivalent isotropic thermal parameter (U_{eq}) of the parent nitrogen atom. All remaining hydrogen atoms were fixed as riding models. The hydrogen bonding is detailed in Table 7, of this section.

Table 2. Atomic coordinates (x 10^4) and equivalent isotropic displacement parameters ($Å^2$x 10^3) for ***(rac)*-7**. U(eq) is defined as one third of the trace of the orthogonalized U^{ij} tensor.

	x	y	z	U(eq)
C(1)	4373(10)	1349(10)	4311(2)	24(2)
C(2)	4417(10)	2692(10)	4176(2)	27(2)
C(3)	3934(9)	2097(9)	3869(2)	22(2)
C(4)	4454(8)	2506(10)	3573(2)	21(2)
C(5)	3592(9)	-336(10)	3927(2)	23(2)
C(6)	3359(9)	-760(10)	3599(3)	24(2)
C(7)	2369(11)	-234(11)	3425(3)	38(3)
C(8)	2076(12)	-718(13)	3140(3)	45(3)
C(9)	2752(13)	-1712(13)	3016(3)	47(3)
C(10)	3747(12)	-2219(12)	3197(3)	42(3)
C(11)	4045(10)	-1746(10)	3489(3)	28(3)
C(12)	6385(10)	2546(10)	3294(2)	26(2)
C(13)	5763(10)	1802(10)	3029(2)	29(2)
C(14)	5900(11)	505(11)	2994(2)	38(3)
C(15)	5379(13)	-131(12)	2743(3)	50(3)
C(16)	4645(11)	482(14)	2522(3)	47(3)
C(17)	4538(14)	1797(13)	2549(3)	60(4)
C(18)	5077(11)	2434(12)	2803(2)	45(3)
C(19)	5241(9)	865(9)	4566(2)	20(2)
C(20)	5029(9)	-339(9)	4688(2)	21(2)
C(21)	5781(10)	-815(10)	4933(2)	28(2)
C(22)	6736(11)	-71(11)	5062(3)	32(3)
C(23)	6952(10)	1112(12)	4950(3)	35(3)
C(24)	6217(9)	1582(11)	4697(2)	29(3)
N(1)	4432(7)	792(8)	3982(2)	24(2)
N(2)	5836(7)	2299(9)	3598(2)	22(2)
Cl(1)	6644(2)	-1248(2)	4098(1)	22(1)

Cl(2)	8233(2)	506(3)	3661(1)	23(1)
Pt(1)	6231(1)	612(1)	3828(1)	18(1)
C(101)	8463(9)	1280(11)	684(2)	23(2)
C(102)	7203(9)	1239(12)	828(2)	23(2)
C(103)	7907(9)	1661(10)	1127(2)	20(2)
C(104)	7656(10)	1083(11)	1427(3)	24(3)
C(105)	10241(9)	2079(10)	1043(3)	24(3)
C(106)	10727(10)	2330(12)	1369(2)	26(3)
C(107)	10204(10)	3265(12)	1541(3)	31(3)
C(108)	10681(12)	3548(13)	1831(3)	46(4)
C(109)	11707(14)	2872(13)	1948(3)	48(4)
C(110)	12296(13)	1988(12)	1785(3)	42(3)
C(111)	11791(11)	1662(11)	1497(3)	30(3)
C(112)	7735(9)	-922(10)	1724(3)	26(3)
C(113)	8519(11)	-301(11)	1983(3)	35(3)
C(114)	9816(10)	-463(13)	2007(3)	33(3)
C(115)	10502(13)	72(13)	2252(3)	47(4)
C(116)	9906(14)	759(14)	2473(3)	50(3)
C(117)	8646(14)	898(13)	2456(3)	52(4)
C(118)	7936(12)	348(13)	2204(3)	46(3)
C(119)	8776(9)	454(11)	425(2)	24(2)
C(120)	9948(10)	538(13)	311(2)	31(2)
C(121)	10247(13)	-155(13)	53(3)	44(3)
C(122)	9379(13)	-889(12)	-95(3)	42(3)
C(123)	8158(14)	-979(11)	9(3)	43(3)
C(124)	7889(12)	-328(10)	271(3)	32(3)
N(101)	9140(7)	1207(9)	1007(2)	19(2)
N(102)	7885(8)	-311(8)	1416(2)	23(2)
Cl(11)	11247(2)	-933(2)	925(1)	24(1)
Cl(12)	9695(2)	-2689(3)	1372(1)	25(1)
Pt(11)	9460(1)	-628(1)	1182(1)	18(1)
C(201)	10235(9)	6385(9)	4329(2)	21(2)
C(202)	10125(9)	7742(9)	4193(2)	22(2)
C(203)	10465(9)	7148(9)	3887(2)	20(2)
C(204)	9800(9)	7546(10)	3593(2)	25(2)
C(205)	10859(10)	4721(10)	3951(2)	26(3)
C(206)	10967(9)	4322(12)	3628(3)	27(3)
C(207)	10290(10)	3250(10)	3506(3)	27(3)
C(208)	10498(12)	2819(11)	3215(3)	38(3)
C(209)	11370(12)	3405(11)	3043(3)	38(3)
C(210)	12030(11)	4483(13)	3157(3)	41(3)
C(211)	11818(10)	4906(11)	3454(3)	29(3)
C(212)	7764(9)	7516(10)	3288(2)	24(2)
C(213)	8259(9)	6793(11)	3033(2)	26(2)
C(214)	8107(10)	5459(12)	3012(3)	34(3)
C(215)	8533(14)	4787(13)	2770(3)	50(4)
C(216)	9143(13)	5462(18)	2543(3)	59(4)
C(217)	9278(15)	6752(17)	2556(3)	65(4)
C(218)	8825(10)	7419(12)	2801(3)	36(3)
C(219)	9506(8)	5875(9)	4575(2)	20(2)
C(220)	9776(9)	4686(10)	4706(2)	26(2)
C(221)	9156(10)	4170(11)	4944(3)	31(3)
C(222)	8256(11)	4937(13)	5070(3)	39(3)
C(223)	7985(10)	6138(13)	4956(2)	33(3)
C(224)	8584(10)	6617(11)	4709(2)	29(3)
N(201)	10011(7)	5842(7)	4002(2)	19(2)
N(202)	8438(7)	7286(9)	3601(2)	21(2)

Cl(21)	7889(2)	3754(2)	4099(1)	22(1)
Cl(22)	6077(2)	5499(3)	3654(1)	23(1)
Pt(21)	8162(1)	5625(1)	3833(1)	17(1)
C(301)	3462(9)	5499(12)	688(2)	21(2)
C(302)	2201(8)	5561(14)	834(2)	26(2)
C(303)	2923(9)	5114(10)	1134(2)	21(2)
C(304)	2658(8)	5689(11)	1430(2)	17(2)
C(305)	5271(9)	4732(10)	1048(3)	26(3)
C(306)	5777(9)	4509(11)	1373(2)	22(2)
C(307)	6754(10)	5227(12)	1503(3)	36(3)
C(308)	7255(14)	5001(13)	1789(3)	50(4)
C(309)	6758(14)	3980(13)	1967(3)	50(4)
C(310)	5765(13)	3288(14)	1840(3)	47(4)
C(311)	5274(12)	3518(12)	1535(3)	38(3)
C(312)	2719(10)	7685(11)	1731(2)	26(3)
C(313)	3536(9)	7139(10)	1986(2)	21(2)
C(314)	4823(10)	7403(13)	2031(3)	35(3)
C(315)	5571(12)	6879(12)	2274(3)	39(3)
C(316)	5066(12)	6087(13)	2477(3)	46(3)
C(317)	3815(12)	5783(14)	2432(3)	47(3)
C(318)	3067(11)	6317(14)	2195(3)	40(3)
C(319)	3806(10)	6380(10)	435(2)	24(3)
C(320)	4997(11)	6275(12)	318(2)	32(3)
C(321)	5268(12)	6945(12)	63(3)	36(3)
C(322)	4336(14)	7717(12)	-91(3)	46(4)
C(323)	3178(13)	7787(12)	16(3)	41(3)
C(324)	2891(12)	7112(10)	276(3)	33(3)
N(301)	4155(7)	5613(10)	1010(2)	20(1)
N(302)	2888(8)	7111(9)	1424(2)	24(2)
Cl(31)	6230(2)	7767(2)	924(1)	23(1)
Cl(32)	4663(2)	9527(2)	1372(1)	25(1)
Pt(31)	4451(1)	7448(1)	1187(1)	18(1)
C(401)	3472(11)	1302(14)	642(3)	31(3)
N(401)	3093(11)	2284(11)	690(3)	52(3)
C(402)	3953(11)	74(12)	576(3)	33(3)
C(501)	4321(11)	6358(11)	4315(2)	28(3)
C(502)	5562(9)	6008(10)	4441(3)	29(3)
N(501)	3349(10)	6666(11)	4220(3)	52(3)
C(601)	8457(10)	5490(13)	639(2)	25(2)
C(602)	8921(11)	6786(10)	583(3)	32(3)
N(601)	8061(10)	4483(11)	681(3)	49(3)
C(701)	300(10)	1384(10)	4311(3)	26(2)
C(702)	-849(9)	1030(10)	4449(3)	28(3)
N(701)	1207(9)	1620(10)	4211(3)	47(3)

Table 3. Bond lengths [Å] and angles [°] for ***(rac)*-7**.

C(1)-C(19)	1.495(15)
C(1)-C(2)	1.526(15)
C(1)-N(1)	1.564(12)
C(1)-H(1)	1.0000
C(2)-C(3)	1.545(15)
C(2)-H(2A)	0.9900
C(2)-H(2B)	0.9900
C(3)-C(4)	1.511(13)
C(3)-N(1)	1.535(13)

C(3)-H(3)	1.0000
C(4)-N(2)	1.491(12)
C(4)-H(4A)	0.9900
C(4)-H(4B)	0.9900
C(5)-N(1)	1.492(13)
C(5)-C(6)	1.513(15)
C(5)-H(5A)	0.9900
C(5)-H(5B)	0.9900
C(6)-C(11)	1.373(15)
C(6)-C(7)	1.377(16)
C(7)-C(8)	1.367(18)
C(7)-H(7)	0.9500
C(8)-C(9)	1.397(18)
C(8)-H(8)	0.9500
C(9)-C(10)	1.387(18)
C(9)-H(9)	0.9500
C(10)-C(11)	1.391(16)
C(10)-H(10)	0.9500
C(11)-H(11)	0.9500
C(12)-C(13)	1.515(15)
C(12)-N(2)	1.517(13)
C(12)-H(12A)	0.9900
C(12)-H(12B)	0.9900
C(13)-C(18)	1.363(12)
C(13)-C(14)	1.374(13)
C(14)-C(15)	1.374(13)
C(14)-H(14)	0.9500
C(15)-C(16)	1.365(14)
C(15)-H(15)	0.9500
C(16)-C(17)	1.385(14)
C(16)-H(16)	0.9500
C(17)-C(18)	1.390(14)
C(17)-H(17)	0.9500
C(18)-H(18)	0.9500
C(19)-C(24)	1.378(14)
C(19)-C(20)	1.392(13)
C(20)-C(21)	1.392(15)
C(20)-H(20)	0.9500
C(21)-C(22)	1.375(16)
C(21)-H(21)	0.9500
C(22)-C(23)	1.359(16)
C(22)-H(22)	0.9500
C(23)-C(24)	1.403(16)
C(23)-H(23)	0.9500
C(24)-H(24)	0.9500
N(1)-Pt(1)	2.092(7)
N(2)-Pt(1)	2.062(9)
N(2)-H(2C)	0.92(2)
Cl(1)-Pt(1)	2.306(2)
Cl(2)-Pt(1)	2.314(2)
C(101)-C(119)	1.486(15)
C(101)-C(102)	1.529(14)
C(101)-N(101)	1.547(13)
C(101)-H(101)	1.0000
C(102)-C(103)	1.532(14)
C(102)-H(10A)	0.9900
C(102)-H(10B)	0.9900

C(103)-C(104)	1.492(15)
C(103)-N(101)	1.530(12)
C(103)-H(103)	1.0000
C(104)-N(102)	1.479(14)
C(104)-H(10C)	0.9900
C(104)-H(10D)	0.9900
C(105)-N(101)	1.490(13)
C(105)-C(106)	1.512(15)
C(105)-H(10E)	0.9900
C(105)-H(10F)	0.9900
C(106)-C(107)	1.378(16)
C(106)-C(111)	1.419(16)
C(107)-C(108)	1.374(18)
C(107)-H(107)	0.9500
C(108)-C(109)	1.38(2)
C(108)-H(108)	0.9500
C(109)-C(110)	1.352(18)
C(109)-H(109)	0.9500
C(110)-C(111)	1.383(17)
C(110)-H(110)	0.9500
C(111)-H(111)	0.9500
C(112)-C(113)	1.511(17)
C(112)-N(102)	1.516(14)
C(112)-H(11A)	0.9900
C(112)-H(11B)	0.9900
C(113)-C(118)	1.369(16)
C(113)-C(114)	1.394(16)
C(114)-C(115)	1.379(17)
C(114)-H(114)	0.9500
C(115)-C(116)	1.395(18)
C(115)-H(115)	0.9500
C(116)-C(117)	1.352(18)
C(116)-H(116)	0.9500
C(117)-C(118)	1.419(19)
C(117)-H(117)	0.9500
C(118)-H(118)	0.9500
C(119)-C(120)	1.385(13)
C(119)-C(124)	1.392(16)
C(120)-C(121)	1.398(18)
C(120)-H(120)	0.9500
C(121)-C(122)	1.339(19)
C(121)-H(121)	0.9500
C(122)-C(123)	1.416(19)
C(122)-H(122)	0.9500
C(123)-C(124)	1.382(16)
C(123)-H(123)	0.9500
C(124)-H(124)	0.9500
N(101)-Pt(11)	2.088(9)
N(102)-Pt(11)	2.060(9)
N(102)-H(12C)	0.901(18)
Cl(11)-Pt(11)	2.309(2)
Cl(12)-Pt(11)	2.319(3)
C(201)-C(219)	1.476(13)
C(201)-C(202)	1.541(14)
C(201)-N(201)	1.549(12)
C(201)-H(201)	1.0000
C(202)-C(203)	1.547(14)

C(202)-H(20A)	0.9900
C(202)-H(20B)	0.9900
C(203)-C(204)	1.491(14)
C(203)-N(201)	1.545(11)
C(203)-H(203)	1.0000
C(204)-N(202)	1.484(12)
C(204)-H(20C)	0.9900
C(204)-H(20D)	0.9900
C(205)-C(206)	1.493(15)
C(205)-N(201)	1.508(12)
C(205)-H(20E)	0.9900
C(205)-H(20F)	0.9900
C(206)-C(211)	1.372(14)
C(206)-C(207)	1.419(16)
C(207)-C(208)	1.388(15)
C(207)-H(207)	0.9500
C(208)-C(209)	1.383(16)
C(208)-H(208)	0.9500
C(209)-C(210)	1.403(17)
C(209)-H(209)	0.9500
C(210)-C(211)	1.409(16)
C(210)-H(210)	0.9500
C(211)-H(211)	0.9500
C(212)-C(213)	1.480(14)
C(212)-N(202)	1.526(13)
C(212)-H(21A)	0.9900
C(212)-H(21B)	0.9900
C(213)-C(218)	1.383(14)
C(213)-C(214)	1.406(16)
C(214)-C(215)	1.377(15)
C(214)-H(214)	0.9500
C(215)-C(216)	1.420(19)
C(215)-H(215)	0.9500
C(216)-C(217)	1.36(2)
C(216)-H(216)	0.9500
C(217)-C(218)	1.397(18)
C(217)-H(217)	0.9500
C(218)-H(218)	0.9500
C(219)-C(220)	1.391(14)
C(219)-C(224)	1.413(13)
C(220)-C(221)	1.389(15)
C(220)-H(220)	0.9500
C(221)-C(222)	1.396(16)
C(221)-H(221)	0.9500
C(222)-C(223)	1.375(17)
C(222)-H(222)	0.9500
C(223)-C(224)	1.393(15)
C(223)-H(223)	0.9500
C(224)-H(224)	0.9500
N(201)-Pt(21)	2.075(8)
N(202)-Pt(21)	2.047(9)
N(202)-H(22C)	0.91(2)
Cl(21)-Pt(21)	2.308(2)
Cl(22)-Pt(21)	2.317(2)
C(301)-C(319)	1.508(15)
C(301)-C(302)	1.532(12)
C(301)-N(301)	1.554(12)

C(301)-H(301)	1.0000
C(302)-C(303)	1.556(14)
C(302)-H(30A)	0.9900
C(302)-H(30B)	0.9900
C(303)-C(304)	1.476(13)
C(303)-N(301)	1.552(12)
C(303)-H(303)	1.0000
C(304)-N(302)	1.508(14)
C(304)-H(30C)	0.9900
C(304)-H(30D)	0.9900
C(305)-N(301)	1.508(13)
C(305)-C(306)	1.512(15)
C(305)-H(30E)	0.9900
C(305)-H(30F)	0.9900
C(306)-C(307)	1.377(16)
C(306)-C(311)	1.387(16)
C(307)-C(308)	1.352(18)
C(307)-H(307)	0.9500
C(308)-C(309)	1.448(19)
C(308)-H(308)	0.9500
C(309)-C(310)	1.37(2)
C(309)-H(309)	0.9500
C(310)-C(311)	1.426(18)
C(310)-H(310)	0.9500
C(311)-H(311)	0.9500
C(312)-C(313)	1.489(15)
C(312)-N(302)	1.496(13)
C(312)-H(31A)	0.9900
C(312)-H(31B)	0.9900
C(313)-C(318)	1.376(15)
C(313)-C(314)	1.404(15)
C(314)-C(315)	1.401(17)
C(314)-H(314)	0.9500
C(315)-C(316)	1.357(17)
C(315)-H(315)	0.9500
C(316)-C(317)	1.376(17)
C(316)-H(316)	0.9500
C(317)-C(318)	1.386(17)
C(317)-H(317)	0.9500
C(318)-H(318)	0.9500
C(319)-C(324)	1.393(16)
C(319)-C(320)	1.411(15)
C(320)-C(321)	1.369(16)
C(320)-H(320)	0.9500
C(321)-C(322)	1.417(19)
C(321)-H(321)	0.9500
C(322)-C(323)	1.356(18)
C(322)-H(322)	0.9500
C(323)-C(324)	1.395(16)
C(323)-H(323)	0.9500
C(324)-H(324)	0.9500
N(301)-Pt(31)	2.087(10)
N(302)-Pt(31)	2.061(9)
N(302)-H(32C)	0.91(2)
Cl(31)-Pt(31)	2.317(2)
Cl(32)-Pt(31)	2.327(3)
C(401)-N(401)	1.130(16)

C(401)-C(402)	1.420(17)
C(402)-H(40A)	0.9800
C(402)-H(40B)	0.9800
C(402)-H(40C)	0.9800
C(501)-N(501)	1.140(14)
C(501)-C(502)	1.450(16)
C(502)-H(50A)	0.9800
C(502)-H(50B)	0.9800
C(502)-H(50C)	0.9800
C(601)-N(601)	1.154(16)
C(601)-C(602)	1.470(16)
C(602)-H(60A)	0.9800
C(602)-H(60B)	0.9800
C(602)-H(60C)	0.9800
C(701)-N(701)	1.121(13)
C(701)-C(702)	1.455(14)
C(702)-H(70A)	0.9800
C(702)-H(70B)	0.9800
C(702)-H(70C)	0.9800
C(19)-C(1)-C(2)	124.6(9)
C(19)-C(1)-N(1)	120.3(8)
C(2)-C(1)-N(1)	88.9(7)
C(19)-C(1)-H(1)	107.0
C(2)-C(1)-H(1)	107.0
N(1)-C(1)-H(1)	107.0
C(1)-C(2)-C(3)	87.1(8)
C(1)-C(2)-H(2A)	114.1
C(3)-C(2)-H(2A)	114.1
C(1)-C(2)-H(2B)	114.1
C(3)-C(2)-H(2B)	114.1
H(2A)-C(2)-H(2B)	111.3
C(4)-C(3)-N(1)	112.9(8)
C(4)-C(3)-C(2)	121.2(8)
N(1)-C(3)-C(2)	89.3(8)
C(4)-C(3)-H(3)	110.5
N(1)-C(3)-H(3)	110.5
C(2)-C(3)-H(3)	110.5
N(2)-C(4)-C(3)	108.4(8)
N(2)-C(4)-H(4A)	110.0
C(3)-C(4)-H(4A)	110.0
N(2)-C(4)-H(4B)	110.0
C(3)-C(4)-H(4B)	110.0
H(4A)-C(4)-H(4B)	108.4
N(1)-C(5)-C(6)	116.3(8)
N(1)-C(5)-H(5A)	108.2
C(6)-C(5)-H(5A)	108.2
N(1)-C(5)-H(5B)	108.2
C(6)-C(5)-H(5B)	108.2
H(5A)-C(5)-H(5B)	107.4
C(11)-C(6)-C(7)	120.9(11)
C(11)-C(6)-C(5)	120.0(10)
C(7)-C(6)-C(5)	118.8(10)
C(8)-C(7)-C(6)	118.6(12)
C(8)-C(7)-H(7)	120.7
C(6)-C(7)-H(7)	120.7
C(7)-C(8)-C(9)	122.6(12)
C(7)-C(8)-H(8)	118.7

C(9)-C(8)-H(8)	118.7
C(10)-C(9)-C(8)	117.3(12)
C(10)-C(9)-H(9)	121.3
C(8)-C(9)-H(9)	121.3
C(9)-C(10)-C(11)	120.6(12)
C(9)-C(10)-H(10)	119.7
C(11)-C(10)-H(10)	119.7
C(6)-C(11)-C(10)	119.8(11)
C(6)-C(11)-H(11)	120.1
C(10)-C(11)-H(11)	120.1
C(13)-C(12)-N(2)	114.4(8)
C(13)-C(12)-H(12A)	108.7
N(2)-C(12)-H(12A)	108.7
C(13)-C(12)-H(12B)	108.7
N(2)-C(12)-H(12B)	108.7
H(12A)-C(12)-H(12B)	107.6
C(18)-C(13)-C(14)	117.0(11)
C(18)-C(13)-C(12)	119.8(10)
C(14)-C(13)-C(12)	123.2(9)
C(15)-C(14)-C(13)	121.8(11)
C(15)-C(14)-H(14)	119.1
C(13)-C(14)-H(14)	119.1
C(16)-C(15)-C(14)	121.7(12)
C(16)-C(15)-H(15)	119.2
C(14)-C(15)-H(15)	119.2
C(15)-C(16)-C(17)	116.8(12)
C(15)-C(16)-H(16)	121.6
C(17)-C(16)-H(16)	121.6
C(16)-C(17)-C(18)	120.9(12)
C(16)-C(17)-H(17)	119.6
C(18)-C(17)-H(17)	119.6
C(13)-C(18)-C(17)	121.6(12)
C(13)-C(18)-H(18)	119.2
C(17)-C(18)-H(18)	119.2
C(24)-C(19)-C(20)	118.1(10)
C(24)-C(19)-C(1)	122.8(9)
C(20)-C(19)-C(1)	119.0(9)
C(21)-C(20)-C(19)	121.3(10)
C(21)-C(20)-H(20)	119.4
C(19)-C(20)-H(20)	119.4
C(22)-C(21)-C(20)	119.4(10)
C(22)-C(21)-H(21)	120.3
C(20)-C(21)-H(21)	120.3
C(23)-C(22)-C(21)	120.3(12)
C(23)-C(22)-H(22)	119.8
C(21)-C(22)-H(22)	119.8
C(22)-C(23)-C(24)	120.5(12)
C(22)-C(23)-H(23)	119.8
C(24)-C(23)-H(23)	119.8
C(19)-C(24)-C(23)	120.4(11)
C(19)-C(24)-H(24)	119.8
C(23)-C(24)-H(24)	119.8
C(5)-N(1)-C(3)	117.3(8)
C(5)-N(1)-C(1)	112.4(7)
C(3)-N(1)-C(1)	86.1(7)
C(5)-N(1)-Pt(1)	115.7(6)
C(3)-N(1)-Pt(1)	106.4(5)

C(1)-N(1)-Pt(1)	115.4(6)
C(4)-N(2)-C(12)	110.6(8)
C(4)-N(2)-Pt(1)	109.3(6)
C(12)-N(2)-Pt(1)	119.8(6)
C(4)-N(2)-H(2C)	118(6)
C(12)-N(2)-H(2C)	94(6)
Pt(1)-N(2)-H(2C)	105(3)
N(2)-Pt(1)-N(1)	85.4(3)
N(2)-Pt(1)-Cl(1)	178.1(2)
N(1)-Pt(1)-Cl(1)	93.3(2)
N(2)-Pt(1)-Cl(2)	92.8(2)
N(1)-Pt(1)-Cl(2)	177.5(3)
Cl(1)-Pt(1)-Cl(2)	88.56(9)
C(119)-C(101)-C(102)	123.6(10)
C(119)-C(101)-N(101)	123.9(8)
C(102)-C(101)-N(101)	89.4(7)
C(119)-C(101)-H(101)	105.9
C(102)-C(101)-H(101)	105.9
N(101)-C(101)-H(101)	105.9
C(101)-C(102)-C(103)	87.2(8)
C(101)-C(102)-H(10A)	114.1
C(103)-C(102)-H(10A)	114.1
C(101)-C(102)-H(10B)	114.1
C(103)-C(102)-H(10B)	114.1
H(10A)-C(102)-H(10B)	111.3
C(104)-C(103)-N(101)	113.1(8)
C(104)-C(103)-C(102)	122.2(9)
N(101)-C(103)-C(102)	90.0(7)
C(104)-C(103)-H(103)	110.0
N(101)-C(103)-H(103)	110.0
C(102)-C(103)-H(103)	110.0
N(102)-C(104)-C(103)	109.2(9)
N(102)-C(104)-H(10C)	109.8
C(103)-C(104)-H(10C)	109.8
N(102)-C(104)-H(10D)	109.8
C(103)-C(104)-H(10D)	109.8
H(10C)-C(104)-H(10D)	108.3
N(101)-C(105)-C(106)	115.1(8)
N(101)-C(105)-H(10E)	108.5
C(106)-C(105)-H(10E)	108.5
N(101)-C(105)-H(10F)	108.5
C(106)-C(105)-H(10F)	108.5
H(10E)-C(105)-H(10F)	107.5
C(107)-C(106)-C(111)	118.5(11)
C(107)-C(106)-C(105)	121.1(10)
C(111)-C(106)-C(105)	120.3(10)
C(108)-C(107)-C(106)	121.5(12)
C(108)-C(107)-H(107)	119.2
C(106)-C(107)-H(107)	119.2
C(107)-C(108)-C(109)	118.2(12)
C(107)-C(108)-H(108)	120.9
C(109)-C(108)-H(108)	120.9
C(110)-C(109)-C(108)	122.8(13)
C(110)-C(109)-H(109)	118.6
C(108)-C(109)-H(109)	118.6
C(109)-C(110)-C(111)	119.1(14)
C(109)-C(110)-H(110)	120.4

C(111)-C(110)-H(110)	120.4
C(110)-C(111)-C(106)	119.6(12)
C(110)-C(111)-H(111)	120.2
C(106)-C(111)-H(111)	120.2
C(113)-C(112)-N(102)	113.8(8)
C(113)-C(112)-H(11A)	108.8
N(102)-C(112)-H(11A)	108.8
C(113)-C(112)-H(11B)	108.8
N(102)-C(112)-H(11B)	108.8
H(11A)-C(112)-H(11B)	107.7
C(118)-C(113)-C(114)	120.4(12)
C(118)-C(113)-C(112)	119.3(11)
C(114)-C(113)-C(112)	120.2(10)
C(115)-C(114)-C(113)	118.9(12)
C(115)-C(114)-H(114)	120.5
C(113)-C(114)-H(114)	120.5
C(114)-C(115)-C(116)	120.5(12)
C(114)-C(115)-H(115)	119.8
C(116)-C(115)-H(115)	119.8
C(117)-C(116)-C(115)	121.2(13)
C(117)-C(116)-H(116)	119.4
C(115)-C(116)-H(116)	119.4
C(116)-C(117)-C(118)	118.6(12)
C(116)-C(117)-H(117)	120.7
C(118)-C(117)-H(117)	120.7
C(113)-C(118)-C(117)	120.5(12)
C(113)-C(118)-H(118)	119.8
C(117)-C(118)-H(118)	119.8
C(120)-C(119)-C(124)	117.7(11)
C(120)-C(119)-C(101)	119.9(11)
C(124)-C(119)-C(101)	122.1(9)
C(119)-C(120)-C(121)	121.5(12)
C(119)-C(120)-H(120)	119.2
C(121)-C(120)-H(120)	119.2
C(122)-C(121)-C(120)	120.1(12)
C(122)-C(121)-H(121)	119.9
C(120)-C(121)-H(121)	119.9
C(121)-C(122)-C(123)	120.2(12)
C(121)-C(122)-H(122)	119.9
C(123)-C(122)-H(122)	119.9
C(124)-C(123)-C(122)	119.1(12)
C(124)-C(123)-H(123)	120.4
C(122)-C(123)-H(123)	120.4
C(123)-C(124)-C(119)	121.2(11)
C(123)-C(124)-H(124)	119.4
C(119)-C(124)-H(124)	119.4
C(105)-N(101)-C(103)	117.9(8)
C(105)-N(101)-C(101)	112.6(8)
C(103)-N(101)-C(101)	86.6(7)
C(105)-N(101)-Pt(11)	114.5(6)
C(103)-N(101)-Pt(11)	106.3(6)
C(101)-N(101)-Pt(11)	116.0(7)
C(104)-N(102)-C(112)	110.9(9)
C(104)-N(102)-Pt(11)	108.6(7)
C(112)-N(102)-Pt(11)	121.2(7)
C(104)-N(102)-H(12C)	108(3)
C(112)-N(102)-H(12C)	103(3)

Pt(11)-N(102)-H(12C)	104(3)
N(102)-Pt(11)-N(101)	85.3(3)
N(102)-Pt(11)-Cl(11)	178.5(3)
N(101)-Pt(11)-Cl(11)	93.8(2)
N(102)-Pt(11)-Cl(12)	92.5(3)
N(101)-Pt(11)-Cl(12)	176.7(2)
Cl(11)-Pt(11)-Cl(12)	88.56(9)
C(219)-C(201)-C(202)	125.9(8)
C(219)-C(201)-N(201)	119.5(8)
C(202)-C(201)-N(201)	88.6(7)
C(219)-C(201)-H(201)	106.9
C(202)-C(201)-H(201)	106.9
N(201)-C(201)-H(201)	106.9
C(201)-C(202)-C(203)	87.2(7)
C(201)-C(202)-H(20A)	114.1
C(203)-C(202)-H(20A)	114.1
C(201)-C(202)-H(20B)	114.1
C(203)-C(202)-H(20B)	114.1
H(20A)-C(202)-H(20B)	111.3
C(204)-C(203)-N(201)	112.8(8)
C(204)-C(203)-C(202)	121.0(8)
N(201)-C(203)-C(202)	88.5(7)
C(204)-C(203)-H(203)	110.9
N(201)-C(203)-H(203)	110.9
C(202)-C(203)-H(203)	110.9
N(202)-C(204)-C(203)	109.8(8)
N(202)-C(204)-H(20C)	109.7
C(203)-C(204)-H(20C)	109.7
N(202)-C(204)-H(20D)	109.7
C(203)-C(204)-H(20D)	109.7
H(20C)-C(204)-H(20D)	108.2
C(206)-C(205)-N(201)	116.4(9)
C(206)-C(205)-H(20E)	108.2
N(201)-C(205)-H(20E)	108.2
C(206)-C(205)-H(20F)	108.2
N(201)-C(205)-H(20F)	108.2
H(20E)-C(205)-H(20F)	107.4
C(211)-C(206)-C(207)	118.9(11)
C(211)-C(206)-C(205)	120.3(11)
C(207)-C(206)-C(205)	120.5(10)
C(208)-C(207)-C(206)	119.5(10)
C(208)-C(207)-H(207)	120.2
C(206)-C(207)-H(207)	120.2
C(209)-C(208)-C(207)	121.1(11)
C(209)-C(208)-H(208)	119.4
C(207)-C(208)-H(208)	119.4
C(208)-C(209)-C(210)	120.2(10)
C(208)-C(209)-H(209)	119.9
C(210)-C(209)-H(209)	119.9
C(209)-C(210)-C(211)	118.2(10)
C(209)-C(210)-H(210)	120.9
C(211)-C(210)-H(210)	120.9
C(206)-C(211)-C(210)	122.1(11)
C(206)-C(211)-H(211)	119.0
C(210)-C(211)-H(211)	119.0
C(213)-C(212)-N(202)	115.5(8)
C(213)-C(212)-H(21A)	108.4

N(202)-C(212)-H(21A)	108.4
C(213)-C(212)-H(21B)	108.4
N(202)-C(212)-H(21B)	108.4
H(21A)-C(212)-H(21B)	107.5
C(218)-C(213)-C(214)	118.5(11)
C(218)-C(213)-C(212)	120.8(10)
C(214)-C(213)-C(212)	120.6(9)
C(215)-C(214)-C(213)	120.8(11)
C(215)-C(214)-H(214)	119.6
C(213)-C(214)-H(214)	119.6
C(214)-C(215)-C(216)	118.8(13)
C(214)-C(215)-H(215)	120.6
C(216)-C(215)-H(215)	120.6
C(217)-C(216)-C(215)	121.0(13)
C(217)-C(216)-H(216)	119.5
C(215)-C(216)-H(216)	119.5
C(216)-C(217)-C(218)	119.3(13)
C(216)-C(217)-H(217)	120.4
C(218)-C(217)-H(217)	120.4
C(213)-C(218)-C(217)	121.5(13)
C(213)-C(218)-H(218)	119.3
C(217)-C(218)-H(218)	119.3
C(220)-C(219)-C(224)	116.8(9)
C(220)-C(219)-C(201)	121.3(9)
C(224)-C(219)-C(201)	121.6(9)
C(221)-C(220)-C(219)	124.2(10)
C(221)-C(220)-H(220)	117.9
C(219)-C(220)-H(220)	117.9
C(220)-C(221)-C(222)	117.0(11)
C(220)-C(221)-H(221)	121.5
C(222)-C(221)-H(221)	121.5
C(223)-C(222)-C(221)	121.0(11)
C(223)-C(222)-H(222)	119.5
C(221)-C(222)-H(222)	119.5
C(222)-C(223)-C(224)	121.0(11)
C(222)-C(223)-H(223)	119.5
C(224)-C(223)-H(223)	119.5
C(223)-C(224)-C(219)	119.9(10)
C(223)-C(224)-H(224)	120.1
C(219)-C(224)-H(224)	120.1
C(205)-N(201)-C(203)	115.7(7)
C(205)-N(201)-C(201)	111.4(7)
C(203)-N(201)-C(201)	86.9(7)
C(205)-N(201)-Pt(21)	115.5(6)
C(203)-N(201)-Pt(21)	107.1(6)
C(201)-N(201)-Pt(21)	117.0(5)
C(204)-N(202)-C(212)	110.7(8)
C(204)-N(202)-Pt(21)	110.0(6)
C(212)-N(202)-Pt(21)	120.5(7)
C(204)-N(202)-H(22C)	106(6)
C(212)-N(202)-H(22C)	99(6)
Pt(21)-N(202)-H(22C)	110(3)
N(202)-Pt(21)-N(201)	85.3(3)
N(202)-Pt(21)-Cl(21)	178.9(3)
N(201)-Pt(21)-Cl(21)	93.6(2)
N(202)-Pt(21)-Cl(22)	92.7(2)
N(201)-Pt(21)-Cl(22)	176.7(2)

Cl(21)-Pt(21)-Cl(22)	88.37(9)
C(319)-C(301)-C(302)	123.2(10)
C(319)-C(301)-N(301)	119.8(9)
C(302)-C(301)-N(301)	89.8(7)
C(319)-C(301)-H(301)	107.4
C(302)-C(301)-H(301)	107.4
N(301)-C(301)-H(301)	107.4
C(301)-C(302)-C(303)	86.7(7)
C(301)-C(302)-H(30A)	114.2
C(303)-C(302)-H(30A)	114.2
C(301)-C(302)-H(30B)	114.2
C(303)-C(302)-H(30B)	114.2
H(30A)-C(302)-H(30B)	111.4
C(304)-C(303)-N(301)	113.1(8)
C(304)-C(303)-C(302)	120.7(9)
N(301)-C(303)-C(302)	89.0(7)
C(304)-C(303)-H(303)	110.7
N(301)-C(303)-H(303)	110.7
C(302)-C(303)-H(303)	110.7
C(303)-C(304)-N(302)	110.2(8)
C(303)-C(304)-H(30C)	109.6
N(302)-C(304)-H(30C)	109.6
C(303)-C(304)-H(30D)	109.6
N(302)-C(304)-H(30D)	109.6
H(30C)-C(304)-H(30D)	108.1
N(301)-C(305)-C(306)	115.3(8)
N(301)-C(305)-H(30E)	108.5
C(306)-C(305)-H(30E)	108.5
N(301)-C(305)-H(30F)	108.5
C(306)-C(305)-H(30F)	108.5
H(30E)-C(305)-H(30F)	107.5
C(307)-C(306)-C(311)	120.4(11)
C(307)-C(306)-C(305)	121.2(10)
C(311)-C(306)-C(305)	118.3(11)
C(308)-C(307)-C(306)	121.9(12)
C(308)-C(307)-H(307)	119.0
C(306)-C(307)-H(307)	119.0
C(307)-C(308)-C(309)	119.7(14)
C(307)-C(308)-H(308)	120.2
C(309)-C(308)-H(308)	120.2
C(310)-C(309)-C(308)	118.2(13)
C(310)-C(309)-H(309)	120.9
C(308)-C(309)-H(309)	120.9
C(309)-C(310)-C(311)	121.1(13)
C(309)-C(310)-H(310)	119.4
C(311)-C(310)-H(310)	119.4
C(306)-C(311)-C(310)	118.5(13)
C(306)-C(311)-H(311)	120.7
C(310)-C(311)-H(311)	120.7
C(313)-C(312)-N(302)	115.1(8)
C(313)-C(312)-H(31A)	108.5
N(302)-C(312)-H(31A)	108.5
C(313)-C(312)-H(31B)	108.5
N(302)-C(312)-H(31B)	108.5
H(31A)-C(312)-H(31B)	107.5
C(318)-C(313)-C(314)	115.5(11)
C(318)-C(313)-C(312)	121.3(9)

C(314)-C(313)-C(312)	123.2(10)
C(315)-C(314)-C(313)	122.1(11)
C(315)-C(314)-H(314)	118.9
C(313)-C(314)-H(314)	118.9
C(316)-C(315)-C(314)	120.5(12)
C(316)-C(315)-H(315)	119.8
C(314)-C(315)-H(315)	119.8
C(315)-C(316)-C(317)	118.3(12)
C(315)-C(316)-H(316)	120.8
C(317)-C(316)-H(316)	120.8
C(316)-C(317)-C(318)	121.4(12)
C(316)-C(317)-H(317)	119.3
C(318)-C(317)-H(317)	119.3
C(313)-C(318)-C(317)	122.2(11)
C(313)-C(318)-H(318)	118.9
C(317)-C(318)-H(318)	118.9
C(324)-C(319)-C(320)	118.6(11)
C(324)-C(319)-C(301)	120.5(10)
C(320)-C(319)-C(301)	119.6(10)
C(321)-C(320)-C(319)	120.4(12)
C(321)-C(320)-H(320)	119.8
C(319)-C(320)-H(320)	119.8
C(320)-C(321)-C(322)	120.0(11)
C(320)-C(321)-H(321)	120.0
C(322)-C(321)-H(321)	120.0
C(323)-C(322)-C(321)	119.6(12)
C(323)-C(322)-H(322)	120.2
C(321)-C(322)-H(322)	120.2
C(322)-C(323)-C(324)	120.9(13)
C(322)-C(323)-H(323)	119.5
C(324)-C(323)-H(323)	119.5
C(319)-C(324)-C(323)	120.2(11)
C(319)-C(324)-H(324)	119.9
C(323)-C(324)-H(324)	119.9
C(305)-N(301)-C(303)	116.2(8)
C(305)-N(301)-C(301)	112.2(8)
C(303)-N(301)-C(301)	86.0(7)
C(305)-N(301)-Pt(31)	115.0(6)
C(303)-N(301)-Pt(31)	106.8(6)
C(301)-N(301)-Pt(31)	117.4(7)
C(312)-N(302)-C(304)	110.5(8)
C(312)-N(302)-Pt(31)	122.6(7)
C(304)-N(302)-Pt(31)	108.4(6)
C(312)-N(302)-H(32C)	109(6)
C(304)-N(302)-H(32C)	98(7)
Pt(31)-N(302)-H(32C)	105(3)
N(302)-Pt(31)-N(301)	85.6(3)
N(302)-Pt(31)-Cl(31)	178.3(3)
N(301)-Pt(31)-Cl(31)	93.2(2)
N(302)-Pt(31)-Cl(32)	92.6(3)
N(301)-Pt(31)-Cl(32)	176.6(2)
Cl(31)-Pt(31)-Cl(32)	88.62(9)
N(401)-C(401)-C(402)	179.0(13)
C(401)-C(402)-H(40A)	109.5
C(401)-C(402)-H(40B)	109.5
H(40A)-C(402)-H(40B)	109.5
C(401)-C(402)-H(40C)	109.5

H(40A)-C(402)-H(40C)	109.5
H(40B)-C(402)-H(40C)	109.5
N(501)-C(501)-C(502)	178.0(14)
C(501)-C(502)-H(50A)	109.5
C(501)-C(502)-H(50B)	109.5
H(50A)-C(502)-H(50B)	109.5
C(501)-C(502)-H(50C)	109.5
H(50A)-C(502)-H(50C)	109.5
H(50B)-C(502)-H(50C)	109.5
N(601)-C(601)-C(602)	178.1(13)
C(601)-C(602)-H(60A)	109.5
C(601)-C(602)-H(60B)	109.5
H(60A)-C(602)-H(60B)	109.5
C(601)-C(602)-H(60C)	109.5
H(60A)-C(602)-H(60C)	109.5
H(60B)-C(602)-H(60C)	109.5
N(701)-C(701)-C(702)	177.4(12)
C(701)-C(702)-H(70A)	109.5
C(701)-C(702)-H(70B)	109.5
H(70A)-C(702)-H(70B)	109.5
C(701)-C(702)-H(70C)	109.5
H(70A)-C(702)-H(70C)	109.5
H(70B)-C(702)-H(70C)	109.5

Symmetry transformations used to generate equivalent atoms:

Table 4. Anisotropic displacement parameters ($Å^2$x 10^3) for ***(rac)*-7**. The anisotropic displacement factor exponent takes the form: -2 ^{2}[$h^2a^{*2}U^{11}$ + ... + 2 h k a* b* U^{12}]

	U^{11}	U^{22}	U^{33}	U^{23}	U^{13}	U^{12}
C(1)	24(4)	28(4)	21(4)	-6(3)	9(4)	-4(4)
C(2)	23(4)	29(4)	29(5)	-5(3)	7(4)	0(4)
C(3)	20(4)	20(4)	27(4)	0(3)	6(4)	-1(4)
C(4)	20(5)	21(5)	23(6)	-1(5)	-1(4)	0(5)
C(5)	23(5)	37(6)	10(5)	-5(4)	8(4)	-8(5)
C(6)	18(5)	21(5)	31(6)	1(5)	-4(5)	-8(5)
C(7)	36(7)	29(6)	47(8)	7(6)	-8(6)	2(5)
C(8)	39(7)	50(8)	44(8)	13(7)	-20(6)	-4(7)
C(9)	55(8)	57(9)	29(7)	-1(6)	-6(7)	-19(7)
C(10)	43(7)	41(7)	40(8)	-9(6)	-7(6)	-4(6)
C(11)	26(6)	28(6)	29(6)	-4(5)	0(5)	-14(5)
C(12)	35(6)	13(5)	32(6)	0(5)	11(5)	-2(5)
C(13)	29(6)	32(5)	28(6)	10(5)	6(4)	10(5)
C(14)	67(7)	22(5)	22(5)	9(5)	-5(5)	4(6)
C(15)	78(9)	48(7)	24(6)	-2(5)	11(6)	0(6)
C(16)	51(6)	68(6)	23(5)	-12(5)	3(4)	5(6)
C(17)	71(9)	82(7)	26(7)	-3(6)	-18(6)	35(8)
C(18)	69(8)	43(6)	23(6)	9(5)	2(5)	24(6)
C(19)	21(5)	21(6)	20(5)	-2(4)	8(4)	10(4)
C(20)	16(5)	23(5)	26(6)	-8(5)	12(4)	4(4)
C(21)	33(6)	25(6)	25(6)	-4(5)	3(5)	1(5)
C(22)	32(5)	46(6)	20(5)	6(5)	15(5)	1(5)
C(23)	17(5)	56(8)	29(7)	-3(6)	-4(5)	-5(5)
C(24)	23(6)	43(7)	23(6)	-5(5)	16(5)	-2(5)
N(1)	26(4)	22(4)	23(4)	1(3)	2(3)	1(4)

N(2)	16(4)	29(5)	21(5)	-5(4)	-1(4)	-4(4)
Cl(1)	23(1)	21(1)	23(1)	3(1)	4(1)	5(1)
Cl(2)	17(1)	24(1)	30(1)	-5(1)	9(1)	1(1)
Pt(1)	15(1)	17(1)	21(1)	-1(1)	2(1)	0(1)
C(101)	20(4)	24(4)	23(4)	0(4)	-4(3)	5(4)
C(102)	18(3)	26(4)	25(5)	2(4)	-4(3)	2(4)
C(103)	18(4)	20(4)	23(4)	4(4)	-3(3)	6(4)
C(104)	21(5)	29(6)	22(6)	-5(5)	4(5)	2(5)
C(105)	16(5)	20(6)	37(7)	2(5)	16(5)	6(4)
C(106)	31(6)	30(6)	19(6)	8(5)	1(5)	-13(6)
C(107)	23(6)	40(7)	29(7)	-9(6)	6(5)	1(5)
C(108)	41(8)	44(8)	55(9)	-21(7)	17(7)	1(7)
C(109)	66(9)	57(9)	23(7)	-16(7)	7(7)	-18(8)
C(110)	49(8)	40(7)	35(8)	2(6)	-4(6)	-22(6)
C(111)	33(7)	28(6)	28(7)	12(5)	2(5)	-7(5)
C(112)	18(5)	23(6)	36(7)	11(5)	4(5)	-1(5)
C(113)	37(7)	37(7)	31(7)	3(6)	6(6)	2(6)
C(114)	21(6)	51(8)	25(6)	-4(6)	2(5)	7(6)
C(115)	38(7)	54(8)	46(9)	1(7)	-19(6)	-5(7)
C(116)	84(7)	43(8)	22(6)	-6(7)	-5(6)	-4(8)
C(117)	79(6)	50(8)	29(6)	-8(5)	12(5)	11(6)
C(118)	54(7)	60(10)	27(6)	-1(6)	13(5)	21(7)
C(119)	24(5)	20(6)	28(6)	12(6)	8(4)	8(5)
C(120)	41(6)	32(6)	22(5)	0(7)	6(5)	7(7)
C(121)	43(8)	44(8)	44(9)	9(7)	4(7)	-1(7)
C(122)	71(9)	36(8)	21(7)	4(6)	12(7)	14(7)
C(123)	76(10)	28(7)	23(7)	-2(6)	1(6)	-11(7)
C(124)	45(7)	29(7)	22(6)	-5(5)	-2(5)	-1(6)
N(101)	15(3)	22(4)	19(4)	-4(4)	1(3)	-1(3)
N(102)	27(5)	18(5)	22(5)	-11(4)	-2(4)	-10(4)
Cl(11)	21(1)	25(2)	28(2)	2(1)	9(1)	6(1)
Cl(12)	21(1)	22(1)	31(2)	5(1)	1(1)	2(1)
Pt(11)	16(1)	20(1)	20(1)	0(1)	2(1)	0(1)
C(201)	15(4)	24(4)	22(4)	-1(3)	-5(4)	-3(4)
C(202)	13(4)	20(4)	32(5)	-4(3)	-2(4)	1(4)
C(203)	18(4)	13(4)	30(4)	3(3)	4(4)	1(4)
C(204)	32(6)	14(5)	30(6)	8(5)	4(5)	2(5)
C(205)	26(6)	28(6)	23(6)	-6(5)	-8(5)	16(5)
C(206)	20(5)	32(6)	30(6)	5(6)	6(5)	2(5)
C(207)	31(6)	25(6)	23(6)	2(5)	-2(5)	4(5)
C(208)	56(8)	30(6)	26(6)	-11(5)	-4(6)	-2(6)
C(209)	52(8)	45(7)	20(6)	-10(6)	11(6)	0(6)
C(210)	43(7)	57(8)	24(6)	14(6)	18(6)	-8(7)
C(211)	25(6)	34(6)	30(6)	6(5)	6(5)	-7(5)
C(212)	19(5)	18(5)	35(6)	6(5)	-2(5)	7(5)
C(213)	11(5)	43(7)	24(6)	0(5)	-3(4)	-6(5)
C(214)	39(6)	32(7)	32(6)	-3(6)	5(5)	0(6)
C(215)	85(10)	42(7)	24(7)	-3(6)	13(7)	16(7)
C(216)	70(9)	88(8)	19(6)	-7(8)	0(6)	28(10)
C(217)	73(10)	100(9)	26(7)	19(7)	29(7)	13(9)
C(218)	41(6)	39(6)	29(6)	18(5)	3(5)	-2(6)
C(219)	15(5)	25(7)	18(5)	-1(4)	-2(4)	5(4)
C(220)	18(5)	41(7)	19(6)	-6(5)	-6(4)	-3(5)
C(221)	34(6)	29(6)	28(6)	-2(5)	-7(5)	-11(5)
C(222)	22(6)	64(8)	30(7)	3(6)	-2(5)	0(6)
C(223)	25(6)	58(8)	17(6)	0(6)	4(5)	9(6)
C(224)	29(6)	33(6)	25(6)	1(5)	-4(5)	1(5)

N(201)	23(4)	19(4)	17(4)	3(3)	2(3)	-2(3)
N(202)	13(4)	29(5)	22(5)	-4(4)	9(3)	9(4)
Cl(21)	21(1)	20(1)	26(1)	2(1)	0(1)	-4(1)
Cl(22)	15(1)	25(1)	30(1)	-3(1)	-2(1)	0(1)
Pt(21)	15(1)	17(1)	20(1)	-2(1)	1(1)	0(1)
C(301)	28(4)	16(4)	20(3)	-6(4)	4(3)	4(4)
C(302)	24(3)	25(4)	27(4)	0(5)	-2(3)	-2(5)
C(303)	21(4)	21(4)	22(4)	2(3)	2(3)	-5(4)
C(304)	17(4)	9(4)	27(4)	4(4)	4(3)	6(4)
C(305)	20(5)	24(6)	34(7)	-7(5)	-7(5)	6(5)
C(306)	11(5)	30(6)	25(6)	3(5)	3(4)	10(5)
C(307)	27(6)	48(9)	31(7)	14(6)	-4(5)	7(6)
C(308)	56(9)	39(7)	51(10)	-1(7)	-17(7)	9(7)
C(309)	69(10)	53(9)	26(7)	-5(7)	-3(7)	12(8)
C(310)	45(8)	56(9)	40(8)	15(7)	13(7)	14(7)
C(311)	40(7)	37(7)	37(8)	9(6)	5(6)	19(6)
C(312)	26(6)	31(7)	23(6)	-6(5)	15(5)	3(5)
C(313)	16(5)	29(6)	20(6)	-9(5)	5(4)	1(5)
C(314)	29(6)	45(7)	32(7)	14(7)	-1(5)	1(6)
C(315)	33(7)	52(8)	30(7)	-6(6)	-3(6)	9(6)
C(316)	53(6)	45(8)	38(8)	6(6)	-9(6)	15(6)
C(317)	65(7)	58(9)	19(6)	13(6)	4(5)	-7(7)
C(318)	33(6)	67(8)	21(6)	4(6)	9(5)	-4(6)
C(319)	36(6)	23(6)	13(5)	-2(5)	-7(5)	-2(5)
C(320)	36(5)	47(6)	15(5)	-7(5)	9(4)	-4(5)
C(321)	46(8)	41(7)	20(7)	-11(6)	7(6)	-19(6)
C(322)	81(10)	28(7)	34(8)	1(6)	31(8)	-21(7)
C(323)	64(9)	34(7)	25(7)	-1(6)	-7(6)	8(7)
C(324)	48(7)	33(7)	18(6)	-2(5)	0(5)	-8(6)
N(301)	23(3)	15(3)	22(3)	-2(4)	4(3)	-2(4)
N(302)	18(4)	30(6)	24(5)	-5(4)	-7(4)	6(4)
Cl(31)	20(1)	26(2)	26(1)	-4(1)	10(1)	-4(1)
Cl(32)	24(1)	22(1)	30(2)	-5(1)	1(1)	-2(1)
Pt(31)	16(1)	19(1)	20(1)	-1(1)	1(1)	0(1)
C(401)	30(7)	36(7)	28(7)	5(6)	2(5)	-9(6)
N(401)	60(7)	35(7)	66(9)	-1(7)	27(7)	-7(6)
C(402)	36(7)	41(7)	24(7)	17(6)	8(5)	1(6)
C(501)	39(7)	32(6)	13(5)	9(5)	-1(5)	-9(6)
C(502)	31(6)	30(6)	28(6)	6(5)	12(5)	4(5)
N(501)	30(6)	65(8)	58(8)	26(6)	-6(6)	-8(6)
C(601)	38(6)	21(6)	16(5)	-1(6)	10(4)	5(6)
C(602)	36(7)	27(6)	34(7)	2(6)	10(6)	-3(5)
N(601)	55(7)	36(6)	61(8)	-4(6)	37(6)	14(6)
C(701)	25(6)	23(6)	31(6)	3(5)	4(5)	1(5)
C(702)	29(6)	29(6)	28(6)	0(5)	8(5)	-2(5)
N(701)	20(5)	63(7)	59(8)	24(6)	7(5)	14(5)

Table 5. Hydrogen coordinates (x 10^4) and isotropic displacement parameters ($Å^2$x 10^3) for ***(rac)*-7**.

	x	y	z	U(eq)
H(1)	3500	1210	4372	29
H(2A)	5271	3058	4177	32
H(2B)	3827	3302	4262	32

H(3)	2997	2096	3849	27
H(4A)	4064	1998	3401	25
H(4B)	4266	3420	3533	25
H(5A)	2773	-134	4008	28
H(5B)	3956	-1064	4047	28
H(7)	1900	452	3501	45
H(8)	1384	-366	3021	55
H(9)	2540	-2027	2816	57
H(10)	4228	-2895	3120	51
H(11)	4721	-2105	3612	33
H(12A)	6317	3470	3248	31
H(12B)	7288	2325	3313	31
H(14)	6367	36	3148	45
H(15)	5534	-1020	2722	60
H(16)	4228	29	2357	57
H(17)	4089	2268	2393	72
H(18)	4966	3332	2820	54
H(20)	4358	-845	4601	25
H(21)	5637	-1646	5010	33
H(22)	7246	-385	5232	38
H(23)	7606	1625	5042	42
H(24)	6394	2399	4616	35
H(2C)	6320(70)	2910(40)	3699(16)	26
H(101)	8567	2180	614	27
H(10A)	6830	374	836	28
H(10B)	6585	1871	743	28
H(103)	7902	2614	1143	24
H(10C)	8213	1474	1591	28
H(10D)	6777	1247	1473	28
H(10E)	10010	2907	945	28
H(10F)	10929	1709	931	28
H(107)	9497	3725	1456	37
H(108)	10312	4193	1948	55
H(109)	12015	3032	2153	58
H(110)	13047	1597	1867	50
H(111)	12154	994	1386	36
H(11A)	6842	-878	1769	31
H(11B)	7968	-1836	1714	31
H(114)	10220	-935	1858	39
H(115)	11387	-28	2271	57
H(116)	10394	1137	2638	60
H(117)	8245	1353	2609	62
H(118)	7049	430	2189	56
H(120)	10563	1080	409	38
H(121)	11067	-106	-16	52
H(122)	9582	-1351	-271	51
H(123)	7534	-1480	-99	51
H(124)	7083	-416	347	39
H(12C)	7260(30)	-670(40)	1297(8)	27
H(201)	11140	6244	4393	25
H(20A)	10757	8352	4282	27
H(20B)	9271	8107	4188	27
H(203)	11393	7145	3871	24
H(20C)	9936	8469	3559	30
H(20D)	10139	7068	3422	30
H(20E)	11708	4933	4041	32
H(20F)	10555	3981	4065	32

H(207)	9697	2831	3623	32
H(208)	10035	2110	3133	45
H(209)	11522	3078	2847	46
H(210)	12604	4915	3037	49
H(211)	12278	5619	3536	35
H(21A)	6868	7293	3299	29
H(21B)	7807	8440	3241	29
H(214)	7705	5017	3167	41
H(215)	8421	3888	2756	60
H(216)	9462	5004	2378	71
H(217)	9676	7196	2401	78
H(218)	8907	8323	2809	43
H(220)	10426	4196	4626	32
H(221)	9335	3333	5019	37
H(222)	7825	4626	5237	47
H(223)	7379	6648	5048	40
H(224)	8374	7441	4630	35
H(22C)	8120(90)	7960(30)	3702(15)	25
H(301)	3574	4602	616	25
H(30A)	1571	4941	748	31
H(30B)	1844	6432	843	31
H(303)	2931	4160	1149	26
H(30C)	3203	5294	1595	21
H(30D)	1775	5523	1472	21
H(30E)	5952	5090	932	32
H(30F)	5034	3895	955	32
H(307)	7085	5901	1388	43
H(308)	7931	5510	1872	60
H(309)	7110	3794	2167	60
H(310)	5398	2644	1958	56
H(311)	4616	3004	1445	46
H(31A)	2883	8615	1720	31
H(31B)	1834	7574	1778	31
H(314)	5198	7955	1891	43
H(315)	6439	7081	2297	46
H(316)	5563	5750	2646	55
H(317)	3457	5195	2566	57
H(318)	2200	6109	2176	48
H(320)	5615	5737	417	39
H(321)	6080	6892	-11	43
H(322)	4522	8184	-268	56
H(323)	2554	8302	-88	50
H(324)	2069	7152	344	40
H(32C)	2230(30)	7330(100)	1293(12)	29
H(40A)	4072	12	357	50
H(40B)	3361	-584	633	50
H(40C)	4759	-52	692	50
H(50A)	6029	5606	4283	44
H(50B)	5489	5405	4610	44
H(50C)	6007	6776	4517	44
H(60A)	8209	7360	537	47
H(60B)	9450	6768	409	47
H(60C)	9413	7094	764	47
H(70A)	-1438	652	4293	43
H(70B)	-1226	1794	4534	43
H(70C)	-658	407	4612	43

Table 6. Torsion angles [°] for **(*rac*)-7**.

C(19)-C(1)-C(2)-C(3)	148.7(9)
N(1)-C(1)-C(2)-C(3)	21.7(7)
C(1)-C(2)-C(3)-C(4)	-138.8(9)
C(1)-C(2)-C(3)-N(1)	-22.2(7)
N(1)-C(3)-C(4)-N(2)	-46.9(11)
C(2)-C(3)-C(4)-N(2)	57.1(12)
N(1)-C(5)-C(6)-C(11)	97.3(11)
N(1)-C(5)-C(6)-C(7)	-89.4(12)
C(11)-C(6)-C(7)-C(8)	0.4(17)
C(5)-C(6)-C(7)-C(8)	-172.9(10)
C(6)-C(7)-C(8)-C(9)	-1.1(18)
C(7)-C(8)-C(9)-C(10)	0.9(18)
C(8)-C(9)-C(10)-C(11)	0.0(18)
C(7)-C(6)-C(11)-C(10)	0.5(16)
C(5)-C(6)-C(11)-C(10)	173.7(10)
C(9)-C(10)-C(11)-C(6)	-0.7(17)
N(2)-C(12)-C(13)-C(18)	112.0(11)
N(2)-C(12)-C(13)-C(14)	-71.3(13)
C(18)-C(13)-C(14)-C(15)	0.4(18)
C(12)-C(13)-C(14)-C(15)	-176.4(11)
C(13)-C(14)-C(15)-C(16)	-3(2)
C(14)-C(15)-C(16)-C(17)	5(2)
C(15)-C(16)-C(17)-C(18)	-5(2)
C(14)-C(13)-C(18)-C(17)	0.2(18)
C(12)-C(13)-C(18)-C(17)	177.1(12)
C(16)-C(17)-C(18)-C(13)	2(2)
C(2)-C(1)-C(19)-C(24)	-3.2(14)
N(1)-C(1)-C(19)-C(24)	109.0(10)
C(2)-C(1)-C(19)-C(20)	173.9(8)
N(1)-C(1)-C(19)-C(20)	-73.9(11)
C(24)-C(19)-C(20)-C(21)	-0.4(13)
C(1)-C(19)-C(20)-C(21)	-177.6(9)
C(19)-C(20)-C(21)-C(22)	1.5(14)
C(20)-C(21)-C(22)-C(23)	-0.8(15)
C(21)-C(22)-C(23)-C(24)	-0.9(15)
C(20)-C(19)-C(24)-C(23)	-1.4(13)
C(1)-C(19)-C(24)-C(23)	175.8(9)
C(22)-C(23)-C(24)-C(19)	2.0(15)
C(6)-C(5)-N(1)-C(3)	69.4(11)
C(6)-C(5)-N(1)-C(1)	166.8(9)
C(6)-C(5)-N(1)-Pt(1)	-57.6(11)
C(4)-C(3)-N(1)-C(5)	-101.3(10)
C(2)-C(3)-N(1)-C(5)	134.8(7)
C(4)-C(3)-N(1)-C(1)	145.5(8)
C(2)-C(3)-N(1)-C(1)	21.6(7)
C(4)-C(3)-N(1)-Pt(1)	30.1(9)
C(2)-C(3)-N(1)-Pt(1)	-93.8(6)
C(19)-C(1)-N(1)-C(5)	89.8(10)
C(2)-C(1)-N(1)-C(5)	-139.9(8)
C(19)-C(1)-N(1)-C(3)	-152.2(9)
C(2)-C(1)-N(1)-C(3)	-21.9(7)
C(19)-C(1)-N(1)-Pt(1)	-45.9(10)
C(2)-C(1)-N(1)-Pt(1)	84.4(7)
C(3)-C(4)-N(2)-C(12)	173.3(8)

C(3)-C(4)-N(2)-Pt(1)	39.4(9)
C(13)-C(12)-N(2)-C(4)	-53.4(11)
C(13)-C(12)-N(2)-Pt(1)	75.0(10)
C(4)-N(2)-Pt(1)-N(1)	-18.6(7)
C(12)-N(2)-Pt(1)-N(1)	-147.6(7)
C(4)-N(2)-Pt(1)-Cl(2)	163.0(6)
C(12)-N(2)-Pt(1)-Cl(2)	34.0(7)
C(5)-N(1)-Pt(1)-N(2)	126.4(7)
C(3)-N(1)-Pt(1)-N(2)	-5.9(6)
C(1)-N(1)-Pt(1)-N(2)	-99.4(7)
C(5)-N(1)-Pt(1)-Cl(1)	-54.9(7)
C(3)-N(1)-Pt(1)-Cl(1)	172.8(6)
C(1)-N(1)-Pt(1)-Cl(1)	79.3(6)
C(119)-C(101)-C(102)-C(103)	150.9(10)
N(101)-C(101)-C(102)-C(103)	19.5(8)
C(101)-C(102)-C(103)-C(104)	-137.3(10)
C(101)-C(102)-C(103)-N(101)	-19.7(8)
N(101)-C(103)-C(104)-N(102)	-46.8(12)
C(102)-C(103)-C(104)-N(102)	58.7(13)
N(101)-C(105)-C(106)-C(107)	-84.5(12)
N(101)-C(105)-C(106)-C(111)	99.8(11)
C(111)-C(106)-C(107)-C(108)	-0.7(17)
C(105)-C(106)-C(107)-C(108)	-176.4(11)
C(106)-C(107)-C(108)-C(109)	0.2(18)
C(107)-C(108)-C(109)-C(110)	3.2(19)
C(108)-C(109)-C(110)-C(111)	-5.8(19)
C(109)-C(110)-C(111)-C(106)	5.1(16)
C(107)-C(106)-C(111)-C(110)	-2.0(15)
C(105)-C(106)-C(111)-C(110)	173.8(10)
N(102)-C(112)-C(113)-C(118)	112.7(12)
N(102)-C(112)-C(113)-C(114)	-71.7(14)
C(118)-C(113)-C(114)-C(115)	-1.9(19)
C(112)-C(113)-C(114)-C(115)	-177.5(11)
C(113)-C(114)-C(115)-C(116)	0(2)
C(114)-C(115)-C(116)-C(117)	1(2)
C(115)-C(116)-C(117)-C(118)	-1(2)
C(114)-C(113)-C(118)-C(117)	2(2)
C(112)-C(113)-C(118)-C(117)	177.6(11)
C(116)-C(117)-C(118)-C(113)	0(2)
C(102)-C(101)-C(119)-C(120)	-179.0(11)
N(101)-C(101)-C(119)-C(120)	-63.5(15)
C(102)-C(101)-C(119)-C(124)	7.2(17)
N(101)-C(101)-C(119)-C(124)	122.7(11)
C(124)-C(119)-C(120)-C(121)	-1.3(18)
C(101)-C(119)-C(120)-C(121)	-175.4(11)
C(119)-C(120)-C(121)-C(122)	2.3(19)
C(120)-C(121)-C(122)-C(123)	-0.7(19)
C(121)-C(122)-C(123)-C(124)	-1.8(19)
C(122)-C(123)-C(124)-C(119)	2.8(18)
C(120)-C(119)-C(124)-C(123)	-1.2(17)
C(101)-C(119)-C(124)-C(123)	172.7(11)
C(106)-C(105)-N(101)-C(103)	65.5(12)
C(106)-C(105)-N(101)-C(101)	164.0(9)
C(106)-C(105)-N(101)-Pt(11)	-60.6(10)
C(104)-C(103)-N(101)-C(105)	-101.3(11)
C(102)-C(103)-N(101)-C(105)	133.3(9)
C(104)-C(103)-N(101)-C(101)	144.8(9)

C(102)-C(103)-N(101)-C(101)	19.5(8)
C(104)-C(103)-N(101)-Pt(11)	28.8(10)
C(102)-C(103)-N(101)-Pt(11)	-96.6(7)
C(119)-C(101)-N(101)-C(105)	90.4(12)
C(102)-C(101)-N(101)-C(105)	-138.4(8)
C(119)-C(101)-N(101)-C(103)	-150.8(11)
C(102)-C(101)-N(101)-C(103)	-19.5(8)
C(119)-C(101)-N(101)-Pt(11)	-44.3(12)
C(102)-C(101)-N(101)-Pt(11)	87.0(8)
C(103)-C(104)-N(102)-C(112)	175.8(8)
C(103)-C(104)-N(102)-Pt(11)	40.2(10)
C(113)-C(112)-N(102)-C(104)	-53.5(12)
C(113)-C(112)-N(102)-Pt(11)	75.6(10)
C(104)-N(102)-Pt(11)-N(101)	-19.6(8)
C(112)-N(102)-Pt(11)-N(101)	-149.7(7)
C(104)-N(102)-Pt(11)-Cl(12)	162.8(7)
C(112)-N(102)-Pt(11)-Cl(12)	32.7(7)
C(105)-N(101)-Pt(11)-N(102)	127.4(7)
C(103)-N(101)-Pt(11)-N(102)	-4.6(6)
C(101)-N(101)-Pt(11)-N(102)	-98.8(6)
C(105)-N(101)-Pt(11)-Cl(11)	-51.5(6)
C(103)-N(101)-Pt(11)-Cl(11)	176.6(6)
C(101)-N(101)-Pt(11)-Cl(11)	82.3(6)
C(219)-C(201)-C(202)-C(203)	-148.3(10)
N(201)-C(201)-C(202)-C(203)	-22.2(7)
C(201)-C(202)-C(203)-C(204)	138.1(9)
C(201)-C(202)-C(203)-N(201)	22.2(7)
N(201)-C(203)-C(204)-N(202)	43.3(11)
C(202)-C(203)-C(204)-N(202)	-59.4(12)
N(201)-C(205)-C(206)-C(211)	85.6(12)
N(201)-C(205)-C(206)-C(207)	-101.0(12)
C(211)-C(206)-C(207)-C(208)	-0.1(16)
C(205)-C(206)-C(207)-C(208)	-173.6(10)
C(206)-C(207)-C(208)-C(209)	1.0(17)
C(207)-C(208)-C(209)-C(210)	-2.3(19)
C(208)-C(209)-C(210)-C(211)	2.7(18)
C(207)-C(206)-C(211)-C(210)	0.6(17)
C(205)-C(206)-C(211)-C(210)	174.2(11)
C(209)-C(210)-C(211)-C(206)	-1.9(18)
N(202)-C(212)-C(213)-C(218)	-113.7(11)
N(202)-C(212)-C(213)-C(214)	69.5(13)
C(218)-C(213)-C(214)-C(215)	1.2(17)
C(212)-C(213)-C(214)-C(215)	178.1(11)
C(213)-C(214)-C(215)-C(216)	0.6(19)
C(214)-C(215)-C(216)-C(217)	-2(2)
C(215)-C(216)-C(217)-C(218)	1(2)
C(214)-C(213)-C(218)-C(217)	-2.1(17)
C(212)-C(213)-C(218)-C(217)	-178.9(12)
C(216)-C(217)-C(218)-C(213)	1(2)
C(202)-C(201)-C(219)-C(220)	-172.5(9)
N(201)-C(201)-C(219)-C(220)	75.5(12)
C(202)-C(201)-C(219)-C(224)	1.4(15)
N(201)-C(201)-C(219)-C(224)	-110.6(11)
C(224)-C(219)-C(220)-C(221)	3.5(15)
C(201)-C(219)-C(220)-C(221)	177.7(9)
C(219)-C(220)-C(221)-C(222)	-3.8(16)
C(220)-C(221)-C(222)-C(223)	1.4(16)

C(221)-C(222)-C(223)-C(224)	1.0(17)
C(222)-C(223)-C(224)-C(219)	-1.3(16)
C(220)-C(219)-C(224)-C(223)	-0.9(15)
C(201)-C(219)-C(224)-C(223)	-175.0(10)
C(206)-C(205)-N(201)-C(203)	-68.1(12)
C(206)-C(205)-N(201)-C(201)	-165.2(9)
C(206)-C(205)-N(201)-Pt(21)	58.2(11)
C(204)-C(203)-N(201)-C(205)	102.4(9)
C(202)-C(203)-N(201)-C(205)	-134.4(8)
C(204)-C(203)-N(201)-C(201)	-145.3(8)
C(202)-C(203)-N(201)-C(201)	-22.1(7)
C(204)-C(203)-N(201)-Pt(21)	-28.0(8)
C(202)-C(203)-N(201)-Pt(21)	95.2(6)
C(219)-C(201)-N(201)-C(205)	-90.0(10)
C(202)-C(201)-N(201)-C(205)	138.6(8)
C(219)-C(201)-N(201)-C(203)	153.5(9)
C(202)-C(201)-N(201)-C(203)	22.2(7)
C(219)-C(201)-N(201)-Pt(21)	45.9(10)
C(202)-C(201)-N(201)-Pt(21)	-85.4(6)
C(203)-C(204)-N(202)-C(212)	-172.4(8)
C(203)-C(204)-N(202)-Pt(21)	-36.7(10)
C(213)-C(212)-N(202)-C(204)	55.4(12)
C(213)-C(212)-N(202)-Pt(21)	-74.9(9)
C(204)-N(202)-Pt(21)-N(201)	16.9(7)
C(212)-N(202)-Pt(21)-N(201)	147.6(7)
C(204)-N(202)-Pt(21)-Cl(22)	-165.7(6)
C(212)-N(202)-Pt(21)-Cl(22)	-35.0(6)
C(205)-N(201)-Pt(21)-N(202)	-124.8(7)
C(203)-N(201)-Pt(21)-N(202)	5.7(5)
C(201)-N(201)-Pt(21)-N(202)	101.1(6)
C(205)-N(201)-Pt(21)-Cl(21)	55.2(6)
C(203)-N(201)-Pt(21)-Cl(21)	-174.3(5)
C(201)-N(201)-Pt(21)-Cl(21)	-78.9(6)
C(319)-C(301)-C(302)-C(303)	-148.1(10)
N(301)-C(301)-C(302)-C(303)	-21.8(8)
C(301)-C(302)-C(303)-C(304)	138.3(10)
C(301)-C(302)-C(303)-N(301)	21.8(8)
N(301)-C(303)-C(304)-N(302)	44.4(11)
C(302)-C(303)-C(304)-N(302)	-59.0(11)
N(301)-C(305)-C(306)-C(307)	-94.7(12)
N(301)-C(305)-C(306)-C(311)	88.4(12)
C(311)-C(306)-C(307)-C(308)	0.0(18)
C(305)-C(306)-C(307)-C(308)	-176.9(11)
C(306)-C(307)-C(308)-C(309)	-0.2(19)
C(307)-C(308)-C(309)-C(310)	-1.4(19)
C(308)-C(309)-C(310)-C(311)	3.3(19)
C(307)-C(306)-C(311)-C(310)	1.8(16)
C(305)-C(306)-C(311)-C(310)	178.8(10)
C(309)-C(310)-C(311)-C(306)	-3.5(18)
N(302)-C(312)-C(313)-C(318)	-105.0(12)
N(302)-C(312)-C(313)-C(314)	74.2(14)
C(318)-C(313)-C(314)-C(315)	-0.9(18)
C(312)-C(313)-C(314)-C(315)	179.8(11)
C(313)-C(314)-C(315)-C(316)	0(2)
C(314)-C(315)-C(316)-C(317)	2(2)
C(315)-C(316)-C(317)-C(318)	-3(2)
C(314)-C(313)-C(318)-C(317)	-0.3(18)

C(312)-C(313)-C(318)-C(317)	179.0(12)
C(316)-C(317)-C(318)-C(313)	2(2)
C(302)-C(301)-C(319)-C(324)	-14.0(16)
N(301)-C(301)-C(319)-C(324)	-125.8(10)
C(302)-C(301)-C(319)-C(320)	178.8(11)
N(301)-C(301)-C(319)-C(320)	67.0(14)
C(324)-C(319)-C(320)-C(321)	4.0(17)
C(301)-C(319)-C(320)-C(321)	171.4(10)
C(319)-C(320)-C(321)-C(322)	-2.0(17)
C(320)-C(321)-C(322)-C(323)	-0.1(18)
C(321)-C(322)-C(323)-C(324)	0.2(19)
C(320)-C(319)-C(324)-C(323)	-3.8(16)
C(301)-C(319)-C(324)-C(323)	-171.1(10)
C(322)-C(323)-C(324)-C(319)	1.8(18)
C(306)-C(305)-N(301)-C(303)	-67.1(11)
C(306)-C(305)-N(301)-C(301)	-163.8(9)
C(306)-C(305)-N(301)-Pt(31)	58.7(10)
C(304)-C(303)-N(301)-C(305)	102.5(10)
C(302)-C(303)-N(301)-C(305)	-134.3(9)
C(304)-C(303)-N(301)-C(301)	-144.7(9)
C(302)-C(303)-N(301)-C(301)	-21.5(8)
C(304)-C(303)-N(301)-Pt(31)	-27.3(9)
C(302)-C(303)-N(301)-Pt(31)	95.9(7)
C(319)-C(301)-N(301)-C(305)	-92.5(11)
C(302)-C(301)-N(301)-C(305)	138.5(9)
C(319)-C(301)-N(301)-C(303)	150.9(9)
C(302)-C(301)-N(301)-C(303)	21.8(9)
C(319)-C(301)-N(301)-Pt(31)	44.0(10)
C(302)-C(301)-N(301)-Pt(31)	-85.0(8)
C(313)-C(312)-N(302)-C(304)	57.8(11)
C(313)-C(312)-N(302)-Pt(31)	-72.0(11)
C(303)-C(304)-N(302)-C(312)	-175.5(8)
C(303)-C(304)-N(302)-Pt(31)	-38.5(9)
C(312)-N(302)-Pt(31)-N(301)	149.1(8)
C(304)-N(302)-Pt(31)-N(301)	18.4(6)
C(312)-N(302)-Pt(31)-Cl(32)	-33.8(8)
C(304)-N(302)-Pt(31)-Cl(32)	-164.5(6)
C(305)-N(301)-Pt(31)-N(302)	-126.4(7)
C(303)-N(301)-Pt(31)-N(302)	4.1(6)
C(301)-N(301)-Pt(31)-N(302)	98.4(6)
C(305)-N(301)-Pt(31)-Cl(31)	52.5(6)
C(303)-N(301)-Pt(31)-Cl(31)	-177.1(6)
C(301)-N(301)-Pt(31)-Cl(31)	-82.8(5)

Symmetry transformations used to generate equivalent atoms:

Table 7. Hydrogen bonds for ***(rac)*-7** [Å and °].

D-H...A	d(D-H)	d(H...A)	d(D...A)	<(DHA)
N(2)-H(2C)...Cl(21)	0.92(2)	2.50(3)	3.363(9)	156(4)
N(2)-H(2C)...Cl(22)	0.92(2)	2.73(4)	3.365(10)	127(4)
N(102)-H(12C)...Cl(31)#1	0.901(18)	2.52(2)	3.365(9)	157(4)
N(102)-H(12C)...Cl(32)#1	0.901(18)	2.83(3)	3.443(9)	127(3)
N(202)-H(22C)...Cl(1)#2	0.91(2)	2.57(4)	3.375(8)	148(5)
N(202)-H(22C)...Cl(2)#2	0.91(2)	2.68(4)	3.387(10)	136(4)
N(302)-H(32C)...Cl(11)#3	0.91(2)	2.60(5)	3.398(10)	146(7)

N(302)-H(32C)...Cl(12)#3	0.91(2)	2.75(4)	3.413(9)	130(4)

Symmetry transformations used to generate equivalent atoms:
#1 x,y-1,z   #2 x,y+1,z   #3 x-1,y+1,z

References

1. (a) J. A. Ellman, T. D. Owens and T. P. Tang, *Acc. Chem. Res.*, 2002, **35**, 984-995; (b) M. T. Robak, M. A. Herbage and J. A. Ellman, *Chem. Rev.*, 2010, **110**, 3600-3740.
2. (a) A. Feula, L. Male and J. S. Fossey, *Org. Lett.*, 2010, **12**, 5044-5047; (b) A. Feula, S. S. Dhillon, R. Byravan, M. Sangha, R. Ebanks, M. A. Hama Salih, N. Spencer, L. Male, I. Magyary, W.-P. Deng, F. Müller and J. S. Fossey, *Org. Biomol. Chem.*, 2013, **11**, 5083-5093.
3. (a) T. Moragas, I. Churcher, W. Lewis and R. A. Stockman, *Org. Lett.*, 2014, **16**, 6290-6293; (b) J. L. G. Ruano, I. Fernandez, M. D. Catalina and A. A. Cruz, *Tetrahedron-Asymmetr.*, 1996, **7**, 3407-3414.
4. N. Plobeck and D. Powell, *Tetrahedron Asymmetr.*, 2002, **13**, 303-310.
5. T. Moragas, I. Churcher, W. Lewis and R. A. Stockman, *Org. Lett.*, 2014, **16**, 6290-6293.
6. D. A. Petrone, H. Yoon, H. Weinstabl and M. Lautens, *Angew. Chem., Int. Ed.*, 2014, **53**, 7908-7912.
7. X.-W. Sun, M.-H. Xu and G.-Q. Lin, *Org. Lett.*, 2006, **8**, 4979-4982.
8. X.-W. Sun, M. Liu, M.-H. Xu and G.-Q. Lin, *Org. Lett.*, 2008, **10**, 1259-1262.
9. R. Almansa, J. F. Collados, D. Guijarro and M. Yus, *Tetrahedron Asymmetr.*, 2010, **21**, 1421-1431.
10. (a) M. Sugiura, K. Hirano and S. Kobayashi, *J. Am. Chem. Soc.*, 2004, **126**, 7182-7183; (b) M. Sugiura, C. Mori and S. Kobayashi, *J. Am. Chem. Soc.*, 2006, **128**, 11038-11039; (c) P. V. Ramachandran and T. E. Burghardt, *Chem.-Eur. J.*, 2005, **11**, 4387-4395.
11. R. A. Fernandes and J. L. Nallasivam, *Org. Biomol. Chem.*, 2012, **10**, 7789-7800.
12. R. A. Fernandes and D. A. Chaudhari, *Eur. J. Org. Chem.*, 2012, **2012**, 1945-1952.
13. Synthesised in a previous study.
14. A. Feula and J. S. Fossey, *RSC Adv.*, 2013, **3**, 5370-5373.
15. F. K. MacDonald, K. M. M. Carneiro and I. R. Pottie, *Tetrahedron Lett.*, 2011, **52**, 891-893.
16. (a) G. Lai, F. Guo, Y. Zheng, Y. Fang, H. Song, K. Xu, S. Wang, Z. Zha and Z. Wang, *Chem. Eur. Jour.*, 2011, **17**, 1114-1117; (b) D. A. Evans, D. Seidel, M. Rueping, H. W. Lam, J. T. Shaw and C. W. Downey, *J. Am. Chem. Soc.*, 2003, **125**, 12692-12693.
17. B. V. Subba Reddy and J. George, *Tetrahedron Asymmetr.*, 2011, **22**, 1169-1175.
18. R. Boobalan, G.-H. Lee and C. Chen, *Adv. Synth. Catal.*, 2012, **354**, 2511–2520.
19. J.-L. Li, L. Liu, Y.-N. Pei and H.-J. Zhu, *Tetrahedron*, 2014, **70**, 9077-9083.
20. H. Vázquez-Villa, S. Reber, M. A. Ariger and E. M. Carreira, *Angew. Chem., Int. Ed.*, 2011, **50**, 8979-8981.
21. O. Soltani, M. A. Ariger, H. Vázquez-Villa and E. M. Carreira, *Org. Lett.*, 2010, **12**, 2893-2895.
22. (a) M. S. Taylor and E. N. Jacobsen, *Angew. Chem., Int. Ed.*, 2006, **45**, 1520-1543; (b) X. H. Yu and W. Wang, *Chem.-Asian J.*, 2008, **3**, 516-532.
23. L. Menguy and F. Couty, *Tetrahedron-Asymmetr.*, 2010, **21**, 2385-2389.
24. L. Zhang, M.-M. Lee, S.-M. Lee, J. Lee, M. Cheng, B.-S. Jeong, H.-g. Park and S.-s. Jew, *Adv. Synth. Catal.*, 2009, **351**, 3063-3066.
25. These preliminary findings are by no means complete, and should be seen only as indicative of the organocatalytic potential, full details will be reported in due course, once comprehensive studies are concluded.
26. CrysAlisPro, 2013, **Version 1.171.36.28**, Agilent Technologies.
27. G. M. Sheldrick, *Acta Crystallogr., Sect. A*, 2008, **A64**, 112-122.
28. O. V. Dolomanov, L. J. Bourhis, R. J. Gildea, J. A. K. Howard and H. Puschmann, *J. Appl. Crystallogr.*, 2009, **42**, 339-341.
29. G. M. Sheldrick, *Acta Crystallogr A*, 2015, **71**, 3-8.
30. S. J. Coles and P. A. Gale, *Chem. Sci.*, 2012, **3**, 683-689.
31. R. W. W. Hooft, *COLLECT Data Collection Software*, 1998, ed. Nonius, B.V., Delft, The Netherlands.
32. Z. Otwinowski and W. Minor, eds. C. W. Carter and R. M. Sweet, Academic Press, New York, 1997, vol. 276, pp. 307-326.
33. G. M. Sheldrick, *SADABS*, Bruker AXS Inc., Madison, Wisconsin, USA, 2007.
34. M. C. Burla, R. Caliandro, M. Camalli, B. Carrozzini, G. L. Cascarano, L. De Caro, G. Giacovazzo, G. Polidori and R. Spagna, *J. Appl. Crystallogr.*, 2005, **38**, 381-388.
